# Identification of exosome protein panels as predictive biomarkers for non-small cell lung cancer

**DOI:** 10.1186/s12575-023-00223-0

**Published:** 2023-11-13

**Authors:** Bin Luo, Zujun Que, Xinyi Lu, Dan Qi, Zhi Qiao, Yun Yang, Fangfang Qian, Yi Jiang, Yan Li, Ronghu Ke, Xiaoyun Shen, Hua Xiao, Hegen Li, Erxi Wu, Jianhui Tian

**Affiliations:** 1 Clinical Oncology Center, Shanghai Municipal Hospital of Traditional Chinese Medicine, Shanghai University of Traditional Chinese Medicine, Shanghai, 200071 China; 2 Department of Oncology, Longhua Hospital, Shanghai University of Traditional Chinese Medicine, Shanghai, 200032 China; 3 Institute of Oncology, Shanghai Municipal Hospital of Traditional Chinese Medicine, Shanghai University of Traditional Chinese Medicine, Shanghai, 200071 China; 4 Department of Neurosurgery and Neuroscience Institute, Baylor Scott & White Health, Temple, TX 76502 USA; 5 Department of Neurosurgery, Baylor College of Medicine, Temple, TX 76508 USA; 6 State Key Laboratory of Microbial Metabolism, Joint International Research Laboratory of Metabolic & Developmental Sciences, School of Life Sciences and Biotechnology, Shanghai Jiao Tong University, Shanghai, 200240 China; 7 Prism Genomic Medicine, Sugar Land, TX 77478 USA; 8 School of Medicine, Texas A&M University, College Station, TX 77843 USA; 9 Irma Lerma Rangel School of Pharmacy, Texas A&M University, College Station, TX 77843 USA; 10 LIVESTRONG Cancer Institutes and Department of Oncology, Dell Medical School, The University of Texas at Austin, Austin, TX 78712 USA

**Keywords:** Exosomes, Proteomics, Biomarkers, Non-small cell lung cancer (NSCLC), Metastasis

## Abstract

**Background:**

Non-small cell lung cancer (NSCLC) remains a leading cause of cancer-related deaths worldwide, primarily due to its propensity for metastasis. Patients diagnosed with localized primary cancer have higher survival rates than those with metastasis. Thus, it is imperative to discover biomarkers for the early detection of NSCLC and the timely prediction of tumor metastasis to improve patient outcomes.

**Methods:**

Here, we utilized an integrated approach to isolate and characterize plasma exosomes from NSCLC patients as well as healthy individuals. We then conducted proteomics analysis and parallel reaction monitoring to identify and validate the top-ranked proteins of plasma exosomes.

**Results:**

Our study revealed that the proteome in exosomes from NSCLC patients with metastasis was distinctly different from that from healthy individuals. The former had larger diameters and lower concentrations of exosomes than the latter. Furthermore, among the 1220 identified exosomal proteins, we identified two distinct panels of biomarkers. The first panel of biomarkers (FGB, FGG, and VWF) showed potential for early NSCLC diagnosis and demonstrated a direct correlation with the survival duration of NSCLC patients. The second panel of biomarkers (CFHR5, C9, and MBL2) emerged as potential biomarkers for assessing NSCLC metastasis, of which CFHR5 alone was significantly associated with the overall survival of NSCLC patients.

**Conclusions:**

These findings underscore the potential of plasma exosomal biomarkers for early NSCLC diagnosis and metastasis prediction. Notably, CFHR5 stands out as a promising prognostic indicator for NSCLC patients. The clinical utility of exosomal biomarkers offers the potential to enhance the management of NSCLC.

**Supplementary Information:**

The online version contains supplementary material available at 10.1186/s12575-023-00223-0.

## Introduction

Despite significant advances in cancer control, the overall survival and quality of life for lung cancer patients have not seen substantial improvements [1–3]. Lung cancer remains a leading cause of cancer-related deaths worldwide [1, 4]. Non-small-cell lung cancer (NSCLC) accounts for approximately 85% of all new lung cancer cases and is characterized by high heterogeneity. Early screening efforts, particularly among high-risk individuals such as smokers, have led to a significant increase in lung cancer diagnoses, with an estimated 3.8 million new cases expected by 2050 [2, 5]. The 5-year survival rates for lung cancer range from 4 to 17% depending on the stage and regional disparities, and it is projected that there will be 3.2 million deaths attributed to lung cancer globally over the next three decades [2, 6].

Surgery is the standard treatment for early-stage lung cancer; however, the challenge lies in the potential for local recurrence or the development of distant metastases, which are typically incurable. The process of metastasis, where cancer cells spread from the lungs to other organs, represents the most devastating and fatal aspect of lung cancer [7]. It is worth noting that the majority of lung cancer-related deaths occur due to metastatic disease rather than the primary tumors themselves. Metastasis involves a complex series of biological events that begin with the invasion of cells from the primary tumor into surrounding tissues, penetrating the mucosa [8]. These cells then disseminate through the bloodstream, lymphatic system, or neighboring structures. Subsequently, secondary tumors are established in distant organs, where they continue to grow and colonize [9, 10]. The progression of metastasis relies on tumor cells acquiring various phenotypic states and manipulating the immune and stromal cells in their environment to promote growth and evade immune surveillance [11]. Unlike primary tumors, which can often be effectively treated with localized therapies such as surgery or radiation, metastatic cancer is a systemic disease that affects multiple organs [9]. It can compromise organ function either by directly colonizing them or by altering their metabolism through changes in secreted molecules. Ultimately, these disruptions contribute to the deterioration of the patient's condition. Importantly, the response to systemic treatments can significantly differ between primary and metastatic tumors within the same individual [12]. Despite some exceptions, clinically detectable metastasis remains largely incurable due to the acquired resistance of metastatic tumors to currently available therapies [13]. Currently, there is a lack of effective biomarkers for predicting metastasis in early postoperative lung cancer patients.

Noninvasive biomarkers play an indispensable role in the early detection of lung cancer and predicting the likelihood of recurrence or metastasis, constituting a significant clinical challenge. In recent years, liquid biopsy has emerged as a promising approach for the noninvasive detection of lung cancer [14]. For instance, Ilie et al. utilized computed tomography to track the counts of circulating tumor cells (CTCs) in peripheral blood from chronic obstructive pulmonary disease patients over a five-year period. They found that CTC counts in peripheral blood can serve as a useful marker to predict the progression from chronic lung disease to lung cancer [15]. Despite the growing body of research on CTCs, substantial clinical evidence supporting their utility as biomarkers for guiding lung cancer treatment is still lacking [16]. Moreover, accurate noninvasive biomarkers that can differentiate between healthy individuals and those with nonmetastatic or metastatic cancers remain elusive. Thus, there is an imperative need to identify valid biomarkers to manage the diagnosis and treatment of lung cancer.

Extracellular vesicles (EVs), particularly exosomes, have emerged as valuable tools in liquid biopsy and offer significant advantages in predicting cancer and metastasis [17]. Originating from endosomes and ranging from approximately 40–160 nm in size, exosomes encapsulate active proteins, phosphoproteins, lipids, and genetic materials (DNA and RNA), known as cargo, that can provide more specific and sensitive representations of the disease state compared to circulating proteins in serum [18–22]. These contents, shielded by a lipid bilayer, offer stability in the bloodstream, making exosomes particularly suitable for detection and analysis. As an illustrative example, the Kalluri group discovered that glypican 1 (GPC1)^+^ exosomes carrying the KRAS mutation in pancreatic cancer patients precisely mirrored mutations found in the tumor tissues, underscoring the heightened specificity exosomes can offer over traditional serum or plasma measurements. Indeed, several independent laboratories have reported that in the diagnosis of pancreatic, breast, and colon cancer, GPC1 is enriched in cancer cell–derived exosomes, thus enabling the detection of cancer and possibly response to therapy [22–32]. Exosomal cargo can mirror cellular alterations at early stages of the disease, even before they become detectable in systemic circulation or serum. This early detection capability is crucial in improving patient outcomes by enabling interventions at earlier stages of the disease. While serum-based markers might only rise after a disease has reached a certain progression level, the contents of exosomes can signal cellular alterations at much earlier stages. Specificity and earliness are particularly pivotal for conditions such as NSCLC, where early and precise detection can significantly influence patient outcomes.

Previous studies have identified plasma exosomes as potential diagnostic markers for advanced NSCLC [33–35]. For example, Peng et al. identified plasma-derived exosomal microRNAs as potential biomarkers for predicting the efficacy of immunotherapy in advanced NSCLCs [36]. Another study highlighted the role of FAM3C in circulating tumor-derived extracellular vesicles, promoting NSCLC growth in secondary sites [37]. These findings suggest that exosomes hold promise as biomarkers for lung cancer metastasis and recurrence. Despite these advancements, the translation of these findings into clinical applications is yet to be realized. In this study, acknowledging the challenges in methodological standardization, we employed a set of measures, including nanoparticle tracking analysis (NTA), transmission electron microscopy (TEM), exosome marker protein detection and liquid chromatography tandem mass spectrometry (LC–MS/MS)-based tandem mass tag (TMT) quantitative proteomics, to identify exosome-carried proteins as potential biomarkers for predicting NSCLC metastasis. We validated these candidate proteins using parallel reaction monitoring (PRM) in clinical samples.

## Methods

### Patient samples

The study protocol received ethical approval from the ethics committee of Longhua Hospital, Shanghai University of Traditional Chinese Medicine, China. Prior to participating in the study, all patients provided written informed consent for the collection of plasma samples and the utilization of their pathological data. Plasma samples were obtained from patients diagnosed with stage Ia-IV NSCLC, including both adenocarcinoma (ADC) and squamous cell carcinoma (SCC), using CT scans and established pathological diagnosis criteria. Sample collection took place at the Department of Oncology, Longhua Hospital, Shanghai, China, from May 2019 to January 2020.

A control group consisting of 6 healthy individuals was included in the study. These individuals were confirmed to be cancer-free based on CT scans and, when applicable, negative biopsy results. Briefly, venous blood was collected from the participants using an evacuated blood collection tube containing ethylenediaminetetraacetic acid (EDTA). The collected samples were allowed to stand for 30 min and then subjected to centrifugation at 4,000 × g for 30 min to remove cell debris and platelets. Finally, the plasma samples were stored at -80 °C for further analysis.

### Plasma exosome isolation

To isolate exosomes from the plasma samples, the following protocol was employed. One milliliter of a relatively cell-free plasma sample was thawed on ice and diluted 20 times with phosphate-buffered saline (PBS). The diluted samples were then subjected to centrifugation at 10,000 × g for 30 min at 4 °C to remove microvesicles. The resulting supernatant was carefully transferred to ultracentrifuge tubes and subjected to ultracentrifugation at 110,000 × g for 2 h at 4 °C. This step aimed to pellet the exosomes.

Following ultracentrifugation, the exosome pellet was washed with 6 mL of cold PBS and subjected to a second round of ultracentrifugation at 110,000 × g for 2 h at 4 °C. This step ensured the removal of contaminants and further concentrated the exosomes. After the second ultracentrifugation, the pelleted exosomes were resuspended in 100 μL of PBS containing a protease inhibitor cocktail (Roche Applied Science, Basel, Switzerland). Finally, the resuspended exosomes were stored at -80 °C for further analysis [38].

### Sodium dodecyl sulfate–polyacrylamide gel electrophoresis (SDS-PAGE)

For the analysis of exosome proteins, a 10% SDS-PAGE gel (InvitrogenTM, Thermo Fisher, New York, USA) was prepared. The exosome protein samples were loaded onto the gel and subjected to electrophoresis at 120 V for 60 min in MOPS SDS running buffer. To monitor the migration of proteins, a prestained protein standard (Life Technologies, Shanghai, China) was included in one of the lanes. After electrophoresis, the gels were stained using a Fast Silver Stain Kit (Beyotime, Beijing, China) to visualize the protein bands and achieve optimal contrast for protein detection.

### Exosome characterization by transmission electron microscopy (TEM)

Transmission electron microscopy (TEM) analysis was employed to examine the plasma vesicles. To prepare the samples, isolated exosomes (10 μL) were diluted 10 times with PBS. Subsequently, the diluted exosomes were carefully loaded onto an ultrathin carbon film mesh 300 copper grid and allowed to dry for fixation. To enhance contrast and facilitate visualization, the exosomes on the grid were stained with 2% phosphotungstic acid for 5 min. This staining step aided in highlighting the structural features of the exosomes during imaging. The images of the exosomes were captured using a Philips CM120 microscope (Eindhoven, Netherlands), which operated at an acceleration voltage of 120 kV. TEM provided high-resolution images, enabling detailed examination of exosome morphology and structure.

### Western blotting analysis

In the analysis of exosome proteins, the following experimental steps were performed. First, the exosomes were lysed using RIPA buffer for 30 min at 4 °C, enabling the release of proteins from the exosomes. Next, 25 μg of exosome proteins were loaded onto 10% SDS-PAGE gels (Invitrogen, Thermo Fisher, New York, USA). The gels were then subjected to electrophoresis at 120 V for 60 min in MOPS SDS running buffer. This process allowed for the separation of the proteins based on their molecular weight. Following electrophoresis, the proteins present in the gel were transferred onto polyvinylidene fluoride (PVDF) membranes (Millipore, Massachusetts, USA) using a transfer apparatus. The transfer was conducted at 200 mA for 1 h, facilitating the transfer of proteins from the gel to the membrane. To visualize the protein bands, the resulting gels were stained using a Fast Silver Stain Kit (Beyotime, Beijing, China), which provided optimal contrast for protein detection. To identify specific proteins of interest, antibodies were utilized. The antibodies used in this study were obtained from Abcam and included CD63 (1:500) and CD81 (1:500). After washing the PVDF membrane to remove any unbound proteins, it was incubated with an anti-rabbit IgG-HRP secondary antibody (Jackson Laboratory, USA) in 5% milk in PBS-T at room temperature for 40 min. This secondary antibody aided in the detection of primary antibody binding. For visualization of the detected proteins, enhanced chemiluminescence (ECL) using Super Signal West Pico (Thermo) was employed. This chemiluminescent substrate generated a signal in the presence of the HRP-conjugated secondary antibody, enabling the visualization of the protein bands.

### Nanoparticle tracking analysis (NTA)

The purified exosomes obtained from serum samples were resuspended in 100 μL of PBS buffer. To facilitate accurate measurement, the exosomes were further diluted 10 times with PBS. The number and size of the exosomes were then assessed using the NanoSight NS300 NTA system (Malvern, United Kingdom), which utilizes nanoparticle tracking analysis (NTA). For NTA analysis, the exosome samples were carefully resuspended in PBS and injected into the sample chamber of the NanoSight instrument. Each sample was measured three times to ensure reliable and reproducible data. The NanoSight system utilizes laser light scattering and particle tracking technology to observe and track the Brownian motion of individual exosomes. By analyzing the particle movement, the system provides information about the size and concentration of the exosomes in the sample.

### Protein preparation

To process the exosome sample for further analysis, the following steps were performed. First, the exosome sample was ground using liquid nitrogen until it formed a cell powder. The cell powder was then transferred to a 5-mL centrifuge tube. Next, four volumes of lysis buffer containing 8 M urea, 1% Triton-100, 10 mM dithiothreitol, and 1% protease inhibitor cocktail were added to the cell powder. To enhance the lysis process, sonication was performed three times on ice using a high-intensity ultrasonic processor (Scientz).

After sonication, the mixture was subjected to centrifugation at 20,000 g at 4 °C for 10 min to remove any remaining debris. To precipitate the protein, the supernatant was discarded, and the remaining solution was treated with cold 20% trichloroacetic acid (TCA) for 2 h at -20 °C. Following this, centrifugation was carried out at 12,000 g and 4 °C for 10 min, and the supernatant was discarded. The protein precipitate was then washed three times with cold acetone.

To prepare the protein for further analysis, the precipitated protein was redissolved in 8 M urea. The protein concentration was determined using a BCA kit following the manufacturer's instructions (Thermo Fisher Scientific. Cat. No. 85165). For digestion, the protein solution was reduced by adding 5 mM dithiothreitol and incubating at 56 °C for 30 min. Then, the protein sample was alkylated with 11 mM iodoacetamide for 15 min at room temperature in darkness. The urea concentration was subsequently reduced to less than 2 M by dilution. Finally, trypsin enzyme was added to the protein solution at a 1:50 trypsin-to-protein mass ratio for the first overnight digestion, followed by a second digestion with a 1:100 trypsin-to-protein mass ratio for 4 h.

### Tandem mass tag (TMT)-based quantitative proteomics

After trypsin digestion, the peptide was desalted by a Strata X C18 SPE column (Phenomenex) and vacuum dried. The peptide was reconstituted in 0.5 M TEAB and processed according to the manufacturer’s protocol for the TMT kit. Briefly, one unit of TMT reagents was thawed and reconstituted in acetonitrile. The peptide mixtures were then incubated for 2 h at room temperature, pooled, desalted, and dried by vacuum centrifugation.

The tryptic peptides were fractionated by high pH reverse-phase high-performance liquid chromatography (HPLC) using a Thermo Betasil C18 column (5 μm particles, 10 mm ID, 250 mm length). Briefly, peptides were first separated with a gradient of 8% to 32% acetonitrile (pH 9.0) over 60 min into 60 fractions. Then, the peptides were combined into 12 fractions and dried by vacuum centrifugation. The tryptic peptides were dissolved in 0.1% formic acid (solvent A) and directly loaded onto a homemade reversed-phase analytical column. The gradient was comprised of an increase from 6 to 23% solvent B (0.1% formic acid in 98% acetonitrile) over 38 min, 23% to 35% in 14 min and climbing to 80% in 4 min, then holding at 80% for the last 4 min, at a constant flow rate of 400 nL/min on an EASY-nLC 1000 ultra-performance liquid chromatography (UPLC) system.

The peptides were subjected to an NSI source followed by tandem mass spectrometry (MS/MS) in Q ExactiveTM Plus (Thermo, USA) coupled online to the UPLC. The electrospray voltage applied was 2.0 kV. The m/z scan range was 350 to 1000 for a full scan, and intact peptides were detected in the Orbitrap at a resolution of 35,000. Peptides were then selected for MS/MS using the normalized collision energy (NCE) setting as 27, and the fragments were detected in the Orbitrap at a resolution of 17,500. A data-independent procedure that alternated between one MS scan followed by 20 MS/MS scans. Automatic gain control (AGC) was set at 3E6 for full MS and 1E5 for MS/MS. The maximum IT was set at 20 ms for full MS and auto for MS/MS. The isolation window for MS/MS was set at 2.0 m/z.

The resulting MS/MS data were processed using the MaxQuant search engine (v.1.5.2.8). Tandem mass spectra were searched against the human UniProt database concatenated with the reverse decoy database. Trypsin/P was specified as a cleavage enzyme, allowing up to 2 missing cleavages. The mass tolerance for precursor ions was set as 20 ppm in the first search and 5 ppm in the main search, and the mass tolerance for fragment ions was set as 0.02 Da. Carbamidomethyl on Cys was specified as a fixed modification, and acetylation modification and oxidation on Met were specified as variable modifications. The false discovery rate (FDR) was adjusted to < 1%, and the minimum score for modified peptides was set to > 40.

### Parallel reaction monitoring (PRM)

Protein candidates with a more than 1.2-fold change and an adjusted *P*-value of less than 0.05 were selected for further validation by targeted liquid chromatography-parallel reaction monitoring (LC-PRM) MS. The MS parameters for peptide identification were the same as described above.

The PRM data analysis was processed using Skyline (v.3.6). Peptide settings: enzyme was set as trypsin [KR/P]; maximum missed cleavage was set as 2; the peptide length was set as 8–25, variable modification was set as carbamidomethyl on Cys and oxidation on Met, and max variable modifications were set as 3. Transition settings: precursor charges were set as 2 and 3, ion charges were set as 1 and 2, and ion types were set as b, y, and p. The productions were set as from ion 3 to the last ion, and the ion match tolerance was set as 0.02 Da. The criteria of two unique peptides, *p* < 0.05 and FDR less than 1% at the protein level, were used for protein identification.

### Survival analysis of NSCLC patients using datasets from the public database

The prognostic value of mRNA expression of discovered exosomal proteins in lung cancer was analyzed using the public microarray database Kaplan–Meier Plot (www.kmplot.com) with aggregate human patient data [39]. Lung cancer patients were divided into high and low expression groups in accordance with median expression. Information about overall survival (OS), progression-free survival (PFS), postprogression survival (PPS), the hazard ratio (HR) with 95% confidence intervals (CIs) and log-rank *P* values can be found at the K-M plotters. Analysis strategies for different combinations of discovered proteins: median, split patients by median, only JetSet best probe set and use mean expression of selected genes. Statistical significance was analyzed using a two–tailed log rank test incorporated in the Kaplan–Meier Plot database, and *p* < 0.05 indicated a statistically significant difference.

### Bioinformatics and statistical analysis

The supplementary materials provide a detailed description of the bioinformatic analysis steps. To identify potential biomarkers, gene set and pathway analyses were conducted using Ingenuity Pathway Analysis (IPA). Statistical significance was determined using appropriate methods, such as t-tests, ANOVA, SNK-q, and receiver operating characteristic (ROC) curve analysis by GraphPad Prism (GraphPad Software, version 8.0, San Diego, CA, USA). The level of significance for the statistical tests was set at 0.05 (*), 0.01 (**) and 0.001 (***), and these values are reported in the figure legends.

## Results

### Characterization of plasma exosomes from NSCLC and healthy individuals

**Fig. 1:**
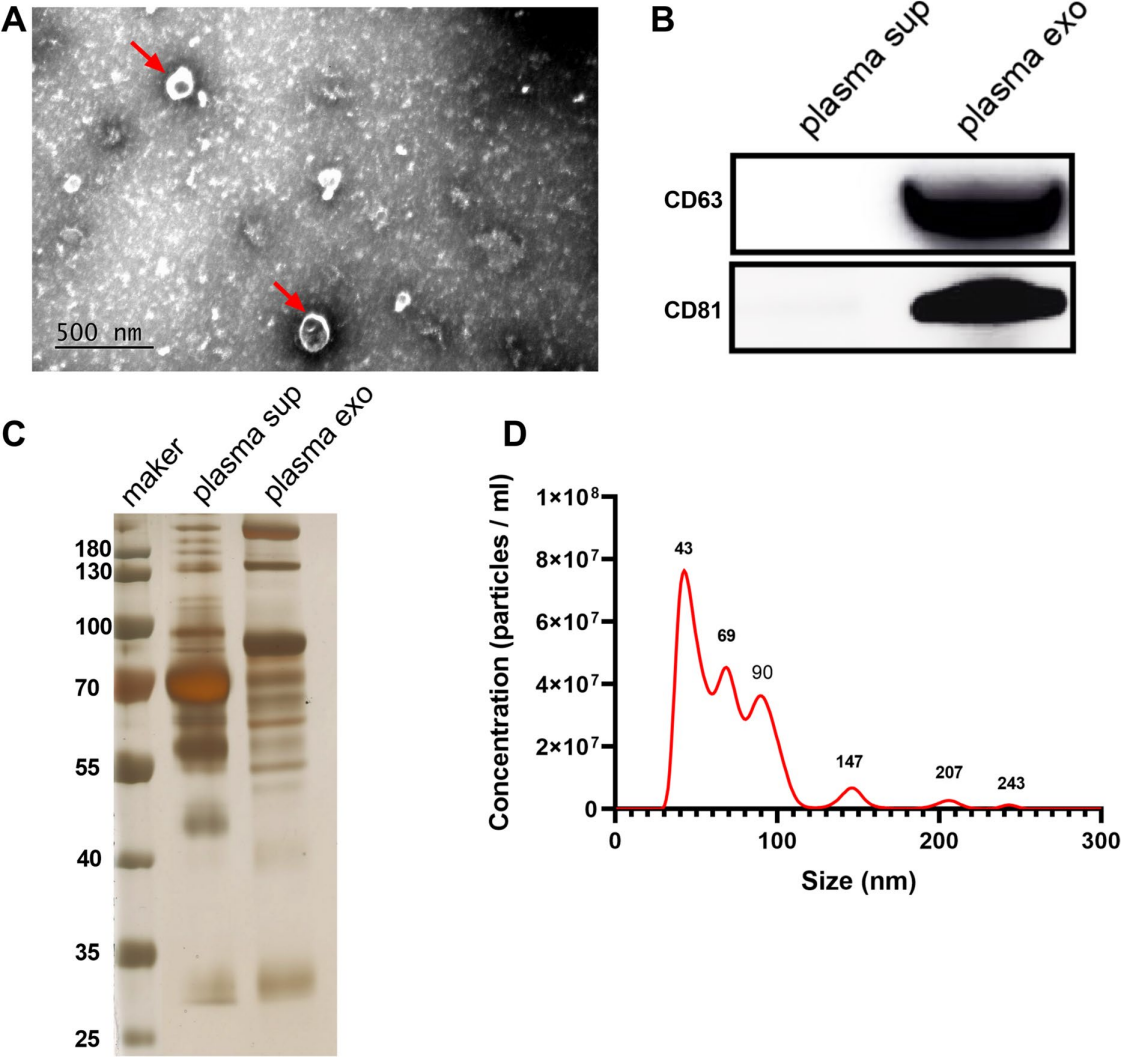
Characteristic proteins and morphology of exosomes. **A** TEM images of exosomes from representative plasma samples. **B** Western blot of CD63 and CD81 in the exosomes of seven representative samples. **C** SDS-PAGE of proteins from plasma supernatant and plasma exosomes. **D** Nanoparticle tracking analysis of the size distribution of representative exosomes

In this study, we analyzed a total of 57 plasma samples from NSCLC patients with metastasis (*n* = 19) and without metastasis (*n* = 32) and healthy subjects (*n* = 6). A detailed overview of the subjects can be found in Table 1. Exosomes were isolated from plasma samples using ultracentrifugation and subsequently characterized using multiple techniques. Transmission electron microscopy (TEM) analysis revealed the distinctive round, cup-shaped, vesicle-like structures of the isolated exosomes, as depicted in Fig. 1A. To confirm the presence of exosomes, immunoblotting was performed to detect exosome marker proteins. The results demonstrated the expression of CD63 and CD81 proteins in the exosome samples, whereas these proteins were not detected in plasma samples, as depicted in Fig. 1B. This confirmed the enrichment of exosomes in the isolated samples. Furthermore, SDS-PAGE analysis revealed significant differences in protein composition between the exosomes and plasma proteins, as illustrated in Fig. 1C. This indicated that the isolated exosomes harbored a distinct protein profile in comparison to the proteins present in plasma. Additionally, nanoparticle tracking analysis (NTA) was employed to determine the size distribution of the exosomes. The NTA results showed that the diameters of the exosomes ranged from approximately 30 to 150 nm, which is consistent with the typical size range of exosomes, as depicted in Fig. 1D. These findings further supported the successful isolation of high-quality exosomes from the plasma samples of the patients. Overall, the combination of TEM, immunoblotting, SDS-PAGE, and NTA analyses provided comprehensive evidence for the successful isolation and characterization of exosomes from patient plasma. This comprehensive characterization confirmed their morphological features, marker protein expression, unique protein composition, and size distribution.

**Table 1:** The clinical information of healthy controls and lung cancer patients

**Characteristics**	**TMT + PRM**	**TMT**		**PRM**	
	**Health Individuals(*n* = 6)**	**Non-metastasis NSCLC (*n* = 17)**	**Metastasis NSCLC(*n* = 7)**	**Non-metastasis NSCLC (*n* = 15)**	**Metastasis NSCLC(*n* = 12)**
**Age**	48.00 ~ 55.00	50.00 ~ 65.00	51.00 ~ 58.00	51.00 ~ 65.00	52.00 ~ 67.00
**Median ± SD**	52.00 ± 2.90	57.47 ± 5.32	55.00 ± 2.58	58.60 ± 4.93	58.92 ± 4.27
**Gender**					
**Male**	3 (50%)	7 (41.18%)	4 (51.14%)	8(53.33%)	7(58.33%)
**Female**	3 (50%)	10 (58.82%)	3 (42.86%)	7(46.67%)	5(41.67%)
**Ethnicity**	Asian	Asian	Asian	Asian	Asian
**Smoking history**					
**Smoking**	2 (33.33%)	7 (31.82%)	5 (71.43%)	3(20.00%)	2(16.67%)
**Non-smoking**	4 (66.67%)	15 (68.18%)	2 (28.57%)	12(80.00%)	10(83.33%)
**Pathological types**					
**ADC**	—	22 (91.67%)	7 (100.00%)	12(80.00%)	11(91.67)
**SCC**	—	2 (8.33%)	0 (0.00%)	3(20.00%)	1(8.33%)

### Exosomes in NSCLC patients with metastasis had larger particle sizes but decreased concentrations

**Fig. 2:**
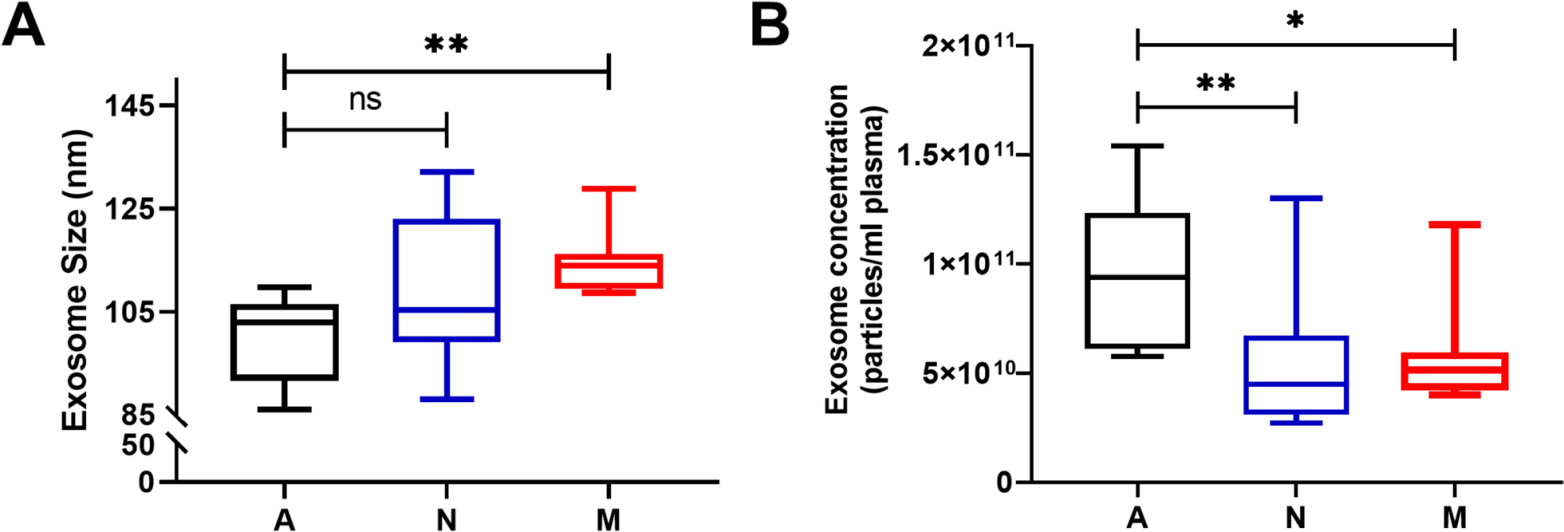
The size and concentration of isolated plasma exosomes in the NSCLC and healthy control groups. **A** The average size of exosomes in the A, N, and M groups. **B** The concentration of exosomes in the A, N, and M groups. (A: healthy control, N: nonmetastatic lung cancer, M: metastatic lung cancer). (SNK test, values of 0.05 (*), 0.01 (**) and 0.001 (***) were assumed as the level of significance for the statistical tests)

Our investigation aimed to discern differences in the physicochemical attributes of plasma exosomes between NSCLC patients and healthy individuals. We employed NTA to assess the particle size and concentration of exosomes. Our findings unveiled notable distinctions in these characteristics between the two groups. Specifically, NSCLC patients exhibited larger particle sizes and lower concentrations of exosomes in comparison to healthy individuals, as shown in Fig. 2A and B. Furthermore, within the NSCLC patient subgroup, those with metastatic conditions displayed even more pronounced increases in particle size (as demonstrated in Fig. 2A). These results suggest that the particle size and concentration of plasma exosomes could potentially serve as important indicators in the early diagnosis of NSCLC.

### Comparative proteome contents of plasma exosomes in NSCLC patients with/without metastasis and healthy individuals

**Fig. 3:**
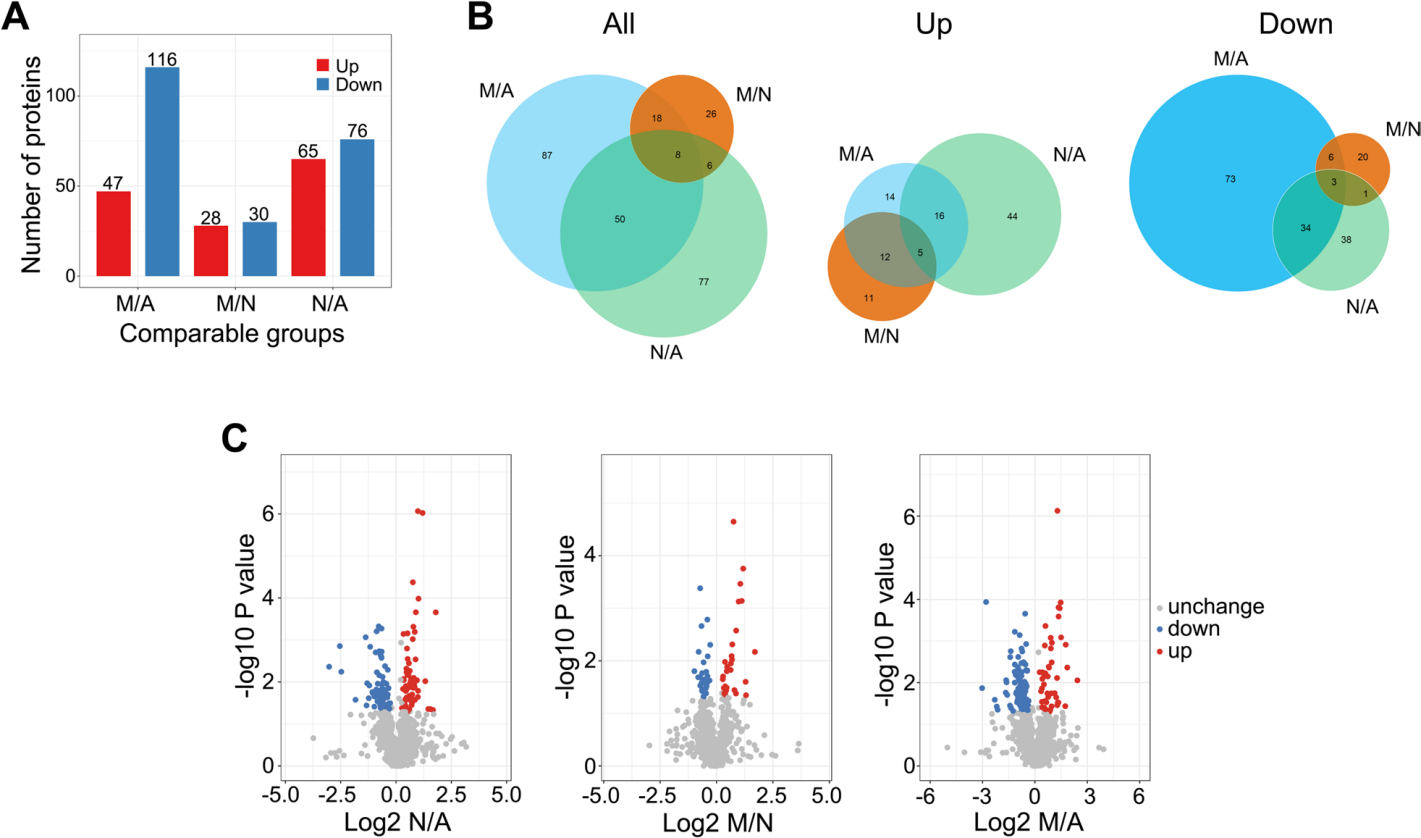
Identification of candidate proteins in plasma exosomes. **A** The histogram results show the downregulated (blue) and upregulated (red) exosome proteins with a fold change greater than 1.2 and *p*-value < 0.05 in groups A, N and M, respectively. **B** Venn diagrams show the differentially expressed proteins among the groups and the overlapping proteins in the A, N, and M groups. Venn diagrams were generated using R. **C** Volcano plots showing the number of proteins identified for each group and proteins shared in each group. (A: healthy control, N: nonmetastatic lung cancer, M: metastatic lung cancer) (SNK test, values of 0.05 (*), 0.01 (**) and 0.001 (***) were assumed as the level of significance for the statistical tests)

In this study, we employed tandem mass tag-based quantitative proteomics technology to analyze the protein composition of plasma exosomes derived from nonmetastatic (N) and metastatic (M) NSCLC patients, as well as healthy individuals (A). A total of 1220 proteins were identified in all plasma exosome samples, of which 1094 proteins were quantifiable (Tab. S1). Significant differential protein expression among the groups was detected, with a threshold set at a fold change of 1.2 (*p* < 0.05), as detailed in Tab. S2. Specifically, 47 proteins were found to be upregulated and 116 proteins were downregulated in the comparison between M and A. Similarly, 28 proteins were upregulated, and 30 proteins were downregulated in the M group when compared with N, while 65 proteins were upregulated, and 76 proteins were downregulated in N compared with A (Fig. 3A). Notably, there were both shared and unique differentially expressed proteins among the groups, as indicated by the Venn diagram analysis (see Fig. 3B and C).

**Fig. 4:**
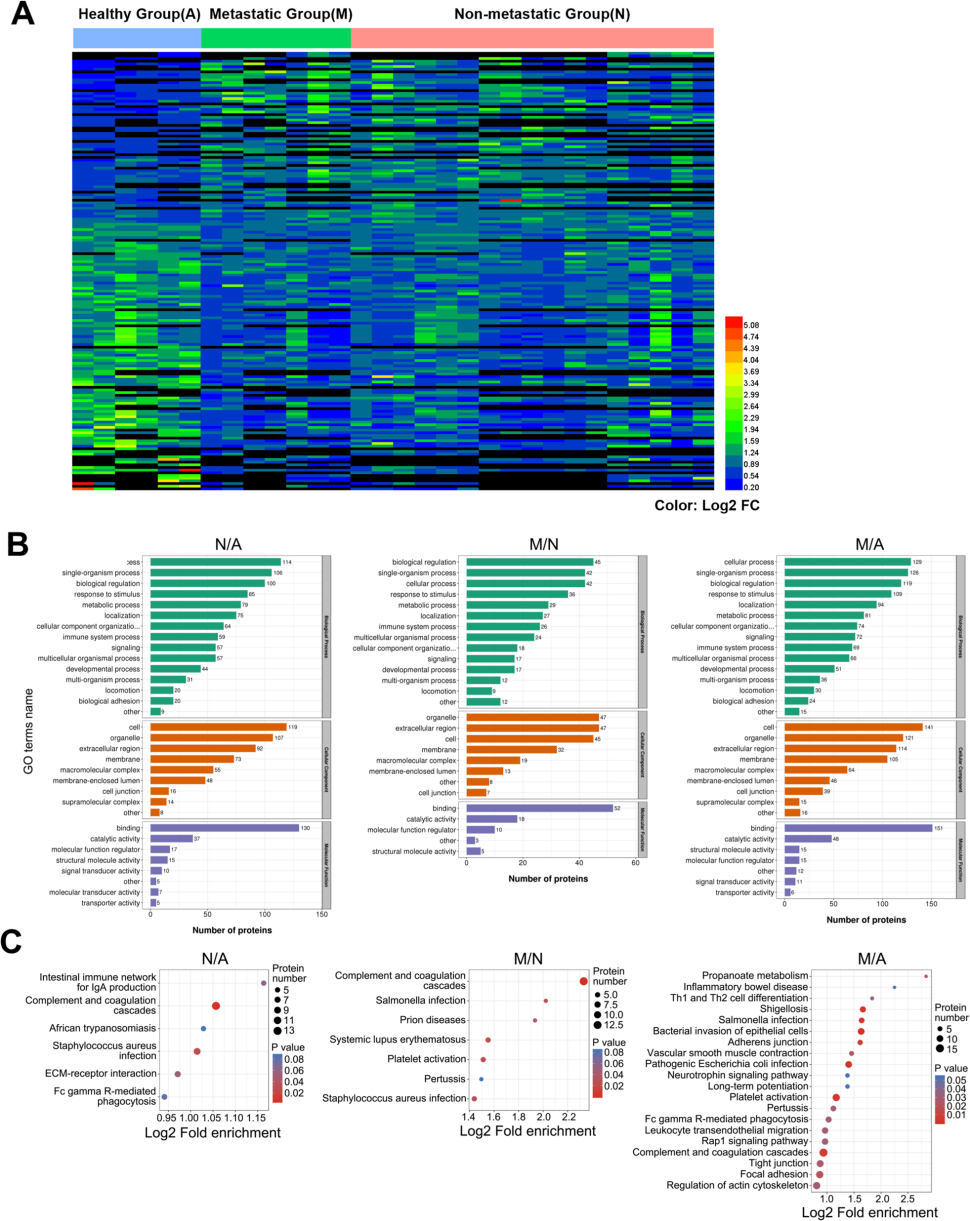
The results showed the major cellular components and main functions of the components. **A** The heat map representing the quantitative analyses of all exosome proteins. **B** The results show the main functions of the components. **C** The major cellular components of isolated exosome proteins. (A: healthy control, N: nonmetastatic lung cancer, M: metastatic lung cancer). (SNK test, values of 0.05 (*), 0.01 (**) and 0.001 (***) were assumed as the level of significance for the statistical tests)

To visualize the patterns of differential protein expression, we conducted heatmap analysis, which clearly delineated distinct expression profiles between the healthy group (A) and the metastatic (M) and nonmetastatic (N) groups (see Fig. 4A). Additionally, we performed Gene Ontology (GO) analysis to explore the functional roles of the differentially expressed proteins (see Figure S1). The results highlighted the predominant involvement of proteins related to the complement and coagulation cascade, platelet activation, and regulation of the immune response in the comparisons of N versus A, M versus N, and M versus A (see Fig. 4B and C). These findings suggest a potentially close association between abnormal coagulation function and the onset and metastasis of lung cancer (see Figure S2). The differential expression of proteins associated with these biological processes underscores their potential significance in the pathogenesis and progression of lung cancer.

### Verification of diagnostic proteins in plasma exosome samples using PRM

**Fig. 5:**
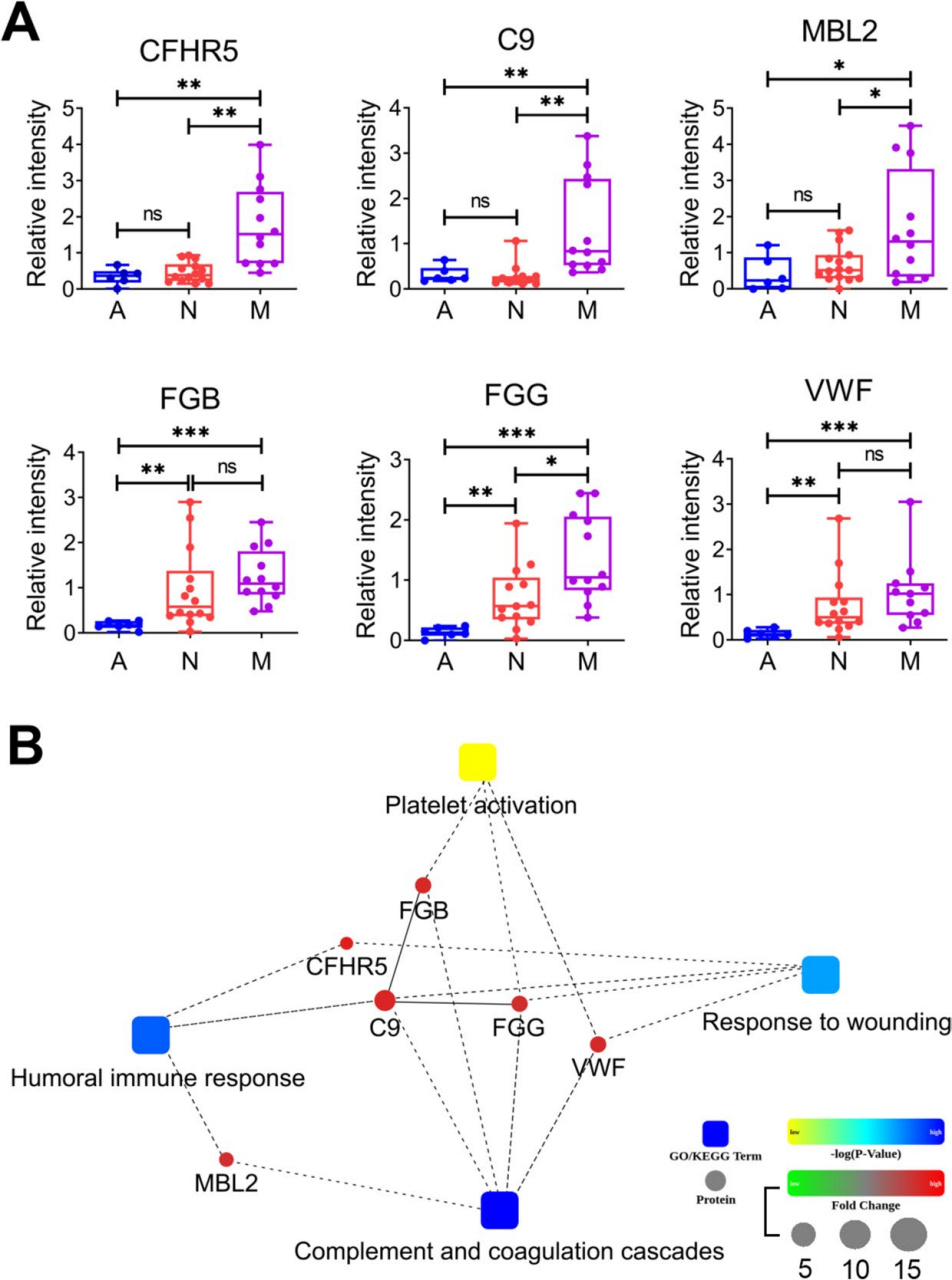
The relative intensity and PPI analysis of the discovered proteins. **A** Relative intensity analysis of the panel proteins to predict metastasis of NSCLC and diagnose early NSCLC. **B** PPI analysis of the six discovered proteins. (A: healthy control, N: nonmetastatic lung cancer, M: metastatic lung cancer). (Values of 0.05 (*), 0.01 (**) and 0.001 (***) were assumed as the level of significance for the statistical tests)

To identify a panel of candidate markers that can serve as indicators of lung cancer occurrence and metastasis, our study harnessed the parallel reaction monitoring (PRM) technique to quantify the expression of 10 selected proteins (detailed in Table 2) within plasma exosomes. This comprehensive analysis involved NSCLC patients with (*n* = 12) and without (*n* = 15) metastasis and healthy individuals (*n* = 6). PRM analysis revealed that recombinant complement factor H-related protein 5 (CFHR5), complement component 9 (C9), and mannose-binding lectin 2 (MBL2) were significantly elevated in NSCLC patients with metastasis compared to nonmetastatic NSCLC patients and healthy individuals (Fig. 5A). However, there was no statistically significant difference observed between nonmetastatic NSCLC patients and healthy individuals (Fig. 5A). Furthermore, the protein levels of fibrinogen beta chain (FGB), fibrinogen gamma chain (FGG), and von Willebrand factor (VWF) were markedly elevated in NSCLC patients, irrespective of metastatic status, when compared to control individuals (as depicted in Fig. 5A). Protein–protein interaction (PPI) analysis revealed that these six selected proteins were primarily associated with key biological processes such as the complement and coagulation cascade, platelet activation, response to wounding, and humoral immune response (as shown in Fig. 5B). Based on these compelling findings, it is suggested that a panel consisting of FGB, FGG, and VWF proteins within plasma exosomes holds potential as a marker for the early diagnosis of NSCLC. The CFHR5, C9, and MBL2 proteins could serve as indicators for assessing the metastatic status of NSCLC patients.

**Table 2:** The selected proteins validated by PRM and discovered proteins

Protein ID	MW [kDa]	Gene Name	M/A			M/N			N/A		
			Ratio (TMT)	Ratio (PRM)	*p*-value (PRM)	Ratio (TMT)	Ratio (PRM)	*p*-value (PRM)	Ratio (TMT)	Ratio (PRM)	*p*-value (PRM)
P02679	51.51	***FGG***	2.78	10.11	0.0009	1.54	1.30	0.3017	1.80	7.79	0.0480
P02675	55.93	***FGB***	2.67	7.41	0.0006	1.55	1.10	0.6350	1.72	6.75	0.0493
Q13201	138.11	MMRN1	1.19	0.94	0.9222	1.27	1.10	0.8581	0.93	0.85	0.7165
P05156	65.75	CFI	1.30	1.38	0.4708	1.61	1.44	0.3402	0.81	0.96	0.9401
P04275	309.26	***VWF***	2.55	9.89	0.0392	1.59	1.34	0.4106	1.61	7.37	0.1001
Q15485	34	FCN2	1.97	3.30	0.0904	1.97	1.25	0.5530	1.30	2.63	0.2956
P02748	63.173	***C9***	1.95	4.21	0.0359	1.62	1.34	0.3978	1.20	3.14	0.2085
Q9BXR6	64.42	***CFHR5***	1.87	5.07	0.0082	1.69	2.71	0.0039	1.11	1.87	0.3566
P11226	26.14	***MBL2***	1.84	4.12	0.0669	1.55	2.49	0.0187	1.19	1.65	0.2736
P43652	69.07	**AFM**	0.86	4.01	0.0880	0.70	2.63	0.0209	1.23	1.52	0.2294

### The predictability of two panels of candidate markers for the early diagnosis and metastasis of NSCLC

**Fig. 6:**
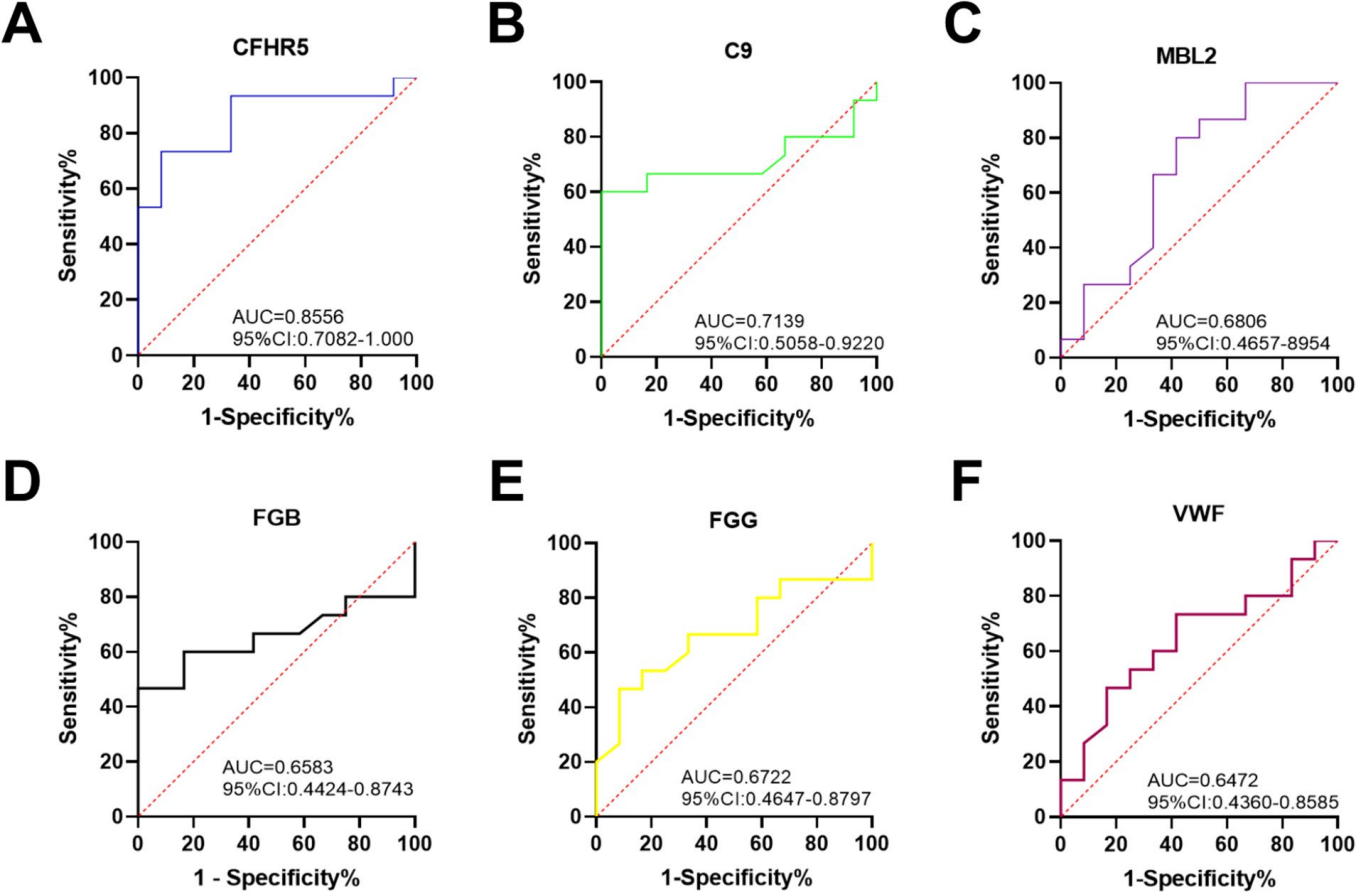
ROC analysis of the identified exosomal proteins. **A** ROC curve analysis of CFHR5. **B** ROC curve analysis of C9. **C** ROC curve analysis of MBL2. **D** ROC curve analysis of FGB. **E** ROC curve analysis of FGG. **F** ROC curve analysis of VWF

To evaluate the diagnostic value of each marker, receiver operating characteristic (ROC) curves were generated. The ROC analysis demonstrated that in plasma exosomes from NSCLC patients with metastasis, CFHR5, C9, and MBL2 exhibited area under the curve (AUC) values of 0.855, 0.713, and 0.680, respectively, when compared to both the healthy control group and nonmetastatic NSCLC (Fig. 6A-C). Moreover, FGB, FGG, and VWF showed AUC values of 0.685, 0.672, and 0.647 (Fig. 6D-F), respectively, in distinguishing NSCLC from the healthy control group.

**Fig. 7:**
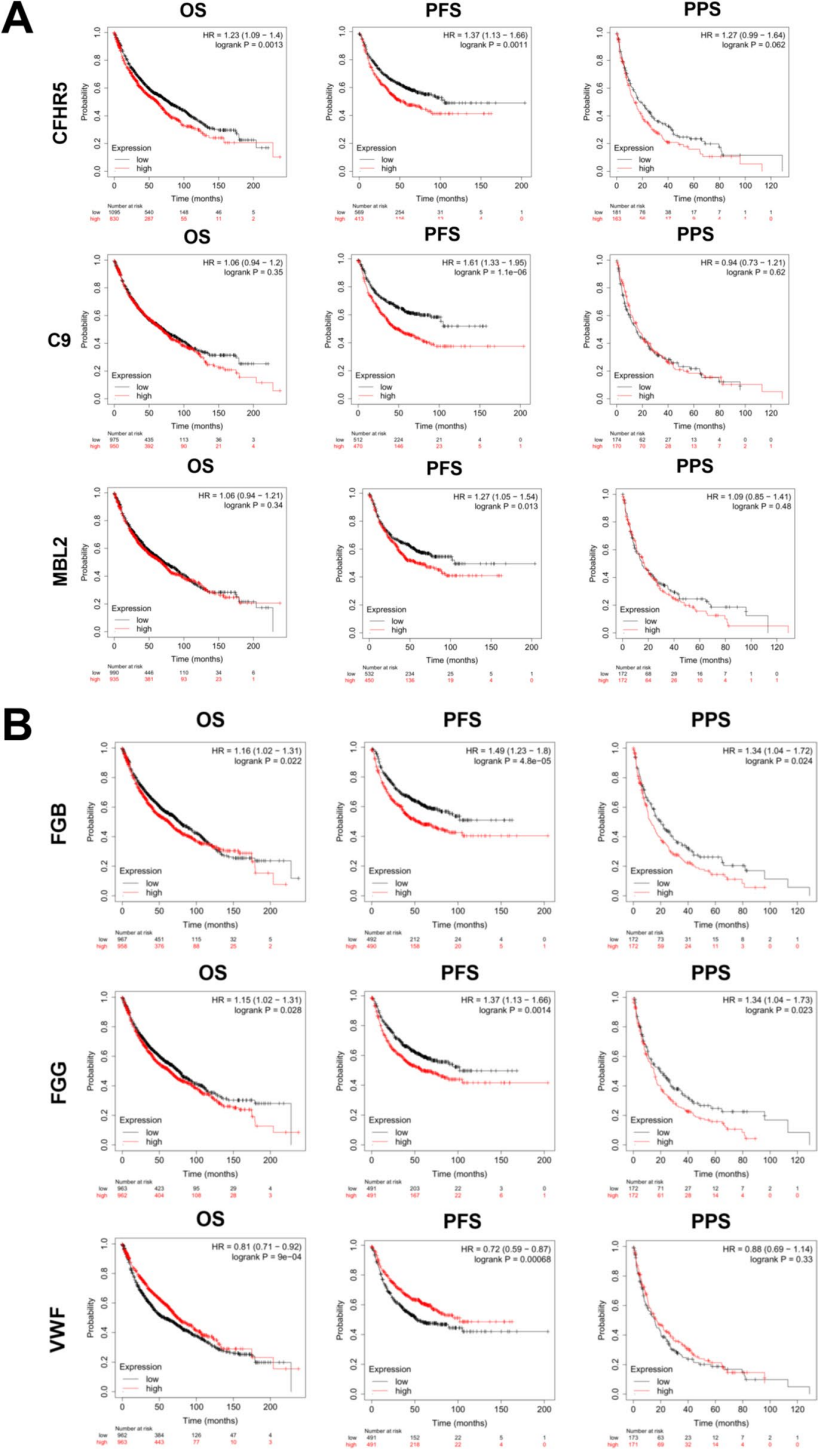
Kaplan–Meier plots of the identified proteins. **A** Survival analyses of CFHR5, C9 and MBL2. **B** Survival analyses of FGG, FGB and VWF. (log-rank test: values of 0.05 (*), 0.01 (**) and 0.001 (***) were assumed as the level of significance for the statistical tests). HR, hazard ratio; OS, overall survival; PFS, progression-free survival; PPS, post-progression survival)

To further assess the clinical significance of mRNA expression, we conducted survival analysis of survival (OS), progression-free survival (PFS), and post-progression survival (PPS) using the publicly available Kaplan–Meier Plot database. The panel of CFHR5, C9, and MBL2 markers exhibited predictive capabilities for PFS in NSCLC patients (*p* < 0.05), and CFHR5 also showed significant predictive value for OS (*p* < 0.05), indicating its potential as a predictive biomarker for metastasis (Fig. 7A). Similarly, we examined the panel of FGB, FGG, and VWF markers, and all three candidates displayed significant predictive abilities for OS/PFS/PPS (*p* < 0.05), except for VWF, which failed to predict PPS (Fig. 7B). These findings suggest that the identified protein markers not only have predictive value for metastasis but also hold potential as biomarkers for early clinical diagnosis. Taken together, the results highlight the significance of the identified proteins as predictive biomarkers for metastasis in lung cancer. The identified panels of markers, including CFHR5, C9, MBL2, FGB, FGG, and VWF, exhibit diagnostic and prognostic capabilities, offering potential utility in guiding clinical decision-making and patient management. These findings emphasize the importance of these proteins as promising candidates for the development of novel and effective biomarkers in lung cancer.

## Discussion

Distant metastasis remains a major cause of mortality in lung cancer patients, and current methods for the early screening and prediction of metastasis using serum tumor markers have limitations in clinical decision-making. Therefore, there is a critical need for more sensitive and perfect biomarkers in clinical practice. Exosome proteins provide new insights for the early screening and metastasis prediction of lung cancer. Exosomal proteins have been reported to be of great clinical value in the diagnosis, therapeutic targets and prediction of the therapeutic efficacy of lung cancer [40]. Nevertheless, there is still a lack of noninvasive clinical biomarkers for the early screening and prediction of metastasis in lung cancer. In this study, we identified two panels of exosomal proteins that can be used as biomarkers for the early screening and prediction of metastasis in lung cancer. KEGG, GO and PPI analyses showed that the biological effects of these identified proteins involved platelet activation, complement system and coagulation cascades, and immune response. This information enhances our understanding of the potential mechanisms underlying lung cancer metastasis and provides valuable insights for clinical applications. At the same time, we validated the clinical predictive ability of the identified proteins for metastasis and early screening via online databases. Although this is validated at the level of serum RNA, studies have suggested that proteins in serum and exosomes have similar potential as biomarkers [41–43]. In short, these identified proteins provide evidence for early screening and therapeutic decision-making of lung cancer in the clinic.

Liquid biopsy is recognized as a non-invasive detection technology. Previous studies have found that long noncoding RNAs and microRNAs in exosomes can promote the progression of lung cancer and may serve as therapeutic targets for lung cancer [44, 45]. However, few studies have been performed on exosome protein as a marker of metastasis prediction. In this study, we first found that exosomes in lung cancer patients with metastasis had a larger size and lower concentration than those in healthy and non-metastatic groups. By TMT and PRM, we identified and validated a previously unreported panel of exosomal proteins, including CFHR5, C9, and MBL2, that can be used to predict metastasis in NSCLC. These identified proteins are associated with immune responses, both adaptive and innate [46–48]. CFHR5, a complement activating protein, has been identified as a significant factor in the development of liver metastasis originating from primary tumors of colorectal carcinoma [46, 49–53]. The present study provides evidence that CFHR5 may serve as a biomarker for the early prediction of metastasis in NSCLC. C9, which serves as a terminal component of the complement pathway, is primarily synthesized in the liver. Previous investigations have revealed that the levels of C9 protein in the plasma of patients with gastric and colorectal cancers exhibit a substantial increase [54, 55]. MBL2 is an essential constituent of the innate immune system and a member of the complement system. The protein recognizes and binds to mannose and N-acetylglucosamine, and this binding activates the classical complement pathway. A recent study discovered that plasma MBL2 levels were higher in the metastatic breast cancer group and were associated with poorer survival outcomes [56]. Similarly, elevated plasma MBL2 levels in colorectal cancer patients have been identified as an indicator of unfavorable patient survival [57]. Together, we have identified a panel of exosomal proteins associated with innate immunity that can predict lung cancer metastasis. The identification of these proteins as both biomarkers and potential therapeutic targets marks a significant advancement in our battle against NSCLC metastasis.

Furthermore, we observed significantly increased levels of FGB, FGG, and VWF proteins in plasma exosomes from NSCLC patients compared to healthy individuals. FGB and FGG, components of the extracellular matrix protein fibrinogen, are crucial for wound healing and hemostasis and function in tumor angiogenesis and metastasis. Kuang et al. conducted a study and revealed that FGB and FGG obtained from plasma exosomes hold potential as biomarkers to differentiate between benign and malignant pulmonary nodules. These biomarkers exhibited higher levels in the malignant group [58]. Furthermore, the levels of FGB and FGG in exosomes have shown promise as early diagnostic markers for colorectal cancer and liver cancer [59, 60]. Moreover, high expression of FGB and FGG in tumor tissue has been associated with a poorer prognosis in patients with gastric, prostate, liver, and colorectal cancers [61–64]. VWF, a complex plasma glycoprotein that facilitates platelet attachment to the endothelium [65], plays a pivotal role in hemostasis, tumor progression, and metastasis. Multiple studies have demonstrated that elevated plasma levels of VWF are linked to a poorer prognosis in patients with liver cancer, breast cancer, and NSCLC [66, 67]. Additionally, the level of VWF can serve as a biomarker for the early diagnosis of liver cancer and lung adenocarcinoma [68, 69]. Our study showed that increased levels of FGB, FGG, and VWF proteins in plasma exosomes from NSCLC patients suggest their potential as noninvasive biomarkers for NSCLC. These findings contribute to the development of more effective diagnostic tools for lung cancer, ultimately improving patient care and outcomes.

Our study, like any research endeavor, has limitations and strengths that necessitate a comprehensive and unbiased interpretation. Drawing from our research experience and insights from previous publications [70, 71], we believe that the chosen sample size was appropriate, and we have successfully identified promising biomarkers capable of predicting NSCLC metastasis. However, to establish the clinical utility and robustness of these biomarkers, it is imperative to conduct further validation using a larger cohort of patients and healthy individuals. Additionally, delving into the molecular mechanisms underlying these biomarkers and exploring their potential as therapeutic targets in the context of NSCLC metastasis is of utmost importance. Furthermore, while our study primarily centered on exosomes for tumor and metastasis prediction, we acknowledge the discrepancies in protein abundance between exosomes and non-exosome tissues. Despite our initial focus on exosomes for tumor and metastasis prediction, we are cognizant of the challenges tied to exosome analysis, both in terms of complexity and cost. Our forthcoming research will thus assess the predictive efficacy of these proteins not only in exosomes but also in serum and tumor tissues, enabling a more holistic clinical utility evaluation.

Nonetheless, our study reported several proteins identified from exosomes that hold the potential to serve as indicators in the diagnosis and prognosis of NSCLC. By unraveling the intricate workings of these biomarkers, we can attain a deeper comprehension of their roles in metastasis, thereby opening new avenues for targeted therapeutic interventions. Our work may ultimately contribute to evidence-based clinical decision-making, enabling individualized prognosis and the exploration of diverse combination therapy approaches. We hold the aspiration that this research will pave the way for improved patient outcomes and advancements in the management of NSCLC metastasis.

## Conclusions

In summary, our study has identified two distinct panels of plasma exosome proteins that hold significant implications for lung cancer management. The first panel, consisting of FGB, FGG, and VWF proteins, exhibits promise as a diagnostic tool for the early detection of lung cancer. The second panel, comprising CFHR5, C9, and MBL2 proteins, shows potential in evaluating the occurrence of metastasis in patients with early-stage lung cancer. While further research is needed to validate the accuracy and reliability of these candidate proteins, our experimental data establish a strong correlation between exosome proteins and lung cancer metastasis. These findings underscore the potential of utilizing plasma exosome proteins as predictive biomarkers for metastasis, thus enhancing their application in cancer screening, monitoring, and clinical management. By integrating these biomarkers into routine clinical practice, healthcare professionals can improve their ability to identify metastatic events and make well-informed treatment decisions, ultimately leading to improved patient outcomes.

### Supplementary Information

**Additional file 1. Figure S1.** The components of exosome proteins.

**Additional file 2. Figure S2.** The networks of the proteins.

**Additional file 3: Table S1.** MS/MS spetrum database search analysis summary.

**Additional file 4. Table S2.** All detected proteins that passed through TMT.

**Additional file 5.** Detailed analysis steps of bioinformatics.

## Data Availability

All data generated or analyzed during this study are included in this published article and its supplementary information files.

## Abbreviations

AUC: Area under curve
C9: Complement component 9
CFHR5: Complement factor H related protein 5
CT: Computed tomography
CTCs: Circulating tumor cells
EV: Extracellular vehicles
FGB: Fibrinogen beta chain
FGG: Fibrinogen gamma chain
LC–MS/MS: Liquid chromatography tandem mass spectrometry
MBL2: Mannose-binding lectin 2
NSCLC: Non-small cell lung cancer
PET-CT: Positron emission tomography-computed tomography
PRM: Parallel reaction monitoring
ROC: Receiver operating characteristic
TMT: Tandem mass tag
VWF: Von Willebrand factor
