# Supplementary figures and images for: Identification of exosome protein panels as predictive biomarkers for non-small cell lung cancer
Source: Biol Proced Online. 2023 Nov 13;25:29. doi: 10.1186/s12575-023-00223-0 (PMC10641949; doi:10.1186/s12575-023-00223-0)

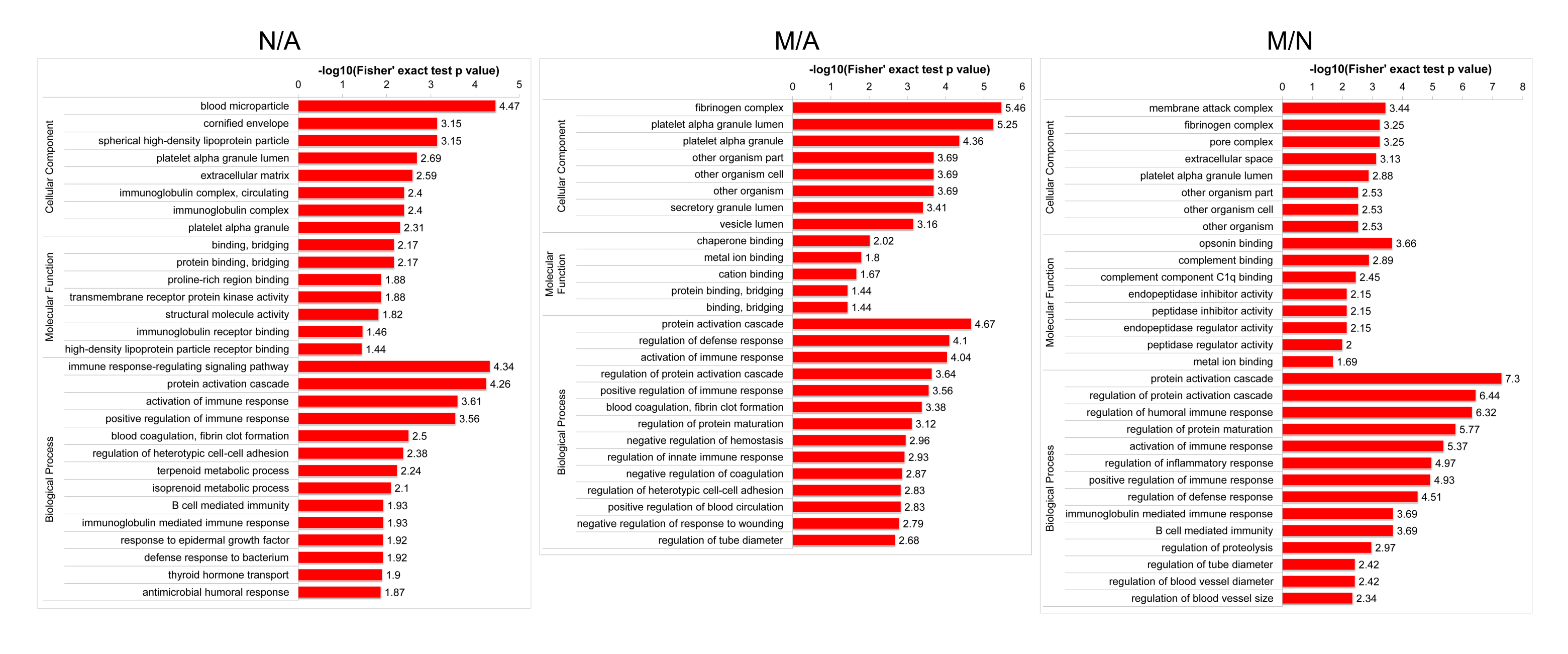

Supplement: Supplementary file 1 — Additional file 1. Figure S1. The components of exosome proteins. [file 12575_2023_223_MOESM1_ESM.tif]

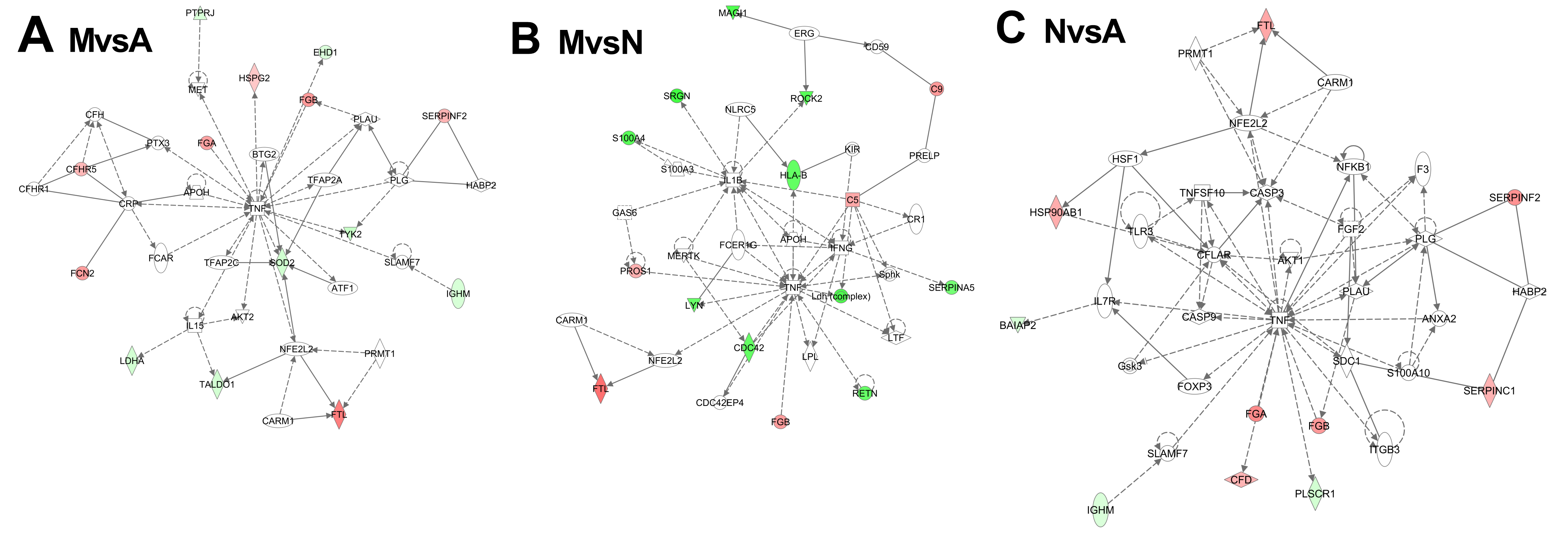

Supplement: Supplementary file 2 — Additional file 2. Figure S2. The networks of the proteins. [file 12575_2023_223_MOESM2_ESM.tif]
