# Supplementary material for: Identification of exosome protein panels as predictive biomarkers for non-small cell lung cancer
Source: Biol Proced Online. 2023 Nov 13;25:29. doi: 10.1186/s12575-023-00223-0 (PMC10641949; doi:10.1186/s12575-023-00223-0)
Supplement: Supplementary file 3 — Additional file 3: Table S1. MS/MS spetrum database search analysis summary. [file 12575_2023_223_MOESM3_ESM.doc]

| Table 1. MS/MS spetrum database search analysis summary | | | | | |
| --- | --- | --- | --- | --- | --- |
| **Total spectrum** | **Matched spectrum** | **Peptides** | **Unique peptides** | **Identified proteins** | **Quantifiable proteins** |
| 1313193 | 52889 (4.0%) | 12633 | 11915 | 1220 | 1097 |

| ***Protein information*** | | | | ***M/A*** | | ***M/N*** | | ***N/A*** | |
| --- | --- | --- | --- | --- | --- | --- | --- | --- | --- |
| ***Protein accession*** | ***Protein description*** | ***Gene name*** | ***MW [kDa]*** | ***Ratio*** | ***P value*** | ***Ratio*** | ***P value*** | ***Ratio*** | ***P value*** |
| P37802 | Transgelin-2 OS=Homo sapiens OX=9606 GN=TAGLN2 PE=1 SV=3 | TAGLN2 | 22.391 | 0.89 | 0.1709 | 0.79 | 0.0621 | 1.12 | 0.2764 |
| P11226 | Mannose-binding protein C OS=Homo sapiens OX=9606 GN=MBL2 PE=1 SV=2 | MBL2 | 26.143 | 1.84 | 0.0879 | 1.55 | 0.0935 | 1.19 | 0.4636 |
| P02656 | Apolipoprotein C-III OS=Homo sapiens OX=9606 GN=APOC3 PE=1 SV=1 | APOC3 | 10.852 | 1.31 | 0.1143 | 0.92 | 0.5737 | 1.43 | 0.0240 |
| Q16706 | Alpha-mannosidase 2 OS=Homo sapiens OX=9606 GN=MAN2A1 PE=1 SV=2 | MAN2A1 | 131.14 | 0.98 | 0.8576 | 0.95 | 0.6777 | 1.04 | 0.8675 |
| P30101 | Protein disulfide-isomerase A3 OS=Homo sapiens OX=9606 GN=PDIA3 PE=1 SV=4 | PDIA3 | 56.782 | 0.81 | 0.0599 | 0.93 | 0.5349 | 0.88 | 0.0415 |
| Q13009 | T-lymphoma invasion and metastasis-inducing protein 1 OS=Homo sapiens OX=9606 GN=TIAM1 PE=1 SV=2 | TIAM1 | 177.51 | 0.86 | 0.5315 | 0.72 | 0.6220 | 1.19 | 0.9279 |
| P01594 | Immunoglobulin kappa variable 1-33 OS=Homo sapiens OX=9606 GN=IGKV1-33 PE=1 SV=2 | IGKV1-33 | 12.848 | 0.71 | 0.0662 | 0.93 | 0.4624 | 0.76 | 0.0467 |
| P28072 | Proteasome subunit beta type-6 OS=Homo sapiens OX=9606 GN=PSMB6 PE=1 SV=4 | PSMB6 | 25.357 | 1.13 | 0.4712 | 1.13 | 0.3423 | 1.00 | 0.9164 |
| P00736 | Complement C1r subcomponent OS=Homo sapiens OX=9606 GN=C1R PE=1 SV=2 | C1R | 80.118 | 1.00 | 0.8773 | 0.95 | 0.4349 | 1.05 | 0.5317 |
| P02724 | Glycophorin-A OS=Homo sapiens OX=9606 GN=GYPA PE=1 SV=2 | GYPA | 16.331 | 0.83 | 0.2903 | 0.88 | 0.3092 | 0.95 | 0.6864 |
| P05155 | Plasma protease C1 inhibitor OS=Homo sapiens OX=9606 GN=SERPING1 PE=1 SV=2 | SERPING1 | 55.154 | 1.04 | 0.7550 | 1.07 | 0.6226 | 0.97 | 0.9760 |
| Q9UQB8 | Brain-specific angiogenesis inhibitor 1-associated protein 2 OS=Homo sapiens OX=9606 GN=BAIAP2 PE=1 SV=1 | BAIAP2 | 60.867 | 0.78 | 0.5201 | 1.16 | 0.9096 | 0.67 | 0.0214 |
| P0DOX5 | Immunoglobulin gamma-1 heavy chain OS=Homo sapiens OX=9606 PE=1 SV=2 | --- | 49.328 | 0.90 | 0.3856 | 1.20 | 0.6732 | 0.75 | 0.0202 |
| A0A0A0MRZ8 | Immunoglobulin kappa variable 3D-11 OS=Homo sapiens OX=9606 GN=IGKV3D-11 PE=3 SV=6 | IGKV3D-11 | 12.625 | 1.01 | 0.9162 | 0.98 | 0.9528 | 1.03 | 0.8687 |
| Q9P2P6 | StAR-related lipid transfer protein 9 OS=Homo sapiens OX=9606 GN=STARD9 PE=1 SV=3 | STARD9 | 516.34 | 0.74 | 0.2933 | 1.04 | 0.9710 | 0.72 | 0.0453 |
| P80723 | Brain acid soluble protein 1 OS=Homo sapiens OX=9606 GN=BASP1 PE=1 SV=2 | BASP1 | 22.693 | 1.22 | 0.3660 | 0.85 | 0.5338 | 1.43 | 0.1432 |
| P01019 | Angiotensinogen OS=Homo sapiens OX=9606 GN=AGT PE=1 SV=1 | AGT | 53.154 | 0.54 | 0.0090 | 0.85 | 0.3712 | 0.64 | 0.0115 |
| O76081 | Regulator of G-protein signaling 20 OS=Homo sapiens OX=9606 GN=RGS20 PE=1 SV=4 | RGS20 | 43.691 | 1.26 | 0.2759 | 1.15 | 0.4387 | 1.10 | 0.8059 |
| P18054 | Arachidonate 12-lipoxygenase, 12S-type OS=Homo sapiens OX=9606 GN=ALOX12 PE=1 SV=4 | ALOX12 | 75.693 | 4.73 | 0.4189 | 2.65 | 0.6509 | 1.79 | 0.1478 |
| P21980 | Protein-glutamine gamma-glutamyltransferase 2 OS=Homo sapiens OX=9606 GN=TGM2 PE=1 SV=2 | TGM2 | 77.328 | 0.97 | 0.9189 | 0.85 | 0.2782 | 1.14 | 0.3562 |
| Q07954 | Prolow-density lipoprotein receptor-related protein 1 OS=Homo sapiens OX=9606 GN=LRP1 PE=1 SV=2 | LRP1 | 504.6 | 1.04 | 0.6645 | 0.93 | 0.4741 | 1.12 | 0.2733 |
| P20929 | Nebulin OS=Homo sapiens OX=9606 GN=NEB PE=1 SV=5 | NEB | 772.91 | 2.01 | 0.0940 | 1.95 | 0.1511 | 1.03 | 0.7811 |
| Q9BUK0 | Coiled-coil-helix-coiled-coil-helix domain-containing protein 7 OS=Homo sapiens OX=9606 GN=CHCHD7 PE=1 SV=1 | CHCHD7 | 10.095 |  |  |  |  |  |  |
| P50990 | T-complex protein 1 subunit theta OS=Homo sapiens OX=9606 GN=CCT8 PE=1 SV=4 | CCT8 | 59.62 | 0.90 | 0.2914 | 0.89 | 0.3758 | 1.01 | 0.9449 |
| P00748 | Coagulation factor XII OS=Homo sapiens OX=9606 GN=F12 PE=1 SV=3 | F12 | 67.791 | 0.96 | 0.8028 | 0.79 | 0.6406 | 1.21 | 0.7963 |
| P14868 | Aspartate--tRNA ligase, cytoplasmic OS=Homo sapiens OX=9606 GN=DARS PE=1 SV=2 | DARS | 57.136 | 1.41 | 0.4940 | 0.50 | 0.4554 | 2.83 | 0.3054 |
| P20585 | DNA mismatch repair protein Msh3 OS=Homo sapiens OX=9606 GN=MSH3 PE=1 SV=4 | MSH3 | 127.41 | 5.18 | 0.3626 | 0.94 | 0.9986 | 5.53 | 0.2302 |
| Q9Y6R7 | IgGFc-binding protein OS=Homo sapiens OX=9606 GN=FCGBP PE=1 SV=3 | FCGBP | 572.01 | 1.09 | 0.5492 | 1.08 | 0.4481 | 1.01 | 0.9250 |
| P63218 | Guanine nucleotide-binding protein G(I)/G(S)/G(O) subunit gamma-5 OS=Homo sapiens OX=9606 GN=GNG5 PE=1 SV=3 | GNG5 | 7.3184 | 0.96 | 0.7192 | 0.96 | 0.8837 | 1.00 | 0.8609 |
| P47929 | Galectin-7 OS=Homo sapiens OX=9606 GN=LGALS7 PE=1 SV=2 | LGALS7 | 15.075 | 1.22 | 0.5251 | 1.05 | 0.6976 | 1.16 | 0.6227 |
| P03952 | Plasma kallikrein OS=Homo sapiens OX=9606 GN=KLKB1 PE=1 SV=1 | KLKB1 | 71.369 | 0.88 | 0.2624 | 0.93 | 0.4732 | 0.94 | 0.4604 |
| P01008 | Antithrombin-III OS=Homo sapiens OX=9606 GN=SERPINC1 PE=1 SV=1 | SERPINC1 | 52.602 | 1.24 | 0.2032 | 0.96 | 0.6333 | 1.30 | 0.0289 |
| Q6ZN06 | Zinc finger protein 813 OS=Homo sapiens OX=9606 GN=ZNF813 PE=2 SV=2 | ZNF813 | 71.72 |  |  |  |  |  |  |
| O75342 | Arachidonate 12-lipoxygenase, 12R-type OS=Homo sapiens OX=9606 GN=ALOX12B PE=1 SV=1 | ALOX12B | 80.355 |  |  |  |  |  |  |
| P02751 | Fibronectin OS=Homo sapiens OX=9606 GN=FN1 PE=1 SV=4 | FN1 | 262.62 | 1.23 | 0.3510 | 0.88 | 0.3941 | 1.41 | 0.0518 |
| P08123 | Collagen alpha-2(I) chain OS=Homo sapiens OX=9606 GN=COL1A2 PE=1 SV=7 | COL1A2 | 129.31 |  |  |  |  |  |  |
| P04259 | Keratin, type II cytoskeletal 6B OS=Homo sapiens OX=9606 GN=KRT6B PE=1 SV=5 | KRT6B | 60.066 | 1.34 | 0.4891 | 1.23 | 0.4545 | 1.09 | 0.7067 |
| Q9NZM3 | Intersectin-2 OS=Homo sapiens OX=9606 GN=ITSN2 PE=1 SV=3 | ITSN2 | 193.46 | 0.97 | 0.7523 | 0.76 | 0.0082 | 1.28 | 0.0090 |
| Q9BTC8 | Metastasis-associated protein MTA3 OS=Homo sapiens OX=9606 GN=MTA3 PE=1 SV=2 | MTA3 | 67.503 | 0.69 | 0.1727 | 0.48 | 0.0574 | 1.43 | 0.6341 |
| P12273 | Prolactin-inducible protein OS=Homo sapiens OX=9606 GN=PIP PE=1 SV=1 | PIP | 16.572 | 1.23 | 0.5320 | 0.78 | 0.5815 | 1.58 | 0.2593 |
| Q96F81 | Protein dispatched homolog 1 OS=Homo sapiens OX=9606 GN=DISP1 PE=1 SV=3 | DISP1 | 170.93 | 1.60 | 0.7232 | 0.45 | 0.4734 | 3.53 | 0.3428 |
| Q16181 | Septin-7 OS=Homo sapiens OX=9606 GN=SEPT7 PE=1 SV=2 | SEPT7 | 50.679 | 0.81 | 0.4991 | 0.74 | 0.4570 | 1.09 | 0.9660 |
| P78371 | T-complex protein 1 subunit beta OS=Homo sapiens OX=9606 GN=CCT2 PE=1 SV=4 | CCT2 | 57.488 | 1.25 | 0.2158 | 0.93 | 0.6194 | 1.34 | 0.1159 |
| Q8WWI5 | Choline transporter-like protein 1 OS=Homo sapiens OX=9606 GN=SLC44A1 PE=1 SV=1 | SLC44A1 | 73.301 | 1.24 | 0.1190 | 1.08 | 0.6375 | 1.15 | 0.6246 |
| Q15365 | Poly(rC)-binding protein 1 OS=Homo sapiens OX=9606 GN=PCBP1 PE=1 SV=2 | PCBP1 | 37.497 | 0.95 | 0.7546 | 0.85 | 0.7550 | 1.12 | 0.9271 |
| P16157 | Ankyrin-1 OS=Homo sapiens OX=9606 GN=ANK1 PE=1 SV=3 | ANK1 | 206.26 | 0.80 | 0.4562 | 0.90 | 0.4505 | 0.89 | 0.7004 |
| P40926 | Malate dehydrogenase, mitochondrial OS=Homo sapiens OX=9606 GN=MDH2 PE=1 SV=3 | MDH2 | 35.503 | 1.42 | 0.6444 | 1.65 | 0.1679 | 0.86 | 0.3947 |
| P01876 | Immunoglobulin heavy constant alpha 1 OS=Homo sapiens OX=9606 GN=IGHA1 PE=1 SV=2 | IGHA1 | 37.654 | 0.65 | 0.0182 | 1.02 | 0.9901 | 0.64 | 0.0005 |
| A0A075B6S2 | Immunoglobulin kappa variable 2D-29 OS=Homo sapiens OX=9606 GN=IGKV2D-29 PE=3 SV=1 | IGKV2D-29 | 13.143 | 0.82 | 0.3532 | 0.91 | 0.5780 | 0.90 | 0.4146 |
| P02792 | Ferritin light chain OS=Homo sapiens OX=9606 GN=FTL PE=1 SV=2 | FTL | 20.019 | 3.40 | 0.0012 | 2.27 | 0.0002 | 1.50 | 0.0036 |
| P78504 | Protein jagged-1 OS=Homo sapiens OX=9606 GN=JAG1 PE=1 SV=3 | JAG1 | 133.8 | 0.90 |  | 1.09 | 0.6519 | 0.82 |  |
| O75916 | Regulator of G-protein signaling 9 OS=Homo sapiens OX=9606 GN=RGS9 PE=1 SV=1 | RGS9 | 76.965 |  |  |  |  |  |  |
| P01023 | Alpha-2-macroglobulin OS=Homo sapiens OX=9606 GN=A2M PE=1 SV=3 | A2M | 163.29 | 1.11 | 0.6604 | 1.12 | 0.5134 | 0.99 | 0.9926 |
| P35241 | Radixin OS=Homo sapiens OX=9606 GN=RDX PE=1 SV=1 | RDX | 68.563 | 1.50 | 0.0474 | 1.24 | 0.1659 | 1.21 | 0.2855 |
| A0A0B4J1U3 | Immunoglobulin lambda variable 1-36 OS=Homo sapiens OX=9606 GN=IGLV1-36 PE=3 SV=5 | IGLV1-36 | 12.478 | 1.07 | 0.8822 | 1.10 | 0.7101 | 0.98 | 0.8567 |
| P09104 | Gamma-enolase OS=Homo sapiens OX=9606 GN=ENO2 PE=1 SV=3 | ENO2 | 47.268 | 1.12 | 0.4092 | 1.85 | 0.1736 | 0.60 | 0.0588 |
| A8MUU1 | Putative fatty acid-binding protein 5-like protein 3 OS=Homo sapiens OX=9606 GN=FABP5P3 PE=5 SV=1 | FABP5P3 | 11.299 | 0.94 | 0.6862 | 0.70 | 0.2670 | 1.34 | 0.4711 |
| A0A0C4DH25 | Immunoglobulin kappa variable 3D-20 OS=Homo sapiens OX=9606 GN=IGKV3D-20 PE=3 SV=1 | IGKV3D-20 | 12.515 | 0.44 | 0.1819 | 0.81 | 0.4854 | 0.55 | 0.1643 |
| P60033 | CD81 antigen OS=Homo sapiens OX=9606 GN=CD81 PE=1 SV=1 | CD81 | 25.809 | 1.40 | 0.1111 | 1.13 | 0.4382 | 1.23 | 0.1026 |
| P00441 | Superoxide dismutase [Cu-Zn] OS=Homo sapiens OX=9606 GN=SOD1 PE=1 SV=2 | SOD1 | 15.936 | 0.83 | 0.1422 | 0.82 | 0.1336 | 1.01 | 0.8824 |
| P51587 | Breast cancer type 2 susceptibility protein OS=Homo sapiens OX=9606 GN=BRCA2 PE=1 SV=3 | BRCA2 | 384.2 | 0.94 | 0.9428 | 1.10 | 0.6217 | 0.86 | 0.6055 |
| P51148 | Ras-related protein Rab-5C OS=Homo sapiens OX=9606 GN=RAB5C PE=1 SV=2 | RAB5C | 23.482 | 1.02 | 0.9191 | 0.94 | 0.7084 | 1.09 | 0.6482 |
| Q9C0H2 | Protein tweety homolog 3 OS=Homo sapiens OX=9606 GN=TTYH3 PE=1 SV=3 | TTYH3 | 57.544 | 1.01 | 0.8496 | 1.00 | 0.7911 | 1.01 | 0.9981 |
| P49746 | Thrombospondin-3 OS=Homo sapiens OX=9606 GN=THBS3 PE=1 SV=1 | THBS3 | 104.2 | 1.01 | 0.9559 | 1.08 | 0.9578 | 0.94 | 0.8796 |
| P30273 | High affinity immunoglobulin epsilon receptor subunit gamma OS=Homo sapiens OX=9606 GN=FCER1G PE=1 SV=1 | FCER1G | 9.6674 | 0.71 | 0.0622 | 0.83 | 0.3636 | 0.86 | 0.2869 |
| A0A075B6I1 | Immunoglobulin lambda variable 4-60 OS=Homo sapiens OX=9606 GN=IGLV4-60 PE=3 SV=1 | IGLV4-60 | 12.987 | 0.93 | 0.6934 | 1.09 | 0.6018 | 0.86 | 0.8769 |
| P01714 | Immunoglobulin lambda variable 3-19 OS=Homo sapiens OX=9606 GN=IGLV3-19 PE=1 SV=2 | IGLV3-19 | 12.042 | 0.87 | 0.3645 | 0.88 | 0.4273 | 0.99 | 0.8549 |
| P62258 | 14-3-3 protein epsilon OS=Homo sapiens OX=9606 GN=YWHAE PE=1 SV=1 | YWHAE | 29.174 | 0.70 | 0.0759 | 0.86 | 0.3790 | 0.82 | 0.1907 |
| P28749 | Retinoblastoma-like protein 1 OS=Homo sapiens OX=9606 GN=RBL1 PE=1 SV=3 | RBL1 | 120.85 | 5.51 | 0.1224 | 4.35 | 0.2598 | 1.27 | 0.0933 |
| Q9UBW5 | Bridging integrator 2 OS=Homo sapiens OX=9606 GN=BIN2 PE=1 SV=3 | BIN2 | 61.874 | 0.96 | 0.6151 | 0.75 | 0.1709 | 1.28 | 0.1253 |
| Q8NEF9 | Serum response factor-binding protein 1 OS=Homo sapiens OX=9606 GN=SRFBP1 PE=1 SV=1 | SRFBP1 | 48.633 | 0.80 | 0.4813 | 1.00 | 0.9265 | 0.80 | 0.1788 |
| Q99832 | T-complex protein 1 subunit eta OS=Homo sapiens OX=9606 GN=CCT7 PE=1 SV=2 | CCT7 | 59.366 | 0.79 | 0.0648 | 0.83 | 0.0555 | 0.94 | 0.6317 |
| A0A0A0MT89 | Immunoglobulin kappa joining 1 OS=Homo sapiens OX=9606 GN=IGKJ1 PE=4 SV=2 | IGKJ1 | 1.3936 | 0.41 | 0.3967 | 0.58 | 0.1561 | 0.72 | 0.6460 |
| P02452 | Collagen alpha-1(I) chain OS=Homo sapiens OX=9606 GN=COL1A1 PE=1 SV=5 | COL1A1 | 138.94 | 1.78 | 0.1301 | 1.05 | 0.9665 | 1.70 | 0.0261 |
| O75390 | Citrate synthase, mitochondrial OS=Homo sapiens OX=9606 GN=CS PE=1 SV=2 | CS | 51.712 | 0.71 | 0.3817 | 0.60 | 0.1825 | 1.18 | 0.8002 |
| Q8WUJ3 | Cell migration-inducing and hyaluronan-binding protein OS=Homo sapiens OX=9606 GN=CEMIP PE=1 SV=2 | CEMIP | 153 | 1.02 | 0.8772 | 1.01 | 0.9823 | 1.01 | 0.8069 |
| Q09428 | ATP-binding cassette sub-family C member 8 OS=Homo sapiens OX=9606 GN=ABCC8 PE=1 SV=6 | ABCC8 | 176.99 | 0.80 | 0.2745 | 0.65 | 0.2674 | 1.23 | 0.6571 |
| Q86UD1 | Out at first protein homolog OS=Homo sapiens OX=9606 GN=OAF PE=2 SV=1 | OAF | 30.688 |  |  |  |  |  |  |
| A0A0C4DH31 | Immunoglobulin heavy variable 1-18 OS=Homo sapiens OX=9606 GN=IGHV1-18 PE=3 SV=1 | IGHV1-18 | 12.82 | 1.05 | 0.4408 | 0.98 | 0.8987 | 1.07 | 0.4039 |
| Q8N392 | Rho GTPase-activating protein 18 OS=Homo sapiens OX=9606 GN=ARHGAP18 PE=1 SV=3 | ARHGAP18 | 74.976 | 0.78 | 0.0375 | 0.71 | 0.1376 | 1.11 | 0.6750 |
| P13501 | C-C motif chemokine 5 OS=Homo sapiens OX=9606 GN=CCL5 PE=1 SV=3 | CCL5 | 9.9896 | 1.06 | 0.7864 | 0.78 | 0.8998 | 1.37 | 0.6026 |
| Q8IV76 | Circadian clock protein PASD1 OS=Homo sapiens OX=9606 GN=PASD1 PE=1 SV=1 | PASD1 | 87.427 | 0.65 | 0.1519 | 0.92 | 0.8702 | 0.71 | 0.2225 |
| P14780 | Matrix metalloproteinase-9 OS=Homo sapiens OX=9606 GN=MMP9 PE=1 SV=3 | MMP9 | 78.457 | 1.00 | 0.8937 | 0.98 | 0.8349 | 1.02 | 0.9806 |
| Q86U17 | Serpin A11 OS=Homo sapiens OX=9606 GN=SERPINA11 PE=2 SV=2 | SERPINA11 | 46.989 | 1.45 | 0.2714 | 0.76 | 0.5926 | 1.92 | 0.1673 |
| P11279 | Lysosome-associated membrane glycoprotein 1 OS=Homo sapiens OX=9606 GN=LAMP1 PE=1 SV=3 | LAMP1 | 44.882 | 1.08 | 0.6301 | 0.92 | 0.8908 | 1.17 | 0.7297 |
| P06734 | Low affinity immunoglobulin epsilon Fc receptor OS=Homo sapiens OX=9606 GN=FCER2 PE=1 SV=1 | FCER2 | 36.468 | 2.26 | 0.0791 | 0.81 | 0.9115 | 2.79 | 0.1725 |
| P61201 | COP9 signalosome complex subunit 2 OS=Homo sapiens OX=9606 GN=COPS2 PE=1 SV=1 | COPS2 | 51.596 | 2.87 | 0.5679 | 1.70 | 0.5248 | 1.69 | 0.8378 |
| O94915 | Protein furry homolog-like OS=Homo sapiens OX=9606 GN=FRYL PE=1 SV=2 | FRYL | 339.59 |  |  |  |  |  |  |
| P16615 | Sarcoplasmic/endoplasmic reticulum calcium ATPase 2 OS=Homo sapiens OX=9606 GN=ATP2A2 PE=1 SV=1 | ATP2A2 | 114.76 | 0.94 | 0.7625 | 0.90 | 0.9422 | 1.05 | 0.8545 |
| Q01518 | Adenylyl cyclase-associated protein 1 OS=Homo sapiens OX=9606 GN=CAP1 PE=1 SV=5 | CAP1 | 51.901 | 0.68 | 0.0513 | 0.80 | 0.1859 | 0.85 | 0.3585 |
| P03973 | Antileukoproteinase OS=Homo sapiens OX=9606 GN=SLPI PE=1 SV=2 | SLPI | 14.326 | 1.40 | 0.3252 | 0.79 | 0.3996 | 1.77 | 0.0108 |
| P30685 | HLA class I histocompatibility antigen, B-35 alpha chain OS=Homo sapiens OX=9606 GN=HLA-B PE=1 SV=1 | HLA-B | 40.455 | 0.64 | 0.1451 | 0.73 | 0.0415 | 0.88 | 0.6060 |
| Q8NBI5 | Solute carrier family 43 member 3 OS=Homo sapiens OX=9606 GN=SLC43A3 PE=1 SV=2 | SLC43A3 | 54.528 | 0.95 | 0.5872 | 0.84 | 0.7072 | 1.14 | 0.8848 |
| Q8TDL5 | BPI fold-containing family B member 1 OS=Homo sapiens OX=9606 GN=BPIFB1 PE=1 SV=1 | BPIFB1 | 52.441 |  |  |  |  |  |  |
| P02748 | Complement component C9 OS=Homo sapiens OX=9606 GN=C9 PE=1 SV=2 | C9 | 63.173 | 1.95 | 0.0033 | 1.62 | 0.0049 | 1.20 | 0.4971 |
| Q06830 | Peroxiredoxin-1 OS=Homo sapiens OX=9606 GN=PRDX1 PE=1 SV=1 | PRDX1 | 22.11 | 0.90 | 0.3766 | 0.89 | 0.4115 | 1.00 | 0.8741 |
| P51149 | Ras-related protein Rab-7a OS=Homo sapiens OX=9606 GN=RAB7A PE=1 SV=1 | RAB7A | 23.489 | 0.88 | 0.2600 | 0.81 | 0.2065 | 1.09 | 0.7619 |
| Q5UIP0 | Telomere-associated protein RIF1 OS=Homo sapiens OX=9606 GN=RIF1 PE=1 SV=2 | RIF1 | 274.46 |  |  |  |  |  |  |
| P84095 | Rho-related GTP-binding protein RhoG OS=Homo sapiens OX=9606 GN=RHOG PE=1 SV=1 | RHOG | 21.308 | 0.94 | 0.3926 | 0.96 | 0.9443 | 0.99 | 0.8226 |
| Q6UWP8 | Suprabasin OS=Homo sapiens OX=9606 GN=SBSN PE=1 SV=2 | SBSN | 60.54 | 1.30 | 0.2330 | 0.96 | 0.5523 | 1.36 | 0.0126 |
| Q9Y6Y1 | Calmodulin-binding transcription activator 1 OS=Homo sapiens OX=9606 GN=CAMTA1 PE=1 SV=4 | CAMTA1 | 183.67 | 1.83 | 0.0725 | 1.45 | 0.0951 | 1.27 | 0.2681 |
| Q9NZP8 | Complement C1r subcomponent-like protein OS=Homo sapiens OX=9606 GN=C1RL PE=1 SV=2 | C1RL | 53.498 | 1.07 | 0.4890 | 1.09 | 0.3274 | 0.98 | 0.7655 |
| Q96QD8 | Sodium-coupled neutral amino acid transporter 2 OS=Homo sapiens OX=9606 GN=SLC38A2 PE=1 SV=2 | SLC38A2 | 56.025 | 0.39 | 0.0667 | 0.73 | 0.4105 | 0.53 | 0.1170 |
| P11413 | Glucose-6-phosphate 1-dehydrogenase OS=Homo sapiens OX=9606 GN=G6PD PE=1 SV=4 | G6PD | 59.256 |  |  |  |  |  |  |
| Q14764 | Major vault protein OS=Homo sapiens OX=9606 GN=MVP PE=1 SV=4 | MVP | 99.326 | 1.12 | 0.8243 | 0.86 | 0.2868 | 1.31 | 0.1589 |
| P02042 | Hemoglobin subunit delta OS=Homo sapiens OX=9606 GN=HBD PE=1 SV=2 | HBD | 16.055 | 0.84 | 0.3803 | 0.92 | 0.5563 | 0.92 | 0.6074 |
| Q7L576 | Cytoplasmic FMR1-interacting protein 1 OS=Homo sapiens OX=9606 GN=CYFIP1 PE=1 SV=1 | CYFIP1 | 145.18 | 0.80 | 0.2323 | 0.73 | 0.2404 | 1.09 | 0.9765 |
| P29401 | Transketolase OS=Homo sapiens OX=9606 GN=TKT PE=1 SV=3 | TKT | 67.877 | 0.87 | 0.5883 | 0.94 | 0.7171 | 0.92 | 0.6367 |
| P11215 | Integrin alpha-M OS=Homo sapiens OX=9606 GN=ITGAM PE=1 SV=2 | ITGAM | 127.18 | 0.91 | 0.5303 | 0.83 | 0.3585 | 1.09 | 0.7815 |
| P63098 | Calcineurin subunit B type 1 OS=Homo sapiens OX=9606 GN=PPP3R1 PE=1 SV=2 | PPP3R1 | 19.3 | 1.06 | 0.8061 | 1.26 | 0.3672 | 0.84 | 0.4390 |
| O75030 | Microphthalmia-associated transcription factor OS=Homo sapiens OX=9606 GN=MITF PE=1 SV=2 | MITF | 58.795 | 0.67 | 0.1163 | 0.29 | 0.3147 | 2.29 | 0.3693 |
| P02765 | Alpha-2-HS-glycoprotein OS=Homo sapiens OX=9606 GN=AHSG PE=1 SV=1 | AHSG | 39.324 | 0.96 | 0.8709 | 0.88 | 0.4743 | 1.09 | 0.6248 |
| Q8N699 | Myc target protein 1 OS=Homo sapiens OX=9606 GN=MYCT1 PE=1 SV=1 | MYCT1 | 26.592 | 0.89 | 0.4579 | 0.93 | 0.4268 | 0.96 | 0.7203 |
| Q93088 | Betaine--homocysteine S-methyltransferase 1 OS=Homo sapiens OX=9606 GN=BHMT PE=1 SV=2 | BHMT | 44.998 | 1.24 | 0.6376 | 1.59 | 0.0927 | 0.78 | 0.3040 |
| P61626 | Lysozyme C OS=Homo sapiens OX=9606 GN=LYZ PE=1 SV=1 | LYZ | 16.537 | 0.96 | 0.8234 | 0.73 | 0.3283 | 1.31 | 0.4752 |
| O14672 | Disintegrin and metalloproteinase domain-containing protein 10 OS=Homo sapiens OX=9606 GN=ADAM10 PE=1 SV=1 | ADAM10 | 84.141 | 0.84 | 0.2480 | 0.82 | 0.4473 | 1.02 | 0.8037 |
| P35443 | Thrombospondin-4 OS=Homo sapiens OX=9606 GN=THBS4 PE=1 SV=2 | THBS4 | 105.87 | 1.01 | 0.9663 | 0.90 | 0.3149 | 1.12 | 0.3183 |
| Q14254 | Flotillin-2 OS=Homo sapiens OX=9606 GN=FLOT2 PE=1 SV=2 | FLOT2 | 47.064 | 0.89 | 0.4177 | 0.96 | 0.7089 | 0.92 | 0.5235 |
| Q08431 | Lactadherin OS=Homo sapiens OX=9606 GN=MFGE8 PE=1 SV=2 | MFGE8 | 43.122 | 1.26 | 0.1114 | 0.95 | 0.8303 | 1.32 | 0.1104 |
| O00151 | PDZ and LIM domain protein 1 OS=Homo sapiens OX=9606 GN=PDLIM1 PE=1 SV=4 | PDLIM1 | 36.071 | 0.73 | 0.0820 | 0.81 | 0.2719 | 0.91 | 0.4150 |
| Q92522 | Histone H1x OS=Homo sapiens OX=9606 GN=H1FX PE=1 SV=1 | H1FX | 22.487 | 0.69 | 0.0106 | 0.45 | 0.5096 | 1.56 | 0.8474 |
| P30040 | Endoplasmic reticulum resident protein 29 OS=Homo sapiens OX=9606 GN=ERP29 PE=1 SV=4 | ERP29 | 28.993 | 0.79 | 0.4825 | 0.99 | 0.9985 | 0.80 | 0.3815 |
| Q99547 | M-phase phosphoprotein 6 OS=Homo sapiens OX=9606 GN=MPHOSPH6 PE=1 SV=2 | MPHOSPH6 | 19.024 | 5.25 | 0.1875 | 1.98 | 0.3164 | 2.65 | 0.3141 |
| P49407 | Beta-arrestin-1 OS=Homo sapiens OX=9606 GN=ARRB1 PE=1 SV=2 | ARRB1 | 47.065 | 0.82 | 0.5525 | 1.10 | 0.8195 | 0.75 | 0.3694 |
| P98160 | Basement membrane-specific heparan sulfate proteoglycan core protein OS=Homo sapiens OX=9606 GN=HSPG2 PE=1 SV=4 | HSPG2 | 468.83 | 1.43 | 0.0110 | 1.00 | 0.9401 | 1.43 | 0.0007 |
| Q12756 | Kinesin-like protein KIF1A OS=Homo sapiens OX=9606 GN=KIF1A PE=1 SV=2 | KIF1A | 191.06 | 1.46 | 0.2784 | 1.01 | 0.7845 | 1.44 | 0.3850 |
| P08185 | Corticosteroid-binding globulin OS=Homo sapiens OX=9606 GN=SERPINA6 PE=1 SV=1 | SERPINA6 | 45.14 | 1.26 | 0.2154 | 0.89 | 0.9884 | 1.41 | 0.5746 |
| Q13586 | Stromal interaction molecule 1 OS=Homo sapiens OX=9606 GN=STIM1 PE=1 SV=3 | STIM1 | 77.422 | 1.05 | 0.8683 | 1.08 | 0.6490 | 0.97 | 0.8592 |
| O15511 | Actin-related protein 2/3 complex subunit 5 OS=Homo sapiens OX=9606 GN=ARPC5 PE=1 SV=3 | ARPC5 | 16.32 | 0.84 | 0.2918 | 0.83 | 0.3560 | 1.02 | 0.7902 |
| Q9BRJ9 | Mesoderm posterior protein 1 OS=Homo sapiens OX=9606 GN=MESP1 PE=2 SV=1 | MESP1 | 28.501 | 1.05 | 0.7664 | 0.57 | 0.5112 | 1.86 | 0.5151 |
| P15169 | Carboxypeptidase N catalytic chain OS=Homo sapiens OX=9606 GN=CPN1 PE=1 SV=1 | CPN1 | 52.286 | 1.17 | 0.0401 | 1.22 | 0.0527 | 0.96 | 0.6477 |
| Q9UJJ9 | N-acetylglucosamine-1-phosphotransferase subunit gamma OS=Homo sapiens OX=9606 GN=GNPTG PE=1 SV=1 | GNPTG | 33.973 | 1.63 | 0.2349 | 1.21 | 0.3553 | 1.35 | 0.1865 |
| Q99715 | Collagen alpha-1(XII) chain OS=Homo sapiens OX=9606 GN=COL12A1 PE=1 SV=2 | COL12A1 | 333.14 | 0.37 | 0.0355 | 0.51 | 0.0157 | 0.74 | 0.1667 |
| P20851 | C4b-binding protein beta chain OS=Homo sapiens OX=9606 GN=C4BPB PE=1 SV=1 | C4BPB | 28.357 | 1.32 | 0.0138 | 1.20 | 0.0751 | 1.10 | 0.0246 |
| P23142 | Fibulin-1 OS=Homo sapiens OX=9606 GN=FBLN1 PE=1 SV=4 | FBLN1 | 77.213 | 1.19 | 0.2945 | 1.20 | 0.1472 | 0.99 | 0.9738 |
| P84077 | ADP-ribosylation factor 1 OS=Homo sapiens OX=9606 GN=ARF1 PE=1 SV=2 | ARF1 | 20.697 | 0.68 | 0.0551 | 0.65 | 0.0839 | 1.06 | 0.9298 |
| P08195 | 4F2 cell-surface antigen heavy chain OS=Homo sapiens OX=9606 GN=SLC3A2 PE=1 SV=3 | SLC3A2 | 67.993 | 1.00 | 0.6661 | 0.98 | 0.8177 | 1.02 | 0.5897 |
| Q13907 | Isopentenyl-diphosphate Delta-isomerase 1 OS=Homo sapiens OX=9606 GN=IDI1 PE=1 SV=2 | IDI1 | 26.319 | 0.76 | 0.4139 | 0.77 | 0.2406 | 0.98 | 0.8385 |
| P04217 | Alpha-1B-glycoprotein OS=Homo sapiens OX=9606 GN=A1BG PE=1 SV=4 | A1BG | 54.253 | 1.08 | 0.3457 | 0.90 | 0.0686 | 1.20 | 0.0419 |
| P49796 | Regulator of G-protein signaling 3 OS=Homo sapiens OX=9606 GN=RGS3 PE=1 SV=2 | RGS3 | 132.33 | 5.08 | 0.7002 | 6.11 | 0.6405 | 0.83 | 0.0320 |
| Q9ULE3 | DENN domain-containing protein 2A OS=Homo sapiens OX=9606 GN=DENND2A PE=2 SV=4 | DENND2A | 113.85 | 0.88 | 0.2508 | 0.87 | 0.9011 | 1.01 | 0.8446 |
| P48740 | Mannan-binding lectin serine protease 1 OS=Homo sapiens OX=9606 GN=MASP1 PE=1 SV=3 | MASP1 | 79.246 | 0.94 | 0.3231 | 1.06 | 0.3821 | 0.89 | 0.1115 |
| P63208 | S-phase kinase-associated protein 1 OS=Homo sapiens OX=9606 GN=SKP1 PE=1 SV=2 | SKP1 | 18.658 | 1.44 | 0.3777 | 0.72 | 0.2462 | 2.01 | 0.0557 |
| Q8IUC6 | TIR domain-containing adapter molecule 1 OS=Homo sapiens OX=9606 GN=TICAM1 PE=1 SV=1 | TICAM1 | 76.421 | 1.99 |  | 0.26 |  | 7.82 | 0.2970 |
| Q96PE3 | Type I inositol 3,4-bisphosphate 4-phosphatase OS=Homo sapiens OX=9606 GN=INPP4A PE=1 SV=1 | INPP4A | 109.95 | 0.53 | 0.1979 | 0.87 | 0.5436 | 0.61 | 0.3415 |
| P02549 | Spectrin alpha chain, erythrocytic 1 OS=Homo sapiens OX=9606 GN=SPTA1 PE=1 SV=5 | SPTA1 | 280.01 | 0.83 | 0.5471 | 0.91 | 0.5212 | 0.91 | 0.8005 |
| P01717 | Immunoglobulin lambda variable 3-25 OS=Homo sapiens OX=9606 GN=IGLV3-25 PE=1 SV=2 | IGLV3-25 | 12.011 | 1.12 | 0.6456 | 1.09 | 0.5941 | 1.02 | 0.9231 |
| P0DOX7 | Immunoglobulin kappa light chain OS=Homo sapiens OX=9606 PE=1 SV=1 | --- | 23.379 | 0.78 | 0.0649 | 0.89 | 0.1627 | 0.87 | 0.1837 |
| Q9UPX8 | SH3 and multiple ankyrin repeat domains protein 2 OS=Homo sapiens OX=9606 GN=SHANK2 PE=1 SV=3 | SHANK2 | 158.82 | 0.35 | 0.2081 | 0.64 | 0.0768 | 0.55 | 0.0006 |
| P04004 | Vitronectin OS=Homo sapiens OX=9606 GN=VTN PE=1 SV=1 | VTN | 54.305 | 1.25 | 0.1289 | 0.93 | 0.7125 | 1.35 | 0.0148 |
| P30457 | HLA class I histocompatibility antigen, A-66 alpha chain OS=Homo sapiens OX=9606 GN=HLA-A PE=1 SV=1 | HLA-A | 41.081 |  |  |  |  |  |  |
| Q14683 | Structural maintenance of chromosomes protein 1A OS=Homo sapiens OX=9606 GN=SMC1A PE=1 SV=2 | SMC1A | 143.23 | 0.58 | 0.0541 | 1.06 | 0.3092 | 0.55 | 0.0059 |
| Q00610 | Clathrin heavy chain 1 OS=Homo sapiens OX=9606 GN=CLTC PE=1 SV=5 | CLTC | 191.61 | 0.86 | 0.1757 | 0.89 | 0.2464 | 0.97 | 0.7080 |
| Q2KHM9 | Protein moonraker OS=Homo sapiens OX=9606 GN=KIAA0753 PE=1 SV=3 | KIAA0753 | 109.41 | 1.12 | 0.7766 | 0.56 | 0.2914 | 2.00 | 0.2990 |
| P11049 | Leukocyte antigen CD37 OS=Homo sapiens OX=9606 GN=CD37 PE=1 SV=2 | CD37 | 31.703 | 1.01 | 0.9350 | 1.02 | 0.8267 | 0.99 | 0.8932 |
| Q99700 | Ataxin-2 OS=Homo sapiens OX=9606 GN=ATXN2 PE=1 SV=2 | ATXN2 | 140.28 | 0.69 | 0.0446 | 0.68 | 0.0555 | 1.01 | 0.9563 |
| P29144 | Tripeptidyl-peptidase 2 OS=Homo sapiens OX=9606 GN=TPP2 PE=1 SV=4 | TPP2 | 138.35 | 0.85 | 0.4303 | 0.82 | 0.4145 | 1.04 | 0.9206 |
| P11217 | Glycogen phosphorylase, muscle form OS=Homo sapiens OX=9606 GN=PYGM PE=1 SV=6 | PYGM | 97.091 | 0.72 | 0.5096 | 1.10 | 0.8818 | 0.66 | 0.0958 |
| Q5M9Q1 | NKAP-like protein OS=Homo sapiens OX=9606 GN=NKAPL PE=2 SV=3 | NKAPL | 46.312 | 0.90 | 0.7675 | 0.83 | 0.8235 | 1.09 | 0.9442 |
| Q7KZF4 | Staphylococcal nuclease domain-containing protein 1 OS=Homo sapiens OX=9606 GN=SND1 PE=1 SV=1 | SND1 | 102 | 0.80 | 0.4408 | 0.91 | 0.3631 | 0.89 | 0.5329 |
| O43290 | U4/U6.U5 tri-snRNP-associated protein 1 OS=Homo sapiens OX=9606 GN=SART1 PE=1 SV=1 | SART1 | 90.254 | 1.11 | 0.4826 | 0.95 | 0.8683 | 1.17 | 0.3961 |
| P16109 | P-selectin OS=Homo sapiens OX=9606 GN=SELP PE=1 SV=3 | SELP | 90.833 | 0.88 | 0.3441 | 0.85 | 0.2834 | 1.04 | 0.9485 |
| P36537 | UDP-glucuronosyltransferase 2B10 OS=Homo sapiens OX=9606 GN=UGT2B10 PE=1 SV=1 | UGT2B10 | 60.773 | 0.79 | 0.6623 | 0.25 | 0.3266 | 3.21 | 0.4767 |
| P31151 | Protein S100-A7 OS=Homo sapiens OX=9606 GN=S100A7 PE=1 SV=4 | S100A7 | 11.471 | 0.91 | 0.9255 | 1.18 | 0.9708 | 0.77 | 0.7824 |
| P30466 | HLA class I histocompatibility antigen, B-18 alpha chain OS=Homo sapiens OX=9606 GN=HLA-B PE=1 SV=1 | HLA-B | 40.274 |  |  |  |  |  |  |
| P06312 | Immunoglobulin kappa variable 4-1 OS=Homo sapiens OX=9606 GN=IGKV4-1 PE=1 SV=1 | IGKV4-1 | 13.38 | 0.61 | 0.0018 | 0.95 | 0.7983 | 0.64 | 0.0019 |
| Q9BS26 | Endoplasmic reticulum resident protein 44 OS=Homo sapiens OX=9606 GN=ERP44 PE=1 SV=1 | ERP44 | 46.971 | 1.13 | 0.3117 | 1.04 | 0.6673 | 1.08 | 0.5043 |
| P04211 | Immunoglobulin lambda variable 7-43 OS=Homo sapiens OX=9606 GN=IGLV7-43 PE=3 SV=2 | IGLV7-43 | 12.451 | 0.45 | 0.0037 | 0.68 | 0.0345 | 0.66 | 0.0271 |
| P08514 | Integrin alpha-IIb OS=Homo sapiens OX=9606 GN=ITGA2B PE=1 SV=3 | ITGA2B | 113.38 | 0.78 | 0.0798 | 0.80 | 0.1872 | 0.97 | 0.6577 |
| Q92626 | Peroxidasin homolog OS=Homo sapiens OX=9606 GN=PXDN PE=1 SV=2 | PXDN | 165.27 | 1.27 | 0.1954 | 1.14 | 0.3908 | 1.11 | 0.4871 |
| O75116 | Rho-associated protein kinase 2 OS=Homo sapiens OX=9606 GN=ROCK2 PE=1 SV=4 | ROCK2 | 160.9 | 0.83 | 0.3402 | 0.67 | 0.0107 | 1.23 | 0.1360 |
| A0A075B6Q5 | Immunoglobulin heavy variable 3-64 OS=Homo sapiens OX=9606 GN=IGHV3-64 PE=3 SV=1 | IGHV3-64 | 12.891 | 0.77 | 0.3213 | 0.86 | 0.4876 | 0.90 | 0.6324 |
| P43034 | Platelet-activating factor acetylhydrolase IB subunit alpha OS=Homo sapiens OX=9606 GN=PAFAH1B1 PE=1 SV=2 | PAFAH1B1 | 46.637 |  |  |  |  |  |  |
| O60496 | Docking protein 2 OS=Homo sapiens OX=9606 GN=DOK2 PE=1 SV=2 | DOK2 | 45.378 | 0.88 | 0.4164 | 0.72 | 0.3014 | 1.22 | 0.7443 |
| P25025 | C-X-C chemokine receptor type 2 OS=Homo sapiens OX=9606 GN=CXCR2 PE=1 SV=2 | CXCR2 | 40.759 | 1.91 | 0.4938 | 1.28 | 0.7036 | 1.49 | 0.2033 |
| P60174 | Triosephosphate isomerase OS=Homo sapiens OX=9606 GN=TPI1 PE=1 SV=3 | TPI1 | 30.791 | 0.88 | 0.3788 | 0.86 | 0.2436 | 1.02 | 0.9630 |
| Q86WS4 | Uncharacterized protein C12orf40 OS=Homo sapiens OX=9606 GN=C12orf40 PE=1 SV=3 | C12orf40 | 74.504 |  |  |  |  |  |  |
| P11172 | Uridine 5'-monophosphate synthase OS=Homo sapiens OX=9606 GN=UMPS PE=1 SV=1 | UMPS | 52.221 | 1.73 | 0.7266 | 2.27 | 0.6007 | 0.77 | 0.5170 |
| O00273 | DNA fragmentation factor subunit alpha OS=Homo sapiens OX=9606 GN=DFFA PE=1 SV=1 | DFFA | 36.521 | 0.63 | 0.0996 | 0.86 | 0.8853 | 0.74 | 0.2760 |
| Q8TF42 | Ubiquitin-associated and SH3 domain-containing protein B OS=Homo sapiens OX=9606 GN=UBASH3B PE=1 SV=2 | UBASH3B | 72.695 | 0.49 | 0.0535 | 0.60 | 0.4011 | 0.81 | 0.4802 |
| P22692 | Insulin-like growth factor-binding protein 4 OS=Homo sapiens OX=9606 GN=IGFBP4 PE=1 SV=2 | IGFBP4 | 27.934 | 1.83 | 0.1396 | 0.96 | 0.9889 | 1.90 | 0.1625 |
| P25705 | ATP synthase subunit alpha, mitochondrial OS=Homo sapiens OX=9606 GN=ATP5F1A PE=1 SV=1 | ATP5F1A | 59.75 | 0.96 | 0.7635 | 0.87 | 0.6690 | 1.10 | 0.9629 |
| P01700 | Immunoglobulin lambda variable 1-47 OS=Homo sapiens OX=9606 GN=IGLV1-47 PE=1 SV=2 | IGLV1-47 | 12.283 | 0.73 | 0.1721 | 0.96 | 0.6261 | 0.76 | 0.2521 |
| P01742 | Immunoglobulin heavy variable 1-69 OS=Homo sapiens OX=9606 GN=IGHV1-69 PE=1 SV=2 | IGHV1-69 | 12.659 | 0.94 | 0.9071 | 1.16 | 0.3548 | 0.80 | 0.7469 |
| O00602 | Ficolin-1 OS=Homo sapiens OX=9606 GN=FCN1 PE=1 SV=2 | FCN1 | 35.078 | 2.32 | 0.0225 | 1.61 | 0.1902 | 1.44 | 0.0028 |
| Q86UU1 | Pleckstrin homology-like domain family B member 1 OS=Homo sapiens OX=9606 GN=PHLDB1 PE=1 SV=1 | PHLDB1 | 151.16 |  |  |  |  |  |  |
| P52732 | Kinesin-like protein KIF11 OS=Homo sapiens OX=9606 GN=KIF11 PE=1 SV=2 | KIF11 | 119.16 |  |  |  |  |  |  |
| Q9NV96 | Cell cycle control protein 50A OS=Homo sapiens OX=9606 GN=TMEM30A PE=1 SV=1 | TMEM30A | 40.683 | 0.96 | 0.8655 | 0.79 | 0.5270 | 1.21 | 0.6195 |
| P32456 | Guanylate-binding protein 2 OS=Homo sapiens OX=9606 GN=GBP2 PE=1 SV=3 | GBP2 | 67.208 | 2.17 | 0.4687 | 1.10 | 0.9270 | 1.98 | 0.0228 |
| Q15517 | Corneodesmosin OS=Homo sapiens OX=9606 GN=CDSN PE=1 SV=3 | CDSN | 51.522 | 1.37 | 0.2533 | 1.06 | 0.6926 | 1.30 | 0.0460 |
| P10643 | Complement component C7 OS=Homo sapiens OX=9606 GN=C7 PE=1 SV=2 | C7 | 93.517 | 1.51 | 0.0700 | 1.25 | 0.1954 | 1.21 | 0.4044 |
| O43184 | Disintegrin and metalloproteinase domain-containing protein 12 OS=Homo sapiens OX=9606 GN=ADAM12 PE=1 SV=3 | ADAM12 | 99.541 |  |  |  |  |  |  |
| P11021 | Endoplasmic reticulum chaperone BiP OS=Homo sapiens OX=9606 GN=HSPA5 PE=1 SV=2 | HSPA5 | 72.332 | 0.70 | 0.0050 | 0.82 | 0.1329 | 0.86 | 0.1428 |
| P62987 | Ubiquitin-60S ribosomal protein L40 OS=Homo sapiens OX=9606 GN=UBA52 PE=1 SV=2 | UBA52 | 14.728 | 0.99 | 0.9543 | 0.89 | 0.4560 | 1.11 | 0.4823 |
| Q9H4G4 | Golgi-associated plant pathogenesis-related protein 1 OS=Homo sapiens OX=9606 GN=GLIPR2 PE=1 SV=3 | GLIPR2 | 17.218 | 1.64 | 0.0617 | 1.39 | 0.1851 | 1.18 | 0.7687 |
| P01833 | Polymeric immunoglobulin receptor OS=Homo sapiens OX=9606 GN=PIGR PE=1 SV=4 | PIGR | 83.283 | 1.02 | 0.7811 | 1.39 | 0.3332 | 0.73 | 0.0482 |
| Q5VT03 | NUT family member 2D OS=Homo sapiens OX=9606 GN=NUTM2D PE=3 SV=2 | NUTM2D | 86.275 | 1.07 | 0.2567 | 0.98 | 0.8763 | 1.09 | 0.3816 |
| Q9H213 | Melanoma-associated antigen H1 OS=Homo sapiens OX=9606 GN=MAGEH1 PE=1 SV=1 | MAGEH1 | 24.44 | 1.41 | 0.6938 | 1.07 | 0.9628 | 1.32 | 0.2073 |
| O43633 | Charged multivesicular body protein 2a OS=Homo sapiens OX=9606 GN=CHMP2A PE=1 SV=1 | CHMP2A | 25.104 | 1.43 | 0.7919 | 0.47 | 0.3690 | 3.05 | 0.2828 |
| P60981 | Destrin OS=Homo sapiens OX=9606 GN=DSTN PE=1 SV=3 | DSTN | 18.506 | 0.62 | 0.0687 | 0.66 | 0.2293 | 0.93 | 0.4988 |
| A6NHR9 | Structural maintenance of chromosomes flexible hinge domain-containing protein 1 OS=Homo sapiens OX=9606 GN=SMCHD1 PE=1 SV=2 | SMCHD1 | 226.37 | 1.25 | 0.1569 | 1.13 | 0.2810 | 1.11 | 0.3246 |
| P30453 | HLA class I histocompatibility antigen, A-34 alpha chain OS=Homo sapiens OX=9606 GN=HLA-A PE=1 SV=1 | HLA-A | 41.054 |  |  |  |  |  |  |
| Q9UNS1 | Protein timeless homolog OS=Homo sapiens OX=9606 GN=TIMELESS PE=1 SV=2 | TIMELESS | 138.66 | 0.15 | 0.4236 | 0.99 | 0.9290 | 0.15 | 0.4923 |
| Q04756 | Hepatocyte growth factor activator OS=Homo sapiens OX=9606 GN=HGFAC PE=1 SV=1 | HGFAC | 70.681 | 0.64 | 0.0032 | 0.86 | 0.0669 | 0.74 | 0.0111 |
| Q658P3 | Metalloreductase STEAP3 OS=Homo sapiens OX=9606 GN=STEAP3 PE=1 SV=2 | STEAP3 | 54.6 | 1.12 | 0.2685 | 1.00 | 0.7795 | 1.12 | 0.3870 |
| P23083 | Immunoglobulin heavy variable 1-2 OS=Homo sapiens OX=9606 GN=IGHV1-2 PE=1 SV=2 | IGHV1-2 | 13.085 | 2.06 | 0.1258 | 1.46 | 0.3351 | 1.41 | 0.1478 |
| Q9Y623 | Myosin-4 OS=Homo sapiens OX=9606 GN=MYH4 PE=2 SV=2 | MYH4 | 223.07 |  |  |  |  |  |  |
| P22891 | Vitamin K-dependent protein Z OS=Homo sapiens OX=9606 GN=PROZ PE=1 SV=2 | PROZ | 44.743 | 0.97 | 0.9869 | 0.87 | 0.4245 | 1.12 | 0.4959 |
| P10809 | 60 kDa heat shock protein, mitochondrial OS=Homo sapiens OX=9606 GN=HSPD1 PE=1 SV=2 | HSPD1 | 61.054 | 0.69 | 0.0837 | 0.78 | 0.4606 | 0.88 | 0.5514 |
| Q9HDB9 | Endogenous retrovirus group K member 5 Gag polyprotein OS=Homo sapiens OX=9606 GN=ERVK-5 PE=1 SV=3 | ERVK-5 | 73.587 | 1.01 | 0.8931 | 0.88 | 0.3641 | 1.15 | 0.3408 |
| Q86UF1 | Tetraspanin-33 OS=Homo sapiens OX=9606 GN=TSPAN33 PE=1 SV=1 | TSPAN33 | 31.538 | 0.64 | 0.1724 | 0.82 | 0.5897 | 0.77 | 0.2375 |
| P56278 | Protein p13 MTCP-1 OS=Homo sapiens OX=9606 GN=MTCP1 PE=1 SV=1 | MTCP1 | 12.6 |  |  |  |  |  |  |
| P26022 | Pentraxin-related protein PTX3 OS=Homo sapiens OX=9606 GN=PTX3 PE=1 SV=3 | PTX3 | 41.975 | 1.13 | 0.7540 | 1.11 | 0.6704 | 1.02 | 0.9256 |
| Q01543 | Friend leukemia integration 1 transcription factor OS=Homo sapiens OX=9606 GN=FLI1 PE=1 SV=1 | FLI1 | 50.981 | 1.29 | 0.5134 | 0.87 | 0.9696 | 1.48 | 0.6050 |
| P10720 | Platelet factor 4 variant OS=Homo sapiens OX=9606 GN=PF4V1 PE=1 SV=1 | PF4V1 | 11.553 | 0.45 | 0.0006 | 0.85 | 0.7248 | 0.53 | 0.0020 |
| O14983 | Sarcoplasmic/endoplasmic reticulum calcium ATPase 1 OS=Homo sapiens OX=9606 GN=ATP2A1 PE=1 SV=1 | ATP2A1 | 110.25 | 0.58 | 0.2368 | 0.61 | 0.1064 | 0.95 | 0.4978 |
| A0A0C4DH33 | Immunoglobulin heavy variable 1-24 OS=Homo sapiens OX=9606 GN=IGHV1-24 PE=3 SV=1 | IGHV1-24 | 12.824 | 0.90 | 0.3098 | 1.17 | 0.2908 | 0.77 | 0.0199 |
| P18206 | Vinculin OS=Homo sapiens OX=9606 GN=VCL PE=1 SV=4 | VCL | 123.8 | 0.60 | 0.0136 | 0.78 | 0.2071 | 0.76 | 0.1080 |
| Q9UMQ6 | Calpain-11 OS=Homo sapiens OX=9606 GN=CAPN11 PE=2 SV=2 | CAPN11 | 84.422 |  |  |  |  |  |  |
| Q5VTJ3 | Kelch domain-containing protein 7A OS=Homo sapiens OX=9606 GN=KLHDC7A PE=1 SV=5 | KLHDC7A | 84.478 | 0.65 | 0.0191 | 0.92 | 0.5645 | 0.71 | 0.0205 |
| O95833 | Chloride intracellular channel protein 3 OS=Homo sapiens OX=9606 GN=CLIC3 PE=1 SV=2 | CLIC3 | 26.648 | 1.98 | 0.1656 | 2.14 | 0.2213 | 0.93 | 0.5752 |
| A0A0B4J1X8 | Immunoglobulin heavy variable 3-43 OS=Homo sapiens OX=9606 GN=IGHV3-43 PE=3 SV=1 | IGHV3-43 | 13.077 | 0.58 | 0.0402 | 0.95 | 0.9966 | 0.61 | 0.0261 |
| P08133 | Annexin A6 OS=Homo sapiens OX=9606 GN=ANXA6 PE=1 SV=3 | ANXA6 | 75.872 | 1.06 | 0.7408 | 0.76 | 0.2253 | 1.40 | 0.1565 |
| Q15542 | Transcription initiation factor TFIID subunit 5 OS=Homo sapiens OX=9606 GN=TAF5 PE=1 SV=3 | TAF5 | 86.829 | 1.52 | 0.6447 | 0.97 | 0.9921 | 1.57 | 0.5642 |
| O14646 | Chromodomain-helicase-DNA-binding protein 1 OS=Homo sapiens OX=9606 GN=CHD1 PE=1 SV=2 | CHD1 | 196.69 | 0.59 | 0.3057 | 0.59 | 0.3043 | 0.99 | 0.7578 |
| P13796 | Plastin-2 OS=Homo sapiens OX=9606 GN=LCP1 PE=1 SV=6 | LCP1 | 70.288 | 0.92 | 0.7489 | 0.97 | 0.9212 | 0.94 | 0.7838 |
| Q12841 | Follistatin-related protein 1 OS=Homo sapiens OX=9606 GN=FSTL1 PE=1 SV=1 | FSTL1 | 34.985 | 0.99 | 0.9914 | 0.91 | 0.4604 | 1.10 | 0.5144 |
| P12931 | Proto-oncogene tyrosine-protein kinase Src OS=Homo sapiens OX=9606 GN=SRC PE=1 SV=3 | SRC | 59.834 | 0.98 | 0.6810 | 0.82 | 0.3310 | 1.20 | 0.3069 |
| P55072 | Transitional endoplasmic reticulum ATPase OS=Homo sapiens OX=9606 GN=VCP PE=1 SV=4 | VCP | 89.321 | 0.84 | 0.2796 | 0.74 | 0.0302 | 1.14 | 0.3792 |
| Q99805 | Transmembrane 9 superfamily member 2 OS=Homo sapiens OX=9606 GN=TM9SF2 PE=1 SV=1 | TM9SF2 | 75.775 | 0.74 | 0.2613 | 0.91 | 0.4564 | 0.81 | 0.1343 |
| Q99829 | Copine-1 OS=Homo sapiens OX=9606 GN=CPNE1 PE=1 SV=1 | CPNE1 | 59.058 | 0.97 | 0.9191 | 0.94 | 0.9977 | 1.04 | 0.9065 |
| Q9NYU2 | UDP-glucose:glycoprotein glucosyltransferase 1 OS=Homo sapiens OX=9606 GN=UGGT1 PE=1 SV=3 | UGGT1 | 177.19 | 0.79 | 0.4711 | 0.93 | 0.7245 | 0.85 | 0.3867 |
| P02753 | Retinol-binding protein 4 OS=Homo sapiens OX=9606 GN=RBP4 PE=1 SV=3 | RBP4 | 23.01 | 1.17 | 0.2546 | 1.08 | 0.3143 | 1.08 | 0.3246 |
| O00187 | Mannan-binding lectin serine protease 2 OS=Homo sapiens OX=9606 GN=MASP2 PE=1 SV=4 | MASP2 | 75.702 | 1.19 | 0.0806 | 1.21 | 0.0224 | 0.98 | 0.7362 |
| Q12907 | Vesicular integral-membrane protein VIP36 OS=Homo sapiens OX=9606 GN=LMAN2 PE=1 SV=1 | LMAN2 | 40.228 | 0.89 | 0.6743 | 0.95 | 0.6392 | 0.94 | 0.7515 |
| P02746 | Complement C1q subcomponent subunit B OS=Homo sapiens OX=9606 GN=C1QB PE=1 SV=3 | C1QB | 26.721 | 1.00 | 0.9977 | 0.95 | 0.3047 | 1.06 | 0.3161 |
| P13473 | Lysosome-associated membrane glycoprotein 2 OS=Homo sapiens OX=9606 GN=LAMP2 PE=1 SV=2 | LAMP2 | 44.96 | 0.71 | 0.3709 | 0.87 | 0.5218 | 0.83 | 0.3538 |
| Q9UPS8 | Ankyrin repeat domain-containing protein 26 OS=Homo sapiens OX=9606 GN=ANKRD26 PE=1 SV=3 | ANKRD26 | 196.32 | 1.23 |  | 1.00 | 0.9559 | 1.23 |  |
| O75531 | Barrier-to-autointegration factor OS=Homo sapiens OX=9606 GN=BANF1 PE=1 SV=1 | BANF1 | 10.058 | 1.07 | 0.9728 | 0.77 | 0.4633 | 1.40 | 0.3834 |
| Q9H0K1 | Serine/threonine-protein kinase SIK2 OS=Homo sapiens OX=9606 GN=SIK2 PE=1 SV=1 | SIK2 | 103.91 | 0.41 | 0.2176 | 0.58 | 0.0067 | 0.70 | 0.6634 |
| Q99808 | Equilibrative nucleoside transporter 1 OS=Homo sapiens OX=9606 GN=SLC29A1 PE=1 SV=3 | SLC29A1 | 50.219 | 1.30 | 0.1035 | 0.86 | 0.4628 | 1.50 | 0.0501 |
| P62491 | Ras-related protein Rab-11A OS=Homo sapiens OX=9606 GN=RAB11A PE=1 SV=3 | RAB11A | 24.393 | 0.76 | 0.3111 | 0.77 | 0.3556 | 0.98 | 0.8128 |
| Q70J99 | Protein unc-13 homolog D OS=Homo sapiens OX=9606 GN=UNC13D PE=1 SV=1 | UNC13D | 123.28 | 0.94 | 0.7820 | 0.71 | 0.4015 | 1.32 | 0.5974 |
| P62136 | Serine/threonine-protein phosphatase PP1-alpha catalytic subunit OS=Homo sapiens OX=9606 GN=PPP1CA PE=1 SV=1 | PPP1CA | 37.512 | 0.84 | 0.2617 | 0.80 | 0.3855 | 1.05 | 0.9181 |
| Q14766 | Latent-transforming growth factor beta-binding protein 1 OS=Homo sapiens OX=9606 GN=LTBP1 PE=1 SV=4 | LTBP1 | 186.79 | 1.18 | 0.1977 | 0.90 | 0.3684 | 1.31 | 0.0329 |
| Q8WUM4 | Programmed cell death 6-interacting protein OS=Homo sapiens OX=9606 GN=PDCD6IP PE=1 SV=1 | PDCD6IP | 96.022 | 1.00 | 0.8057 | 0.94 | 0.8048 | 1.06 | 0.6559 |
| P49908 | Selenoprotein P OS=Homo sapiens OX=9606 GN=SELENOP PE=1 SV=3 | SELENOP | 43.173 | 0.87 | 0.4142 | 0.82 | 0.2291 | 1.06 | 0.8154 |
| O75695 | Protein XRP2 OS=Homo sapiens OX=9606 GN=RP2 PE=1 SV=4 | RP2 | 39.641 | 0.94 | 0.8654 | 0.75 | 0.7260 | 1.25 | 0.8306 |
| Q99973 | Telomerase protein component 1 OS=Homo sapiens OX=9606 GN=TEP1 PE=1 SV=2 | TEP1 | 290.49 | 1.51 | 0.3315 | 0.99 | 0.9306 | 1.52 | 0.2824 |
| P62942 | Peptidyl-prolyl cis-trans isomerase FKBP1A OS=Homo sapiens OX=9606 GN=FKBP1A PE=1 SV=2 | FKBP1A | 11.951 | 0.84 | 0.4542 | 0.89 | 0.7902 | 0.95 | 0.7144 |
| Q99969 | Retinoic acid receptor responder protein 2 OS=Homo sapiens OX=9606 GN=RARRES2 PE=1 SV=1 | RARRES2 | 18.617 | 1.80 | 0.0773 | 1.14 | 0.4509 | 1.58 | 0.0224 |
| A0A087WW87 | Immunoglobulin kappa variable 2-40 OS=Homo sapiens OX=9606 GN=IGKV2-40 PE=3 SV=2 | IGKV2-40 | 13.31 | 0.91 | 0.4669 | 0.94 | 0.5559 | 0.98 | 0.7526 |
| Q6ZUX7 | LHFPL tetraspan subfamily member 2 protein OS=Homo sapiens OX=9606 GN=LHFPL2 PE=2 SV=2 | LHFPL2 | 24.486 | 0.91 | 0.4016 | 0.92 | 0.4812 | 0.98 | 0.8094 |
| O94986 | Centrosomal protein of 152 kDa OS=Homo sapiens OX=9606 GN=CEP152 PE=1 SV=4 | CEP152 | 195.62 | 0.20 | 0.0258 | 0.60 | 0.3086 | 0.34 | 0.0623 |
| P29350 | Tyrosine-protein phosphatase non-receptor type 6 OS=Homo sapiens OX=9606 GN=PTPN6 PE=1 SV=1 | PTPN6 | 67.56 | 0.81 | 0.1454 | 0.91 | 0.5583 | 0.89 | 0.3926 |
| P04430 | Immunoglobulin kappa variable 1-16 OS=Homo sapiens OX=9606 GN=IGKV1-16 PE=1 SV=2 | IGKV1-16 | 12.618 | 0.58 | 0.0932 | 0.81 | 0.2495 | 0.72 | 0.1160 |
| P08571 | Monocyte differentiation antigen CD14 OS=Homo sapiens OX=9606 GN=CD14 PE=1 SV=2 | CD14 | 40.076 | 0.95 | 0.8394 | 1.01 | 0.8657 | 0.93 | 0.6889 |
| P02749 | Beta-2-glycoprotein 1 OS=Homo sapiens OX=9606 GN=APOH PE=1 SV=3 | APOH | 38.298 | 0.93 | 0.6165 | 0.78 | 0.2063 | 1.19 | 0.4757 |
| P04792 | Heat shock protein beta-1 OS=Homo sapiens OX=9606 GN=HSPB1 PE=1 SV=2 | HSPB1 | 22.782 | 1.08 | 0.7989 | 0.81 | 0.1973 | 1.33 | 0.0575 |
| P0DOX2 | Immunoglobulin alpha-2 heavy chain OS=Homo sapiens OX=9606 PE=1 SV=2 | --- | 48.934 | 0.78 | 0.2235 | 1.08 | 0.5392 | 0.72 | 0.0433 |
| A2VDJ0 | Transmembrane protein 131-like OS=Homo sapiens OX=9606 GN=TMEM131L PE=1 SV=2 | TMEM131L | 179.34 | 1.44 | 0.0527 | 1.19 | 0.3111 | 1.20 | 0.3957 |
| P14923 | Junction plakoglobin OS=Homo sapiens OX=9606 GN=JUP PE=1 SV=3 | JUP | 81.744 | 1.37 | 0.5016 | 1.01 | 0.8893 | 1.37 | 0.3141 |
| Q6WKZ4 | Rab11 family-interacting protein 1 OS=Homo sapiens OX=9606 GN=RAB11FIP1 PE=1 SV=3 | RAB11FIP1 | 137.17 | 0.87 | 0.2776 | 0.99 | 0.8070 | 0.88 | 0.1198 |
| P02760 | Protein AMBP OS=Homo sapiens OX=9606 GN=AMBP PE=1 SV=1 | AMBP | 38.999 | 1.14 | 0.0728 | 1.05 | 0.3150 | 1.08 | 0.1214 |
| P28289 | Tropomodulin-1 OS=Homo sapiens OX=9606 GN=TMOD1 PE=1 SV=1 | TMOD1 | 40.569 | 1.10 | 0.8596 | 1.19 | 0.5073 | 0.92 | 0.3317 |
| P00746 | Complement factor D OS=Homo sapiens OX=9606 GN=CFD PE=1 SV=5 | CFD | 27.033 | 1.18 | 0.2594 | 0.93 | 0.5355 | 1.27 | 0.0085 |
| P46531 | Neurogenic locus notch homolog protein 1 OS=Homo sapiens OX=9606 GN=NOTCH1 PE=1 SV=4 | NOTCH1 | 272.5 | 0.97 | 0.8875 | 1.27 | 0.3600 | 0.76 | 0.3062 |
| Q6P158 | Putative ATP-dependent RNA helicase DHX57 OS=Homo sapiens OX=9606 GN=DHX57 PE=1 SV=2 | DHX57 | 155.6 | 0.73 | 0.3862 | 1.44 | 0.3104 | 0.51 | 0.0388 |
| Q86W50 | U6 small nuclear RNA (adenine-(43)-N(6))-methyltransferase OS=Homo sapiens OX=9606 GN=METTL16 PE=1 SV=2 | METTL16 | 63.62 |  |  |  |  |  |  |
| P00739 | Haptoglobin-related protein OS=Homo sapiens OX=9606 GN=HPR PE=2 SV=2 | HPR | 39.029 | 0.59 | 0.0069 | 1.01 | 0.9983 | 0.58 | 0.0005 |
| P19086 | Guanine nucleotide-binding protein G(z) subunit alpha OS=Homo sapiens OX=9606 GN=GNAZ PE=1 SV=3 | GNAZ | 40.923 | 0.67 | 0.0394 | 0.72 | 0.1148 | 0.94 | 0.3279 |
| P00734 | Prothrombin OS=Homo sapiens OX=9606 GN=F2 PE=1 SV=2 | F2 | 70.036 | 1.04 | 0.7920 | 0.92 | 0.3771 | 1.13 | 0.2220 |
| Q9BUZ4 | TNF receptor-associated factor 4 OS=Homo sapiens OX=9606 GN=TRAF4 PE=1 SV=1 | TRAF4 | 53.542 | 1.16 | 0.3969 | 1.09 | 0.5458 | 1.07 | 0.5982 |
| P22392 | Nucleoside diphosphate kinase B OS=Homo sapiens OX=9606 GN=NME2 PE=1 SV=1 | NME2 | 17.298 | 0.69 | 0.1503 | 0.83 | 0.3515 | 0.83 | 0.3503 |
| P61586 | Transforming protein RhoA OS=Homo sapiens OX=9606 GN=RHOA PE=1 SV=1 | RHOA | 21.768 | 0.70 | 0.0132 | 0.85 | 0.1999 | 0.83 | 0.0992 |
| Q8N1K5 | Protein THEMIS OS=Homo sapiens OX=9606 GN=THEMIS PE=1 SV=3 | THEMIS | 73.451 | 2.32 | 0.6743 | 0.67 | 0.9962 | 3.44 | 0.6899 |
| P16189 | HLA class I histocompatibility antigen, A-31 alpha chain OS=Homo sapiens OX=9606 GN=HLA-A PE=1 SV=2 | HLA-A | 41.003 | 0.69 | 0.6821 | 1.16 | 0.6514 | 0.59 | 0.4149 |
| Q06033 | Inter-alpha-trypsin inhibitor heavy chain H3 OS=Homo sapiens OX=9606 GN=ITIH3 PE=1 SV=2 | ITIH3 | 99.848 | 1.23 | 0.2539 | 1.16 | 0.2705 | 1.06 | 0.6419 |
| P00918 | Carbonic anhydrase 2 OS=Homo sapiens OX=9606 GN=CA2 PE=1 SV=2 | CA2 | 29.246 | 0.80 | 0.0711 | 0.82 | 0.1951 | 0.97 | 0.4924 |
| P07360 | Complement component C8 gamma chain OS=Homo sapiens OX=9606 GN=C8G PE=1 SV=3 | C8G | 22.277 | 1.05 | 0.8529 | 1.19 | 0.1640 | 0.88 | 0.2294 |
| P13224 | Platelet glycoprotein Ib beta chain OS=Homo sapiens OX=9606 GN=GP1BB PE=1 SV=1 | GP1BB | 21.717 | 0.53 | 0.0192 | 0.82 | 0.4482 | 0.65 | 0.0359 |
| Q96L03 | Spermatogenesis-associated protein 17 OS=Homo sapiens OX=9606 GN=SPATA17 PE=2 SV=1 | SPATA17 | 43.499 |  |  |  |  |  |  |
| Q1MSJ5 | Centrosome and spindle pole-associated protein 1 OS=Homo sapiens OX=9606 GN=CSPP1 PE=1 SV=4 | CSPP1 | 145.52 | 1.13 | 0.7049 | 1.02 | 0.8875 | 1.11 | 0.6872 |
| P56557 | Transmembrane protein 50B OS=Homo sapiens OX=9606 GN=TMEM50B PE=1 SV=2 | TMEM50B | 17.936 | 0.89 | 0.6757 | 0.92 | 0.7887 | 0.97 | 0.8310 |
| Q8IZP0 | Abl interactor 1 OS=Homo sapiens OX=9606 GN=ABI1 PE=1 SV=4 | ABI1 | 55.08 | 0.87 | 0.0961 | 0.68 | 0.1960 | 1.28 | 0.4242 |
| Q6UX06 | Olfactomedin-4 OS=Homo sapiens OX=9606 GN=OLFM4 PE=1 SV=1 | OLFM4 | 57.279 | 2.08 | 0.2810 | 1.21 | 0.8801 | 1.72 | 0.1416 |
| P17301 | Integrin alpha-2 OS=Homo sapiens OX=9606 GN=ITGA2 PE=1 SV=1 | ITGA2 | 129.29 | 0.78 | 0.1544 | 0.69 | 0.1897 | 1.13 | 0.8357 |
| O75427 | Leucine-rich repeat and calponin homology domain-containing protein 4 OS=Homo sapiens OX=9606 GN=LRCH4 PE=1 SV=2 | LRCH4 | 73.449 | 0.20 | 0.2879 | 0.81 | 0.5340 | 0.24 | 0.0599 |
| Q6ZNX1 | Shieldin complex subunit 3 OS=Homo sapiens OX=9606 GN=SHLD3 PE=1 SV=1 | SHLD3 | 28.842 |  |  |  |  |  |  |
| Q14643 | Inositol 1,4,5-trisphosphate receptor type 1 OS=Homo sapiens OX=9606 GN=ITPR1 PE=1 SV=3 | ITPR1 | 313.93 | 0.43 | 0.0551 | 0.66 | 0.4198 | 0.65 | 0.2669 |
| P01859 | Immunoglobulin heavy constant gamma 2 OS=Homo sapiens OX=9606 GN=IGHG2 PE=1 SV=2 | IGHG2 | 35.9 | 0.69 | 0.0037 | 0.73 | 0.2143 | 0.95 | 0.2366 |
| Q15836 | Vesicle-associated membrane protein 3 OS=Homo sapiens OX=9606 GN=VAMP3 PE=1 SV=3 | VAMP3 | 11.309 | 0.89 | 0.4628 | 0.73 | 0.2016 | 1.21 | 0.5234 |
| Q9BXJ4 | Complement C1q tumor necrosis factor-related protein 3 OS=Homo sapiens OX=9606 GN=C1QTNF3 PE=1 SV=1 | C1QTNF3 | 26.994 | 1.10 | 0.5356 | 1.01 | 0.9520 | 1.09 | 0.2400 |
| P12109 | Collagen alpha-1(VI) chain OS=Homo sapiens OX=9606 GN=COL6A1 PE=1 SV=3 | COL6A1 | 108.53 | 1.09 | 0.4647 | 0.93 | 0.3348 | 1.17 | 0.0589 |
| P07996 | Thrombospondin-1 OS=Homo sapiens OX=9606 GN=THBS1 PE=1 SV=2 | THBS1 | 129.38 | 0.87 | 0.2249 | 0.68 | 0.3639 | 1.28 | 0.6153 |
| P13497 | Bone morphogenetic protein 1 OS=Homo sapiens OX=9606 GN=BMP1 PE=1 SV=2 | BMP1 | 111.25 | 0.83 | 0.0764 | 0.98 | 0.7795 | 0.85 | 0.0865 |
| Q93084 | Sarcoplasmic/endoplasmic reticulum calcium ATPase 3 OS=Homo sapiens OX=9606 GN=ATP2A3 PE=1 SV=2 | ATP2A3 | 113.98 | 0.82 | 0.2038 | 0.82 | 0.4029 | 1.00 | 0.6053 |
| Q9NVF7 | F-box only protein 28 OS=Homo sapiens OX=9606 GN=FBXO28 PE=1 SV=1 | FBXO28 | 41.149 | 1.79 | 0.0494 | 0.88 | 0.8655 | 2.05 | 0.0641 |
| Q9ULV4 | Coronin-1C OS=Homo sapiens OX=9606 GN=CORO1C PE=1 SV=1 | CORO1C | 53.248 | 0.66 | 0.0321 | 0.89 | 0.4912 | 0.74 | 0.0541 |
| P02774 | Vitamin D-binding protein OS=Homo sapiens OX=9606 GN=GC PE=1 SV=1 | GC | 52.963 | 0.74 | 0.0051 | 0.88 | 0.2105 | 0.84 | 0.0682 |
| P00450 | Ceruloplasmin OS=Homo sapiens OX=9606 GN=CP PE=1 SV=1 | CP | 122.2 | 1.59 | 0.1350 | 1.22 | 0.4401 | 1.31 | 0.1773 |
| P50991 | T-complex protein 1 subunit delta OS=Homo sapiens OX=9606 GN=CCT4 PE=1 SV=4 | CCT4 | 57.924 | 0.66 | 0.0095 | 0.77 | 0.1353 | 0.86 | 0.2425 |
| P10301 | Ras-related protein R-Ras OS=Homo sapiens OX=9606 GN=RRAS PE=1 SV=1 | RRAS | 23.48 | 0.54 | 0.1531 | 0.79 | 0.2599 | 0.68 | 0.1252 |
| P55145 | Mesencephalic astrocyte-derived neurotrophic factor OS=Homo sapiens OX=9606 GN=MANF PE=1 SV=3 | MANF | 20.7 | 0.97 | 0.7482 | 0.82 | 0.5544 | 1.19 | 0.8380 |
| Q6P3R8 | Serine/threonine-protein kinase Nek5 OS=Homo sapiens OX=9606 GN=NEK5 PE=2 SV=1 | NEK5 | 81.445 |  |  |  |  |  |  |
| P0DJI9 | Serum amyloid A-2 protein OS=Homo sapiens OX=9606 GN=SAA2 PE=1 SV=1 | SAA2 | 13.527 | 1.70 | 0.1014 | 1.58 | 0.1561 | 1.08 | 0.3467 |
| A2RUS2 | DENN domain-containing protein 3 OS=Homo sapiens OX=9606 GN=DENND3 PE=1 SV=2 | DENND3 | 135.89 |  |  |  |  |  |  |
| O43451 | Maltase-glucoamylase, intestinal OS=Homo sapiens OX=9606 GN=MGAM PE=1 SV=5 | MGAM | 209.85 |  |  |  |  |  |  |
| Q5T619 | Zinc finger protein 648 OS=Homo sapiens OX=9606 GN=ZNF648 PE=2 SV=1 | ZNF648 | 62.34 | 3.12 | 0.1470 | 1.62 | 0.4583 | 1.93 | 0.0091 |
| O14867 | Transcription regulator protein BACH1 OS=Homo sapiens OX=9606 GN=BACH1 PE=1 SV=2 | BACH1 | 81.957 |  |  |  |  |  |  |
| A6NMZ7 | Collagen alpha-6(VI) chain OS=Homo sapiens OX=9606 GN=COL6A6 PE=1 SV=2 | COL6A6 | 247.17 |  |  | 1.01 | 0.9470 |  |  |
| P07358 | Complement component C8 beta chain OS=Homo sapiens OX=9606 GN=C8B PE=1 SV=3 | C8B | 67.046 | 1.06 | 0.8052 | 1.35 | 0.0364 | 0.79 | 0.0748 |
| P14209 | CD99 antigen OS=Homo sapiens OX=9606 GN=CD99 PE=1 SV=1 | CD99 | 18.848 | 0.89 | 0.5412 | 0.96 | 0.9862 | 0.93 | 0.6015 |
| P01703 | Immunoglobulin lambda variable 1-40 OS=Homo sapiens OX=9606 GN=IGLV1-40 PE=1 SV=2 | IGLV1-40 | 12.301 | 0.86 | 0.2089 | 0.91 | 0.5119 | 0.95 | 0.2979 |
| P32119 | Peroxiredoxin-2 OS=Homo sapiens OX=9606 GN=PRDX2 PE=1 SV=5 | PRDX2 | 21.892 | 1.37 | 0.3771 | 1.05 | 0.9196 | 1.31 | 0.3210 |
| O15078 | Centrosomal protein of 290 kDa OS=Homo sapiens OX=9606 GN=CEP290 PE=1 SV=2 | CEP290 | 290.38 | 1.69 | 0.0682 | 1.15 | 0.3622 | 1.47 | 0.0227 |
| Q8IWV8 | E3 ubiquitin-protein ligase UBR2 OS=Homo sapiens OX=9606 GN=UBR2 PE=1 SV=1 | UBR2 | 200.54 | 0.96 | 0.9490 | 0.66 | 0.1402 | 1.46 | 0.1769 |
| Q3L8U1 | Chromodomain-helicase-DNA-binding protein 9 OS=Homo sapiens OX=9606 GN=CHD9 PE=1 SV=2 | CHD9 | 326.02 | 0.56 | 0.1314 | 0.88 | 0.4351 | 0.64 | 0.0152 |
| O75563 | Src kinase-associated phosphoprotein 2 OS=Homo sapiens OX=9606 GN=SKAP2 PE=1 SV=1 | SKAP2 | 41.216 |  |  |  |  |  |  |
| P13987 | CD59 glycoprotein OS=Homo sapiens OX=9606 GN=CD59 PE=1 SV=1 | CD59 | 14.177 | 0.92 | 0.3837 | 0.99 | 0.8731 | 0.93 | 0.5133 |
| Q96AP7 | Endothelial cell-selective adhesion molecule OS=Homo sapiens OX=9606 GN=ESAM PE=1 SV=1 | ESAM | 41.176 | 0.84 | 0.2951 | 0.62 | 0.1579 | 1.35 | 0.5284 |
| Q5VZK9 | F-actin-uncapping protein LRRC16A OS=Homo sapiens OX=9606 GN=CARMIL1 PE=1 SV=1 | CARMIL1 | 151.56 | 0.71 | 0.1514 | 1.02 | 0.8366 | 0.70 | 0.1478 |
| Q05586 | Glutamate receptor ionotropic, NMDA 1 OS=Homo sapiens OX=9606 GN=GRIN1 PE=1 SV=1 | GRIN1 | 105.37 | 0.72 | 0.0121 | 0.83 | 0.0528 | 0.87 | 0.1375 |
| P50895 | Basal cell adhesion molecule OS=Homo sapiens OX=9606 GN=BCAM PE=1 SV=2 | BCAM | 67.404 | 0.97 | 0.8061 | 0.92 | 0.6473 | 1.05 | 0.8964 |
| Q08722 | Leukocyte surface antigen CD47 OS=Homo sapiens OX=9606 GN=CD47 PE=1 SV=1 | CD47 | 35.213 | 0.77 | 0.0970 | 0.78 | 0.2097 | 0.98 | 0.7029 |
| P63279 | SUMO-conjugating enzyme UBC9 OS=Homo sapiens OX=9606 GN=UBE2I PE=1 SV=1 | UBE2I | 18.007 |  |  |  |  |  |  |
| Q9NRY6 | Phospholipid scramblase 3 OS=Homo sapiens OX=9606 GN=PLSCR3 PE=1 SV=2 | PLSCR3 | 31.648 |  |  |  |  |  |  |
| O14960 | Leukocyte cell-derived chemotaxin-2 OS=Homo sapiens OX=9606 GN=LECT2 PE=1 SV=2 | LECT2 | 16.39 | 1.33 | 0.5442 | 1.05 | 0.8300 | 1.27 | 0.6867 |
| O00506 | Serine/threonine-protein kinase 25 OS=Homo sapiens OX=9606 GN=STK25 PE=1 SV=1 | STK25 | 48.111 | 0.66 | 0.2465 | 0.70 | 0.2778 | 0.95 | 0.7238 |
| Q12846 | Syntaxin-4 OS=Homo sapiens OX=9606 GN=STX4 PE=1 SV=2 | STX4 | 34.18 | 1.18 | 0.5183 | 0.85 | 0.8645 | 1.38 | 0.4833 |
| P00387 | NADH-cytochrome b5 reductase 3 OS=Homo sapiens OX=9606 GN=CYB5R3 PE=1 SV=3 | CYB5R3 | 34.234 | 0.74 | 0.1727 | 0.70 | 0.1899 | 1.06 | 0.9347 |
| Q9UPA5 | Protein bassoon OS=Homo sapiens OX=9606 GN=BSN PE=1 SV=4 | BSN | 416.46 | 0.70 | 0.7512 | 2.07 | 0.7786 | 0.34 | 0.5066 |
| P57773 | Gap junction alpha-9 protein OS=Homo sapiens OX=9606 GN=GJA9 PE=2 SV=2 | GJA9 | 58.842 | 1.16 | 0.3771 | 1.20 | 0.2940 | 0.96 | 0.7532 |
| P55160 | Nck-associated protein 1-like OS=Homo sapiens OX=9606 GN=NCKAP1L PE=1 SV=3 | NCKAP1L | 128.15 | 0.67 | 0.0522 | 0.65 | 0.0262 | 1.03 | 0.9188 |
| P04040 | Catalase OS=Homo sapiens OX=9606 GN=CAT PE=1 SV=3 | CAT | 59.755 | 1.13 | 0.2309 | 0.94 | 0.4408 | 1.20 | 0.0309 |
| P48059 | LIM and senescent cell antigen-like-containing domain protein 1 OS=Homo sapiens OX=9606 GN=LIMS1 PE=1 SV=4 | LIMS1 | 37.251 | 0.57 | 0.0288 | 0.77 | 0.1584 | 0.75 | 0.1391 |
| A0A0C4DH43 | Immunoglobulin heavy variable 2-70D OS=Homo sapiens OX=9606 GN=IGHV2-70D PE=3 SV=1 | IGHV2-70D | 13.312 | 1.04 | 0.4949 | 1.10 | 0.4884 | 0.95 | 0.7197 |
| Q09666 | Neuroblast differentiation-associated protein AHNAK OS=Homo sapiens OX=9606 GN=AHNAK PE=1 SV=2 | AHNAK | 629.09 | 1.12 | 0.6299 | 1.00 | 0.9614 | 1.12 | 0.6503 |
| P01871 | Immunoglobulin heavy constant mu OS=Homo sapiens OX=9606 GN=IGHM PE=1 SV=4 | IGHM | 49.439 | 0.70 | 0.0249 | 0.93 | 0.4468 | 0.76 | 0.0235 |
| P63261 | Actin, cytoplasmic 2 OS=Homo sapiens OX=9606 GN=ACTG1 PE=1 SV=1 | ACTG1 | 41.792 | 0.66 | 0.0608 | 0.80 | 0.1648 | 0.82 | 0.2256 |
| Q86UX7 | Fermitin family homolog 3 OS=Homo sapiens OX=9606 GN=FERMT3 PE=1 SV=1 | FERMT3 | 75.952 | 0.58 | 0.0146 | 0.76 | 0.1088 | 0.76 | 0.0863 |
| P43652 | Afamin OS=Homo sapiens OX=9606 GN=AFM PE=1 SV=1 | AFM | 69.068 | 0.86 | 0.2209 | 0.70 | 0.0237 | 1.23 | 0.2482 |
| P21579 | Synaptotagmin-1 OS=Homo sapiens OX=9606 GN=SYT1 PE=1 SV=1 | SYT1 | 47.573 |  |  |  |  |  |  |
| P04839 | Cytochrome b-245 heavy chain OS=Homo sapiens OX=9606 GN=CYBB PE=1 SV=2 | CYBB | 65.335 | 1.05 | 0.7884 | 0.80 | 0.5714 | 1.32 | 0.4415 |
| A0A0G2JS06 | Immunoglobulin lambda variable 5-39 OS=Homo sapiens OX=9606 GN=IGLV5-39 PE=3 SV=1 | IGLV5-39 | 13.394 | 0.89 | 0.4082 | 0.76 | 0.1144 | 1.16 | 0.6005 |
| Q96AY3 | Peptidyl-prolyl cis-trans isomerase FKBP10 OS=Homo sapiens OX=9606 GN=FKBP10 PE=1 SV=1 | FKBP10 | 64.244 | 1.19 | 0.2577 | 0.51 | 0.5993 | 2.34 | 0.4294 |
| Q9Y2A7 | Nck-associated protein 1 OS=Homo sapiens OX=9606 GN=NCKAP1 PE=1 SV=1 | NCKAP1 | 128.79 | 0.90 | 0.1360 | 0.97 | 0.9916 | 0.93 | 0.6253 |
| P01780 | Immunoglobulin heavy variable 3-7 OS=Homo sapiens OX=9606 GN=IGHV3-7 PE=1 SV=2 | IGHV3-7 | 12.943 | 0.60 | 0.0034 | 0.94 | 0.7343 | 0.63 | 0.0027 |
| O15018 | PDZ domain-containing protein 2 OS=Homo sapiens OX=9606 GN=PDZD2 PE=1 SV=4 | PDZD2 | 301.64 | 0.88 | 0.5770 | 0.99 | 0.8728 | 0.89 | 0.3747 |
| Q5VXU9 | Protein shortage in chiasmata 1 ortholog OS=Homo sapiens OX=9606 GN=SHOC1 PE=1 SV=1 | SHOC1 | 165.2 |  |  |  |  |  |  |
| O00139 | Kinesin-like protein KIF2A OS=Homo sapiens OX=9606 GN=KIF2A PE=1 SV=3 | KIF2A | 79.954 | 0.70 | 0.0275 | 0.76 | 0.1702 | 0.92 | 0.4993 |
| Q8TD26 | Chromodomain-helicase-DNA-binding protein 6 OS=Homo sapiens OX=9606 GN=CHD6 PE=1 SV=4 | CHD6 | 305.41 | 2.74 | 0.3253 | 0.71 | 0.7740 | 3.87 | 0.1694 |
| Q8WVN8 | Ubiquitin-conjugating enzyme E2 Q2 OS=Homo sapiens OX=9606 GN=UBE2Q2 PE=1 SV=1 | UBE2Q2 | 42.818 | 2.12 | 0.3907 | 1.22 | 0.7982 | 1.74 | 0.0860 |
| P01709 | Immunoglobulin lambda variable 2-8 OS=Homo sapiens OX=9606 GN=IGLV2-8 PE=1 SV=2 | IGLV2-8 | 12.382 | 0.64 | 0.0399 | 1.08 | 0.6666 | 0.59 | 0.0019 |
| O75912 | Diacylglycerol kinase iota OS=Homo sapiens OX=9606 GN=DGKI PE=1 SV=1 | DGKI | 117 |  |  |  |  |  |  |
| P16150 | Leukosialin OS=Homo sapiens OX=9606 GN=SPN PE=1 SV=1 | SPN | 40.321 | 1.05 | 0.7816 | 0.91 | 0.9141 | 1.16 | 0.6819 |
| O75636 | Ficolin-3 OS=Homo sapiens OX=9606 GN=FCN3 PE=1 SV=2 | FCN3 | 32.903 | 0.98 | 0.8769 | 1.05 | 0.5506 | 0.93 | 0.4996 |
| Q6ZW61 | Bardet-Biedl syndrome 12 protein OS=Homo sapiens OX=9606 GN=BBS12 PE=1 SV=2 | BBS12 | 79.084 | 1.00 | 0.7651 | 0.82 | 0.3785 | 1.23 | 0.2648 |
| P00558 | Phosphoglycerate kinase 1 OS=Homo sapiens OX=9606 GN=PGK1 PE=1 SV=3 | PGK1 | 44.614 | 0.85 | 0.1981 | 0.93 | 0.3696 | 0.92 | 0.3111 |
| Q9NSI6 | Bromodomain and WD repeat-containing protein 1 OS=Homo sapiens OX=9606 GN=BRWD1 PE=1 SV=4 | BRWD1 | 262.93 | 0.62 | 0.2324 | 0.94 | 0.5437 | 0.66 | 0.0719 |
| P0DOX4 | Immunoglobulin epsilon heavy chain OS=Homo sapiens OX=9606 PE=1 SV=1 | --- | 60.322 | 1.01 | 0.9501 | 0.70 | 0.3054 | 1.45 | 0.3219 |
| Q7Z333 | Probable helicase senataxin OS=Homo sapiens OX=9606 GN=SETX PE=1 SV=4 | SETX | 302.88 | 1.23 | 0.4764 | 0.99 | 0.7524 | 1.24 | 0.1650 |
| Q5VU43 | Myomegalin OS=Homo sapiens OX=9606 GN=PDE4DIP PE=1 SV=3 | PDE4DIP | 265.1 |  |  |  |  |  |  |
| Q9UGM3 | Deleted in malignant brain tumors 1 protein OS=Homo sapiens OX=9606 GN=DMBT1 PE=1 SV=2 | DMBT1 | 260.73 | 2.16 | 0.0937 | 1.18 | 0.4701 | 1.83 | 0.5728 |
| P31146 | Coronin-1A OS=Homo sapiens OX=9606 GN=CORO1A PE=1 SV=4 | CORO1A | 51.026 | 0.66 | 0.0152 | 0.82 | 0.1743 | 0.81 | 0.1084 |
| O00391 | Sulfhydryl oxidase 1 OS=Homo sapiens OX=9606 GN=QSOX1 PE=1 SV=3 | QSOX1 | 82.577 | 0.95 | 0.9627 | 0.95 | 0.6273 | 1.01 | 0.7932 |
| O94885 | SAM and SH3 domain-containing protein 1 OS=Homo sapiens OX=9606 GN=SASH1 PE=1 SV=3 | SASH1 | 136.65 | 0.70 | 0.1500 | 1.17 | 0.4881 | 0.60 | 0.0555 |
| P61026 | Ras-related protein Rab-10 OS=Homo sapiens OX=9606 GN=RAB10 PE=1 SV=1 | RAB10 | 22.541 |  |  |  |  |  |  |
| Q16555 | Dihydropyrimidinase-related protein 2 OS=Homo sapiens OX=9606 GN=DPYSL2 PE=1 SV=1 | DPYSL2 | 62.293 |  |  |  |  |  |  |
| Q8WUY9 | DEP domain-containing protein 1B OS=Homo sapiens OX=9606 GN=DEPDC1B PE=1 SV=2 | DEPDC1B | 61.77 | 1.03 | 0.8284 | 0.75 | 0.2355 | 1.38 | 0.1982 |
| O00299 | Chloride intracellular channel protein 1 OS=Homo sapiens OX=9606 GN=CLIC1 PE=1 SV=4 | CLIC1 | 26.922 | 0.54 | 0.0063 | 0.76 | 0.1815 | 0.71 | 0.0549 |
| P51575 | P2X purinoceptor 1 OS=Homo sapiens OX=9606 GN=P2RX1 PE=1 SV=1 | P2RX1 | 44.98 | 0.83 | 0.3300 | 0.73 | 0.2723 | 1.14 | 0.9965 |
| Q8NFI4 | Putative protein FAM10A5 OS=Homo sapiens OX=9606 GN=ST13P5 PE=5 SV=1 | ST13P5 | 41.377 | 1.00 | 0.9389 | 0.76 | 0.3849 | 1.33 | 0.3918 |
| Q7RTS9 | Dymeclin OS=Homo sapiens OX=9606 GN=DYM PE=1 SV=1 | DYM | 75.935 | 0.72 | 0.5307 | 0.95 | 0.9092 | 0.76 | 0.3913 |
| P35557 | Glucokinase OS=Homo sapiens OX=9606 GN=GCK PE=1 SV=1 | GCK | 52.191 | 0.97 | 0.2628 | 0.88 | 0.2466 | 1.11 | 0.3553 |
| Q9BX67 | Junctional adhesion molecule C OS=Homo sapiens OX=9606 GN=JAM3 PE=1 SV=1 | JAM3 | 35.02 | 0.90 | 0.9391 | 0.93 | 0.6528 | 0.97 | 0.8783 |
| Q9HBI1 | Beta-parvin OS=Homo sapiens OX=9606 GN=PARVB PE=1 SV=1 | PARVB | 41.714 | 0.55 | 0.0347 | 0.82 | 0.3146 | 0.67 | 0.0528 |
| O15049 | NEDD4-binding protein 3 OS=Homo sapiens OX=9606 GN=N4BP3 PE=1 SV=3 | N4BP3 | 60.469 | 0.22 | 0.0375 | 1.22 | 0.6872 | 0.18 | 0.0057 |
| P11171 | Protein 4.1 OS=Homo sapiens OX=9606 GN=EPB41 PE=1 SV=4 | EPB41 | 97.016 | 0.77 | 0.4126 | 1.03 | 0.8999 | 0.75 | 0.2143 |
| P52566 | Rho GDP-dissociation inhibitor 2 OS=Homo sapiens OX=9606 GN=ARHGDIB PE=1 SV=3 | ARHGDIB | 22.988 | 0.97 | 0.7542 | 0.89 | 0.5778 | 1.08 | 0.7870 |
| Q92796 | Disks large homolog 3 OS=Homo sapiens OX=9606 GN=DLG3 PE=1 SV=2 | DLG3 | 90.313 | 1.14 | 0.7202 | 0.74 | 0.1713 | 1.54 | 0.0505 |
| P07737 | Profilin-1 OS=Homo sapiens OX=9606 GN=PFN1 PE=1 SV=2 | PFN1 | 15.054 | 0.48 | 0.1392 | 1.07 | 0.7358 | 0.45 | 0.0015 |
| Q9H8S9 | MOB kinase activator 1A OS=Homo sapiens OX=9606 GN=MOB1A PE=1 SV=4 | MOB1A | 25.079 | 0.61 | 0.1300 | 0.65 | 0.4630 | 0.94 | 0.6801 |
| Q9NUL3 | Double-stranded RNA-binding protein Staufen homolog 2 OS=Homo sapiens OX=9606 GN=STAU2 PE=1 SV=2 | STAU2 | 62.608 | 1.00 | 0.9288 | 0.96 | 0.9999 | 1.04 | 0.9204 |
| A0A0C4DH73 | Immunoglobulin kappa variable 1-12 OS=Homo sapiens OX=9606 GN=IGKV1-12 PE=3 SV=1 | IGKV1-12 | 12.645 | 3.23 | 0.4429 | 3.40 | 0.4952 | 0.95 | 0.6278 |
| Q8N9W4 | Golgin subfamily A member 6-like protein 2 OS=Homo sapiens OX=9606 GN=GOLGA6L2 PE=1 SV=3 | GOLGA6L2 | 100.61 | 0.82 | 0.1857 | 0.96 | 0.8514 | 0.85 | 0.3557 |
| A0A0J9YXX1 | Immunoglobulin heavy variable 5-10-1 OS=Homo sapiens OX=9606 GN=IGHV5-10-1 PE=3 SV=1 | IGHV5-10-1 | 12.772 | 1.44 | 0.7964 | 0.88 | 0.3880 | 1.65 | 0.2096 |
| O14791 | Apolipoprotein L1 OS=Homo sapiens OX=9606 GN=APOL1 PE=1 SV=5 | APOL1 | 43.974 | 0.59 | 0.0015 | 0.96 | 0.9107 | 0.61 | 0.0023 |
| Q9BQ67 | Glutamate-rich WD repeat-containing protein 1 OS=Homo sapiens OX=9606 GN=GRWD1 PE=1 SV=1 | GRWD1 | 49.419 |  |  |  |  |  |  |
| Q9NR20 | Dual specificity tyrosine-phosphorylation-regulated kinase 4 OS=Homo sapiens OX=9606 GN=DYRK4 PE=1 SV=2 | DYRK4 | 59.608 | 0.52 | 0.0138 | 0.83 | 0.4393 | 0.62 | 0.0258 |
| O75131 | Copine-3 OS=Homo sapiens OX=9606 GN=CPNE3 PE=1 SV=1 | CPNE3 | 60.13 | 0.98 | 0.7210 | 0.70 | 0.1455 | 1.40 | 0.0634 |
| Q96NW4 | Ankyrin repeat domain-containing protein 27 OS=Homo sapiens OX=9606 GN=ANKRD27 PE=1 SV=2 | ANKRD27 | 116.98 |  |  |  |  |  |  |
| P27701 | CD82 antigen OS=Homo sapiens OX=9606 GN=CD82 PE=1 SV=1 | CD82 | 29.625 | 0.72 | 0.2238 | 0.64 | 0.1601 | 1.12 | 0.7675 |
| A0A0A0MS14 | Immunoglobulin heavy variable 1-45 OS=Homo sapiens OX=9606 GN=IGHV1-45 PE=3 SV=1 | IGHV1-45 | 13.508 | 0.87 | 0.3514 | 1.29 | 0.1152 | 0.67 | 0.0101 |
| P30085 | UMP-CMP kinase OS=Homo sapiens OX=9606 GN=CMPK1 PE=1 SV=3 | CMPK1 | 22.222 | 1.87 | 0.3664 | 1.19 | 0.7843 | 1.57 | 0.1597 |
| Q6ZS17 | Rho family-interacting cell polarization regulator 1 OS=Homo sapiens OX=9606 GN=RIPOR1 PE=1 SV=1 | RIPOR1 | 132.31 | 1.39 | 0.2744 | 0.88 | 0.3678 | 1.58 | 0.0097 |
| P34947 | G protein-coupled receptor kinase 5 OS=Homo sapiens OX=9606 GN=GRK5 PE=1 SV=1 | GRK5 | 67.786 | 1.30 | 0.2547 | 0.93 | 0.9635 | 1.41 | 0.3938 |
| P01834 | Immunoglobulin kappa constant OS=Homo sapiens OX=9606 GN=IGKC PE=1 SV=2 | IGKC | 11.765 | 0.70 | 0.0370 | 0.91 | 0.3455 | 0.77 | 0.0419 |
| Q9NP58 | ATP-binding cassette sub-family B member 6, mitochondrial OS=Homo sapiens OX=9606 GN=ABCB6 PE=1 SV=1 | ABCB6 | 93.884 | 0.62 |  | 0.99 | 0.9709 | 0.62 |  |
| Q569K6 | Coiled-coil domain-containing protein 157 OS=Homo sapiens OX=9606 GN=CCDC157 PE=2 SV=3 | CCDC157 | 83.94 | 0.79 | 0.5756 | 1.28 | 0.4233 | 0.62 | 0.1189 |
| Q08380 | Galectin-3-binding protein OS=Homo sapiens OX=9606 GN=LGALS3BP PE=1 SV=1 | LGALS3BP | 65.33 | 1.04 | 0.8789 | 1.07 | 0.5459 | 0.97 | 0.6459 |
| P28799 | Granulins OS=Homo sapiens OX=9606 GN=GRN PE=1 SV=2 | GRN | 63.544 | 0.86 | 0.1016 | 0.93 | 0.3426 | 0.93 | 0.3773 |
| Q9UK55 | Protein Z-dependent protease inhibitor OS=Homo sapiens OX=9606 GN=SERPINA10 PE=1 SV=1 | SERPINA10 | 50.706 | 1.04 | 0.5732 | 1.18 | 0.0412 | 0.88 | 0.3487 |
| Q29963 | HLA class I histocompatibility antigen, Cw-6 alpha chain OS=Homo sapiens OX=9606 GN=HLA-C PE=1 SV=2 | HLA-C | 40.968 |  |  |  |  |  |  |
| A0A0B4J1X5 | Immunoglobulin heavy variable 3-74 OS=Homo sapiens OX=9606 GN=IGHV3-74 PE=3 SV=1 | IGHV3-74 | 12.839 | 0.33 | 0.0197 | 0.84 | 0.1901 | 0.40 | 0.0363 |
| P62847 | 40S ribosomal protein S24 OS=Homo sapiens OX=9606 GN=RPS24 PE=1 SV=1 | RPS24 | 15.423 |  |  |  |  |  |  |
| P05164 | Myeloperoxidase OS=Homo sapiens OX=9606 GN=MPO PE=1 SV=1 | MPO | 83.868 | 1.30 | 0.8168 | 1.15 | 0.6760 | 1.13 | 0.8591 |
| Q03001 | Dystonin OS=Homo sapiens OX=9606 GN=DST PE=1 SV=4 | DST | 860.65 | 1.13 | 0.5983 | 1.03 | 0.8974 | 1.10 | 0.5463 |
| O95445 | Apolipoprotein M OS=Homo sapiens OX=9606 GN=APOM PE=1 SV=2 | APOM | 21.253 | 1.13 | 0.4459 | 1.07 | 0.5554 | 1.05 | 0.7356 |
| Q13418 | Integrin-linked protein kinase OS=Homo sapiens OX=9606 GN=ILK PE=1 SV=2 | ILK | 51.419 | 0.56 | 0.0229 | 0.83 | 0.3245 | 0.68 | 0.0448 |
| Q9H3D4 | Tumor protein 63 OS=Homo sapiens OX=9606 GN=TP63 PE=1 SV=1 | TP63 | 76.785 | 1.24 | 0.1435 | 1.06 | 0.6240 | 1.17 | 0.0841 |
| Q15428 | Splicing factor 3A subunit 2 OS=Homo sapiens OX=9606 GN=SF3A2 PE=1 SV=2 | SF3A2 | 49.255 | 1.40 | 0.4179 | 1.08 | 0.7753 | 1.30 | 0.4172 |
| P60660 | Myosin light polypeptide 6 OS=Homo sapiens OX=9606 GN=MYL6 PE=1 SV=2 | MYL6 | 16.93 | 0.47 | 0.0182 | 0.77 | 0.2463 | 0.62 | 0.0579 |
| P0DMV9 | Heat shock 70 kDa protein 1B OS=Homo sapiens OX=9606 GN=HSPA1B PE=1 SV=1 | HSPA1B | 70.051 | 1.14 | 0.3162 | 0.94 | 0.7318 | 1.21 | 0.1370 |
| Q04917 | 14-3-3 protein eta OS=Homo sapiens OX=9606 GN=YWHAH PE=1 SV=4 | YWHAH | 28.218 | 0.57 | 0.0258 | 0.80 | 0.3411 | 0.72 | 0.0862 |
| Q8TF66 | Leucine-rich repeat-containing protein 15 OS=Homo sapiens OX=9606 GN=LRRC15 PE=2 SV=2 | LRRC15 | 64.365 |  |  | 12.50 | 0.3763 |  |  |
| Q9BVC6 | Transmembrane protein 109 OS=Homo sapiens OX=9606 GN=TMEM109 PE=1 SV=1 | TMEM109 | 26.21 | 0.89 | 0.6120 | 0.73 | 0.3692 | 1.21 | 0.7298 |
| Q8IZ83 | Aldehyde dehydrogenase family 16 member A1 OS=Homo sapiens OX=9606 GN=ALDH16A1 PE=1 SV=2 | ALDH16A1 | 85.126 | 0.99 | 0.7931 | 0.89 | 0.2207 | 1.11 | 0.3201 |
| P12277 | Creatine kinase B-type OS=Homo sapiens OX=9606 GN=CKB PE=1 SV=1 | CKB | 42.644 | 1.04 | 0.8959 | 0.99 | 0.9995 | 1.05 | 0.8782 |
| P49747 | Cartilage oligomeric matrix protein OS=Homo sapiens OX=9606 GN=COMP PE=1 SV=2 | COMP | 82.86 | 1.38 | 0.2117 | 1.41 | 0.1731 | 0.98 | 0.7370 |
| Q9BXR5 | Toll-like receptor 10 OS=Homo sapiens OX=9606 GN=TLR10 PE=1 SV=2 | TLR10 | 94.563 | 1.44 | 0.5762 | 0.80 | 0.2973 | 1.79 | 0.0132 |
| P08670 | Vimentin OS=Homo sapiens OX=9606 GN=VIM PE=1 SV=4 | VIM | 53.651 | 1.14 | 0.8319 | 1.09 | 0.9380 | 1.05 | 0.6378 |
| O75558 | Syntaxin-11 OS=Homo sapiens OX=9606 GN=STX11 PE=1 SV=1 | STX11 | 33.195 | 0.51 | 0.0043 | 0.78 | 0.1983 | 0.65 | 0.0236 |
| P27169 | Serum paraoxonase/arylesterase 1 OS=Homo sapiens OX=9606 GN=PON1 PE=1 SV=3 | PON1 | 39.731 | 1.03 | 0.9994 | 1.02 | 0.9989 | 1.01 | 0.9979 |
| Q14161 | ARF GTPase-activating protein GIT2 OS=Homo sapiens OX=9606 GN=GIT2 PE=1 SV=2 | GIT2 | 84.542 | 2.57 | 0.4152 | 2.22 | 0.2019 | 1.16 | 0.7793 |
| A0A0C4DH38 | Immunoglobulin heavy variable 5-51 OS=Homo sapiens OX=9606 GN=IGHV5-51 PE=3 SV=1 | IGHV5-51 | 12.674 | 0.97 | 0.7086 | 0.86 | 0.3194 | 1.13 | 0.5005 |
| Q13530 | Serine incorporator 3 OS=Homo sapiens OX=9606 GN=SERINC3 PE=2 SV=2 | SERINC3 | 52.58 | 1.08 | 0.7265 | 0.79 | 0.4066 | 1.37 | 0.3166 |
| Q06547 | GA-binding protein subunit beta-1 OS=Homo sapiens OX=9606 GN=GABPB1 PE=1 SV=2 | GABPB1 | 42.482 |  |  |  |  |  |  |
| Q8WUA8 | Tsukushin OS=Homo sapiens OX=9606 GN=TSKU PE=2 SV=3 | TSKU | 37.807 | 1.48 | 0.1811 | 1.27 | 0.1760 | 1.17 | 0.4235 |
| O75382 | Tripartite motif-containing protein 3 OS=Homo sapiens OX=9606 GN=TRIM3 PE=1 SV=2 | TRIM3 | 80.829 | 1.24 | 0.4243 | 1.18 | 0.2910 | 1.05 | 0.7480 |
| Q4KMQ2 | Anoctamin-6 OS=Homo sapiens OX=9606 GN=ANO6 PE=1 SV=2 | ANO6 | 106.16 | 0.87 | 0.3291 | 0.81 | 0.3398 | 1.07 | 0.9390 |
| Q5SZL2 | Centrosomal protein of 85 kDa-like OS=Homo sapiens OX=9606 GN=CEP85L PE=1 SV=1 | CEP85L | 91.807 | 0.76 | 0.2137 | 0.89 | 0.5437 | 0.85 | 0.3515 |
| P01137 | Transforming growth factor beta-1 OS=Homo sapiens OX=9606 GN=TGFB1 PE=1 SV=2 | TGFB1 | 44.341 | 1.17 | 0.1718 | 1.03 | 0.6771 | 1.13 | 0.1453 |
| P61224 | Ras-related protein Rap-1b OS=Homo sapiens OX=9606 GN=RAP1B PE=1 SV=1 | RAP1B | 20.825 | 0.61 | 0.0244 | 0.80 | 0.2667 | 0.77 | 0.1046 |
| P41214 | Eukaryotic translation initiation factor 2D OS=Homo sapiens OX=9606 GN=EIF2D PE=1 SV=3 | EIF2D | 64.706 | 1.41 | 0.1866 | 1.15 | 0.3428 | 1.23 | 0.1394 |
| Q8NI99 | Angiopoietin-related protein 6 OS=Homo sapiens OX=9606 GN=ANGPTL6 PE=1 SV=1 | ANGPTL6 | 51.694 | 1.27 | 0.0935 | 1.02 | 0.8537 | 1.24 | 0.0147 |
| Q8N612 | FTS and Hook-interacting protein OS=Homo sapiens OX=9606 GN=FAM160A2 PE=1 SV=3 | FAM160A2 | 105.57 | 1.61 | 0.1679 | 1.33 | 0.2120 | 1.21 | 0.9503 |
| P60900 | Proteasome subunit alpha type-6 OS=Homo sapiens OX=9606 GN=PSMA6 PE=1 SV=1 | PSMA6 | 27.399 | 1.02 | 0.8218 | 0.94 | 0.7895 | 1.09 | 0.6569 |
| P15090 | Fatty acid-binding protein, adipocyte OS=Homo sapiens OX=9606 GN=FABP4 PE=1 SV=3 | FABP4 | 14.719 | 0.92 | 0.6047 | 0.72 | 0.1345 | 1.28 | 0.5357 |
| P14174 | Macrophage migration inhibitory factor OS=Homo sapiens OX=9606 GN=MIF PE=1 SV=4 | MIF | 12.476 |  |  |  |  |  |  |
| Q9NRW1 | Ras-related protein Rab-6B OS=Homo sapiens OX=9606 GN=RAB6B PE=1 SV=1 | RAB6B | 23.461 | 0.73 | 0.0301 | 0.77 | 0.1918 | 0.94 | 0.2840 |
| P02776 | Platelet factor 4 OS=Homo sapiens OX=9606 GN=PF4 PE=1 SV=2 | PF4 | 10.845 | 0.32 | 0.0098 | 0.60 | 0.5516 | 0.54 | 0.1411 |
| P51809 | Vesicle-associated membrane protein 7 OS=Homo sapiens OX=9606 GN=VAMP7 PE=1 SV=3 | VAMP7 | 24.935 | 1.10 | 0.5895 | 0.84 | 0.7613 | 1.31 | 0.5507 |
| P07585 | Decorin OS=Homo sapiens OX=9606 GN=DCN PE=1 SV=1 | DCN | 39.746 |  |  |  |  |  |  |
| P30498 | HLA class I histocompatibility antigen, B-78 alpha chain OS=Homo sapiens OX=9606 GN=HLA-B PE=1 SV=1 | HLA-B | 40.478 |  |  |  |  |  |  |
| Q9HC84 | Mucin-5B OS=Homo sapiens OX=9606 GN=MUC5B PE=1 SV=3 | MUC5B | 596.33 | 0.81 | 0.2899 | 1.02 | 0.8304 | 0.80 | 0.0328 |
| P61088 | Ubiquitin-conjugating enzyme E2 N OS=Homo sapiens OX=9606 GN=UBE2N PE=1 SV=1 | UBE2N | 17.138 | 1.03 | 0.9017 | 0.76 | 0.1912 | 1.36 | 0.0251 |
| Q9H0W8 | Protein SMG9 OS=Homo sapiens OX=9606 GN=SMG9 PE=1 SV=1 | SMG9 | 57.65 |  |  |  |  |  |  |
| P00488 | Coagulation factor XIII A chain OS=Homo sapiens OX=9606 GN=F13A1 PE=1 SV=4 | F13A1 | 83.266 | 1.44 | 0.0070 | 0.85 | 0.5726 | 1.69 | 0.0010 |
| P30626 | Sorcin OS=Homo sapiens OX=9606 GN=SRI PE=1 SV=1 | SRI | 21.676 | 1.02 | 0.9603 | 0.90 | 0.6591 | 1.13 | 0.6978 |
| Q5T0Z8 | Uncharacterized protein C6orf132 OS=Homo sapiens OX=9606 GN=C6orf132 PE=1 SV=4 | C6orf132 | 124.03 | 1.48 | 0.0013 | 1.83 | 0.0027 | 0.81 | 0.2909 |
| P14151 | L-selectin OS=Homo sapiens OX=9606 GN=SELL PE=1 SV=2 | SELL | 42.187 | 1.13 | 0.4488 | 1.12 | 0.3356 | 1.01 | 0.9167 |
| Q6ZS10 | C-type lectin domain family 17, member A OS=Homo sapiens OX=9606 GN=CLEC17A PE=1 SV=2 | CLEC17A | 42.935 | 1.60 | 0.2631 | 0.97 | 0.9360 | 1.65 | 0.2479 |
| P00740 | Coagulation factor IX OS=Homo sapiens OX=9606 GN=F9 PE=1 SV=2 | F9 | 51.778 | 1.14 | 0.3085 | 1.02 | 0.8774 | 1.12 | 0.2510 |
| A0A0B4J1Y8 | Immunoglobulin lambda variable 9-49 OS=Homo sapiens OX=9606 GN=IGLV9-49 PE=1 SV=1 | IGLV9-49 | 13.024 | 0.96 | 0.9788 | 1.37 | 0.2913 | 0.70 | 0.3676 |
| O14745 | Na(+)/H(+) exchange regulatory cofactor NHE-RF1 OS=Homo sapiens OX=9606 GN=SLC9A3R1 PE=1 SV=4 | SLC9A3R1 | 38.868 | 0.99 | 0.9811 | 0.83 | 0.3113 | 1.19 | 0.3837 |
| P62993 | Growth factor receptor-bound protein 2 OS=Homo sapiens OX=9606 GN=GRB2 PE=1 SV=1 | GRB2 | 25.206 | 0.86 | 0.2362 | 0.84 | 0.3654 | 1.02 | 0.8905 |
| P16070 | CD44 antigen OS=Homo sapiens OX=9606 GN=CD44 PE=1 SV=3 | CD44 | 81.537 | 1.27 | 0.2219 | 1.16 | 0.1703 | 1.09 | 0.4680 |
| Q00013 | 55 kDa erythrocyte membrane protein OS=Homo sapiens OX=9606 GN=MPP1 PE=1 SV=2 | MPP1 | 52.296 |  |  |  |  |  |  |
| Q7Z478 | ATP-dependent RNA helicase DHX29 OS=Homo sapiens OX=9606 GN=DHX29 PE=1 SV=2 | DHX29 | 155.23 | 1.26 | 0.8258 | 1.15 | 0.9448 | 1.10 | 0.6187 |
| P20073 | Annexin A7 OS=Homo sapiens OX=9606 GN=ANXA7 PE=1 SV=3 | ANXA7 | 52.739 | 1.00 | 0.9268 | 0.71 | 0.2343 | 1.41 | 0.2907 |
| P27918 | Properdin OS=Homo sapiens OX=9606 GN=CFP PE=1 SV=2 | CFP | 51.276 | 1.15 | 0.2380 | 0.99 | 0.9880 | 1.16 | 0.2259 |
| Q712K3 | Ubiquitin-conjugating enzyme E2 R2 OS=Homo sapiens OX=9606 GN=UBE2R2 PE=1 SV=1 | UBE2R2 | 27.166 |  |  |  |  |  |  |
| Q9NZN3 | EH domain-containing protein 3 OS=Homo sapiens OX=9606 GN=EHD3 PE=1 SV=2 | EHD3 | 60.886 | 0.65 | 0.0795 | 0.88 | 0.6353 | 0.74 | 0.1394 |
| O00429 | Dynamin-1-like protein OS=Homo sapiens OX=9606 GN=DNM1L PE=1 SV=2 | DNM1L | 81.876 | 0.59 | 0.2301 | 0.55 | 0.1659 | 1.07 | 0.9480 |
| Q9Y6Z7 | Collectin-10 OS=Homo sapiens OX=9606 GN=COLEC10 PE=1 SV=2 | COLEC10 | 30.705 | 1.11 | 0.6715 | 1.01 | 0.9844 | 1.10 | 0.6270 |
| Q6IBS0 | Twinfilin-2 OS=Homo sapiens OX=9606 GN=TWF2 PE=1 SV=2 | TWF2 | 39.548 | 0.69 | 0.1508 | 0.84 | 0.3095 | 0.82 | 0.1624 |
| Q5VZ89 | DENN domain-containing protein 4C OS=Homo sapiens OX=9606 GN=DENND4C PE=1 SV=3 | DENND4C | 212.71 | 5.40 | 0.0088 | 2.32 | 0.0553 | 2.33 | 0.0994 |
| Q15599 | Na(+)/H(+) exchange regulatory cofactor NHE-RF2 OS=Homo sapiens OX=9606 GN=SLC9A3R2 PE=1 SV=2 | SLC9A3R2 | 37.413 | 1.32 | 0.4854 | 0.88 | 0.5251 | 1.50 | 0.0954 |
| P68363 | Tubulin alpha-1B chain OS=Homo sapiens OX=9606 GN=TUBA1B PE=1 SV=1 | TUBA1B | 50.151 | 0.52 | 0.1792 | 0.83 | 0.8716 | 0.63 | 0.2802 |
| Q9Y448 | Small kinetochore-associated protein OS=Homo sapiens OX=9606 GN=KNSTRN PE=1 SV=2 | KNSTRN | 35.438 | 0.99 | 0.9212 | 0.80 | 0.3570 | 1.25 | 0.3714 |
| Q9Y6M0 | Testisin OS=Homo sapiens OX=9606 GN=PRSS21 PE=1 SV=1 | PRSS21 | 34.884 | 1.78 | 0.0665 | 1.33 | 0.1946 | 1.34 | 0.3185 |
| Q9Y251 | Heparanase OS=Homo sapiens OX=9606 GN=HPSE PE=1 SV=2 | HPSE | 61.148 | 0.94 | 0.7193 | 0.90 | 0.5490 | 1.04 | 0.9695 |
| A1L4H1 | Soluble scavenger receptor cysteine-rich domain-containing protein SSC5D OS=Homo sapiens OX=9606 GN=SSC5D PE=1 SV=3 | SSC5D | 165.74 | 1.55 | 0.4189 | 1.29 | 0.5905 | 1.20 | 0.5626 |
| O75083 | WD repeat-containing protein 1 OS=Homo sapiens OX=9606 GN=WDR1 PE=1 SV=4 | WDR1 | 66.193 | 0.52 | 0.0024 | 0.76 | 0.1814 | 0.68 | 0.0211 |
| P06733 | Alpha-enolase OS=Homo sapiens OX=9606 GN=ENO1 PE=1 SV=2 | ENO1 | 47.168 | 0.67 | 0.0167 | 0.84 | 0.2682 | 0.80 | 0.0888 |
| P01591 | Immunoglobulin J chain OS=Homo sapiens OX=9606 GN=JCHAIN PE=1 SV=4 | JCHAIN | 18.098 | 0.79 | 0.0384 | 0.91 | 0.1560 | 0.87 | 0.1030 |
| P04070 | Vitamin K-dependent protein C OS=Homo sapiens OX=9606 GN=PROC PE=1 SV=1 | PROC | 52.071 | 1.11 | 0.3620 | 0.90 | 0.2978 | 1.23 | 0.0517 |
| Q6YHU6 | Thyroid adenoma-associated protein OS=Homo sapiens OX=9606 GN=THADA PE=1 SV=1 | THADA | 219.6 |  |  |  |  |  |  |
| Q8IYT4 | Katanin p60 ATPase-containing subunit A-like 2 OS=Homo sapiens OX=9606 GN=KATNAL2 PE=1 SV=3 | KATNAL2 | 61.252 | 0.55 | 0.0088 | 0.63 | 0.0022 | 0.87 | 0.3021 |
| P11169 | Solute carrier family 2, facilitated glucose transporter member 3 OS=Homo sapiens OX=9606 GN=SLC2A3 PE=1 SV=1 | SLC2A3 | 53.924 | 0.71 | 0.0932 | 0.82 | 0.3830 | 0.86 | 0.3502 |
| P00338 | L-lactate dehydrogenase A chain OS=Homo sapiens OX=9606 GN=LDHA PE=1 SV=2 | LDHA | 36.688 | 0.62 | 0.0224 | 0.80 | 0.1487 | 0.77 | 0.1040 |
| O60832 | H/ACA ribonucleoprotein complex subunit DKC1 OS=Homo sapiens OX=9606 GN=DKC1 PE=1 SV=3 | DKC1 | 57.673 | 0.84 | 0.3971 | 1.18 | 0.3385 | 0.71 | 0.0460 |
| P35247 | Pulmonary surfactant-associated protein D OS=Homo sapiens OX=9606 GN=SFTPD PE=1 SV=3 | SFTPD | 37.728 |  |  |  |  |  |  |
| O60508 | Pre-mRNA-processing factor 17 OS=Homo sapiens OX=9606 GN=CDC40 PE=1 SV=1 | CDC40 | 65.521 | 1.92 | 0.0181 | 0.96 | 0.9485 | 1.99 | 0.0163 |
| Q9Y4P3 | Transducin beta-like protein 2 OS=Homo sapiens OX=9606 GN=TBL2 PE=1 SV=1 | TBL2 | 49.797 | 0.53 | 0.3129 | 1.11 | 0.7078 | 0.48 | 0.0184 |
| Q5T7N8 | Protein FAM27D1 OS=Homo sapiens OX=9606 GN=FAM27D1 PE=3 SV=2 | FAM27D1 | 24.905 | 4.23 | 0.3089 | 0.73 | 0.8963 | 5.78 | 0.2031 |
| P08962 | CD63 antigen OS=Homo sapiens OX=9606 GN=CD63 PE=1 SV=2 | CD63 | 25.636 | 0.83 | 0.3032 | 0.80 | 0.5064 | 1.04 | 0.8346 |
| Q92954 | Proteoglycan 4 OS=Homo sapiens OX=9606 GN=PRG4 PE=1 SV=3 | PRG4 | 151.06 | 1.42 | 0.2468 | 1.09 | 0.7169 | 1.30 | 0.2268 |
| P01344 | Insulin-like growth factor II OS=Homo sapiens OX=9606 GN=IGF2 PE=1 SV=1 | IGF2 | 20.14 | 1.15 | 0.7779 | 0.79 | 0.5971 | 1.47 | 0.4430 |
| P02787 | Serotransferrin OS=Homo sapiens OX=9606 GN=TF PE=1 SV=3 | TF | 77.063 | 1.00 | 0.9716 | 0.83 | 0.2388 | 1.21 | 0.2549 |
| O43639 | Cytoplasmic protein NCK2 OS=Homo sapiens OX=9606 GN=NCK2 PE=1 SV=2 | NCK2 | 42.915 | 0.51 | 0.1794 | 0.60 | 0.1866 | 0.85 | 0.6050 |
| P35858 | Insulin-like growth factor-binding protein complex acid labile subunit OS=Homo sapiens OX=9606 GN=IGFALS PE=1 SV=1 | IGFALS | 66.034 | 1.13 | 0.1435 | 0.90 | 0.3868 | 1.26 | 0.1236 |
| P06702 | Protein S100-A9 OS=Homo sapiens OX=9606 GN=S100A9 PE=1 SV=1 | S100A9 | 13.242 | 0.63 | 0.1812 | 1.17 | 0.4271 | 0.54 | 0.0152 |
| P10909 | Clusterin OS=Homo sapiens OX=9606 GN=CLU PE=1 SV=1 | CLU | 52.494 | 1.51 | 0.0060 | 0.89 | 0.5221 | 1.69 | 0.0000 |
| Q9H4K1 | RIB43A-like with coiled-coils protein 2 OS=Homo sapiens OX=9606 GN=RIBC2 PE=1 SV=1 | RIBC2 | 37.059 | 2.09 | 0.2679 | 1.28 | 0.5385 | 1.63 | 0.1345 |
| P07602 | Prosaposin OS=Homo sapiens OX=9606 GN=PSAP PE=1 SV=2 | PSAP | 58.112 | 0.84 | 0.2940 | 0.78 | 0.2766 | 1.08 | 0.9419 |
| Q12805 | EGF-containing fibulin-like extracellular matrix protein 1 OS=Homo sapiens OX=9606 GN=EFEMP1 PE=1 SV=2 | EFEMP1 | 54.64 | 1.34 | 0.2503 | 0.77 | 0.1356 | 1.73 | 0.0138 |
| P07355 | Annexin A2 OS=Homo sapiens OX=9606 GN=ANXA2 PE=1 SV=2 | ANXA2 | 38.604 | 1.09 | 0.5927 | 0.92 | 0.3492 | 1.18 | 0.0843 |
| P23284 | Peptidyl-prolyl cis-trans isomerase B OS=Homo sapiens OX=9606 GN=PPIB PE=1 SV=2 | PPIB | 23.742 | 0.74 | 0.1598 | 0.81 | 0.3082 | 0.91 | 0.4663 |
| O14818 | Proteasome subunit alpha type-7 OS=Homo sapiens OX=9606 GN=PSMA7 PE=1 SV=1 | PSMA7 | 27.887 | 1.21 | 0.1887 | 1.05 | 0.4986 | 1.15 | 0.5393 |
| Q8NC51 | Plasminogen activator inhibitor 1 RNA-binding protein OS=Homo sapiens OX=9606 GN=SERBP1 PE=1 SV=2 | SERBP1 | 44.965 |  |  |  |  |  |  |
| Q7Z3D4 | LysM and putative peptidoglycan-binding domain-containing protein 3 OS=Homo sapiens OX=9606 GN=LYSMD3 PE=1 SV=2 | LYSMD3 | 34.537 | 0.41 | 0.0784 | 0.69 | 0.2836 | 0.58 | 0.1705 |
| P84074 | Neuron-specific calcium-binding protein hippocalcin OS=Homo sapiens OX=9606 GN=HPCA PE=1 SV=2 | HPCA | 22.427 | 1.47 | 0.2973 | 1.02 | 0.9548 | 1.44 | 0.2392 |
| P55058 | Phospholipid transfer protein OS=Homo sapiens OX=9606 GN=PLTP PE=1 SV=1 | PLTP | 54.739 | 1.01 | 0.8726 | 1.01 | 0.7841 | 1.00 | 0.9196 |
| P00747 | Plasminogen OS=Homo sapiens OX=9606 GN=PLG PE=1 SV=2 | PLG | 90.568 | 0.91 | 0.5307 | 1.08 | 0.5846 | 0.84 | 0.2380 |
| Q14314 | Fibroleukin OS=Homo sapiens OX=9606 GN=FGL2 PE=1 SV=1 | FGL2 | 50.228 | 0.95 | 0.5317 | 0.86 | 0.3208 | 1.10 | 0.6343 |
| P46952 | 3-hydroxyanthranilate 3,4-dioxygenase OS=Homo sapiens OX=9606 GN=HAAO PE=1 SV=2 | HAAO | 32.556 | 2.41 | 0.0077 | 1.13 | 0.5780 | 2.13 | 0.2288 |
| Q9Y613 | FH1/FH2 domain-containing protein 1 OS=Homo sapiens OX=9606 GN=FHOD1 PE=1 SV=3 | FHOD1 | 126.55 | 0.76 | 0.1333 | 0.71 | 0.1557 | 1.08 | 0.9778 |
| A6NGU5 | Putative glutathione hydrolase 3 proenzyme OS=Homo sapiens OX=9606 GN=GGT3P PE=5 SV=2 | GGT3P | 61.501 | 1.49 | 0.1840 | 1.18 | 0.4652 | 1.26 | 0.2960 |
| O15381 | Nuclear valosin-containing protein-like OS=Homo sapiens OX=9606 GN=NVL PE=1 SV=1 | NVL | 95.05 | 0.63 | 0.0702 | 0.61 | 0.3292 | 1.02 | 0.8543 |
| P13716 | Delta-aminolevulinic acid dehydratase OS=Homo sapiens OX=9606 GN=ALAD PE=1 SV=1 | ALAD | 36.294 | 1.01 | 0.9456 | 0.67 | 0.3619 | 1.51 | 0.4635 |
| P0DP03 | Immunoglobulin heavy variable 3-30-5 OS=Homo sapiens OX=9606 GN=IGHV3-30-5 PE=3 SV=1 | IGHV3-30-5 | 12.947 | 0.74 | 0.0934 | 0.84 | 0.5140 | 0.88 | 0.4462 |
| A0A075B6I9 | Immunoglobulin lambda variable 7-46 OS=Homo sapiens OX=9606 GN=IGLV7-46 PE=3 SV=4 | IGLV7-46 | 12.468 | 0.76 | 0.1136 | 0.78 | 0.0633 | 0.97 | 0.8479 |
| P52209 | 6-phosphogluconate dehydrogenase, decarboxylating OS=Homo sapiens OX=9606 GN=PGD PE=1 SV=3 | PGD | 53.139 | 0.55 | 0.1036 | 0.76 | 0.1666 | 0.71 | 0.0528 |
| O15265 | Ataxin-7 OS=Homo sapiens OX=9606 GN=ATXN7 PE=1 SV=1 | ATXN7 | 95.45 | 0.90 | 0.2529 | 0.36 | 0.1804 | 2.53 | 0.4553 |
| O00461 | Golgi integral membrane protein 4 OS=Homo sapiens OX=9606 GN=GOLIM4 PE=1 SV=1 | GOLIM4 | 81.879 |  |  |  |  |  |  |
| P22792 | Carboxypeptidase N subunit 2 OS=Homo sapiens OX=9606 GN=CPN2 PE=1 SV=3 | CPN2 | 60.556 | 1.14 | 0.1646 | 1.11 | 0.1666 | 1.03 | 0.7762 |
| Q8TBC5 | Zinc finger and SCAN domain-containing protein 18 OS=Homo sapiens OX=9606 GN=ZSCAN18 PE=2 SV=2 | ZSCAN18 | 54.803 | 1.07 | 0.7433 | 0.64 | 0.0346 | 1.66 | 0.0306 |
| O43242 | 26S proteasome non-ATPase regulatory subunit 3 OS=Homo sapiens OX=9606 GN=PSMD3 PE=1 SV=2 | PSMD3 | 60.977 | 0.68 | 0.6060 | 0.93 | 0.5443 | 0.73 | 0.7203 |
| Q9HCU9 | Breast cancer metastasis-suppressor 1 OS=Homo sapiens OX=9606 GN=BRMS1 PE=1 SV=1 | BRMS1 | 28.46 | 1.34 | 0.0392 | 0.49 | 0.6351 | 2.75 | 0.3735 |
| Q6P435 | Putative uncharacterized SMG1-like protein OS=Homo sapiens OX=9606 PE=5 SV=1 | --- | 17.652 | 0.56 | 0.0116 | 0.79 | 0.5974 | 0.70 | 0.2572 |
| O75143 | Autophagy-related protein 13 OS=Homo sapiens OX=9606 GN=ATG13 PE=1 SV=1 | ATG13 | 56.571 |  |  |  |  |  |  |
| P11047 | Laminin subunit gamma-1 OS=Homo sapiens OX=9606 GN=LAMC1 PE=1 SV=3 | LAMC1 | 177.6 | 1.73 | 0.2299 | 1.21 | 0.3220 | 1.43 | 0.0068 |
| O95670 | V-type proton ATPase subunit G 2 OS=Homo sapiens OX=9606 GN=ATP6V1G2 PE=1 SV=1 | ATP6V1G2 | 13.604 |  |  |  |  |  |  |
| O60293 | Zinc finger C3H1 domain-containing protein OS=Homo sapiens OX=9606 GN=ZFC3H1 PE=1 SV=3 | ZFC3H1 | 226.35 | 1.31 | 0.2334 | 0.96 | 0.9155 | 1.37 | 0.4367 |
| Q03591 | Complement factor H-related protein 1 OS=Homo sapiens OX=9606 GN=CFHR1 PE=1 SV=2 | CFHR1 | 37.65 | 1.34 | 0.2930 | 1.06 | 0.5579 | 1.26 | 0.7797 |
| Q9H6Z4 | Ran-binding protein 3 OS=Homo sapiens OX=9606 GN=RANBP3 PE=1 SV=1 | RANBP3 | 60.209 | 0.90 | 0.4370 | 0.90 | 0.3325 | 0.99 | 0.9537 |
| Q969X1 | Protein lifeguard 3 OS=Homo sapiens OX=9606 GN=TMBIM1 PE=1 SV=2 | TMBIM1 | 34.607 | 1.09 | 0.7024 | 0.82 | 0.4692 | 1.32 | 0.1203 |
| O75954 | Tetraspanin-9 OS=Homo sapiens OX=9606 GN=TSPAN9 PE=1 SV=1 | TSPAN9 | 26.779 | 0.66 | 0.0234 | 0.77 | 0.2643 | 0.86 | 0.3046 |
| A0A0B4J1V2 | Immunoglobulin heavy variable 2-26 OS=Homo sapiens OX=9606 GN=IGHV2-26 PE=3 SV=1 | IGHV2-26 | 13.182 | 0.83 | 0.5189 | 1.06 | 0.7421 | 0.78 | 0.4030 |
| Q14624 | Inter-alpha-trypsin inhibitor heavy chain H4 OS=Homo sapiens OX=9606 GN=ITIH4 PE=1 SV=4 | ITIH4 | 103.36 | 0.89 | 0.4067 | 0.94 | 0.6239 | 0.95 | 0.6193 |
| O15439 | Multidrug resistance-associated protein 4 OS=Homo sapiens OX=9606 GN=ABCC4 PE=1 SV=3 | ABCC4 | 149.52 | 0.57 | 0.3949 | 0.66 | 0.1602 | 0.86 | 0.6644 |
| Q495T6 | Membrane metallo-endopeptidase-like 1 OS=Homo sapiens OX=9606 GN=MMEL1 PE=2 SV=2 | MMEL1 | 89.366 | 3.27 | 0.2986 | 1.06 | 0.9685 | 3.10 | 0.1166 |
| P22694 | cAMP-dependent protein kinase catalytic subunit beta OS=Homo sapiens OX=9606 GN=PRKACB PE=1 SV=2 | PRKACB | 40.622 | 0.79 | 0.3834 | 0.67 | 0.4463 | 1.19 | 0.9469 |
| O95810 | Caveolae-associated protein 2 OS=Homo sapiens OX=9606 GN=CAVIN2 PE=1 SV=3 | CAVIN2 | 47.173 | 0.64 | 0.0108 | 0.68 | 0.0710 | 0.95 | 0.4972 |
| Q8IY82 | Dynein regulatory complex subunit 7 OS=Homo sapiens OX=9606 GN=DRC7 PE=1 SV=3 | DRC7 | 103.5 | 0.16 | 0.5936 | 1.05 | 0.9109 | 0.16 | 0.6104 |
| P80188 | Neutrophil gelatinase-associated lipocalin OS=Homo sapiens OX=9606 GN=LCN2 PE=1 SV=2 | LCN2 | 22.588 | 0.89 | 0.6984 | 1.17 | 0.9135 | 0.76 | 0.0721 |
| Q9NQC3 | Reticulon-4 OS=Homo sapiens OX=9606 GN=RTN4 PE=1 SV=2 | RTN4 | 129.93 | 0.73 | 0.0357 | 0.77 | 0.1260 | 0.95 | 0.2878 |
| P02679 | Fibrinogen gamma chain OS=Homo sapiens OX=9606 GN=FGG PE=1 SV=3 | FGG | 51.511 | 2.78 | 0.0001 | 1.54 | 0.0149 | 1.80 | 0.0006 |
| P05556 | Integrin beta-1 OS=Homo sapiens OX=9606 GN=ITGB1 PE=1 SV=2 | ITGB1 | 88.414 | 0.66 | 0.0364 | 0.80 | 0.2458 | 0.83 | 0.2188 |
| O15144 | Actin-related protein 2/3 complex subunit 2 OS=Homo sapiens OX=9606 GN=ARPC2 PE=1 SV=1 | ARPC2 | 34.333 | 0.51 | 0.0222 | 0.73 | 0.1823 | 0.70 | 0.0910 |
| Q96QZ7 | Membrane-associated guanylate kinase, WW and PDZ domain-containing protein 1 OS=Homo sapiens OX=9606 GN=MAGI1 PE=1 SV=3 | MAGI1 | 164.58 | 0.43 | 0.0971 | 0.64 | 0.0172 | 0.66 | 0.0180 |
| O43149 | Zinc finger ZZ-type and EF-hand domain-containing protein 1 OS=Homo sapiens OX=9606 GN=ZZEF1 PE=1 SV=6 | ZZEF1 | 331.07 | 0.53 | 0.0179 | 0.94 | 0.7690 | 0.56 | 0.0110 |
| Q9Y6D5 | Brefeldin A-inhibited guanine nucleotide-exchange protein 2 OS=Homo sapiens OX=9606 GN=ARFGEF2 PE=1 SV=3 | ARFGEF2 | 202.04 | 1.71 | 0.2836 | 1.05 | 0.8179 | 1.63 | 0.0353 |
| P00709 | Alpha-lactalbumin OS=Homo sapiens OX=9606 GN=LALBA PE=1 SV=1 | LALBA | 16.225 | 0.71 | 0.2516 | 0.83 | 0.9772 | 0.86 | 0.3154 |
| Q6YHK3 | CD109 antigen OS=Homo sapiens OX=9606 GN=CD109 PE=1 SV=2 | CD109 | 161.69 | 0.73 | 0.2951 | 0.57 | 0.0205 | 1.28 | 0.1518 |
| Q9BYK8 | Helicase with zinc finger domain 2 OS=Homo sapiens OX=9606 GN=HELZ2 PE=1 SV=6 | HELZ2 | 294.65 | 0.72 | 0.5564 | 0.97 | 0.9175 | 0.75 | 0.5014 |
| Q2KJY2 | Kinesin-like protein KIF26B OS=Homo sapiens OX=9606 GN=KIF26B PE=2 SV=1 | KIF26B | 223.88 | 0.44 | 0.0858 | 0.74 | 0.5337 | 0.60 | 0.1981 |
| Q9P2E9 | Ribosome-binding protein 1 OS=Homo sapiens OX=9606 GN=RRBP1 PE=1 SV=5 | RRBP1 | 152.45 | 1.02 | 0.8778 | 0.62 | 0.2921 | 1.64 | 0.2914 |
| P35613 | Basigin OS=Homo sapiens OX=9606 GN=BSG PE=1 SV=2 | BSG | 42.2 | 1.18 | 0.2125 | 1.07 | 0.5350 | 1.10 | 0.6356 |
| P02763 | Alpha-1-acid glycoprotein 1 OS=Homo sapiens OX=9606 GN=ORM1 PE=1 SV=1 | ORM1 | 23.511 | 1.03 | 0.8081 | 1.21 | 0.1310 | 0.85 | 0.2799 |
| P01861 | Immunoglobulin heavy constant gamma 4 OS=Homo sapiens OX=9606 GN=IGHG4 PE=1 SV=1 | IGHG4 | 35.94 | 0.82 | 0.2270 | 0.76 | 0.2678 | 1.08 | 0.9960 |
| P30405 | Peptidyl-prolyl cis-trans isomerase F, mitochondrial OS=Homo sapiens OX=9606 GN=PPIF PE=1 SV=1 | PPIF | 22.04 | 0.52 | 0.0899 | 0.77 | 0.2467 | 0.67 | 0.0917 |
| Q08495 | Dematin OS=Homo sapiens OX=9606 GN=DMTN PE=1 SV=3 | DMTN | 45.514 | 1.32 | 0.6035 | 1.54 | 0.2460 | 0.86 | 0.8684 |
| Q96HR3 | Mediator of RNA polymerase II transcription subunit 30 OS=Homo sapiens OX=9606 GN=MED30 PE=1 SV=1 | MED30 | 20.277 | 0.82 |  | 0.60 |  | 1.36 | 0.4681 |
| Q9H4I2 | Zinc fingers and homeoboxes protein 3 OS=Homo sapiens OX=9606 GN=ZHX3 PE=1 SV=3 | ZHX3 | 104.66 | 1.16 | 0.5908 | 1.08 | 0.6543 | 1.07 | 0.7069 |
| P19971 | Thymidine phosphorylase OS=Homo sapiens OX=9606 GN=TYMP PE=1 SV=2 | TYMP | 49.955 | 1.08 | 0.7931 | 0.74 | 0.1896 | 1.46 | 0.1576 |
| P62328 | Thymosin beta-4 OS=Homo sapiens OX=9606 GN=TMSB4X PE=1 SV=2 | TMSB4X | 5.0526 | 0.50 | 0.0912 | 0.86 | 0.7378 | 0.59 | 0.0953 |
| P09543 | 2',3'-cyclic-nucleotide 3'-phosphodiesterase OS=Homo sapiens OX=9606 GN=CNP PE=1 SV=2 | CNP | 47.578 | 0.69 | 0.2512 | 1.00 | 0.9403 | 0.70 | 0.0350 |
| O00231 | 26S proteasome non-ATPase regulatory subunit 11 OS=Homo sapiens OX=9606 GN=PSMD11 PE=1 SV=3 | PSMD11 | 47.463 | 0.31 | 0.0174 | 0.74 | 0.4559 | 0.43 | 0.0244 |
| P47756 | F-actin-capping protein subunit beta OS=Homo sapiens OX=9606 GN=CAPZB PE=1 SV=4 | CAPZB | 31.35 | 0.79 | 0.0661 | 0.78 | 0.1387 | 1.02 | 0.9222 |
| Q14940 | Sodium/hydrogen exchanger 5 OS=Homo sapiens OX=9606 GN=SLC9A5 PE=1 SV=2 | SLC9A5 | 99.01 | 12.75 | 0.3636 | 5.51 | 0.6119 | 2.32 | 0.1578 |
| P59768 | Guanine nucleotide-binding protein G(I)/G(S)/G(O) subunit gamma-2 OS=Homo sapiens OX=9606 GN=GNG2 PE=1 SV=2 | GNG2 | 7.8501 | 1.04 | 0.9618 | 1.09 | 0.7146 | 0.95 | 0.7334 |
| P01031 | Complement C5 OS=Homo sapiens OX=9606 GN=C5 PE=1 SV=4 | C5 | 188.3 | 0.98 | 0.7346 | 1.39 | 0.0130 | 0.71 | 0.0042 |
| P09496 | Clathrin light chain A OS=Homo sapiens OX=9606 GN=CLTA PE=1 SV=1 | CLTA | 27.076 | 0.96 | 0.8473 | 0.92 | 0.8227 | 1.04 | 0.9468 |
| O94927 | HAUS augmin-like complex subunit 5 OS=Homo sapiens OX=9606 GN=HAUS5 PE=1 SV=2 | HAUS5 | 71.682 |  |  |  |  |  |  |
| Q99592 | Zinc finger and BTB domain-containing protein 18 OS=Homo sapiens OX=9606 GN=ZBTB18 PE=1 SV=1 | ZBTB18 | 58.354 | 1.54 | 0.1078 | 0.93 | 0.6489 | 1.66 | 0.0199 |
| P03951 | Coagulation factor XI OS=Homo sapiens OX=9606 GN=F11 PE=1 SV=1 | F11 | 70.108 | 0.50 | 0.0268 | 0.78 | 0.5222 | 0.65 | 0.0636 |
| Q6WBX8 | Cell cycle checkpoint control protein RAD9B OS=Homo sapiens OX=9606 GN=RAD9B PE=1 SV=2 | RAD9B | 47.831 |  |  |  |  |  |  |
| P24557 | Thromboxane-A synthase OS=Homo sapiens OX=9606 GN=TBXAS1 PE=1 SV=3 | TBXAS1 | 60.518 | 0.78 | 0.1333 | 0.81 | 0.4266 | 0.96 | 0.7461 |
| Q9H1V8 | Sodium-dependent neutral amino acid transporter SLC6A17 OS=Homo sapiens OX=9606 GN=SLC6A17 PE=1 SV=3 | SLC6A17 | 81.001 | 1.11 | 0.5601 | 0.81 | 0.2403 | 1.38 | 0.1135 |
| A0A075B6I0 | Immunoglobulin lambda variable 8-61 OS=Homo sapiens OX=9606 GN=IGLV8-61 PE=3 SV=7 | IGLV8-61 | 12.814 | 0.55 | 0.2598 | 0.64 | 0.0373 | 0.86 | 0.9150 |
| O15212 | Prefoldin subunit 6 OS=Homo sapiens OX=9606 GN=PFDN6 PE=1 SV=1 | PFDN6 | 14.582 | 0.57 | 0.2925 | 1.11 | 0.8528 | 0.51 | 0.0222 |
| Q9H756 | Leucine-rich repeat-containing protein 19 OS=Homo sapiens OX=9606 GN=LRRC19 PE=2 SV=1 | LRRC19 | 42.334 | 1.86 | 0.1624 | 1.22 | 0.4814 | 1.52 | 0.2653 |
| Q96L93 | Kinesin-like protein KIF16B OS=Homo sapiens OX=9606 GN=KIF16B PE=1 SV=2 | KIF16B | 152.01 |  |  |  |  |  |  |
| Q9Y4D1 | Disheveled-associated activator of morphogenesis 1 OS=Homo sapiens OX=9606 GN=DAAM1 PE=1 SV=2 | DAAM1 | 123.47 | 1.07 | 0.9267 | 0.85 | 0.8418 | 1.27 | 0.9189 |
| Q9P212 | 1-phosphatidylinositol 4,5-bisphosphate phosphodiesterase epsilon-1 OS=Homo sapiens OX=9606 GN=PLCE1 PE=1 SV=3 | PLCE1 | 258.71 | 1.12 | 0.6535 | 1.21 | 0.4349 | 0.92 | 0.6575 |
| Q68DA7 | Formin-1 OS=Homo sapiens OX=9606 GN=FMN1 PE=1 SV=3 | FMN1 | 157.58 |  |  |  |  |  |  |
| P00915 | Carbonic anhydrase 1 OS=Homo sapiens OX=9606 GN=CA1 PE=1 SV=2 | CA1 | 28.87 | 1.01 | 0.8688 | 0.83 | 0.2057 | 1.23 | 0.1988 |
| Q6UXG3 | CMRF35-like molecule 9 OS=Homo sapiens OX=9606 GN=CD300LG PE=1 SV=2 | CD300LG | 36.06 | 0.74 | 0.4568 | 0.83 | 0.9178 | 0.89 | 0.5236 |
| Q92686 | Neurogranin OS=Homo sapiens OX=9606 GN=NRGN PE=1 SV=1 | NRGN | 7.6184 | 2.33 | 0.3037 | 1.02 | 0.8654 | 2.30 | 0.3816 |
| Q9Y2I8 | WD repeat-containing protein 37 OS=Homo sapiens OX=9606 GN=WDR37 PE=1 SV=2 | WDR37 | 54.665 | 1.59 | 0.4748 | 1.05 | 0.9323 | 1.52 | 0.0441 |
| P40227 | T-complex protein 1 subunit zeta OS=Homo sapiens OX=9606 GN=CCT6A PE=1 SV=3 | CCT6A | 58.024 | 0.82 | 0.1255 | 0.73 | 0.0162 | 1.12 | 0.4104 |
| P49368 | T-complex protein 1 subunit gamma OS=Homo sapiens OX=9606 GN=CCT3 PE=1 SV=4 | CCT3 | 60.533 | 0.98 | 0.8851 | 0.95 | 0.8501 | 1.03 | 0.9747 |
| P11277 | Spectrin beta chain, erythrocytic OS=Homo sapiens OX=9606 GN=SPTB PE=1 SV=5 | SPTB | 246.47 | 0.79 | 0.5221 | 0.90 | 0.4480 | 0.88 | 0.8077 |
| P0DOX6 | Immunoglobulin mu heavy chain OS=Homo sapiens OX=9606 PE=1 SV=2 | --- | 63.485 | 0.73 | 0.0419 | 0.86 | 0.1335 | 0.85 | 0.1728 |
| Q8WW32 | High mobility group protein B4 OS=Homo sapiens OX=9606 GN=HMGB4 PE=1 SV=2 | HMGB4 | 22.49 | 1.82 | 0.5131 | 0.22 | 0.3811 | 8.35 | 0.2768 |
| Q5T848 | Probable G-protein coupled receptor 158 OS=Homo sapiens OX=9606 GN=GPR158 PE=1 SV=1 | GPR158 | 135.49 |  |  |  |  |  |  |
| P05109 | Protein S100-A8 OS=Homo sapiens OX=9606 GN=S100A8 PE=1 SV=1 | S100A8 | 10.834 | 0.67 | 0.2687 | 1.08 | 0.6233 | 0.62 | 0.1865 |
| P80108 | Phosphatidylinositol-glycan-specific phospholipase D OS=Homo sapiens OX=9606 GN=GPLD1 PE=1 SV=3 | GPLD1 | 92.335 | 1.12 | 0.6695 | 1.12 | 0.4806 | 1.00 | 0.9045 |
| P04196 | Histidine-rich glycoprotein OS=Homo sapiens OX=9606 GN=HRG PE=1 SV=1 | HRG | 59.578 | 0.73 | 0.1568 | 0.75 | 0.4396 | 0.97 | 0.4842 |
| Q9P1Z2 | Calcium-binding and coiled-coil domain-containing protein 1 OS=Homo sapiens OX=9606 GN=CALCOCO1 PE=1 SV=2 | CALCOCO1 | 77.335 | 1.07 | 0.9749 | 0.85 | 0.7302 | 1.27 | 0.7713 |
| P26038 | Moesin OS=Homo sapiens OX=9606 GN=MSN PE=1 SV=3 | MSN | 67.819 | 0.73 | 0.0625 | 0.90 | 0.4912 | 0.81 | 0.1499 |
| A0A0A0MT36 | Immunoglobulin kappa variable 6D-21 OS=Homo sapiens OX=9606 GN=IGKV6D-21 PE=3 SV=1 | IGKV6D-21 | 12.34 | 1.07 | 0.5583 | 0.83 | 0.3805 | 1.29 | 0.1972 |
| B0I1T2 | Unconventional myosin-Ig OS=Homo sapiens OX=9606 GN=MYO1G PE=1 SV=2 | MYO1G | 116.44 | 1.22 | 0.0831 | 1.05 | 0.6212 | 1.16 | 0.1977 |
| Q9UGM5 | Fetuin-B OS=Homo sapiens OX=9606 GN=FETUB PE=1 SV=2 | FETUB | 42.054 | 1.36 | 0.0796 | 1.30 | 0.0420 | 1.04 | 0.7166 |
| Q6ICG8 | Postacrosomal sheath WW domain-binding protein OS=Homo sapiens OX=9606 GN=WBP2NL PE=2 SV=1 | WBP2NL | 31.909 | 1.25 | 0.4383 | 1.08 | 0.5739 | 1.16 | 0.1799 |
| Q5TA45 | Integrator complex subunit 11 OS=Homo sapiens OX=9606 GN=INTS11 PE=1 SV=2 | INTS11 | 67.662 | 1.53 | 0.4683 | 0.72 | 0.6581 | 2.12 | 0.2535 |
| Q02985 | Complement factor H-related protein 3 OS=Homo sapiens OX=9606 GN=CFHR3 PE=1 SV=2 | CFHR3 | 37.323 | 3.01 | 0.0749 | 3.25 | 0.0068 | 0.93 | 0.6892 |
| P30536 | Translocator protein OS=Homo sapiens OX=9606 GN=TSPO PE=1 SV=3 | TSPO | 18.828 | 0.87 | 0.4263 | 0.81 | 0.1471 | 1.07 | 0.6746 |
| P17066 | Heat shock 70 kDa protein 6 OS=Homo sapiens OX=9606 GN=HSPA6 PE=1 SV=2 | HSPA6 | 71.027 | 1.07 | 0.6960 | 1.08 | 0.5354 | 0.99 | 0.9158 |
| P02675 | Fibrinogen beta chain OS=Homo sapiens OX=9606 GN=FGB PE=1 SV=2 | FGB | 55.928 | 2.67 | 0.0002 | 1.55 | 0.0112 | 1.72 | 0.0005 |
| Q9NRX1 | RNA-binding protein PNO1 OS=Homo sapiens OX=9606 GN=PNO1 PE=1 SV=1 | PNO1 | 27.924 | 0.75 | 0.2066 | 0.71 | 0.0931 | 1.06 | 0.8219 |
| Q16627 | C-C motif chemokine 14 OS=Homo sapiens OX=9606 GN=CCL14 PE=1 SV=1 | CCL14 | 10.678 | 1.05 | 0.9082 | 0.85 | 0.8992 | 1.23 | 0.9828 |
| P11678 | Eosinophil peroxidase OS=Homo sapiens OX=9606 GN=EPX PE=1 SV=2 | EPX | 81.04 | 0.50 | 0.5170 | 0.56 | 0.2901 | 0.89 | 0.9901 |
| P05107 | Integrin beta-2 OS=Homo sapiens OX=9606 GN=ITGB2 PE=1 SV=2 | ITGB2 | 84.781 | 1.07 | 0.7316 | 1.01 | 0.8790 | 1.06 | 0.8279 |
| P55774 | C-C motif chemokine 18 OS=Homo sapiens OX=9606 GN=CCL18 PE=1 SV=1 | CCL18 | 9.8487 | 0.79 | 0.5291 | 0.88 | 0.9667 | 0.89 | 0.6313 |
| O60308 | Centrosomal protein of 104 kDa OS=Homo sapiens OX=9606 GN=CEP104 PE=1 SV=1 | CEP104 | 104.45 | 1.27 | 0.8766 | 1.03 | 0.9870 | 1.23 | 0.8564 |
| Q13867 | Bleomycin hydrolase OS=Homo sapiens OX=9606 GN=BLMH PE=1 SV=1 | BLMH | 52.562 | 1.57 | 0.0296 | 1.10 | 0.5199 | 1.43 | 0.0142 |
| O15145 | Actin-related protein 2/3 complex subunit 3 OS=Homo sapiens OX=9606 GN=ARPC3 PE=1 SV=3 | ARPC3 | 20.546 | 0.48 | 0.0169 | 0.76 | 0.1785 | 0.63 | 0.0297 |
| Q9BV40 | Vesicle-associated membrane protein 8 OS=Homo sapiens OX=9606 GN=VAMP8 PE=1 SV=1 | VAMP8 | 11.438 | 1.05 | 0.6580 | 0.91 | 0.7199 | 1.15 | 0.5745 |
| Q31610 | HLA class I histocompatibility antigen, B-81 alpha chain OS=Homo sapiens OX=9606 GN=HLA-B PE=1 SV=1 | HLA-B | 40.4 |  |  |  |  |  |  |
| A0A075B6K5 | Immunoglobulin lambda variable 3-9 OS=Homo sapiens OX=9606 GN=IGLV3-9 PE=3 SV=1 | IGLV3-9 | 12.332 | 0.74 | 0.1733 | 1.07 | 0.8724 | 0.69 | 0.0152 |
| Q96RL7 | Vacuolar protein sorting-associated protein 13A OS=Homo sapiens OX=9606 GN=VPS13A PE=1 SV=2 | VPS13A | 360.27 |  |  |  |  |  |  |
| P19823 | Inter-alpha-trypsin inhibitor heavy chain H2 OS=Homo sapiens OX=9606 GN=ITIH2 PE=1 SV=2 | ITIH2 | 106.46 | 1.14 | 0.4091 | 1.04 | 0.6886 | 1.09 | 0.5965 |
| P61073 | C-X-C chemokine receptor type 4 OS=Homo sapiens OX=9606 GN=CXCR4 PE=1 SV=1 | CXCR4 | 39.745 | 0.95 | 0.7860 | 0.75 | 0.5288 | 1.28 | 0.4242 |
| P19447 | General transcription and DNA repair factor IIH helicase subunit XPB OS=Homo sapiens OX=9606 GN=ERCC3 PE=1 SV=1 | ERCC3 | 89.277 | 0.47 | 0.0885 | 0.62 | 0.0617 | 0.76 | 0.2586 |
| P02741 | C-reactive protein OS=Homo sapiens OX=9606 GN=CRP PE=1 SV=1 | CRP | 25.038 | 3.08 | 0.0663 | 2.44 | 0.0250 | 1.26 | 0.5326 |
| Q96MC4 | CEP295 N-terminal-like protein OS=Homo sapiens OX=9606 GN=CEP295NL PE=2 SV=1 | CEP295NL | 69.838 | 2.08 | 0.5743 | 0.86 | 0.8313 | 2.42 | 0.0799 |
| P08473 | Neprilysin OS=Homo sapiens OX=9606 GN=MME PE=1 SV=2 | MME | 85.513 | 1.43 | 0.4158 | 1.37 | 0.3761 | 1.04 | 0.7192 |
| Q8NG11 | Tetraspanin-14 OS=Homo sapiens OX=9606 GN=TSPAN14 PE=1 SV=1 | TSPAN14 | 30.69 | 0.65 | 0.0654 | 0.69 | 0.2443 | 0.94 | 0.5413 |
| P14618 | Pyruvate kinase PKM OS=Homo sapiens OX=9606 GN=PKM PE=1 SV=4 | PKM | 57.936 | 0.89 | 0.4197 | 0.79 | 0.1591 | 1.12 | 0.5905 |
| Q8TBP0 | TBC1 domain family member 16 OS=Homo sapiens OX=9606 GN=TBC1D16 PE=2 SV=1 | TBC1D16 | 86.371 | 0.88 | 0.8205 | 0.47 | 0.4314 | 1.87 | 0.5307 |
| O00451 | GDNF family receptor alpha-2 OS=Homo sapiens OX=9606 GN=GFRA2 PE=1 SV=2 | GFRA2 | 51.543 | 0.75 | 0.1445 | 0.81 | 0.5659 | 0.92 | 0.6321 |
| Q6DN90 | IQ motif and SEC7 domain-containing protein 1 OS=Homo sapiens OX=9606 GN=IQSEC1 PE=1 SV=1 | IQSEC1 | 108.31 |  |  |  |  |  |  |
| O15551 | Claudin-3 OS=Homo sapiens OX=9606 GN=CLDN3 PE=1 SV=1 | CLDN3 | 23.318 |  |  |  |  |  |  |
| Q00325 | Phosphate carrier protein, mitochondrial OS=Homo sapiens OX=9606 GN=SLC25A3 PE=1 SV=2 | SLC25A3 | 40.094 | 1.14 | 0.8929 | 1.55 | 0.8172 | 0.73 | 0.1871 |
| P10599 | Thioredoxin OS=Homo sapiens OX=9606 GN=TXN PE=1 SV=3 | TXN | 11.737 | 0.84 | 0.2198 | 0.86 | 0.2169 | 0.99 | 0.7760 |
| Q9Y2R2 | Tyrosine-protein phosphatase non-receptor type 22 OS=Homo sapiens OX=9606 GN=PTPN22 PE=1 SV=2 | PTPN22 | 91.704 | 1.70 | 0.0220 | 1.23 | 0.1133 | 1.39 | 0.0048 |
| P08572 | Collagen alpha-2(IV) chain OS=Homo sapiens OX=9606 GN=COL4A2 PE=1 SV=4 | COL4A2 | 167.55 | 1.68 | 0.0768 | 1.14 | 0.5135 | 1.48 | 0.4253 |
| Q5T2D3 | OTU domain-containing protein 3 OS=Homo sapiens OX=9606 GN=OTUD3 PE=1 SV=1 | OTUD3 | 45.124 | 1.21 | 0.1524 | 1.44 | 0.0795 | 0.84 | 0.4289 |
| Q99570 | Phosphoinositide 3-kinase regulatory subunit 4 OS=Homo sapiens OX=9606 GN=PIK3R4 PE=1 SV=3 | PIK3R4 | 153.1 | 1.75 | 0.0043 | 1.00 | 0.8511 | 1.76 | 0.1463 |
| O43760 | Synaptogyrin-2 OS=Homo sapiens OX=9606 GN=SYNGR2 PE=1 SV=1 | SYNGR2 | 24.81 |  |  |  |  |  |  |
| Q9H2D6 | TRIO and F-actin-binding protein OS=Homo sapiens OX=9606 GN=TRIOBP PE=1 SV=3 | TRIOBP | 261.37 |  |  |  |  |  |  |
| P22352 | Glutathione peroxidase 3 OS=Homo sapiens OX=9606 GN=GPX3 PE=1 SV=2 | GPX3 | 25.552 | 0.49 | 0.1485 | 1.33 | 0.1679 | 0.37 | 0.0970 |
| Q9ULA0 | Aspartyl aminopeptidase OS=Homo sapiens OX=9606 GN=DNPEP PE=1 SV=1 | DNPEP | 52.428 |  |  |  |  |  |  |
| P22105 | Tenascin-X OS=Homo sapiens OX=9606 GN=TNXB PE=1 SV=4 | TNXB | 458.22 | 1.47 | 0.2829 | 1.24 | 0.3893 | 1.18 | 0.3191 |
| O95841 | Angiopoietin-related protein 1 OS=Homo sapiens OX=9606 GN=ANGPTL1 PE=2 SV=1 | ANGPTL1 | 56.719 | 0.23 | 0.0448 | 0.56 | 0.1079 | 0.41 | 0.0108 |
| P80748 | Immunoglobulin lambda variable 3-21 OS=Homo sapiens OX=9606 GN=IGLV3-21 PE=1 SV=2 | IGLV3-21 | 12.446 | 1.03 | 0.5708 | 0.53 | 0.1394 | 1.97 | 0.0642 |
| P02647 | Apolipoprotein A-I OS=Homo sapiens OX=9606 GN=APOA1 PE=1 SV=1 | APOA1 | 30.777 | 0.71 | 0.0012 | 0.92 | 0.3494 | 0.77 | 0.0051 |
| P07948 | Tyrosine-protein kinase Lyn OS=Homo sapiens OX=9606 GN=LYN PE=1 SV=3 | LYN | 58.573 | 0.90 | 0.3884 | 0.75 | 0.0220 | 1.20 | 0.3440 |
| P06681 | Complement C2 OS=Homo sapiens OX=9606 GN=C2 PE=1 SV=2 | C2 | 83.267 | 0.99 | 0.8400 | 0.88 | 0.4893 | 1.12 | 0.6272 |
| P59998 | Actin-related protein 2/3 complex subunit 4 OS=Homo sapiens OX=9606 GN=ARPC4 PE=1 SV=3 | ARPC4 | 19.667 | 0.50 | 0.0181 | 0.81 | 0.4609 | 0.62 | 0.0392 |
| Q96Q89 | Kinesin-like protein KIF20B OS=Homo sapiens OX=9606 GN=KIF20B PE=1 SV=3 | KIF20B | 210.63 | 0.96 | 0.8130 | 0.22 | 0.5196 | 4.38 | 0.5765 |
| Q01780 | Exosome component 10 OS=Homo sapiens OX=9606 GN=EXOSC10 PE=1 SV=2 | EXOSC10 | 100.83 | 1.11 | 0.6758 | 1.05 | 0.7879 | 1.06 | 0.8846 |
| P01817 | Immunoglobulin heavy variable 2-5 OS=Homo sapiens OX=9606 GN=IGHV2-5 PE=1 SV=2 | IGHV2-5 | 13.231 | 0.92 | 0.4258 | 0.93 | 0.3899 | 0.99 | 0.9091 |
| Q02224 | Centromere-associated protein E OS=Homo sapiens OX=9606 GN=CENPE PE=1 SV=2 | CENPE | 316.41 | 0.75 | 0.4076 | 1.34 | 0.1336 | 0.56 | 0.0289 |
| Q9BXN1 | Asporin OS=Homo sapiens OX=9606 GN=ASPN PE=1 SV=2 | ASPN | 43.417 | 0.85 | 0.3423 | 0.97 | 0.7387 | 0.88 | 0.2884 |
| P11142 | Heat shock cognate 71 kDa protein OS=Homo sapiens OX=9606 GN=HSPA8 PE=1 SV=1 | HSPA8 | 70.897 | 0.94 | 0.4964 | 0.96 | 0.9256 | 0.98 | 0.7147 |
| P01766 | Immunoglobulin heavy variable 3-13 OS=Homo sapiens OX=9606 GN=IGHV3-13 PE=1 SV=2 | IGHV3-13 | 12.506 | 0.91 | 0.7196 | 1.28 | 0.3168 | 0.71 | 0.2050 |
| P03950 | Angiogenin OS=Homo sapiens OX=9606 GN=ANG PE=1 SV=1 | ANG | 16.55 | 1.05 | 0.7564 | 0.82 | 0.9442 | 1.28 | 0.6419 |
| Q13423 | NAD(P) transhydrogenase, mitochondrial OS=Homo sapiens OX=9606 GN=NNT PE=1 SV=3 | NNT | 113.89 | 1.23 | 0.1361 | 1.49 | 0.2018 | 0.82 | 0.4310 |
| Q9Y315 | Deoxyribose-phosphate aldolase OS=Homo sapiens OX=9606 GN=DERA PE=1 SV=2 | DERA | 35.23 | 1.02 | 0.9849 | 0.96 | 0.8595 | 1.06 | 0.7999 |
| P05534 | HLA class I histocompatibility antigen, A-24 alpha chain OS=Homo sapiens OX=9606 GN=HLA-A PE=1 SV=2 | HLA-A | 40.688 | 0.75 | 0.3350 | 1.05 | 0.7658 | 0.71 | 0.1176 |
| Q9Y2D4 | Exocyst complex component 6B OS=Homo sapiens OX=9606 GN=EXOC6B PE=1 SV=3 | EXOC6B | 94.2 | 1.45 | 0.6647 | 1.73 | 0.5501 | 0.84 | 0.6218 |
| P18465 | HLA class I histocompatibility antigen, B-57 alpha chain OS=Homo sapiens OX=9606 GN=HLA-B PE=1 SV=1 | HLA-B | 40.224 |  |  |  |  |  |  |
| Q96N64 | PWWP domain-containing protein 2A OS=Homo sapiens OX=9606 GN=PWWP2A PE=1 SV=2 | PWWP2A | 81.959 | 1.03 | 0.8233 | 0.33 | 0.5941 | 3.15 | 0.5649 |
| Q8IZT6 | Abnormal spindle-like microcephaly-associated protein OS=Homo sapiens OX=9606 GN=ASPM PE=1 SV=2 | ASPM | 409.8 | 0.72 | 0.4166 | 0.93 | 0.6084 | 0.77 | 0.1184 |
| P47755 | F-actin-capping protein subunit alpha-2 OS=Homo sapiens OX=9606 GN=CAPZA2 PE=1 SV=3 | CAPZA2 | 32.949 | 1.20 | 0.8323 | 0.93 | 0.9023 | 1.28 | 0.6362 |
| P11216 | Glycogen phosphorylase, brain form OS=Homo sapiens OX=9606 GN=PYGB PE=1 SV=5 | PYGB | 96.695 | 0.81 | 0.3812 | 0.90 | 0.6449 | 0.91 | 0.5808 |
| O43674 | NADH dehydrogenase [ubiquinone] 1 beta subcomplex subunit 5, mitochondrial OS=Homo sapiens OX=9606 GN=NDUFB5 PE=1 SV=1 | NDUFB5 | 21.75 | 1.69 | 0.5974 | 0.93 | 0.7898 | 1.82 | 0.1885 |
| P04179 | Superoxide dismutase [Mn], mitochondrial OS=Homo sapiens OX=9606 GN=SOD2 PE=1 SV=3 | SOD2 | 24.75 | 0.59 | 0.0493 | 0.68 | 0.4462 | 0.87 | 0.5589 |
| Q9BWP8 | Collectin-11 OS=Homo sapiens OX=9606 GN=COLEC11 PE=1 SV=1 | COLEC11 | 28.665 | 1.15 | 0.2858 | 0.99 | 0.8274 | 1.17 | 0.0954 |
| A0A0C4DH32 | Immunoglobulin heavy variable 3-20 OS=Homo sapiens OX=9606 GN=IGHV3-20 PE=3 SV=2 | IGHV3-20 | 12.673 | 1.11 | 0.5649 | 0.92 | 0.7225 | 1.20 | 0.3628 |
| Q13790 | Apolipoprotein F OS=Homo sapiens OX=9606 GN=APOF PE=1 SV=2 | APOF | 35.399 | 1.28 | 0.3279 | 1.21 | 0.3312 | 1.06 | 0.5536 |
| O43707 | Alpha-actinin-4 OS=Homo sapiens OX=9606 GN=ACTN4 PE=1 SV=2 | ACTN4 | 104.85 | 0.78 | 0.2282 | 0.80 | 0.3144 | 0.99 | 0.7012 |
| P07357 | Complement component C8 alpha chain OS=Homo sapiens OX=9606 GN=C8A PE=1 SV=2 | C8A | 65.163 | 1.15 | 0.5363 | 1.28 | 0.0945 | 0.90 | 0.3771 |
| P07225 | Vitamin K-dependent protein S OS=Homo sapiens OX=9606 GN=PROS1 PE=1 SV=1 | PROS1 | 75.122 | 1.34 | 0.0081 | 1.25 | 0.0329 | 1.07 | 0.1030 |
| Q01813 | ATP-dependent 6-phosphofructokinase, platelet type OS=Homo sapiens OX=9606 GN=PFKP PE=1 SV=2 | PFKP | 85.595 | 1.18 | 0.9705 | 0.63 | 0.4571 | 1.88 | 0.4681 |
| P17936 | Insulin-like growth factor-binding protein 3 OS=Homo sapiens OX=9606 GN=IGFBP3 PE=1 SV=2 | IGFBP3 | 31.674 | 0.82 | 0.1378 | 0.92 | 0.9140 | 0.89 | 0.0958 |
| A0A0C4DH39 | Immunoglobulin heavy variable 1-58 OS=Homo sapiens OX=9606 GN=IGHV1-58 PE=3 SV=1 | IGHV1-58 | 13.005 | 2.08 | 0.1583 | 1.57 | 0.2826 | 1.33 | 0.2850 |
| P27348 | 14-3-3 protein theta OS=Homo sapiens OX=9606 GN=YWHAQ PE=1 SV=1 | YWHAQ | 27.764 | 0.83 | 0.2057 | 0.77 | 0.2808 | 1.07 | 0.9903 |
| P06307 | Cholecystokinin OS=Homo sapiens OX=9606 GN=CCK PE=1 SV=1 | CCK | 12.669 | 1.34 | 0.4743 | 0.87 | 0.5136 | 1.53 | 0.0802 |
| P04899 | Guanine nucleotide-binding protein G(i) subunit alpha-2 OS=Homo sapiens OX=9606 GN=GNAI2 PE=1 SV=3 | GNAI2 | 40.45 | 0.88 | 0.4375 | 0.92 | 0.6061 | 0.96 | 0.7505 |
| P21291 | Cysteine and glycine-rich protein 1 OS=Homo sapiens OX=9606 GN=CSRP1 PE=1 SV=3 | CSRP1 | 20.567 | 0.89 | 0.0403 | 0.89 | 0.5993 | 1.00 | 0.8111 |
| Q9BXR6 | Complement factor H-related protein 5 OS=Homo sapiens OX=9606 GN=CFHR5 PE=1 SV=1 | CFHR5 | 64.419 | 1.87 | 0.0008 | 1.69 | 0.0000 | 1.11 | 0.2279 |
| Q92769 | Histone deacetylase 2 OS=Homo sapiens OX=9606 GN=HDAC2 PE=1 SV=2 | HDAC2 | 55.364 | 1.02 | 0.8682 | 0.87 | 0.2534 | 1.18 | 0.1071 |
| Q86U86 | Protein polybromo-1 OS=Homo sapiens OX=9606 GN=PBRM1 PE=1 SV=1 | PBRM1 | 192.95 | 2.23 | 0.3486 | 0.70 | 0.7922 | 3.18 | 0.0465 |
| P29622 | Kallistatin OS=Homo sapiens OX=9606 GN=SERPINA4 PE=1 SV=3 | SERPINA4 | 48.541 | 0.94 | 0.6343 | 0.93 | 0.3323 | 1.02 | 0.7922 |
| P22314 | Ubiquitin-like modifier-activating enzyme 1 OS=Homo sapiens OX=9606 GN=UBA1 PE=1 SV=3 | UBA1 | 117.85 |  |  |  |  |  |  |
| P78563 | Double-stranded RNA-specific editase 1 OS=Homo sapiens OX=9606 GN=ADARB1 PE=1 SV=1 | ADARB1 | 80.762 | 1.04 | 0.8125 | 0.63 | 0.6927 | 1.66 | 0.6127 |
| O95866 | Megakaryocyte and platelet inhibitory receptor G6b OS=Homo sapiens OX=9606 GN=MPIG6B PE=1 SV=1 | MPIG6B | 26.163 | 0.89 | 0.4912 | 0.79 | 0.0956 | 1.14 | 0.4032 |
| Q6KC79 | Nipped-B-like protein OS=Homo sapiens OX=9606 GN=NIPBL PE=1 SV=2 | NIPBL | 316.05 |  |  |  |  |  |  |
| Q8WWZ8 | Oncoprotein-induced transcript 3 protein OS=Homo sapiens OX=9606 GN=OIT3 PE=1 SV=2 | OIT3 | 60.021 | 1.64 | 0.0181 | 1.21 | 0.2173 | 1.36 | 0.0381 |
| P01705 | Immunoglobulin lambda variable 2-23 OS=Homo sapiens OX=9606 GN=IGLV2-23 PE=1 SV=2 | IGLV2-23 | 11.893 | 1.94 | 0.0371 | 1.20 | 0.3681 | 1.61 | 0.0780 |
| P05160 | Coagulation factor XIII B chain OS=Homo sapiens OX=9606 GN=F13B PE=1 SV=3 | F13B | 75.51 | 1.45 | 0.0398 | 1.37 | 0.0158 | 1.06 | 0.6595 |
| P05543 | Thyroxine-binding globulin OS=Homo sapiens OX=9606 GN=SERPINA7 PE=1 SV=2 | SERPINA7 | 46.324 | 1.34 | 0.2169 | 0.77 | 0.3611 | 1.74 | 0.0080 |
| P02766 | Transthyretin OS=Homo sapiens OX=9606 GN=TTR PE=1 SV=1 | TTR | 15.887 | 1.35 | 0.1364 | 0.96 | 0.6853 | 1.40 | 0.0260 |
| Q7Z406 | Myosin-14 OS=Homo sapiens OX=9606 GN=MYH14 PE=1 SV=2 | MYH14 | 227.87 | 1.08 | 0.9271 | 0.71 | 0.4565 | 1.51 | 0.5289 |
| Q8N5C6 | S1 RNA-binding domain-containing protein 1 OS=Homo sapiens OX=9606 GN=SRBD1 PE=1 SV=2 | SRBD1 | 111.77 | 0.76 | 0.5385 | 0.24 | 0.2201 | 3.23 | 0.6363 |
| A0A0J9YX35 | Immunoglobulin heavy variable 3-64D OS=Homo sapiens OX=9606 GN=IGHV3-64D PE=3 SV=1 | IGHV3-64D | 12.822 | 0.85 | 0.0398 | 0.86 | 0.2411 | 0.98 | 0.6762 |
| Q15848 | Adiponectin OS=Homo sapiens OX=9606 GN=ADIPOQ PE=1 SV=1 | ADIPOQ | 26.413 | 0.61 | 0.6309 | 1.08 | 0.4995 | 0.57 | 0.3000 |
| P05089 | Arginase-1 OS=Homo sapiens OX=9606 GN=ARG1 PE=1 SV=2 | ARG1 | 34.735 | 1.51 | 0.2937 | 1.30 | 0.3055 | 1.16 | 0.5004 |
| P0CF74 | Immunoglobulin lambda constant 6 OS=Homo sapiens OX=9606 GN=IGLC6 PE=1 SV=1 | IGLC6 | 11.276 |  |  |  |  |  |  |
| P01011 | Alpha-1-antichymotrypsin OS=Homo sapiens OX=9606 GN=SERPINA3 PE=1 SV=2 | SERPINA3 | 47.65 | 1.46 | 0.0418 | 1.24 | 0.1283 | 1.17 | 0.5141 |
| Q7L2Z9 | Centromere protein Q OS=Homo sapiens OX=9606 GN=CENPQ PE=1 SV=1 | CENPQ | 30.595 | 0.66 | 0.5628 | 0.68 | 0.2837 | 0.97 | 0.9338 |
| P31150 | Rab GDP dissociation inhibitor alpha OS=Homo sapiens OX=9606 GN=GDI1 PE=1 SV=2 | GDI1 | 50.582 | 0.78 | 0.2431 | 0.75 | 0.3548 | 1.05 | 0.9667 |
| A0A0A0MRZ9 | Immunoglobulin lambda variable 5-52 OS=Homo sapiens OX=9606 GN=IGLV5-52 PE=3 SV=1 | IGLV5-52 | 13.446 | 0.99 | 0.9217 | 0.60 | 0.2806 | 1.66 | 0.2928 |
| P15144 | Aminopeptidase N OS=Homo sapiens OX=9606 GN=ANPEP PE=1 SV=4 | ANPEP | 109.54 | 1.67 | 0.0713 | 1.45 | 0.0700 | 1.16 | 0.5177 |
| P00751 | Complement factor B OS=Homo sapiens OX=9606 GN=CFB PE=1 SV=2 | CFB | 85.532 | 1.26 | 0.2312 | 1.12 | 0.3855 | 1.13 | 0.7190 |
| P0DOX8 | Immunoglobulin lambda-1 light chain OS=Homo sapiens OX=9606 PE=1 SV=1 | --- | 22.83 | 0.64 | 0.0502 | 1.02 | 0.7628 | 0.63 | 0.0025 |
| P04083 | Annexin A1 OS=Homo sapiens OX=9606 GN=ANXA1 PE=1 SV=2 | ANXA1 | 38.714 | 0.92 | 0.7160 | 0.84 | 0.2690 | 1.10 | 0.5574 |
| P49790 | Nuclear pore complex protein Nup153 OS=Homo sapiens OX=9606 GN=NUP153 PE=1 SV=2 | NUP153 | 153.94 | 0.06 | 0.4716 | 0.55 | 0.4939 | 0.11 | 0.6299 |
| Q2M243 | Coiled-coil domain-containing protein 27 OS=Homo sapiens OX=9606 GN=CCDC27 PE=2 SV=2 | CCDC27 | 75.354 | 0.19 | 0.0891 | 1.09 | 0.6823 | 0.17 | 0.0014 |
| P12259 | Coagulation factor V OS=Homo sapiens OX=9606 GN=F5 PE=1 SV=4 | F5 | 251.7 | 1.01 | 0.9811 | 1.08 | 0.4086 | 0.94 | 0.4160 |
| Q9H4M9 | EH domain-containing protein 1 OS=Homo sapiens OX=9606 GN=EHD1 PE=1 SV=2 | EHD1 | 60.626 | 0.78 | 0.0275 | 0.76 | 0.1273 | 1.03 | 0.9038 |
| P31025 | Lipocalin-1 OS=Homo sapiens OX=9606 GN=LCN1 PE=1 SV=1 | LCN1 | 19.25 | 1.82 | 0.3930 | 1.24 | 0.6456 | 1.47 | 0.4365 |
| P36955 | Pigment epithelium-derived factor OS=Homo sapiens OX=9606 GN=SERPINF1 PE=1 SV=4 | SERPINF1 | 46.312 | 1.05 | 0.9810 | 0.65 | 0.3044 | 1.62 | 0.3118 |
| Q99878 | Histone H2A type 1-J OS=Homo sapiens OX=9606 GN=HIST1H2AJ PE=1 SV=3 | HIST1H2AJ | 13.936 | 0.70 | 0.7003 | 2.48 | 0.0451 | 0.28 | 0.0266 |
| P17693 | HLA class I histocompatibility antigen, alpha chain G OS=Homo sapiens OX=9606 GN=HLA-G PE=1 SV=1 | HLA-G | 38.224 | 0.78 | 0.2456 | 0.85 | 0.3619 | 0.92 | 0.5851 |
| Q92187 | CMP-N-acetylneuraminate-poly-alpha-2,8-sialyltransferase OS=Homo sapiens OX=9606 GN=ST8SIA4 PE=1 SV=1 | ST8SIA4 | 41.294 | 1.13 | 0.5276 | 0.88 | 0.5581 | 1.29 | 0.2541 |
| P35749 | Myosin-11 OS=Homo sapiens OX=9606 GN=MYH11 PE=1 SV=3 | MYH11 | 227.34 | 0.63 | 0.2271 | 0.89 | 0.4608 | 0.71 | 0.1573 |
| P22748 | Carbonic anhydrase 4 OS=Homo sapiens OX=9606 GN=CA4 PE=1 SV=2 | CA4 | 35.032 | 1.02 | 0.7040 | 0.81 | 0.0237 | 1.27 | 0.1003 |
| P05091 | Aldehyde dehydrogenase, mitochondrial OS=Homo sapiens OX=9606 GN=ALDH2 PE=1 SV=2 | ALDH2 | 56.381 | 1.80 | 0.3869 | 0.29 | 0.7682 | 6.22 | 0.4396 |
| P10124 | Serglycin OS=Homo sapiens OX=9606 GN=SRGN PE=1 SV=3 | SRGN | 17.652 | 0.59 | 0.0034 | 0.60 | 0.0204 | 0.97 | 0.6561 |
| P01877 | Immunoglobulin heavy constant alpha 2 OS=Homo sapiens OX=9606 GN=IGHA2 PE=1 SV=4 | IGHA2 | 36.591 | 4.17 | 0.1852 | 1.00 | 0.8323 | 4.18 | 0.1957 |
| P06727 | Apolipoprotein A-IV OS=Homo sapiens OX=9606 GN=APOA4 PE=1 SV=3 | APOA4 | 45.398 | 0.95 | 0.5778 | 0.82 | 0.1795 | 1.15 | 0.4200 |
| P67936 | Tropomyosin alpha-4 chain OS=Homo sapiens OX=9606 GN=TPM4 PE=1 SV=3 | TPM4 | 28.521 | 0.53 | 0.0243 | 0.76 | 0.3324 | 0.69 | 0.0862 |
| Q71U36 | Tubulin alpha-1A chain OS=Homo sapiens OX=9606 GN=TUBA1A PE=1 SV=1 | TUBA1A | 50.135 | 0.50 | 0.1230 | 1.05 | 0.8343 | 0.48 | 0.0174 |
| P63000 | Ras-related C3 botulinum toxin substrate 1 OS=Homo sapiens OX=9606 GN=RAC1 PE=1 SV=1 | RAC1 | 21.45 | 0.72 | 0.0547 | 0.87 | 0.3832 | 0.83 | 0.1923 |
| P16452 | Erythrocyte membrane protein band 4.2 OS=Homo sapiens OX=9606 GN=EPB42 PE=1 SV=3 | EPB42 | 77.008 | 0.90 | 0.6631 | 0.93 | 0.7246 | 0.97 | 0.8415 |
| Q96AJ9 | Vesicle transport through interaction with t-SNAREs homolog 1A OS=Homo sapiens OX=9606 GN=VTI1A PE=1 SV=2 | VTI1A | 25.217 | 0.57 | 0.1467 | 1.32 | 0.3374 | 0.43 | 0.0121 |
| Q9Y657 | Spindlin-1 OS=Homo sapiens OX=9606 GN=SPIN1 PE=1 SV=3 | SPIN1 | 29.6 | 1.18 | 0.3598 | 1.02 | 0.8888 | 1.16 | 0.3860 |
| Q08379 | Golgin subfamily A member 2 OS=Homo sapiens OX=9606 GN=GOLGA2 PE=1 SV=3 | GOLGA2 | 113.08 |  |  |  |  |  |  |
| A0A075B6H9 | Immunoglobulin lambda variable 4-69 OS=Homo sapiens OX=9606 GN=IGLV4-69 PE=1 SV=1 | IGLV4-69 | 12.773 | 0.96 | 0.9619 | 0.85 | 0.6472 | 1.12 | 0.6575 |
| P08151 | Zinc finger protein GLI1 OS=Homo sapiens OX=9606 GN=GLI1 PE=1 SV=1 | GLI1 | 117.9 | 2.10 | 0.1378 | 2.26 | 0.0644 | 0.93 | 0.6456 |
| Q16236 | Nuclear factor erythroid 2-related factor 2 OS=Homo sapiens OX=9606 GN=NFE2L2 PE=1 SV=3 | NFE2L2 | 67.826 | 0.48 | 0.2601 | 0.81 | 0.6268 | 0.59 | 0.1801 |
| P18085 | ADP-ribosylation factor 4 OS=Homo sapiens OX=9606 GN=ARF4 PE=1 SV=3 | ARF4 | 20.511 | 0.79 | 0.4058 | 0.72 | 0.3440 | 1.10 | 0.8929 |
| Q9Y4B5 | Microtubule cross-linking factor 1 OS=Homo sapiens OX=9606 GN=MTCL1 PE=1 SV=5 | MTCL1 | 209.52 | 2.63 | 0.3032 | 2.71 | 0.3588 | 0.97 | 0.9816 |
| Q9NW15 | Anoctamin-10 OS=Homo sapiens OX=9606 GN=ANO10 PE=1 SV=2 | ANO10 | 76.328 | 0.85 | 0.4603 | 0.80 | 0.2558 | 1.07 | 0.8167 |
| Q6P093 | Arylacetamide deacetylase-like 2 OS=Homo sapiens OX=9606 GN=AADACL2 PE=2 SV=3 | AADACL2 | 46.099 | 2.18 | 0.4800 | 0.62 | 0.5729 | 3.52 | 0.1687 |
| P21796 | Voltage-dependent anion-selective channel protein 1 OS=Homo sapiens OX=9606 GN=VDAC1 PE=1 SV=2 | VDAC1 | 30.772 | 1.14 | 0.6710 | 1.94 | 0.1816 | 0.59 | 0.0568 |
| P01116 | GTPase KRas OS=Homo sapiens OX=9606 GN=KRAS PE=1 SV=1 | KRAS | 21.656 |  |  |  |  |  |  |
| P17927 | Complement receptor type 1 OS=Homo sapiens OX=9606 GN=CR1 PE=1 SV=3 | CR1 | 223.66 | 1.13 | 0.7925 | 0.73 | 0.2059 | 1.54 | 0.0759 |
| P01024 | Complement C3 OS=Homo sapiens OX=9606 GN=C3 PE=1 SV=2 | C3 | 187.15 | 1.16 | 0.2075 | 1.17 | 0.1435 | 0.99 | 0.7939 |
| P27824 | Calnexin OS=Homo sapiens OX=9606 GN=CANX PE=1 SV=2 | CANX | 67.567 | 0.83 | 0.3017 | 0.74 | 0.1897 | 1.12 | 0.8046 |
| P62879 | Guanine nucleotide-binding protein G(I)/G(S)/G(T) subunit beta-2 OS=Homo sapiens OX=9606 GN=GNB2 PE=1 SV=3 | GNB2 | 37.331 | 1.13 | 0.6170 | 0.94 | 0.9523 | 1.20 | 0.7677 |
| P0DP08 | Immunoglobulin heavy variable 4-38-2 OS=Homo sapiens OX=9606 GN=IGHV4-38-2 PE=3 SV=1 | IGHV4-38-2 | 13.016 | 0.90 | 0.6835 | 1.08 | 0.3825 | 0.84 | 0.2366 |
| P30740 | Leukocyte elastase inhibitor OS=Homo sapiens OX=9606 GN=SERPINB1 PE=1 SV=1 | SERPINB1 | 42.741 | 0.85 | 0.3108 | 0.92 | 0.6395 | 0.93 | 0.5432 |
| Q13402 | Unconventional myosin-VIIa OS=Homo sapiens OX=9606 GN=MYO7A PE=1 SV=2 | MYO7A | 254.39 | 0.28 | 0.4824 | 0.74 | 0.6256 | 0.38 | 0.3176 |
| Q9BXF9 | Tektin-3 OS=Homo sapiens OX=9606 GN=TEKT3 PE=1 SV=1 | TEKT3 | 56.635 | 1.86 | 0.2011 | 0.57 | 0.3638 | 3.25 | 0.0588 |
| P01814 | Immunoglobulin heavy variable 2-70 OS=Homo sapiens OX=9606 GN=IGHV2-70 PE=1 SV=2 | IGHV2-70 | 13.26 | 2.68 | 0.2505 | 1.85 | 0.3719 | 1.45 | 0.2473 |
| P48741 | Putative heat shock 70 kDa protein 7 OS=Homo sapiens OX=9606 GN=HSPA7 PE=5 SV=2 | HSPA7 | 40.244 | 0.73 | 0.1900 | 0.80 | 0.2865 | 0.91 | 0.5416 |
| Q9UJU6 | Drebrin-like protein OS=Homo sapiens OX=9606 GN=DBNL PE=1 SV=1 | DBNL | 48.207 | 0.77 | 0.4716 | 0.62 | 0.3142 | 1.23 | 0.9114 |
| P16284 | Platelet endothelial cell adhesion molecule OS=Homo sapiens OX=9606 GN=PECAM1 PE=1 SV=2 | PECAM1 | 82.521 | 0.65 | 0.0311 | 0.76 | 0.1289 | 0.86 | 0.3094 |
| O60242 | Adhesion G protein-coupled receptor B3 OS=Homo sapiens OX=9606 GN=ADGRB3 PE=1 SV=2 | ADGRB3 | 171.52 | 0.81 | 0.2960 | 0.91 | 0.6595 | 0.89 | 0.4337 |
| P0DP01 | Immunoglobulin heavy variable 1-8 OS=Homo sapiens OX=9606 GN=IGHV1-8 PE=3 SV=1 | IGHV1-8 | 12.992 | 1.15 | 0.5714 | 1.25 | 0.3133 | 0.92 | 0.7004 |
| A0A0C4DH34 | Immunoglobulin heavy variable 4-28 OS=Homo sapiens OX=9606 GN=IGHV4-28 PE=3 SV=1 | IGHV4-28 | 13.124 | 0.96 | 0.9551 | 1.06 | 0.6148 | 0.91 | 0.6244 |
| Q99567 | Nuclear pore complex protein Nup88 OS=Homo sapiens OX=9606 GN=NUP88 PE=1 SV=2 | NUP88 | 83.541 | 0.59 | 0.0056 | 0.74 | 0.2054 | 0.81 | 0.0332 |
| Q9Y2H5 | Pleckstrin homology domain-containing family A member 6 OS=Homo sapiens OX=9606 GN=PLEKHA6 PE=1 SV=4 | PLEKHA6 | 117.13 |  |  |  |  |  |  |
| P62333 | 26S proteasome regulatory subunit 10B OS=Homo sapiens OX=9606 GN=PSMC6 PE=1 SV=1 | PSMC6 | 44.172 | 0.68 |  | 0.79 |  | 0.86 |  |
| Q29940 | HLA class I histocompatibility antigen, B-59 alpha chain OS=Homo sapiens OX=9606 GN=HLA-B PE=1 SV=1 | HLA-B | 40.584 |  |  |  |  |  |  |
| P31946 | 14-3-3 protein beta/alpha OS=Homo sapiens OX=9606 GN=YWHAB PE=1 SV=3 | YWHAB | 28.082 | 0.52 | 0.0142 | 0.76 | 0.1927 | 0.68 | 0.0642 |
| Q14574 | Desmocollin-3 OS=Homo sapiens OX=9606 GN=DSC3 PE=1 SV=3 | DSC3 | 99.968 | 0.62 | 0.3001 | 0.50 | 0.6636 | 1.25 | 0.8465 |
| O94811 | Tubulin polymerization-promoting protein OS=Homo sapiens OX=9606 GN=TPPP PE=1 SV=1 | TPPP | 23.693 | 2.08 | 0.1802 | 1.39 | 0.2774 | 1.49 | 0.1592 |
| P26927 | Hepatocyte growth factor-like protein OS=Homo sapiens OX=9606 GN=MST1 PE=1 SV=2 | MST1 | 80.319 | 0.87 | 0.3016 | 1.04 | 0.6501 | 0.84 | 0.0702 |
| Q5JQC9 | A-kinase anchor protein 4 OS=Homo sapiens OX=9606 GN=AKAP4 PE=1 SV=1 | AKAP4 | 94.476 |  |  |  |  |  |  |
| P61769 | Beta-2-microglobulin OS=Homo sapiens OX=9606 GN=B2M PE=1 SV=1 | B2M | 13.714 | 0.73 | 0.0723 | 0.79 | 0.2048 | 0.92 | 0.5779 |
| Q14344 | Guanine nucleotide-binding protein subunit alpha-13 OS=Homo sapiens OX=9606 GN=GNA13 PE=1 SV=2 | GNA13 | 44.049 | 0.80 | 0.1428 | 0.77 | 0.2808 | 1.04 | 0.8332 |
| P23229 | Integrin alpha-6 OS=Homo sapiens OX=9606 GN=ITGA6 PE=1 SV=5 | ITGA6 | 126.6 | 0.75 | 0.1250 | 0.79 | 0.2741 | 0.95 | 0.6272 |
| Q8WUW1 | Protein BRICK1 OS=Homo sapiens OX=9606 GN=BRK1 PE=1 SV=1 | BRK1 | 8.7448 | 0.79 | 0.0806 | 0.75 | 0.0662 | 1.05 | 0.9472 |
| O00161 | Synaptosomal-associated protein 23 OS=Homo sapiens OX=9606 GN=SNAP23 PE=1 SV=1 | SNAP23 | 23.354 | 0.94 | 0.5105 | 0.79 | 0.3056 | 1.20 | 0.5533 |
| P15814 | Immunoglobulin lambda-like polypeptide 1 OS=Homo sapiens OX=9606 GN=IGLL1 PE=1 SV=1 | IGLL1 | 22.963 | 1.21 | 0.4295 | 1.27 | 0.1740 | 0.95 | 0.9043 |
| Q12913 | Receptor-type tyrosine-protein phosphatase eta OS=Homo sapiens OX=9606 GN=PTPRJ PE=1 SV=3 | PTPRJ | 145.94 | 0.71 | 0.0349 | 0.81 | 0.1883 | 0.89 | 0.3076 |
| O95498 | Vascular non-inflammatory molecule 2 OS=Homo sapiens OX=9606 GN=VNN2 PE=1 SV=3 | VNN2 | 58.502 | 1.40 | 0.1789 | 1.05 | 0.6372 | 1.32 | 0.3084 |
| Q14094 | Cyclin-I OS=Homo sapiens OX=9606 GN=CCNI PE=1 SV=1 | CCNI | 42.557 | 0.95 | 0.9424 | 0.29 | 0.4119 | 3.26 | 0.4353 |
| Q7L5D6 | Golgi to ER traffic protein 4 homolog OS=Homo sapiens OX=9606 GN=GET4 PE=1 SV=1 | GET4 | 36.504 |  |  |  |  |  |  |
| P02794 | Ferritin heavy chain OS=Homo sapiens OX=9606 GN=FTH1 PE=1 SV=2 | FTH1 | 21.225 | 0.91 | 0.8312 | 0.75 | 0.3998 | 1.22 | 0.5656 |
| P61981 | 14-3-3 protein gamma OS=Homo sapiens OX=9606 GN=YWHAG PE=1 SV=2 | YWHAG | 28.302 | 0.83 | 0.3041 | 0.69 | 0.2045 | 1.21 | 0.6321 |
| P25786 | Proteasome subunit alpha type-1 OS=Homo sapiens OX=9606 GN=PSMA1 PE=1 SV=1 | PSMA1 | 29.555 | 0.94 | 0.8012 | 1.00 | 0.7966 | 0.94 | 0.6603 |
| P15291 | Beta-1,4-galactosyltransferase 1 OS=Homo sapiens OX=9606 GN=B4GALT1 PE=1 SV=5 | B4GALT1 | 43.92 | 1.18 | 0.9028 | 1.78 | 0.1252 | 0.66 | 0.0515 |
| P13598 | Intercellular adhesion molecule 2 OS=Homo sapiens OX=9606 GN=ICAM2 PE=1 SV=2 | ICAM2 | 30.654 |  |  |  |  |  |  |
| Q15834 | Coiled-coil domain-containing protein 85B OS=Homo sapiens OX=9606 GN=CCDC85B PE=1 SV=2 | CCDC85B | 22.091 |  |  |  |  |  |  |
| A5D8V7 | Coiled-coil domain-containing protein 151 OS=Homo sapiens OX=9606 GN=CCDC151 PE=1 SV=1 | CCDC151 | 69.139 |  |  |  |  |  |  |
| O95393 | Bone morphogenetic protein 10 OS=Homo sapiens OX=9606 GN=BMP10 PE=1 SV=1 | BMP10 | 48.047 | 1.05 | 0.9109 | 1.50 | 0.1542 | 0.70 | 0.2613 |
| Q14697 | Neutral alpha-glucosidase AB OS=Homo sapiens OX=9606 GN=GANAB PE=1 SV=3 | GANAB | 106.87 | 0.81 | 0.3127 | 1.10 | 0.5827 | 0.74 | 0.0356 |
| Q8TDG4 | Helicase POLQ-like OS=Homo sapiens OX=9606 GN=HELQ PE=1 SV=2 | HELQ | 124.13 |  |  |  |  |  |  |
| P10316 | HLA class I histocompatibility antigen, A-69 alpha chain OS=Homo sapiens OX=9606 GN=HLA-A PE=1 SV=2 | HLA-A | 40.976 |  |  |  |  |  |  |
| P10644 | cAMP-dependent protein kinase type I-alpha regulatory subunit OS=Homo sapiens OX=9606 GN=PRKAR1A PE=1 SV=1 | PRKAR1A | 42.981 | 0.80 | 0.2024 | 0.73 | 0.2053 | 1.08 | 0.9342 |
| P12111 | Collagen alpha-3(VI) chain OS=Homo sapiens OX=9606 GN=COL6A3 PE=1 SV=5 | COL6A3 | 343.67 | 1.18 | 0.2071 | 1.04 | 0.7150 | 1.13 | 0.1282 |
| Q8IY33 | MICAL-like protein 2 OS=Homo sapiens OX=9606 GN=MICALL2 PE=1 SV=1 | MICALL2 | 97.501 | 1.34 | 0.1560 | 1.33 | 0.1159 | 1.01 | 0.9005 |
| P07437 | Tubulin beta chain OS=Homo sapiens OX=9606 GN=TUBB PE=1 SV=2 | TUBB | 49.67 | 0.81 | 0.5539 | 0.89 | 0.7125 | 0.92 | 0.7228 |
| O75110 | Probable phospholipid-transporting ATPase IIA OS=Homo sapiens OX=9606 GN=ATP9A PE=1 SV=3 | ATP9A | 118.58 | 1.78 | 0.9737 | 3.96 | 0.6217 | 0.45 | 0.0603 |
| Q9H299 | SH3 domain-binding glutamic acid-rich-like protein 3 OS=Homo sapiens OX=9606 GN=SH3BGRL3 PE=1 SV=1 | SH3BGRL3 | 10.438 | 0.45 | 0.0055 | 0.72 | 0.5275 | 0.62 | 0.0649 |
| P50552 | Vasodilator-stimulated phosphoprotein OS=Homo sapiens OX=9606 GN=VASP PE=1 SV=3 | VASP | 39.829 | 0.68 | 0.2328 | 0.72 | 0.4029 | 0.95 | 0.6930 |
| Q16610 | Extracellular matrix protein 1 OS=Homo sapiens OX=9606 GN=ECM1 PE=1 SV=2 | ECM1 | 60.673 | 1.73 | 0.0041 | 0.86 | 0.6018 | 2.02 | 0.0001 |
| O75368 | SH3 domain-binding glutamic acid-rich-like protein OS=Homo sapiens OX=9606 GN=SH3BGRL PE=1 SV=1 | SH3BGRL | 12.774 | 0.85 | 0.3143 | 0.69 | 0.1702 | 1.22 | 0.4561 |
| P62330 | ADP-ribosylation factor 6 OS=Homo sapiens OX=9606 GN=ARF6 PE=1 SV=2 | ARF6 | 20.082 | 0.84 | 0.2499 | 0.82 | 0.4479 | 1.03 | 0.9533 |
| P30044 | Peroxiredoxin-5, mitochondrial OS=Homo sapiens OX=9606 GN=PRDX5 PE=1 SV=4 | PRDX5 | 22.086 | 0.57 | 0.1949 | 0.63 | 0.4852 | 0.91 | 0.6432 |
| P02747 | Complement C1q subcomponent subunit C OS=Homo sapiens OX=9606 GN=C1QC PE=1 SV=3 | C1QC | 25.773 | 0.98 | 0.8649 | 0.95 | 0.5024 | 1.03 | 0.6788 |
| Q99590 | Protein SCAF11 OS=Homo sapiens OX=9606 GN=SCAF11 PE=1 SV=2 | SCAF11 | 164.65 | 0.64 | 0.1428 | 0.63 | 0.3839 | 1.01 | 0.6169 |
| Q9HAV0 | Guanine nucleotide-binding protein subunit beta-4 OS=Homo sapiens OX=9606 GN=GNB4 PE=1 SV=3 | GNB4 | 37.567 | 1.02 | 0.8582 | 0.80 | 0.5651 | 1.27 | 0.5130 |
| Q9NY65 | Tubulin alpha-8 chain OS=Homo sapiens OX=9606 GN=TUBA8 PE=1 SV=1 | TUBA8 | 50.093 | 0.87 | 0.1215 | 0.96 | 0.7598 | 0.90 | 0.3016 |
| A0A075B6J9 | Immunoglobulin lambda variable 2-18 OS=Homo sapiens OX=9606 GN=IGLV2-18 PE=3 SV=2 | IGLV2-18 | 12.412 | 0.79 | 0.3135 | 1.21 | 0.2040 | 0.66 | 0.0718 |
| P18065 | Insulin-like growth factor-binding protein 2 OS=Homo sapiens OX=9606 GN=IGFBP2 PE=1 SV=2 | IGFBP2 | 34.814 | 1.06 | 0.8989 | 0.89 | 0.4464 | 1.19 | 0.3452 |
| P04075 | Fructose-bisphosphate aldolase A OS=Homo sapiens OX=9606 GN=ALDOA PE=1 SV=2 | ALDOA | 39.42 | 0.56 | 0.0074 | 0.77 | 0.1088 | 0.73 | 0.0358 |
| P02743 | Serum amyloid P-component OS=Homo sapiens OX=9606 GN=APCS PE=1 SV=2 | APCS | 25.387 | 3.07 | 0.0960 | 2.55 | 0.1773 | 1.20 | 0.2667 |
| P49913 | Cathelicidin antimicrobial peptide OS=Homo sapiens OX=9606 GN=CAMP PE=1 SV=1 | CAMP | 19.301 | 0.77 | 0.4782 | 0.78 | 0.4769 | 0.98 | 0.8723 |
| P0DP25 | Calmodulin-3 OS=Homo sapiens OX=9606 GN=CALM3 PE=1 SV=1 | CALM3 | 16.837 | 0.70 | 0.0338 | 0.81 | 0.3602 | 0.86 | 0.3150 |
| P05023 | Sodium/potassium-transporting ATPase subunit alpha-1 OS=Homo sapiens OX=9606 GN=ATP1A1 PE=1 SV=1 | ATP1A1 | 112.89 | 0.92 | 0.6759 | 0.92 | 0.4978 | 1.00 | 0.9362 |
| Q6UWL2 | Sushi domain-containing protein 1 OS=Homo sapiens OX=9606 GN=SUSD1 PE=1 SV=1 | SUSD1 | 82.709 | 0.66 | 0.0709 | 0.78 | 0.1073 | 0.86 | 0.3785 |
| Q8N3C7 | CAP-Gly domain-containing linker protein 4 OS=Homo sapiens OX=9606 GN=CLIP4 PE=1 SV=1 | CLIP4 | 76.316 | 0.56 | 0.3946 | 0.93 | 0.6391 | 0.61 | 0.1264 |
| Q3ZCW2 | Galectin-related protein OS=Homo sapiens OX=9606 GN=LGALSL PE=1 SV=2 | LGALSL | 18.986 |  |  |  |  |  |  |
| O15143 | Actin-related protein 2/3 complex subunit 1B OS=Homo sapiens OX=9606 GN=ARPC1B PE=1 SV=3 | ARPC1B | 40.949 | 0.71 | 0.0676 | 0.81 | 0.4349 | 0.88 | 0.4043 |
| P15170 | Eukaryotic peptide chain release factor GTP-binding subunit ERF3A OS=Homo sapiens OX=9606 GN=GSPT1 PE=1 SV=1 | GSPT1 | 55.755 | 1.32 | 0.3818 | 0.95 | 0.6110 | 1.39 | 0.0058 |
| Q9BT92 | Trichoplein keratin filament-binding protein OS=Homo sapiens OX=9606 GN=TCHP PE=1 SV=1 | TCHP | 61.071 | 0.18 | 0.0563 | 0.38 | 0.3027 | 0.48 | 0.2081 |
| Q08554 | Desmocollin-1 OS=Homo sapiens OX=9606 GN=DSC1 PE=1 SV=2 | DSC1 | 99.986 | 1.28 | 0.0161 | 0.95 | 0.6988 | 1.35 | 0.0140 |
| P62820 | Ras-related protein Rab-1A OS=Homo sapiens OX=9606 GN=RAB1A PE=1 SV=3 | RAB1A | 22.677 | 0.96 | 0.7621 | 0.90 | 0.6351 | 1.07 | 0.8585 |
| P23528 | Cofilin-1 OS=Homo sapiens OX=9606 GN=CFL1 PE=1 SV=3 | CFL1 | 18.502 | 0.38 | 0.0018 | 0.68 | 0.2533 | 0.56 | 0.0162 |
| Q6N043 | Zinc finger protein 280D OS=Homo sapiens OX=9606 GN=ZNF280D PE=1 SV=3 | ZNF280D | 109.28 | 0.56 | 0.3282 | 0.64 | 0.2091 | 0.86 | 0.5697 |
| Q02161 | Blood group Rh(D) polypeptide OS=Homo sapiens OX=9606 GN=RHD PE=1 SV=3 | RHD | 45.21 | 0.54 | 0.1965 | 0.40 | 0.0948 | 1.36 | 0.6366 |
| Q9H6D3 | XK-related protein 8 OS=Homo sapiens OX=9606 GN=XKR8 PE=1 SV=1 | XKR8 | 44.654 | 1.03 | 0.8758 | 0.68 | 0.6424 | 1.50 | 0.5588 |
| Q6ZRY4 | RNA-binding protein with multiple splicing 2 OS=Homo sapiens OX=9606 GN=RBPMS2 PE=1 SV=1 | RBPMS2 | 22.496 | 0.56 | 0.0574 | 0.72 | 0.0330 | 0.78 | 0.0568 |
| P57739 | Claudin-2 OS=Homo sapiens OX=9606 GN=CLDN2 PE=1 SV=1 | CLDN2 | 24.548 | 1.66 | 0.0071 | 1.08 | 0.5259 | 1.54 | 0.0054 |
| P19256 | Lymphocyte function-associated antigen 3 OS=Homo sapiens OX=9606 GN=CD58 PE=1 SV=1 | CD58 | 28.147 | 1.11 | 0.6465 | 1.05 | 0.8402 | 1.05 | 0.5946 |
| Q9UK10 | Zinc finger protein 225 OS=Homo sapiens OX=9606 GN=ZNF225 PE=1 SV=2 | ZNF225 | 82.471 | 1.91 | 0.2642 | 1.00 | 0.9774 | 1.90 | 0.1020 |
| P02654 | Apolipoprotein C-I OS=Homo sapiens OX=9606 GN=APOC1 PE=1 SV=1 | APOC1 | 9.3318 | 1.07 | 0.5679 | 1.09 | 0.4080 | 0.99 | 0.8755 |
| A4UGR9 | Xin actin-binding repeat-containing protein 2 OS=Homo sapiens OX=9606 GN=XIRP2 PE=1 SV=2 | XIRP2 | 382.3 | 0.71 | 0.4295 | 1.03 | 0.9198 | 0.69 | 0.1121 |
| Q93050 | V-type proton ATPase 116 kDa subunit a isoform 1 OS=Homo sapiens OX=9606 GN=ATP6V0A1 PE=1 SV=3 | ATP6V0A1 | 96.412 | 1.06 | 0.9090 | 0.79 | 0.6084 | 1.34 | 0.5348 |
| O43866 | CD5 antigen-like OS=Homo sapiens OX=9606 GN=CD5L PE=1 SV=1 | CD5L | 38.087 | 0.76 | 0.0343 | 0.88 | 0.1896 | 0.87 | 0.1909 |
| P84085 | ADP-ribosylation factor 5 OS=Homo sapiens OX=9606 GN=ARF5 PE=1 SV=2 | ARF5 | 20.529 |  |  |  |  |  |  |
| Q16851 | UTP--glucose-1-phosphate uridylyltransferase OS=Homo sapiens OX=9606 GN=UGP2 PE=1 SV=5 | UGP2 | 56.94 | 0.55 | 0.3961 | 0.68 | 0.2997 | 0.81 | 0.8188 |
| Q15166 | Serum paraoxonase/lactonase 3 OS=Homo sapiens OX=9606 GN=PON3 PE=1 SV=3 | PON3 | 39.607 | 0.82 | 0.2301 | 0.99 | 0.8576 | 0.83 | 0.4233 |
| Q96SJ8 | Tetraspanin-18 OS=Homo sapiens OX=9606 GN=TSPAN18 PE=2 SV=1 | TSPAN18 | 27.71 | 0.78 | 0.2357 | 0.82 | 0.2065 | 0.95 | 0.7900 |
| P02655 | Apolipoprotein C-II OS=Homo sapiens OX=9606 GN=APOC2 PE=1 SV=1 | APOC2 | 11.284 | 1.55 | 0.0526 | 1.07 | 0.5223 | 1.45 | 0.0497 |
| P25311 | Zinc-alpha-2-glycoprotein OS=Homo sapiens OX=9606 GN=AZGP1 PE=1 SV=2 | AZGP1 | 34.258 | 1.05 | 0.8656 | 0.85 | 0.1921 | 1.24 | 0.1050 |
| P50995 | Annexin A11 OS=Homo sapiens OX=9606 GN=ANXA11 PE=1 SV=1 | ANXA11 | 54.389 | 0.88 | 0.3872 | 0.73 | 0.2724 | 1.21 | 0.8308 |
| Q9H788 | SH2 domain-containing protein 4A OS=Homo sapiens OX=9606 GN=SH2D4A PE=1 SV=1 | SH2D4A | 52.726 |  |  |  |  |  |  |
| P13746 | HLA class I histocompatibility antigen, A-11 alpha chain OS=Homo sapiens OX=9606 GN=HLA-A PE=1 SV=1 | HLA-A | 40.936 | 0.64 | 0.4617 | 0.97 | 0.8676 | 0.66 | 0.3365 |
| P05362 | Intercellular adhesion molecule 1 OS=Homo sapiens OX=9606 GN=ICAM1 PE=1 SV=2 | ICAM1 | 57.825 | 1.05 | 0.6963 | 1.13 | 0.3614 | 0.93 | 0.8289 |
| O15067 | Phosphoribosylformylglycinamidine synthase OS=Homo sapiens OX=9606 GN=PFAS PE=1 SV=4 | PFAS | 144.73 | 0.74 | 0.2550 | 1.16 | 0.5628 | 0.63 | 0.0860 |
| Q8NDX2 | Vesicular glutamate transporter 3 OS=Homo sapiens OX=9606 GN=SLC17A8 PE=1 SV=1 | SLC17A8 | 64.99 | 0.74 | 0.4254 | 0.85 | 0.4767 | 0.87 | 0.5206 |
| P52907 | F-actin-capping protein subunit alpha-1 OS=Homo sapiens OX=9606 GN=CAPZA1 PE=1 SV=3 | CAPZA1 | 32.922 | 0.85 | 0.2469 | 0.76 | 0.2213 | 1.12 | 0.7740 |
| P01721 | Immunoglobulin lambda variable 6-57 OS=Homo sapiens OX=9606 GN=IGLV6-57 PE=1 SV=2 | IGLV6-57 | 12.566 | 1.65 | 0.2806 | 1.82 | 0.0418 | 0.91 | 0.3747 |
| Q9UHB6 | LIM domain and actin-binding protein 1 OS=Homo sapiens OX=9606 GN=LIMA1 PE=1 SV=1 | LIMA1 | 85.225 |  |  |  |  |  |  |
| Q15019 | Septin-2 OS=Homo sapiens OX=9606 GN=SEPT2 PE=1 SV=1 | SEPT2 | 41.487 | 0.74 | 0.4368 | 0.56 | 0.3197 | 1.32 | 0.8876 |
| P18428 | Lipopolysaccharide-binding protein OS=Homo sapiens OX=9606 GN=LBP PE=1 SV=3 | LBP | 53.383 | 2.19 | 0.0179 | 2.17 | 0.0007 | 1.01 | 0.9992 |
| Q08188 | Protein-glutamine gamma-glutamyltransferase E OS=Homo sapiens OX=9606 GN=TGM3 PE=1 SV=4 | TGM3 | 76.631 | 1.42 | 0.2468 | 1.01 | 0.9888 | 1.41 | 0.1378 |
| Q9H1C7 | Cysteine-rich and transmembrane domain-containing protein 1 OS=Homo sapiens OX=9606 GN=CYSTM1 PE=1 SV=1 | CYSTM1 | 10.631 | 1.02 | 0.8271 | 0.95 | 0.7807 | 1.08 | 0.6713 |
| Q9Y2Q0 | Phospholipid-transporting ATPase IA OS=Homo sapiens OX=9606 GN=ATP8A1 PE=1 SV=1 | ATP8A1 | 131.37 | 0.81 | 0.5983 | 0.82 | 0.1021 | 0.98 | 0.9296 |
| Q8NCR6 | Spermatid-specific manchette-related protein 1 OS=Homo sapiens OX=9606 GN=SMRP1 PE=1 SV=2 | SMRP1 | 30.166 |  |  |  |  |  |  |
| P52565 | Rho GDP-dissociation inhibitor 1 OS=Homo sapiens OX=9606 GN=ARHGDIA PE=1 SV=3 | ARHGDIA | 23.207 |  |  |  |  |  |  |
| Q9Y6D9 | Mitotic spindle assembly checkpoint protein MAD1 OS=Homo sapiens OX=9606 GN=MAD1L1 PE=1 SV=2 | MAD1L1 | 83.066 | 1.53 | 0.3061 | 1.05 | 0.8650 | 1.45 | 0.1940 |
| Q9Y696 | Chloride intracellular channel protein 4 OS=Homo sapiens OX=9606 GN=CLIC4 PE=1 SV=4 | CLIC4 | 28.772 | 0.92 | 0.7138 | 0.57 | 0.2788 | 1.61 | 0.4904 |
| P12270 | Nucleoprotein TPR OS=Homo sapiens OX=9606 GN=TPR PE=1 SV=3 | TPR | 267.29 | 1.01 | 0.8685 | 0.99 | 0.7923 | 1.03 | 0.9149 |
| P08519 | Apolipoprotein(a) OS=Homo sapiens OX=9606 GN=LPA PE=1 SV=1 | LPA | 501.31 | 1.39 | 0.1120 | 1.12 | 0.4342 | 1.24 | 0.1230 |
| P42357 | Histidine ammonia-lyase OS=Homo sapiens OX=9606 GN=HAL PE=1 SV=1 | HAL | 72.697 | 0.92 | 0.5605 | 1.10 | 0.2384 | 0.83 | 0.0929 |
| P26447 | Protein S100-A4 OS=Homo sapiens OX=9606 GN=S100A4 PE=1 SV=1 | S100A4 | 11.728 | 0.76 | 0.1407 | 0.67 | 0.0490 | 1.12 | 0.6524 |
| Q92994 | Transcription factor IIIB 90 kDa subunit OS=Homo sapiens OX=9606 GN=BRF1 PE=1 SV=1 | BRF1 | 73.839 | 0.37 | 0.0693 | 0.76 | 0.4095 | 0.49 | 0.0689 |
| Q8N323 | NXPE family member 1 OS=Homo sapiens OX=9606 GN=NXPE1 PE=2 SV=2 | NXPE1 | 63.177 |  |  |  |  |  |  |
| P47989 | Xanthine dehydrogenase/oxidase OS=Homo sapiens OX=9606 GN=XDH PE=1 SV=4 | XDH | 146.42 |  |  |  |  |  |  |
| O76074 | cGMP-specific 3',5'-cyclic phosphodiesterase OS=Homo sapiens OX=9606 GN=PDE5A PE=1 SV=2 | PDE5A | 99.984 | 0.73 | 0.1639 | 0.63 | 0.0578 | 1.16 | 0.5707 |
| P07237 | Protein disulfide-isomerase OS=Homo sapiens OX=9606 GN=P4HB PE=1 SV=3 | P4HB | 57.116 | 0.82 | 0.2452 | 0.82 | 0.2396 | 1.00 | 0.9038 |
| P61106 | Ras-related protein Rab-14 OS=Homo sapiens OX=9606 GN=RAB14 PE=1 SV=4 | RAB14 | 23.897 | 1.00 | 0.9697 | 0.81 | 0.3772 | 1.24 | 0.4330 |
| O95858 | Tetraspanin-15 OS=Homo sapiens OX=9606 GN=TSPAN15 PE=1 SV=1 | TSPAN15 | 33.165 | 0.99 | 0.8224 | 0.80 | 0.2919 | 1.23 | 0.1074 |
| P01715 | Immunoglobulin lambda variable 3-1 OS=Homo sapiens OX=9606 GN=IGLV3-1 PE=1 SV=2 | IGLV3-1 | 12.296 | 0.92 | 0.9836 | 1.37 | 0.0610 | 0.67 | 0.1046 |
| P27708 | CAD protein OS=Homo sapiens OX=9606 GN=CAD PE=1 SV=3 | CAD | 242.98 | 1.04 | 0.9709 | 0.90 | 0.6104 | 1.16 | 0.5808 |
| Q7Z408 | CUB and sushi domain-containing protein 2 OS=Homo sapiens OX=9606 GN=CSMD2 PE=1 SV=2 | CSMD2 | 380.03 | 2.43 | 0.0347 | 1.31 | 0.0306 | 1.86 | 0.0002 |
| P28715 | DNA repair protein complementing XP-G cells OS=Homo sapiens OX=9606 GN=ERCC5 PE=1 SV=3 | ERCC5 | 133.11 | 1.23 | 0.2410 | 0.90 | 0.3953 | 1.36 | 0.0164 |
| A0A0C4DH68 | Immunoglobulin kappa variable 2-24 OS=Homo sapiens OX=9606 GN=IGKV2-24 PE=3 SV=1 | IGKV2-24 | 13.079 | 0.55 | 0.0128 | 0.84 | 0.1851 | 0.66 | 0.0188 |
| P55209 | Nucleosome assembly protein 1-like 1 OS=Homo sapiens OX=9606 GN=NAP1L1 PE=1 SV=1 | NAP1L1 | 45.374 | 0.78 | 0.1467 | 0.86 | 0.5030 | 0.91 | 0.2162 |
| P01009 | Alpha-1-antitrypsin OS=Homo sapiens OX=9606 GN=SERPINA1 PE=1 SV=3 | SERPINA1 | 46.736 | 1.28 | 0.1830 | 0.87 | 0.7878 | 1.48 | 0.1877 |
| Q8IWA5 | Choline transporter-like protein 2 OS=Homo sapiens OX=9606 GN=SLC44A2 PE=1 SV=3 | SLC44A2 | 80.123 | 1.23 | 0.5898 | 0.84 | 0.7146 | 1.46 | 0.3908 |
| Q96LP6 | Uncharacterized protein C12orf42 OS=Homo sapiens OX=9606 GN=C12orf42 PE=2 SV=2 | C12orf42 | 39.738 | 1.76 | 0.1203 | 0.70 | 0.2494 | 2.50 | 0.0096 |
| Q02818 | Nucleobindin-1 OS=Homo sapiens OX=9606 GN=NUCB1 PE=1 SV=4 | NUCB1 | 53.879 | 1.31 | 0.4018 | 0.82 | 0.4854 | 1.59 | 0.1695 |
| Q9H0U4 | Ras-related protein Rab-1B OS=Homo sapiens OX=9606 GN=RAB1B PE=1 SV=1 | RAB1B | 22.171 | 0.71 | 0.0355 | 0.82 | 0.3123 | 0.87 | 0.3030 |
| O00560 | Syntenin-1 OS=Homo sapiens OX=9606 GN=SDCBP PE=1 SV=1 | SDCBP | 32.444 | 1.07 | 0.5295 | 0.92 | 0.5800 | 1.16 | 0.2734 |
| A0A0B4J1V6 | Immunoglobulin heavy variable 3-73 OS=Homo sapiens OX=9606 GN=IGHV3-73 PE=3 SV=1 | IGHV3-73 | 12.858 | 0.72 | 0.8343 | 0.56 | 0.2385 | 1.28 | 0.4428 |
| P54920 | Alpha-soluble NSF attachment protein OS=Homo sapiens OX=9606 GN=NAPA PE=1 SV=3 | NAPA | 33.232 | 0.83 | 0.1242 | 0.77 | 0.2420 | 1.08 | 0.9348 |
| Q9NQ79 | Cartilage acidic protein 1 OS=Homo sapiens OX=9606 GN=CRTAC1 PE=1 SV=2 | CRTAC1 | 71.42 | 0.91 | 0.1547 | 0.75 | 0.0016 | 1.21 | 0.1333 |
| P12830 | Cadherin-1 OS=Homo sapiens OX=9606 GN=CDH1 PE=1 SV=3 | CDH1 | 97.455 | 0.73 | 0.1506 | 0.75 | 0.6296 | 0.97 | 0.2192 |
| A0A075B6K0 | Immunoglobulin lambda variable 3-16 OS=Homo sapiens OX=9606 GN=IGLV3-16 PE=3 SV=2 | IGLV3-16 | 12.466 | 1.49 | 0.0276 | 0.95 | 0.9304 | 1.56 | 0.0787 |
| P07195 | L-lactate dehydrogenase B chain OS=Homo sapiens OX=9606 GN=LDHB PE=1 SV=2 | LDHB | 36.638 | 0.52 | 0.0049 | 0.63 | 0.0258 | 0.83 | 0.2164 |
| Q9UKX3 | Myosin-13 OS=Homo sapiens OX=9606 GN=MYH13 PE=2 SV=2 | MYH13 | 223.6 | 0.64 | 0.4610 | 0.96 | 0.7835 | 0.67 | 0.1869 |
| Q15758 | Neutral amino acid transporter B(0) OS=Homo sapiens OX=9606 GN=SLC1A5 PE=1 SV=2 | SLC1A5 | 56.598 | 1.08 | 0.4349 | 0.92 | 0.6049 | 1.17 | 0.1498 |
| P12814 | Alpha-actinin-1 OS=Homo sapiens OX=9606 GN=ACTN1 PE=1 SV=2 | ACTN1 | 103.06 | 0.56 | 0.0293 | 0.80 | 0.3181 | 0.70 | 0.0760 |
| P14770 | Platelet glycoprotein IX OS=Homo sapiens OX=9606 GN=GP9 PE=1 SV=3 | GP9 | 19.046 | 0.79 | 0.1729 | 1.05 | 0.7004 | 0.76 | 0.0348 |
| A0A087WSY6 | Immunoglobulin kappa variable 3D-15 OS=Homo sapiens OX=9606 GN=IGKV3D-15 PE=3 SV=6 | IGKV3D-15 | 12.534 | 0.86 | 0.3934 | 0.86 | 0.3228 | 1.00 | 0.9183 |
| P0DOY3 | Immunoglobulin lambda constant 3 OS=Homo sapiens OX=9606 GN=IGLC3 PE=1 SV=1 | IGLC3 | 11.265 | 0.80 | 0.1344 | 0.94 | 0.4888 | 0.85 | 0.1513 |
| P60709 | Actin, cytoplasmic 1 OS=Homo sapiens OX=9606 GN=ACTB PE=1 SV=1 | ACTB | 41.736 | 0.45 | 0.0100 | 0.71 | 0.4136 | 0.64 | 0.0644 |
| Q9P0L0 | Vesicle-associated membrane protein-associated protein A OS=Homo sapiens OX=9606 GN=VAPA PE=1 SV=3 | VAPA | 27.893 | 1.09 | 0.7763 | 1.07 | 0.6984 | 1.02 | 0.7837 |
| P21926 | CD9 antigen OS=Homo sapiens OX=9606 GN=CD9 PE=1 SV=4 | CD9 | 25.416 | 0.66 | 0.0776 | 0.74 | 0.3556 | 0.90 | 0.4359 |
| Q9BYV6 | Tripartite motif-containing protein 55 OS=Homo sapiens OX=9606 GN=TRIM55 PE=1 SV=2 | TRIM55 | 60.465 | 0.60 | 0.0973 | 0.82 | 0.2352 | 0.73 | 0.1589 |
| Q15691 | Microtubule-associated protein RP/EB family member 1 OS=Homo sapiens OX=9606 GN=MAPRE1 PE=1 SV=3 | MAPRE1 | 29.999 | 1.11 | 0.7958 | 1.01 | 0.9438 | 1.11 | 0.6256 |
| P24593 | Insulin-like growth factor-binding protein 5 OS=Homo sapiens OX=9606 GN=IGFBP5 PE=1 SV=1 | IGFBP5 | 30.57 | 1.12 | 0.9406 | 0.70 | 0.6151 | 1.60 | 0.5898 |
| P28676 | Grancalcin OS=Homo sapiens OX=9606 GN=GCA PE=1 SV=2 | GCA | 24.01 | 1.35 | 0.9400 | 1.00 | 0.8298 | 1.35 | 0.6894 |
| A0A075B6I4 | Immunoglobulin lambda variable 10-54 OS=Homo sapiens OX=9606 GN=IGLV10-54 PE=3 SV=1 | IGLV10-54 | 12.395 | 0.55 | 0.1218 | 0.79 | 0.4589 | 0.69 | 0.2489 |
| P20160 | Azurocidin OS=Homo sapiens OX=9606 GN=AZU1 PE=1 SV=3 | AZU1 | 26.885 | 3.00 | 0.6190 | 2.84 | 0.6769 | 1.06 | 0.8561 |
| P80511 | Protein S100-A12 OS=Homo sapiens OX=9606 GN=S100A12 PE=1 SV=2 | S100A12 | 10.575 | 1.45 | 0.4695 | 1.26 | 0.5054 | 1.15 | 0.6173 |
| P02730 | Band 3 anion transport protein OS=Homo sapiens OX=9606 GN=SLC4A1 PE=1 SV=3 | SLC4A1 | 101.79 | 0.70 | 0.2187 | 0.84 | 0.2213 | 0.83 | 0.4684 |
| Q6ZUS5 | Coiled-coil domain-containing protein 121 OS=Homo sapiens OX=9606 GN=CCDC121 PE=1 SV=1 | CCDC121 | 33.06 | 3.61 | 0.0043 | 1.57 | 0.1067 | 2.30 | 0.0000 |
| Q9UBV8 | Peflin OS=Homo sapiens OX=9606 GN=PEF1 PE=1 SV=1 | PEF1 | 30.381 | 1.91 | 0.4147 | 1.34 | 0.5478 | 1.42 | 0.1661 |
| Q76LX8 | A disintegrin and metalloproteinase with thrombospondin motifs 13 OS=Homo sapiens OX=9606 GN=ADAMTS13 PE=1 SV=1 | ADAMTS13 | 153.6 | 1.40 |  | 0.67 | 0.2091 | 2.08 |  |
| Q15904 | V-type proton ATPase subunit S1 OS=Homo sapiens OX=9606 GN=ATP6AP1 PE=1 SV=2 | ATP6AP1 | 52.025 | 0.84 | 0.7075 | 0.51 | 0.2126 | 1.64 | 0.4650 |
| Q96IY4 | Carboxypeptidase B2 OS=Homo sapiens OX=9606 GN=CPB2 PE=1 SV=2 | CPB2 | 48.424 | 0.78 | 0.0849 | 0.82 | 0.5765 | 0.94 | 0.2327 |
| Q6UXI9 | Nephronectin OS=Homo sapiens OX=9606 GN=NPNT PE=2 SV=3 | NPNT | 61.906 | 1.28 | 0.5536 | 1.07 | 0.8824 | 1.20 | 0.3415 |
| P15924 | Desmoplakin OS=Homo sapiens OX=9606 GN=DSP PE=1 SV=3 | DSP | 331.77 | 1.49 | 0.1780 | 1.06 | 0.9156 | 1.41 | 0.0016 |
| Q8NBF2 | NHL repeat-containing protein 2 OS=Homo sapiens OX=9606 GN=NHLRC2 PE=1 SV=1 | NHLRC2 | 79.443 |  |  |  |  |  |  |
| Q8WVV4 | Protein POF1B OS=Homo sapiens OX=9606 GN=POF1B PE=1 SV=3 | POF1B | 68.064 | 0.61 | 0.0794 | 0.67 | 0.0228 | 0.91 | 0.3874 |
| P68366 | Tubulin alpha-4A chain OS=Homo sapiens OX=9606 GN=TUBA4A PE=1 SV=1 | TUBA4A | 49.924 | 0.67 | 0.0958 | 0.80 | 0.2044 | 0.83 | 0.2593 |
| P10635 | Cytochrome P450 2D6 OS=Homo sapiens OX=9606 GN=CYP2D6 PE=1 SV=2 | CYP2D6 | 55.769 | 0.16 | 0.4253 | 0.92 | 0.8524 | 0.17 | 0.4372 |
| P51572 | B-cell receptor-associated protein 31 OS=Homo sapiens OX=9606 GN=BCAP31 PE=1 SV=3 | BCAP31 | 27.991 | 0.84 | 0.3320 | 0.79 | 0.3615 | 1.06 | 0.9136 |
| O60610 | Protein diaphanous homolog 1 OS=Homo sapiens OX=9606 GN=DIAPH1 PE=1 SV=2 | DIAPH1 | 141.35 | 0.82 | 0.2097 | 0.75 | 0.2254 | 1.09 | 0.9234 |
| O60911 | Cathepsin L2 OS=Homo sapiens OX=9606 GN=CTSV PE=1 SV=2 | CTSV | 37.329 |  |  |  |  |  |  |
| O60234 | Glia maturation factor gamma OS=Homo sapiens OX=9606 GN=GMFG PE=1 SV=1 | GMFG | 16.801 | 0.78 | 0.1628 | 0.68 | 0.1432 | 1.15 | 0.7409 |
| P04114 | Apolipoprotein B-100 OS=Homo sapiens OX=9606 GN=APOB PE=1 SV=2 | APOB | 515.6 | 1.00 | 0.9023 | 1.12 | 0.4765 | 0.89 | 0.3594 |
| P08567 | Pleckstrin OS=Homo sapiens OX=9606 GN=PLEK PE=1 SV=3 | PLEK | 40.124 | 0.63 | 0.0236 | 0.76 | 0.1149 | 0.83 | 0.2005 |
| Q7Z401 | C-myc promoter-binding protein OS=Homo sapiens OX=9606 GN=DENND4A PE=1 SV=2 | DENND4A | 209.24 | 1.15 | 0.2852 | 0.13 | 0.4073 | 9.03 | 0.3515 |
| P01599 | Immunoglobulin kappa variable 1-17 OS=Homo sapiens OX=9606 GN=IGKV1-17 PE=1 SV=2 | IGKV1-17 | 12.778 | 0.78 | 0.3810 | 1.09 | 0.4552 | 0.72 | 0.0910 |
| Q14195 | Dihydropyrimidinase-related protein 3 OS=Homo sapiens OX=9606 GN=DPYSL3 PE=1 SV=1 | DPYSL3 | 61.963 | 1.15 | 0.1389 | 1.73 | 0.0360 | 0.67 | 0.2605 |
| P11166 | Solute carrier family 2, facilitated glucose transporter member 1 OS=Homo sapiens OX=9606 GN=SLC2A1 PE=1 SV=2 | SLC2A1 | 54.083 | 0.85 | 0.3151 | 0.88 | 0.1952 | 0.96 | 0.7925 |
| Q9H040 | SprT-like domain-containing protein Spartan OS=Homo sapiens OX=9606 GN=SPRTN PE=1 SV=2 | SPRTN | 55.133 | 0.47 | 0.1177 | 0.73 | 0.3835 | 0.65 | 0.1557 |
| P01743 | Immunoglobulin heavy variable 1-46 OS=Homo sapiens OX=9606 GN=IGHV1-46 PE=1 SV=2 | IGHV1-46 | 12.933 | 0.89 | 0.2926 | 0.97 | 0.9092 | 0.92 | 0.3779 |
| Q8TE73 | Dynein heavy chain 5, axonemal OS=Homo sapiens OX=9606 GN=DNAH5 PE=1 SV=3 | DNAH5 | 529.01 | 0.82 | 0.4707 | 0.96 | 0.7287 | 0.86 | 0.3899 |
| P06576 | ATP synthase subunit beta, mitochondrial OS=Homo sapiens OX=9606 GN=ATP5F1B PE=1 SV=3 | ATP5F1B | 56.559 | 0.86 | 0.2806 | 0.86 | 0.3573 | 1.00 | 0.8313 |
| Q9UBC0 | Hepatocyte nuclear factor 6 OS=Homo sapiens OX=9606 GN=ONECUT1 PE=2 SV=1 | ONECUT1 | 51.023 | 1.09 | 0.8276 | 1.10 | 0.6826 | 0.99 | 0.9189 |
| Q9H9A7 | RecQ-mediated genome instability protein 1 OS=Homo sapiens OX=9606 GN=RMI1 PE=1 SV=3 | RMI1 | 70.144 | 1.03 | 0.8507 | 1.09 | 0.4937 | 0.95 | 0.7078 |
| Q15555 | Microtubule-associated protein RP/EB family member 2 OS=Homo sapiens OX=9606 GN=MAPRE2 PE=1 SV=1 | MAPRE2 | 37.031 | 0.75 | 0.3668 | 0.86 | 0.3612 | 0.87 | 0.6687 |
| P08758 | Annexin A5 OS=Homo sapiens OX=9606 GN=ANXA5 PE=1 SV=2 | ANXA5 | 35.936 | 0.84 | 0.2482 | 0.67 | 0.0471 | 1.25 | 0.3775 |
| Q9HDC9 | Adipocyte plasma membrane-associated protein OS=Homo sapiens OX=9606 GN=APMAP PE=1 SV=2 | APMAP | 46.48 | 1.15 | 0.7030 | 1.32 | 0.2521 | 0.87 | 0.5714 |
| Q6UVK1 | Chondroitin sulfate proteoglycan 4 OS=Homo sapiens OX=9606 GN=CSPG4 PE=1 SV=2 | CSPG4 | 250.53 |  |  |  |  |  |  |
| Q6ZNJ1 | Neurobeachin-like protein 2 OS=Homo sapiens OX=9606 GN=NBEAL2 PE=1 SV=2 | NBEAL2 | 302.51 |  |  |  |  |  |  |
| P20742 | Pregnancy zone protein OS=Homo sapiens OX=9606 GN=PZP PE=1 SV=4 | PZP | 163.86 | 1.38 | 0.1489 | 1.39 | 0.0642 | 0.99 | 0.8181 |
| P26367 | Paired box protein Pax-6 OS=Homo sapiens OX=9606 GN=PAX6 PE=1 SV=2 | PAX6 | 46.683 | 1.02 | 0.8950 | 1.01 | 0.8442 | 1.01 | 0.9853 |
| Q86WA8 | Lon protease homolog 2, peroxisomal OS=Homo sapiens OX=9606 GN=LONP2 PE=1 SV=1 | LONP2 | 94.615 | 0.58 | 0.3227 | 0.56 | 0.4130 | 1.03 | 0.7627 |
| P01042 | Kininogen-1 OS=Homo sapiens OX=9606 GN=KNG1 PE=1 SV=2 | KNG1 | 71.957 | 0.58 | 0.0288 | 0.72 | 0.3543 | 0.81 | 0.1938 |
| Q15853 | Upstream stimulatory factor 2 OS=Homo sapiens OX=9606 GN=USF2 PE=1 SV=1 | USF2 | 36.954 | 1.38 |  | 0.80 | 0.6502 | 1.73 |  |
| Q9Y6C7 | Putative uncharacterized protein encoded by LINC00312 OS=Homo sapiens OX=9606 GN=LINC00312 PE=5 SV=1 | LINC00312 | 11.024 | 2.18 | 0.3213 | 0.96 | 0.9778 | 2.28 | 0.1480 |
| P12532 | Creatine kinase U-type, mitochondrial OS=Homo sapiens OX=9606 GN=CKMT1A PE=1 SV=1 | CKMT1A | 47.036 | 0.64 | 0.1796 | 0.61 | 0.0004 | 1.05 | 0.5956 |
| O75955 | Flotillin-1 OS=Homo sapiens OX=9606 GN=FLOT1 PE=1 SV=3 | FLOT1 | 47.355 |  |  |  |  |  |  |
| Q15700 | Disks large homolog 2 OS=Homo sapiens OX=9606 GN=DLG2 PE=1 SV=3 | DLG2 | 97.551 | 0.88 | 0.7009 | 0.75 | 0.6038 | 1.18 | 0.8515 |
| Q6PIF6 | Unconventional myosin-VIIb OS=Homo sapiens OX=9606 GN=MYO7B PE=1 SV=2 | MYO7B | 241.6 | 1.22 | 0.3327 | 0.94 | 0.5830 | 1.30 | 0.0708 |
| Q9NUQ9 | Protein FAM49B OS=Homo sapiens OX=9606 GN=FAM49B PE=1 SV=1 | FAM49B | 36.748 |  |  |  |  |  |  |
| Q8WZ69 | Putative uncharacterized protein C11orf40 OS=Homo sapiens OX=9606 GN=C11orf40 PE=2 SV=1 | C11orf40 | 24.619 | 0.56 | 0.0239 | 0.81 | 0.1940 | 0.69 | 0.0677 |
| Q9GZZ9 | Ubiquitin-like modifier-activating enzyme 5 OS=Homo sapiens OX=9606 GN=UBA5 PE=1 SV=1 | UBA5 | 44.863 | 15.24 | 0.3984 | 12.12 | 0.5072 | 1.26 | 0.7000 |
| Q9Y6B6 | GTP-binding protein SAR1b OS=Homo sapiens OX=9606 GN=SAR1B PE=1 SV=1 | SAR1B | 22.41 | 0.70 | 0.4993 | 0.87 | 0.8168 | 0.81 | 0.5128 |
| P22061 | Protein-L-isoaspartate(D-aspartate) O-methyltransferase OS=Homo sapiens OX=9606 GN=PCMT1 PE=1 SV=4 | PCMT1 | 24.636 | 0.69 | 0.4028 | 0.88 | 0.7244 | 0.78 | 0.3528 |
| Q15404 | Ras suppressor protein 1 OS=Homo sapiens OX=9606 GN=RSU1 PE=1 SV=3 | RSU1 | 31.54 | 0.67 | 0.0648 | 0.87 | 0.5116 | 0.78 | 0.1492 |
| P60520 | Gamma-aminobutyric acid receptor-associated protein-like 2 OS=Homo sapiens OX=9606 GN=GABARAPL2 PE=1 SV=1 | GABARAPL2 | 13.667 |  |  |  |  |  |  |
| O95229 | ZW10 interactor OS=Homo sapiens OX=9606 GN=ZWINT PE=1 SV=2 | ZWINT | 31.293 | 0.87 | 0.7539 | 0.35 | 0.4361 | 2.49 | 0.5472 |
| Q9BQE3 | Tubulin alpha-1C chain OS=Homo sapiens OX=9606 GN=TUBA1C PE=1 SV=1 | TUBA1C | 49.895 | 1.12 | 0.8889 | 0.86 | 0.5839 | 1.31 | 0.6791 |
| P31944 | Caspase-14 OS=Homo sapiens OX=9606 GN=CASP14 PE=1 SV=2 | CASP14 | 27.679 | 1.22 | 0.6295 | 0.87 | 0.4826 | 1.40 | 0.0234 |
| P01860 | Immunoglobulin heavy constant gamma 3 OS=Homo sapiens OX=9606 GN=IGHG3 PE=1 SV=2 | IGHG3 | 41.287 | 0.91 | 0.5141 | 0.99 | 0.7745 | 0.92 | 0.5049 |
| P09172 | Dopamine beta-hydroxylase OS=Homo sapiens OX=9606 GN=DBH PE=1 SV=3 | DBH | 69.064 | 0.32 | 0.4769 | 0.78 | 0.1133 | 0.41 | 0.5797 |
| P06753 | Tropomyosin alpha-3 chain OS=Homo sapiens OX=9606 GN=TPM3 PE=1 SV=2 | TPM3 | 32.95 | 0.66 | 0.1039 | 0.68 | 0.1995 | 0.97 | 0.5986 |
| Q8WWA0 | Intelectin-1 OS=Homo sapiens OX=9606 GN=ITLN1 PE=1 SV=1 | ITLN1 | 34.961 | 0.43 | 0.1527 | 0.37 | 0.1706 | 1.14 | 0.9996 |
| Q8NF91 | Nesprin-1 OS=Homo sapiens OX=9606 GN=SYNE1 PE=1 SV=4 | SYNE1 | 1011.1 | 0.93 | 0.7119 | 1.09 | 0.8763 | 0.86 | 0.5201 |
| Q15485 | Ficolin-2 OS=Homo sapiens OX=9606 GN=FCN2 PE=1 SV=2 | FCN2 | 34.001 | 2.56 | 0.0003 | 1.97 | 0.0007 | 1.30 | 0.3013 |
| Q7Z7G1 | Cytokine-dependent hematopoietic cell linker OS=Homo sapiens OX=9606 GN=CLNK PE=1 SV=2 | CLNK | 49.553 |  |  |  |  |  |  |
| Q99708 | DNA endonuclease RBBP8 OS=Homo sapiens OX=9606 GN=RBBP8 PE=1 SV=2 | RBBP8 | 101.94 | 0.53 | 0.1039 | 0.81 | 0.5676 | 0.65 | 0.1532 |
| Q8TEP8 | Centrosomal protein of 192 kDa OS=Homo sapiens OX=9606 GN=CEP192 PE=1 SV=3 | CEP192 | 279.11 | 0.14 | 0.0001 | 0.30 | 0.0879 | 0.48 | 0.1546 |
| Q5JV73 | FERM and PDZ domain-containing protein 3 OS=Homo sapiens OX=9606 GN=FRMPD3 PE=2 SV=2 | FRMPD3 | 199.21 | 2.48 | 0.0301 | 0.71 | 0.0718 | 3.47 | 0.0002 |
| Q15389 | Angiopoietin-1 OS=Homo sapiens OX=9606 GN=ANGPT1 PE=1 SV=2 | ANGPT1 | 57.512 | 0.80 | 0.1760 | 1.06 | 0.7051 | 0.76 | 0.0393 |
| Q6Q788 | Apolipoprotein A-V OS=Homo sapiens OX=9606 GN=APOA5 PE=1 SV=1 | APOA5 | 41.212 | 1.23 | 0.1898 | 1.10 | 0.3773 | 1.12 | 0.3267 |
| P12429 | Annexin A3 OS=Homo sapiens OX=9606 GN=ANXA3 PE=1 SV=3 | ANXA3 | 36.375 | 0.88 | 0.3893 | 0.77 | 0.3274 | 1.13 | 0.8643 |
| P15153 | Ras-related C3 botulinum toxin substrate 2 OS=Homo sapiens OX=9606 GN=RAC2 PE=1 SV=1 | RAC2 | 21.429 | 0.60 | 0.1008 | 0.78 | 0.2656 | 0.77 | 0.2158 |
| Q16828 | Dual specificity protein phosphatase 6 OS=Homo sapiens OX=9606 GN=DUSP6 PE=1 SV=2 | DUSP6 | 42.319 | 0.03 | 0.3605 | 0.41 | 0.3930 | 0.08 | 0.2173 |
| Q14520 | Hyaluronan-binding protein 2 OS=Homo sapiens OX=9606 GN=HABP2 PE=1 SV=1 | HABP2 | 62.671 | 1.30 | 0.1049 | 1.08 | 0.4352 | 1.21 | 0.4150 |
| A0A0C4DH67 | Immunoglobulin kappa variable 1-8 OS=Homo sapiens OX=9606 GN=IGKV1-8 PE=3 SV=1 | IGKV1-8 | 12.537 | 0.93 | 0.9749 | 1.16 | 0.5841 | 0.80 | 0.5953 |
| P04921 | Glycophorin-C OS=Homo sapiens OX=9606 GN=GYPC PE=1 SV=1 | GYPC | 13.81 | 0.97 | 0.8058 | 1.07 | 0.8408 | 0.90 | 0.5253 |
| P08238 | Heat shock protein HSP 90-beta OS=Homo sapiens OX=9606 GN=HSP90AB1 PE=1 SV=4 | HSP90AB1 | 83.263 | 1.31 | 0.4348 | 0.95 | 0.6026 | 1.39 | 0.0130 |
| Q15286 | Ras-related protein Rab-35 OS=Homo sapiens OX=9606 GN=RAB35 PE=1 SV=1 | RAB35 | 23.025 | 1.02 | 0.7695 | 0.84 | 0.3553 | 1.22 | 0.2979 |
| Q5TDH0 | Protein DDI1 homolog 2 OS=Homo sapiens OX=9606 GN=DDI2 PE=1 SV=1 | DDI2 | 44.522 |  |  |  |  |  |  |
| P0DP04 | Immunoglobulin heavy variable 3-43D OS=Homo sapiens OX=9606 GN=IGHV3-43D PE=3 SV=1 | IGHV3-43D | 13.017 | 0.68 | 0.0002 | 0.76 | 0.0195 | 0.90 | 0.3178 |
| P07203 | Glutathione peroxidase 1 OS=Homo sapiens OX=9606 GN=GPX1 PE=1 SV=4 | GPX1 | 22.088 | 0.69 | 0.1210 | 0.68 | 0.1134 | 1.00 | 0.8264 |
| Q9BR76 | Coronin-1B OS=Homo sapiens OX=9606 GN=CORO1B PE=1 SV=1 | CORO1B | 54.234 | 1.19 | 0.7065 | 1.07 | 0.7780 | 1.12 | 0.9069 |
| P63267 | Actin, gamma-enteric smooth muscle OS=Homo sapiens OX=9606 GN=ACTG2 PE=1 SV=1 | ACTG2 | 41.876 | 0.52 | 0.0078 | 0.75 | 0.2853 | 0.69 | 0.0536 |
| Q5VTE0 | Putative elongation factor 1-alpha-like 3 OS=Homo sapiens OX=9606 GN=EEF1A1P5 PE=5 SV=1 | EEF1A1P5 | 50.184 | 0.94 | 0.7200 | 0.94 | 0.4589 | 1.00 | 0.8948 |
| Q8NDA2 | Hemicentin-2 OS=Homo sapiens OX=9606 GN=HMCN2 PE=2 SV=3 | HMCN2 | 541.97 | 0.81 | 0.5535 | 0.43 | 0.1548 | 1.91 | 0.2918 |
| P02775 | Platelet basic protein OS=Homo sapiens OX=9606 GN=PPBP PE=1 SV=3 | PPBP | 13.894 | 0.65 | 0.1146 | 0.70 | 0.4776 | 0.94 | 0.3492 |
| P31939 | Bifunctional purine biosynthesis protein PURH OS=Homo sapiens OX=9606 GN=ATIC PE=1 SV=3 | ATIC | 64.615 |  |  | 0.61 | 0.2379 |  |  |
| P30041 | Peroxiredoxin-6 OS=Homo sapiens OX=9606 GN=PRDX6 PE=1 SV=3 | PRDX6 | 25.035 | 0.66 | 0.0507 | 0.85 | 0.4112 | 0.79 | 0.1516 |
| P61421 | V-type proton ATPase subunit d 1 OS=Homo sapiens OX=9606 GN=ATP6V0D1 PE=1 SV=1 | ATP6V0D1 | 40.329 | 1.04 | 0.7677 | 0.95 | 0.9836 | 1.09 | 0.8182 |
| P50993 | Sodium/potassium-transporting ATPase subunit alpha-2 OS=Homo sapiens OX=9606 GN=ATP1A2 PE=1 SV=1 | ATP1A2 | 112.26 |  |  |  |  |  |  |
| P63104 | 14-3-3 protein zeta/delta OS=Homo sapiens OX=9606 GN=YWHAZ PE=1 SV=1 | YWHAZ | 27.745 | 0.58 | 0.0219 | 0.80 | 0.3428 | 0.72 | 0.0729 |
| Q14247 | Src substrate cortactin OS=Homo sapiens OX=9606 GN=CTTN PE=1 SV=2 | CTTN | 61.585 | 0.65 | 0.0264 | 0.81 | 0.3011 | 0.80 | 0.1970 |
| A0A0B4J1V0 | Immunoglobulin heavy variable 3-15 OS=Homo sapiens OX=9606 GN=IGHV3-15 PE=3 SV=1 | IGHV3-15 | 12.926 | 0.74 | 0.0052 | 0.87 | 0.2009 | 0.85 | 0.0709 |
| C9JLR9 | Uncharacterized protein C11orf95 OS=Homo sapiens OX=9606 GN=C11orf95 PE=1 SV=1 | C11orf95 | 73.197 |  |  |  |  |  |  |
| P15311 | Ezrin OS=Homo sapiens OX=9606 GN=EZR PE=1 SV=4 | EZR | 69.412 |  |  |  |  |  |  |
| P0DPH8 | Tubulin alpha-3D chain OS=Homo sapiens OX=9606 GN=TUBA3D PE=1 SV=1 | TUBA3D | 49.959 |  |  |  |  |  |  |
| P02649 | Apolipoprotein E OS=Homo sapiens OX=9606 GN=APOE PE=1 SV=1 | APOE | 36.154 | 1.05 | 0.7682 | 0.92 | 0.5392 | 1.14 | 0.3781 |
| O00194 | Ras-related protein Rab-27B OS=Homo sapiens OX=9606 GN=RAB27B PE=1 SV=4 | RAB27B | 24.608 | 0.85 | 0.2480 | 0.79 | 0.1331 | 1.08 | 0.7303 |
| P20618 | Proteasome subunit beta type-1 OS=Homo sapiens OX=9606 GN=PSMB1 PE=1 SV=2 | PSMB1 | 26.489 | 1.61 | 0.1188 | 1.10 | 0.5737 | 1.46 | 0.5293 |
| P02745 | Complement C1q subcomponent subunit A OS=Homo sapiens OX=9606 GN=C1QA PE=1 SV=2 | C1QA | 26.016 | 1.00 | 0.9803 | 0.95 | 0.4616 | 1.05 | 0.4948 |
| A0A0A0MS15 | Immunoglobulin heavy variable 3-49 OS=Homo sapiens OX=9606 GN=IGHV3-49 PE=3 SV=1 | IGHV3-49 | 13.056 | 0.98 | 0.8161 | 1.25 | 0.4024 | 0.79 | 0.2065 |
| O95197 | Reticulon-3 OS=Homo sapiens OX=9606 GN=RTN3 PE=1 SV=2 | RTN3 | 112.61 | 0.89 | 0.5417 | 0.92 | 0.4988 | 0.96 | 0.6248 |
| P48735 | Isocitrate dehydrogenase [NADP], mitochondrial OS=Homo sapiens OX=9606 GN=IDH2 PE=1 SV=2 | IDH2 | 50.909 | 0.59 | 0.1010 | 0.79 | 0.3014 | 0.75 | 0.2012 |
| P02671 | Fibrinogen alpha chain OS=Homo sapiens OX=9606 GN=FGA PE=1 SV=2 | FGA | 94.972 | 2.44 | 0.0000 | 1.23 | 0.0947 | 1.98 | 0.0000 |
| P24043 | Laminin subunit alpha-2 OS=Homo sapiens OX=9606 GN=LAMA2 PE=1 SV=4 | LAMA2 | 343.9 |  |  |  |  |  |  |
| P09972 | Fructose-bisphosphate aldolase C OS=Homo sapiens OX=9606 GN=ALDOC PE=1 SV=2 | ALDOC | 39.455 | 0.95 | 0.5689 | 0.83 | 0.2577 | 1.14 | 0.5176 |
| P0DKL9 | ARL14 effector protein-like OS=Homo sapiens OX=9606 GN=ARL14EPL PE=4 SV=1 | ARL14EPL | 17.712 | 0.39 | 0.0406 | 0.49 | 0.2731 | 0.78 | 0.4518 |
| P61158 | Actin-related protein 3 OS=Homo sapiens OX=9606 GN=ACTR3 PE=1 SV=3 | ACTR3 | 47.371 | 0.65 | 0.0491 | 0.82 | 0.2451 | 0.79 | 0.1679 |
| Q8WZA2 | Rap guanine nucleotide exchange factor 4 OS=Homo sapiens OX=9606 GN=RAPGEF4 PE=1 SV=1 | RAPGEF4 | 115.52 | 1.08 | 0.5875 | 0.78 | 0.2889 | 1.38 | 0.1275 |
| Q92734 | Protein TFG OS=Homo sapiens OX=9606 GN=TFG PE=1 SV=2 | TFG | 43.447 | 0.88 | 0.5644 | 0.86 | 0.6140 | 1.03 | 0.9694 |
| P60953 | Cell division control protein 42 homolog OS=Homo sapiens OX=9606 GN=CDC42 PE=1 SV=2 | CDC42 | 21.258 | 0.55 | 0.0007 | 0.73 | 0.0239 | 0.75 | 0.0691 |
| A0A075B6S6 | Immunoglobulin kappa variable 2D-30 OS=Homo sapiens OX=9606 GN=IGKV2D-30 PE=3 SV=1 | IGKV2D-30 | 13.215 | 0.84 | 0.6705 | 0.95 | 0.9600 | 0.89 | 0.6615 |
| Q6UW60 | Proprotein convertase subtilisin/kexin type 4 OS=Homo sapiens OX=9606 GN=PCSK4 PE=1 SV=2 | PCSK4 | 82.794 | 0.60 | 0.0232 | 1.02 | 0.8821 | 0.59 | 0.0069 |
| Q8IYK2 | Coiled-coil domain-containing protein 105 OS=Homo sapiens OX=9606 GN=CCDC105 PE=2 SV=3 | CCDC105 | 56.908 |  |  |  |  |  |  |
| Q4LDE5 | Sushi, von Willebrand factor type A, EGF and pentraxin domain-containing protein 1 OS=Homo sapiens OX=9606 GN=SVEP1 PE=1 SV=3 | SVEP1 | 390.17 | 1.29 | 0.1808 | 1.22 | 0.1132 | 1.05 | 0.5268 |
| P37837 | Transaldolase OS=Homo sapiens OX=9606 GN=TALDO1 PE=1 SV=2 | TALDO1 | 37.54 | 0.62 | 0.0268 | 0.74 | 0.2006 | 0.84 | 0.2792 |
| P25789 | Proteasome subunit alpha type-4 OS=Homo sapiens OX=9606 GN=PSMA4 PE=1 SV=1 | PSMA4 | 29.483 | 1.18 | 0.5092 | 0.97 | 0.9512 | 1.21 | 0.4949 |
| P17987 | T-complex protein 1 subunit alpha OS=Homo sapiens OX=9606 GN=TCP1 PE=1 SV=1 | TCP1 | 60.343 | 0.99 | 0.8426 | 0.94 | 0.4570 | 1.05 | 0.3267 |
| Q9HD89 | Resistin OS=Homo sapiens OX=9606 GN=RETN PE=1 SV=1 | RETN | 11.419 | 0.47 | 0.0065 | 0.74 | 0.0267 | 0.63 | 0.0184 |
| P06744 | Glucose-6-phosphate isomerase OS=Homo sapiens OX=9606 GN=GPI PE=1 SV=4 | GPI | 63.146 | 0.72 | 0.1048 | 0.81 | 0.2591 | 0.89 | 0.4284 |
| P25788 | Proteasome subunit alpha type-3 OS=Homo sapiens OX=9606 GN=PSMA3 PE=1 SV=2 | PSMA3 | 28.433 | 2.31 | 0.0676 | 1.23 | 0.4235 | 1.87 | 0.3662 |
| P14625 | Endoplasmin OS=Homo sapiens OX=9606 GN=HSP90B1 PE=1 SV=1 | HSP90B1 | 92.468 | 0.67 | 0.0904 | 0.86 | 0.4323 | 0.78 | 0.1855 |
| P27487 | Dipeptidyl peptidase 4 OS=Homo sapiens OX=9606 GN=DPP4 PE=1 SV=2 | DPP4 | 88.278 | 2.19 | 0.3141 | 1.98 | 0.1468 | 1.11 | 0.7337 |
| P51884 | Lumican OS=Homo sapiens OX=9606 GN=LUM PE=1 SV=2 | LUM | 38.429 | 1.10 | 0.4991 | 1.01 | 0.8361 | 1.10 | 0.7682 |
| Q9BVK6 | Transmembrane emp24 domain-containing protein 9 OS=Homo sapiens OX=9606 GN=TMED9 PE=1 SV=2 | TMED9 | 27.277 | 0.95 | 0.6594 | 1.12 | 0.4723 | 0.85 | 0.3113 |
| Q86X52 | Chondroitin sulfate synthase 1 OS=Homo sapiens OX=9606 GN=CHSY1 PE=1 SV=3 | CHSY1 | 91.783 | 1.54 | 0.0440 | 1.31 | 0.0464 | 1.18 | 0.2926 |
| Q15907 | Ras-related protein Rab-11B OS=Homo sapiens OX=9606 GN=RAB11B PE=1 SV=4 | RAB11B | 24.488 | 0.74 | 0.0673 | 0.75 | 0.0997 | 0.98 | 0.8161 |
| Q9Y4G8 | Rap guanine nucleotide exchange factor 2 OS=Homo sapiens OX=9606 GN=RAPGEF2 PE=1 SV=1 | RAPGEF2 | 167.41 | 1.52 | 0.2251 | 1.20 | 0.3063 | 1.27 | 0.1498 |
| P61160 | Actin-related protein 2 OS=Homo sapiens OX=9606 GN=ACTR2 PE=1 SV=1 | ACTR2 | 44.76 | 0.69 | 0.1238 | 0.80 | 0.3383 | 0.86 | 0.3375 |
| Q15773 | Myeloid leukemia factor 2 OS=Homo sapiens OX=9606 GN=MLF2 PE=1 SV=1 | MLF2 | 28.147 | 0.12 | 0.4682 | 0.90 | 0.5683 | 0.13 | 0.4906 |
| P0DJI8 | Serum amyloid A-1 protein OS=Homo sapiens OX=9606 GN=SAA1 PE=1 SV=1 | SAA1 | 13.532 | 2.97 | 0.0524 | 2.79 | 0.0685 | 1.07 | 0.5045 |
| P08603 | Complement factor H OS=Homo sapiens OX=9606 GN=CFH PE=1 SV=4 | CFH | 139.09 | 0.91 | 0.1373 | 1.16 | 0.0771 | 0.79 | 0.0141 |
| P62937 | Peptidyl-prolyl cis-trans isomerase A OS=Homo sapiens OX=9606 GN=PPIA PE=1 SV=2 | PPIA | 18.012 | 0.66 | 0.0483 | 0.79 | 0.2421 | 0.84 | 0.2283 |
| P04278 | Sex hormone-binding globulin OS=Homo sapiens OX=9606 GN=SHBG PE=1 SV=2 | SHBG | 43.779 | 1.25 | 0.2865 | 1.23 | 0.1873 | 1.01 | 0.8780 |
| Q15833 | Syntaxin-binding protein 2 OS=Homo sapiens OX=9606 GN=STXBP2 PE=1 SV=2 | STXBP2 | 66.452 | 0.86 | 0.3800 | 0.74 | 0.2280 | 1.16 | 0.7750 |
| Q9GZT6 | Coiled-coil domain-containing protein 90B, mitochondrial OS=Homo sapiens OX=9606 GN=CCDC90B PE=1 SV=2 | CCDC90B | 29.505 | 0.42 | 0.1877 | 0.71 | 0.8062 | 0.60 | 0.2512 |
| P02786 | Transferrin receptor protein 1 OS=Homo sapiens OX=9606 GN=TFRC PE=1 SV=2 | TFRC | 84.87 | 1.16 | 0.2886 | 1.10 | 0.4884 | 1.06 | 0.3976 |
| P30825 | High affinity cationic amino acid transporter 1 OS=Homo sapiens OX=9606 GN=SLC7A1 PE=1 SV=1 | SLC7A1 | 67.638 | 1.62 | 0.1982 | 1.00 | 0.9455 | 1.62 | 0.1195 |
| Q27J81 | Inverted formin-2 OS=Homo sapiens OX=9606 GN=INF2 PE=1 SV=2 | INF2 | 135.62 | 0.66 | 0.0900 | 0.73 | 0.3262 | 0.90 | 0.5224 |
| P0C0L4 | Complement C4-A OS=Homo sapiens OX=9606 GN=C4A PE=1 SV=2 | C4A | 192.78 | 1.21 | 0.0056 | 1.22 | 0.0199 | 0.99 | 0.7516 |
| A0A075B6K4 | Immunoglobulin lambda variable 3-10 OS=Homo sapiens OX=9606 GN=IGLV3-10 PE=3 SV=2 | IGLV3-10 | 12.441 | 0.42 | 0.0478 | 0.80 | 0.4633 | 0.53 | 0.0548 |
| P24821 | Tenascin OS=Homo sapiens OX=9606 GN=TNC PE=1 SV=3 | TNC | 240.85 | 1.29 | 0.1755 | 1.13 | 0.3687 | 1.14 | 0.2764 |
| P04180 | Phosphatidylcholine-sterol acyltransferase OS=Homo sapiens OX=9606 GN=LCAT PE=1 SV=1 | LCAT | 49.577 | 1.07 | 0.6799 | 1.08 | 0.5398 | 0.99 | 0.9940 |
| Q86UW7 | Calcium-dependent secretion activator 2 OS=Homo sapiens OX=9606 GN=CADPS2 PE=1 SV=2 | CADPS2 | 147.73 | 1.35 | 0.0056 | 1.01 | 0.5607 | 1.33 | 0.3296 |
| Q9UBQ6 | Exostosin-like 2 OS=Homo sapiens OX=9606 GN=EXTL2 PE=1 SV=1 | EXTL2 | 37.465 | 0.84 | 0.7189 | 0.30 | 0.2517 | 2.79 | 0.3428 |
| Q9UPN3 | Microtubule-actin cross-linking factor 1, isoforms 1/2/3/5 OS=Homo sapiens OX=9606 GN=MACF1 PE=1 SV=4 | MACF1 | 838.3 | 0.73 | 0.1838 | 1.32 | 0.1569 | 0.55 | 0.0210 |
| Q13185 | Chromobox protein homolog 3 OS=Homo sapiens OX=9606 GN=CBX3 PE=1 SV=4 | CBX3 | 20.811 |  |  |  |  |  |  |
| Q14644 | Ras GTPase-activating protein 3 OS=Homo sapiens OX=9606 GN=RASA3 PE=1 SV=3 | RASA3 | 95.698 | 0.60 | 0.0613 | 0.67 | 0.0736 | 0.89 | 0.5088 |
| Q5T749 | Keratinocyte proline-rich protein OS=Homo sapiens OX=9606 GN=KPRP PE=1 SV=1 | KPRP | 64.135 | 1.77 | 0.1737 | 1.11 | 0.8981 | 1.60 | 0.0082 |
| P55103 | Inhibin beta C chain OS=Homo sapiens OX=9606 GN=INHBC PE=2 SV=1 | INHBC | 38.237 | 1.13 | 0.3884 | 1.13 | 0.2856 | 1.01 | 0.9912 |
| P01602 | Immunoglobulin kappa variable 1-5 OS=Homo sapiens OX=9606 GN=IGKV1-5 PE=1 SV=2 | IGKV1-5 | 12.781 | 0.38 | 0.0024 | 0.61 | 0.0296 | 0.62 | 0.0416 |
| P04003 | C4b-binding protein alpha chain OS=Homo sapiens OX=9606 GN=C4BPA PE=1 SV=2 | C4BPA | 67.033 | 1.52 | 0.0004 | 1.30 | 0.0105 | 1.17 | 0.0012 |
| P03989 | HLA class I histocompatibility antigen, B-27 alpha chain OS=Homo sapiens OX=9606 GN=HLA-B PE=1 SV=2 | HLA-B | 40.428 |  |  |  |  |  |  |
| A2NJV5 | Immunoglobulin kappa variable 2-29 OS=Homo sapiens OX=9606 GN=IGKV2-29 PE=3 SV=2 | IGKV2-29 | 13.085 | 1.00 | 0.9861 | 1.00 | 0.7528 | 1.00 | 0.7558 |
| A0A0B4J1U7 | Immunoglobulin heavy variable 6-1 OS=Homo sapiens OX=9606 GN=IGHV6-1 PE=3 SV=1 | IGHV6-1 | 13.481 | 0.86 | 0.5461 | 0.95 | 0.6885 | 0.90 | 0.6345 |
| P78356 | Phosphatidylinositol 5-phosphate 4-kinase type-2 beta OS=Homo sapiens OX=9606 GN=PIP4K2B PE=1 SV=1 | PIP4K2B | 47.377 | 0.52 | 0.1275 | 0.62 | 0.0758 | 0.84 | 0.4580 |
| Q8N6Q3 | CD177 antigen OS=Homo sapiens OX=9606 GN=CD177 PE=1 SV=2 | CD177 | 46.363 | 0.96 | 0.6997 | 0.91 | 0.7867 | 1.05 | 0.9326 |
| Q92576 | PHD finger protein 3 OS=Homo sapiens OX=9606 GN=PHF3 PE=1 SV=3 | PHF3 | 229.48 |  |  |  |  |  |  |
| P0C0L5 | Complement C4-B OS=Homo sapiens OX=9606 GN=C4B PE=1 SV=2 | C4B | 192.75 | 1.26 | 0.0740 | 1.19 | 0.0538 | 1.06 | 0.5185 |
| P21810 | Biglycan OS=Homo sapiens OX=9606 GN=BGN PE=1 SV=2 | BGN | 41.654 | 1.24 | 0.5089 | 1.25 | 0.5107 | 0.99 | 0.9867 |
| P62873 | Guanine nucleotide-binding protein G(I)/G(S)/G(T) subunit beta-1 OS=Homo sapiens OX=9606 GN=GNB1 PE=1 SV=3 | GNB1 | 37.377 | 0.98 | 0.8030 | 1.00 | 0.9121 | 0.98 | 0.7405 |
| P07359 | Platelet glycoprotein Ib alpha chain OS=Homo sapiens OX=9606 GN=GP1BA PE=1 SV=2 | GP1BA | 71.539 | 0.81 | 0.2355 | 0.69 | 0.1605 | 1.18 | 0.7229 |
| P61019 | Ras-related protein Rab-2A OS=Homo sapiens OX=9606 GN=RAB2A PE=1 SV=1 | RAB2A | 23.545 | 0.80 | 0.5723 | 0.76 | 0.2318 | 1.05 | 0.8059 |
| P02750 | Leucine-rich alpha-2-glycoprotein OS=Homo sapiens OX=9606 GN=LRG1 PE=1 SV=2 | LRG1 | 38.177 | 0.93 | 0.0477 | 0.53 | 0.1365 | 1.75 | 0.1924 |
| Q9Y6K1 | DNA (cytosine-5)-methyltransferase 3A OS=Homo sapiens OX=9606 GN=DNMT3A PE=1 SV=4 | DNMT3A | 101.86 |  |  |  |  |  |  |
| Q4G0X9 | Coiled-coil domain-containing protein 40 OS=Homo sapiens OX=9606 GN=CCDC40 PE=2 SV=2 | CCDC40 | 130.11 | 0.99 | 0.8308 | 0.88 | 0.5409 | 1.12 | 0.6937 |
| Q14061 | Cytochrome c oxidase copper chaperone OS=Homo sapiens OX=9606 GN=COX17 PE=1 SV=2 | COX17 | 6.9151 | 0.84 | 0.2509 | 1.21 | 0.2591 | 0.70 | 0.0582 |
| O15335 | Chondroadherin OS=Homo sapiens OX=9606 GN=CHAD PE=1 SV=2 | CHAD | 40.475 | 1.22 | 0.9791 | 0.78 | 0.6571 | 1.57 | 0.6386 |
| P02652 | Apolipoprotein A-II OS=Homo sapiens OX=9606 GN=APOA2 PE=1 SV=1 | APOA2 | 11.175 | 0.89 | 0.2479 | 1.00 | 0.9205 | 0.89 | 0.2010 |
| P08476 | Inhibin beta A chain OS=Homo sapiens OX=9606 GN=INHBA PE=1 SV=2 | INHBA | 47.442 |  |  |  |  |  |  |
| Q9ULD9 | Zinc finger protein 608 OS=Homo sapiens OX=9606 GN=ZNF608 PE=1 SV=4 | ZNF608 | 162.21 | 0.32 | 0.0087 | 0.83 | 0.1947 | 0.39 | 0.0009 |
| P05090 | Apolipoprotein D OS=Homo sapiens OX=9606 GN=APOD PE=1 SV=1 | APOD | 21.275 | 1.08 | 0.5423 | 1.04 | 0.6210 | 1.03 | 0.8515 |
| Q8IZM9 | Probable sodium-coupled neutral amino acid transporter 6 OS=Homo sapiens OX=9606 GN=SLC38A6 PE=1 SV=2 | SLC38A6 | 50.928 | 1.46 | 0.3542 | 1.20 | 0.1292 | 1.22 | 0.3864 |
| Q6ZS30 | Neurobeachin-like protein 1 OS=Homo sapiens OX=9606 GN=NBEAL1 PE=2 SV=3 | NBEAL1 | 307.23 | 0.12 | 0.0135 | 1.00 | 0.9316 | 0.12 | 0.0043 |
| P48061 | Stromal cell-derived factor 1 OS=Homo sapiens OX=9606 GN=CXCL12 PE=1 SV=1 | CXCL12 | 10.666 | 0.61 | 0.1423 | 0.97 | 0.8706 | 0.63 | 0.0878 |
| Q15120 | [Pyruvate dehydrogenase (acetyl-transferring)] kinase isozyme 3, mitochondrial OS=Homo sapiens OX=9606 GN=PDK3 PE=1 SV=1 | PDK3 | 46.938 | 2.00 | 0.1396 | 1.31 | 0.1315 | 1.53 | 0.0214 |
| P19320 | Vascular cell adhesion protein 1 OS=Homo sapiens OX=9606 GN=VCAM1 PE=1 SV=1 | VCAM1 | 81.275 | 0.99 | 0.6955 | 0.80 | 0.1171 | 1.23 | 0.2289 |
| Q9UBT6 | DNA polymerase kappa OS=Homo sapiens OX=9606 GN=POLK PE=1 SV=1 | POLK | 98.808 | 1.14 | 0.3881 | 1.08 | 0.5593 | 1.06 | 0.8020 |
| Q9Y624 | Junctional adhesion molecule A OS=Homo sapiens OX=9606 GN=F11R PE=1 SV=1 | F11R | 32.583 | 0.83 | 0.2659 | 0.78 | 0.3444 | 1.07 | 0.9873 |
| Q9Y490 | Talin-1 OS=Homo sapiens OX=9606 GN=TLN1 PE=1 SV=3 | TLN1 | 269.76 | 0.58 | 0.0191 | 0.76 | 0.1736 | 0.76 | 0.1247 |
| Q9C0B0 | RING finger protein unkempt homolog OS=Homo sapiens OX=9606 GN=UNK PE=1 SV=2 | UNK | 88.084 | 1.21 | 0.6675 | 0.84 | 0.9952 | 1.44 | 0.6447 |
| P01701 | Immunoglobulin lambda variable 1-51 OS=Homo sapiens OX=9606 GN=IGLV1-51 PE=1 SV=2 | IGLV1-51 | 12.249 | 0.86 | 0.4395 | 1.10 | 0.4940 | 0.79 | 0.1030 |
| Q13103 | Secreted phosphoprotein 24 OS=Homo sapiens OX=9606 GN=SPP2 PE=1 SV=1 | SPP2 | 24.337 | 1.20 | 0.5003 | 0.98 | 0.9347 | 1.22 | 0.5748 |
| P00491 | Purine nucleoside phosphorylase OS=Homo sapiens OX=9606 GN=PNP PE=1 SV=2 | PNP | 32.118 | 1.10 | 0.7962 | 0.77 | 0.7498 | 1.43 | 0.9614 |
| Q8NG08 | DNA helicase B OS=Homo sapiens OX=9606 GN=HELB PE=1 SV=2 | HELB | 123.25 | 1.24 | 0.4622 | 0.88 | 0.4804 | 1.42 | 0.0949 |
| Q8NHH9 | Atlastin-2 OS=Homo sapiens OX=9606 GN=ATL2 PE=1 SV=2 | ATL2 | 66.228 |  |  |  |  |  |  |
| Q96CX2 | BTB/POZ domain-containing protein KCTD12 OS=Homo sapiens OX=9606 GN=KCTD12 PE=1 SV=1 | KCTD12 | 35.7 | 0.69 | 0.4086 | 0.76 | 0.2555 | 0.92 | 0.9496 |
| P13591 | Neural cell adhesion molecule 1 OS=Homo sapiens OX=9606 GN=NCAM1 PE=1 SV=3 | NCAM1 | 94.573 | 0.81 | 0.3010 | 0.61 | 0.0952 | 1.32 | 0.4094 |
| Q13201 | Multimerin-1 OS=Homo sapiens OX=9606 GN=MMRN1 PE=1 SV=3 | MMRN1 | 138.11 | 1.19 | 0.2736 | 1.27 | 0.0414 | 0.93 | 0.6333 |
| Q15058 | Kinesin-like protein KIF14 OS=Homo sapiens OX=9606 GN=KIF14 PE=1 SV=1 | KIF14 | 186.49 | 0.76 | 0.2296 | 1.06 | 0.7515 | 0.71 | 0.1441 |
| P0DP09 | Immunoglobulin kappa variable 1-13 OS=Homo sapiens OX=9606 GN=IGKV1-13 PE=3 SV=1 | IGKV1-13 | 12.569 | 1.04 | 0.7396 | 0.95 | 0.6935 | 1.10 | 0.4388 |
| P01704 | Immunoglobulin lambda variable 2-14 OS=Homo sapiens OX=9606 GN=IGLV2-14 PE=1 SV=2 | IGLV2-14 | 12.597 | 0.88 | 0.7765 | 0.73 | 0.4389 | 1.20 | 0.3584 |
| P0DPD5 | Zinc finger protein 723 OS=Homo sapiens OX=9606 GN=ZNF723 PE=3 SV=1 | ZNF723 | 59.151 |  |  |  |  |  |  |
| O15524 | Suppressor of cytokine signaling 1 OS=Homo sapiens OX=9606 GN=SOCS1 PE=1 SV=1 | SOCS1 | 23.551 | 1.42 | 0.4765 | 1.12 | 0.7710 | 1.27 | 0.4307 |
| P42345 | Serine/threonine-protein kinase mTOR OS=Homo sapiens OX=9606 GN=MTOR PE=1 SV=1 | MTOR | 288.89 | 1.69 | 0.5483 | 1.01 | 0.9902 | 1.68 | 0.3624 |
| P19397 | Leukocyte surface antigen CD53 OS=Homo sapiens OX=9606 GN=CD53 PE=1 SV=1 | CD53 | 24.341 |  |  |  |  |  |  |
| P09871 | Complement C1s subcomponent OS=Homo sapiens OX=9606 GN=C1S PE=1 SV=1 | C1S | 76.684 | 0.99 | 0.8049 | 0.93 | 0.2255 | 1.07 | 0.3334 |
| P0DOX3 | Immunoglobulin delta heavy chain OS=Homo sapiens OX=9606 PE=1 SV=1 | --- | 56.224 | 0.61 | 0.0136 | 0.78 | 0.0670 | 0.78 | 0.1241 |
| P05154 | Plasma serine protease inhibitor OS=Homo sapiens OX=9606 GN=SERPINA5 PE=1 SV=3 | SERPINA5 | 45.674 | 1.04 | 0.6625 | 0.82 | 0.0050 | 1.26 | 0.0007 |
| P09525 | Annexin A4 OS=Homo sapiens OX=9606 GN=ANXA4 PE=1 SV=4 | ANXA4 | 35.882 | 1.01 | 0.6278 | 0.77 | 0.1554 | 1.31 | 0.2971 |
| Q8NCM2 | Potassium voltage-gated channel subfamily H member 5 OS=Homo sapiens OX=9606 GN=KCNH5 PE=1 SV=3 | KCNH5 | 111.88 |  |  |  |  |  |  |
| P0DP02 | Immunoglobulin heavy variable 3-30-3 OS=Homo sapiens OX=9606 GN=IGHV3-30-3 PE=3 SV=1 | IGHV3-30-3 | 12.989 |  |  |  |  |  |  |
| P42892 | Endothelin-converting enzyme 1 OS=Homo sapiens OX=9606 GN=ECE1 PE=1 SV=2 | ECE1 | 87.163 | 1.34 | 0.5419 | 0.96 | 0.7985 | 1.40 | 0.2524 |
| P35555 | Fibrillin-1 OS=Homo sapiens OX=9606 GN=FBN1 PE=1 SV=3 | FBN1 | 312.24 | 0.96 | 0.8566 | 0.95 | 0.6392 | 1.00 | 0.8861 |
| O60613 | Selenoprotein F OS=Homo sapiens OX=9606 GN=SELENOF PE=1 SV=4 | SELENOF | 18.092 |  |  |  |  |  |  |
| P16671 | Platelet glycoprotein 4 OS=Homo sapiens OX=9606 GN=CD36 PE=1 SV=2 | CD36 | 53.053 | 0.73 | 0.0708 | 0.98 | 0.9751 | 0.75 | 0.0526 |
| Q9UEU0 | Vesicle transport through interaction with t-SNAREs homolog 1B OS=Homo sapiens OX=9606 GN=VTI1B PE=1 SV=3 | VTI1B | 26.688 | 2.29 | 0.1124 | 1.55 | 0.2125 | 1.48 | 0.4212 |
| Q99880 | Histone H2B type 1-L OS=Homo sapiens OX=9606 GN=HIST1H2BL PE=1 SV=3 | HIST1H2BL | 13.952 | 0.67 | 0.1723 | 1.10 | 0.7301 | 0.61 | 0.0342 |
| P81605 | Dermcidin OS=Homo sapiens OX=9606 GN=DCD PE=1 SV=2 | DCD | 11.284 | 1.50 | 0.0906 | 1.08 | 0.6882 | 1.39 | 0.0918 |
| P59544 | Taste receptor type 2 member 50 OS=Homo sapiens OX=9606 GN=TAS2R50 PE=2 SV=2 | TAS2R50 | 34.557 | 0.21 | 0.1276 | 0.42 | 0.1305 | 0.50 | 0.1413 |
| P11597 | Cholesteryl ester transfer protein OS=Homo sapiens OX=9606 GN=CETP PE=1 SV=2 | CETP | 54.756 | 1.15 | 0.5303 | 1.46 | 0.0510 | 0.79 | 0.0540 |
| P48509 | CD151 antigen OS=Homo sapiens OX=9606 GN=CD151 PE=1 SV=3 | CD151 | 28.295 | 0.95 | 0.6248 | 0.86 | 0.3180 | 1.11 | 0.6397 |
| P53801 | Pituitary tumor-transforming gene 1 protein-interacting protein OS=Homo sapiens OX=9606 GN=PTTG1IP PE=1 SV=1 | PTTG1IP | 20.324 | 0.73 | 0.1521 | 0.70 | 0.2345 | 1.04 | 0.9976 |
| P19827 | Inter-alpha-trypsin inhibitor heavy chain H1 OS=Homo sapiens OX=9606 GN=ITIH1 PE=1 SV=3 | ITIH1 | 101.39 | 1.12 | 0.3738 | 1.04 | 0.6067 | 1.07 | 0.5757 |
| Q14019 | Coactosin-like protein OS=Homo sapiens OX=9606 GN=COTL1 PE=1 SV=3 | COTL1 | 15.945 | 0.78 | 0.2364 | 0.69 | 0.1719 | 1.13 | 0.9303 |
| A0A0C4DH72 | Immunoglobulin kappa variable 1-6 OS=Homo sapiens OX=9606 GN=IGKV1-6 PE=3 SV=1 | IGKV1-6 | 12.697 |  |  |  |  |  |  |
| P01619 | Immunoglobulin kappa variable 3-20 OS=Homo sapiens OX=9606 GN=IGKV3-20 PE=1 SV=2 | IGKV3-20 | 12.557 | 0.28 | 0.1220 | 0.53 | 0.2622 | 0.52 | 0.1569 |
| O43734 | Adapter protein CIKS OS=Homo sapiens OX=9606 GN=TRAF3IP2 PE=1 SV=3 | TRAF3IP2 | 64.665 | 1.64 | 0.3282 | 1.14 | 0.7606 | 1.44 | 0.1018 |
| P48426 | Phosphatidylinositol 5-phosphate 4-kinase type-2 alpha OS=Homo sapiens OX=9606 GN=PIP4K2A PE=1 SV=2 | PIP4K2A | 46.224 | 0.83 | 0.5182 | 0.73 | 0.4180 | 1.15 | 0.8958 |
| P68871 | Hemoglobin subunit beta OS=Homo sapiens OX=9606 GN=HBB PE=1 SV=2 | HBB | 15.998 | 0.64 | 0.0696 | 0.80 | 0.1381 | 0.80 | 0.2082 |
| Q9UPN9 | E3 ubiquitin-protein ligase TRIM33 OS=Homo sapiens OX=9606 GN=TRIM33 PE=1 SV=3 | TRIM33 | 122.53 | 1.88 | 0.0015 | 1.31 | 0.0589 | 1.43 | 0.0763 |
| P58546 | Myotrophin OS=Homo sapiens OX=9606 GN=MTPN PE=1 SV=2 | MTPN | 12.895 | 0.58 | 0.0169 | 0.59 | 0.1232 | 0.97 | 0.7835 |
| P01706 | Immunoglobulin lambda variable 2-11 OS=Homo sapiens OX=9606 GN=IGLV2-11 PE=1 SV=2 | IGLV2-11 | 12.644 | 1.55 | 0.2884 | 1.68 | 0.0934 | 0.92 | 0.9641 |
| O15400 | Syntaxin-7 OS=Homo sapiens OX=9606 GN=STX7 PE=1 SV=4 | STX7 | 29.815 | 1.04 | 0.4412 | 0.84 | 0.3329 | 1.24 | 0.4202 |
| P02790 | Hemopexin OS=Homo sapiens OX=9606 GN=HPX PE=1 SV=2 | HPX | 51.676 | 1.10 | 0.4300 | 0.82 | 0.2979 | 1.35 | 0.0257 |
| A0A0C4DH24 | Immunoglobulin kappa variable 6-21 OS=Homo sapiens OX=9606 GN=IGKV6-21 PE=3 SV=1 | IGKV6-21 | 12.43 | 0.58 | 0.2208 | 0.67 | 0.1927 | 0.86 | 0.7033 |
| P01718 | Immunoglobulin lambda variable 3-27 OS=Homo sapiens OX=9606 GN=IGLV3-27 PE=1 SV=2 | IGLV3-27 | 12.165 | 1.02 | 0.9100 | 0.77 | 0.5547 | 1.33 | 0.4363 |
| P31947 | 14-3-3 protein sigma OS=Homo sapiens OX=9606 GN=SFN PE=1 SV=1 | SFN | 27.774 | 0.69 | 0.0669 | 0.89 | 0.6755 | 0.78 | 0.1333 |
| Q9ULI3 | Protein HEG homolog 1 OS=Homo sapiens OX=9606 GN=HEG1 PE=1 SV=3 | HEG1 | 147.46 | 1.32 | 0.0289 | 1.36 | 0.0324 | 0.97 | 0.7313 |
| P04632 | Calpain small subunit 1 OS=Homo sapiens OX=9606 GN=CAPNS1 PE=1 SV=1 | CAPNS1 | 28.315 | 0.86 | 0.3246 | 0.72 | 0.2562 | 1.18 | 0.6939 |
| P19105 | Myosin regulatory light chain 12A OS=Homo sapiens OX=9606 GN=MYL12A PE=1 SV=2 | MYL12A | 19.794 | 0.82 | 0.2268 | 0.77 | 0.1531 | 1.07 | 0.8133 |
| Q6TDU7 | Protein CASC1 OS=Homo sapiens OX=9606 GN=CASC1 PE=2 SV=2 | CASC1 | 83.159 | 3.31 | 0.2702 | 1.28 | 0.7341 | 2.60 | 0.0596 |
| A0A087WSX0 | Immunoglobulin lambda variable 5-45 OS=Homo sapiens OX=9606 GN=IGLV5-45 PE=3 SV=1 | IGLV5-45 | 13.162 | 0.69 | 0.0363 | 1.12 | 0.4871 | 0.61 | 0.1631 |
| P30043 | Flavin reductase (NADPH) OS=Homo sapiens OX=9606 GN=BLVRB PE=1 SV=3 | BLVRB | 22.119 | 1.15 | 0.0019 | 0.99 | 0.9280 | 1.16 | 0.0089 |
| Q9NVA2 | Septin-11 OS=Homo sapiens OX=9606 GN=SEPT11 PE=1 SV=3 | SEPT11 | 49.398 | 0.89 | 0.5228 | 0.79 | 0.3761 | 1.12 | 0.7797 |
| Q96JM2 | Zinc finger protein 462 OS=Homo sapiens OX=9606 GN=ZNF462 PE=1 SV=3 | ZNF462 | 284.68 | 1.60 | 0.4220 | 1.48 | 0.3460 | 1.08 | 0.8645 |
| Q96PD5 | N-acetylmuramoyl-L-alanine amidase OS=Homo sapiens OX=9606 GN=PGLYRP2 PE=1 SV=1 | PGLYRP2 | 62.216 | 0.90 | 0.4532 | 0.89 | 0.3070 | 1.01 | 0.9034 |
| P78509 | Reelin OS=Homo sapiens OX=9606 GN=RELN PE=1 SV=3 | RELN | 388.38 | 1.19 | 0.2608 | 1.14 | 0.3402 | 1.04 | 0.5117 |
| Q9UHG3 | Prenylcysteine oxidase 1 OS=Homo sapiens OX=9606 GN=PCYOX1 PE=1 SV=3 | PCYOX1 | 56.639 | 1.03 | 0.9004 | 1.00 | 0.8310 | 1.03 | 0.6983 |
| P00738 | Haptoglobin OS=Homo sapiens OX=9606 GN=HP PE=1 SV=1 | HP | 45.205 | 1.25 | 0.1112 | 1.17 | 0.1806 | 1.07 | 0.7460 |
| P78417 | Glutathione S-transferase omega-1 OS=Homo sapiens OX=9606 GN=GSTO1 PE=1 SV=2 | GSTO1 | 27.566 | 0.64 | 0.1356 | 0.73 | 0.2402 | 0.87 | 0.4165 |
| Q96F63 | Coiled-coil domain-containing protein 97 OS=Homo sapiens OX=9606 GN=CCDC97 PE=1 SV=1 | CCDC97 | 38.946 | 1.25 | 0.2094 | 0.42 | 0.5554 | 3.01 | 0.3705 |
| Q13642 | Four and a half LIM domains protein 1 OS=Homo sapiens OX=9606 GN=FHL1 PE=1 SV=4 | FHL1 | 36.263 | 0.77 | 0.3849 | 0.80 | 0.6319 | 0.96 | 0.7127 |
| P00742 | Coagulation factor X OS=Homo sapiens OX=9606 GN=F10 PE=1 SV=2 | F10 | 54.731 | 0.97 | 0.7689 | 1.03 | 0.7887 | 0.94 | 0.4422 |
| Q9HCM1 | Uncharacterized protein KIAA1551 OS=Homo sapiens OX=9606 GN=KIAA1551 PE=1 SV=3 | KIAA1551 | 194.86 | 1.10 | 0.8762 | 0.29 | 0.5730 | 3.77 | 0.6125 |
| Q13576 | Ras GTPase-activating-like protein IQGAP2 OS=Homo sapiens OX=9606 GN=IQGAP2 PE=1 SV=4 | IQGAP2 | 180.58 | 0.57 | 0.0310 | 0.61 | 0.1474 | 0.92 | 0.6241 |
| B2RUZ4 | Small integral membrane protein 1 OS=Homo sapiens OX=9606 GN=SMIM1 PE=1 SV=1 | SMIM1 | 8.749 | 1.09 | 0.5496 | 0.82 | 0.1365 | 1.34 | 0.0777 |
| P10412 | Histone H1.4 OS=Homo sapiens OX=9606 GN=HIST1H1E PE=1 SV=2 | HIST1H1E | 21.865 | 1.19 | 0.3969 | 0.71 | 0.3998 | 1.69 | 0.1666 |
| P07384 | Calpain-1 catalytic subunit OS=Homo sapiens OX=9606 GN=CAPN1 PE=1 SV=1 | CAPN1 | 81.889 | 0.66 | 0.1049 | 0.77 | 0.3278 | 0.85 | 0.3899 |
| Q9BZW5 | Transmembrane 6 superfamily member 1 OS=Homo sapiens OX=9606 GN=TM6SF1 PE=1 SV=2 | TM6SF1 | 41.636 | 1.76 | 0.1254 | 1.14 | 0.4315 | 1.54 | 0.0111 |
| P21333 | Filamin-A OS=Homo sapiens OX=9606 GN=FLNA PE=1 SV=4 | FLNA | 280.74 | 0.59 | 0.0116 | 0.78 | 0.2363 | 0.75 | 0.0762 |
| P62805 | Histone H4 OS=Homo sapiens OX=9606 GN=HIST1H4A PE=1 SV=2 | HIST1H4A | 11.367 | 0.90 | 0.6562 | 1.43 | 0.1844 | 0.63 | 0.0588 |
| P50148 | Guanine nucleotide-binding protein G(q) subunit alpha OS=Homo sapiens OX=9606 GN=GNAQ PE=1 SV=4 | GNAQ | 42.142 | 0.76 | 0.0458 | 0.76 | 0.2009 | 0.99 | 0.7724 |
| P05106 | Integrin beta-3 OS=Homo sapiens OX=9606 GN=ITGB3 PE=1 SV=2 | ITGB3 | 87.057 | 0.64 | 0.0584 | 0.75 | 0.2871 | 0.86 | 0.3655 |
| Q15113 | Procollagen C-endopeptidase enhancer 1 OS=Homo sapiens OX=9606 GN=PCOLCE PE=1 SV=2 | PCOLCE | 47.972 | 1.00 | 0.8039 | 0.77 | 0.1761 | 1.30 | 0.0838 |
| P08575 | Receptor-type tyrosine-protein phosphatase C OS=Homo sapiens OX=9606 GN=PTPRC PE=1 SV=3 | PTPRC | 147.48 | 0.92 | 0.5009 | 0.97 | 0.7384 | 0.95 | 0.6103 |
| P08697 | Alpha-2-antiplasmin OS=Homo sapiens OX=9606 GN=SERPINF2 PE=1 SV=3 | SERPINF2 | 54.565 | 1.96 | 0.0011 | 1.06 | 0.6097 | 1.84 | 0.0029 |
| P13671 | Complement component C6 OS=Homo sapiens OX=9606 GN=C6 PE=1 SV=3 | C6 | 104.79 | 1.69 | 0.0665 | 1.34 | 0.1357 | 1.25 | 0.4285 |
| P30492 | HLA class I histocompatibility antigen, B-54 alpha chain OS=Homo sapiens OX=9606 GN=HLA-B PE=1 SV=1 | HLA-B | 40.38 |  |  |  |  |  |  |
| P59666 | Neutrophil defensin 3 OS=Homo sapiens OX=9606 GN=DEFA3 PE=1 SV=1 | DEFA3 | 10.245 | 0.99 | 0.9585 | 1.18 | 0.8903 | 0.84 | 0.8782 |
| P48643 | T-complex protein 1 subunit epsilon OS=Homo sapiens OX=9606 GN=CCT5 PE=1 SV=1 | CCT5 | 59.67 | 0.84 | 0.1185 | 0.82 | 0.2113 | 1.03 | 0.9559 |
| Q8IUR6 | CREB3 regulatory factor OS=Homo sapiens OX=9606 GN=CREBRF PE=1 SV=2 | CREBRF | 72.148 | 1.10 | 0.8712 | 0.87 | 0.5270 | 1.27 | 0.3122 |
| Q02413 | Desmoglein-1 OS=Homo sapiens OX=9606 GN=DSG1 PE=1 SV=2 | DSG1 | 113.75 | 1.46 | 0.1982 | 0.99 | 0.6057 | 1.47 | 0.0067 |
| Q92930 | Ras-related protein Rab-8B OS=Homo sapiens OX=9606 GN=RAB8B PE=1 SV=2 | RAB8B | 23.584 | 0.93 | 0.5933 | 0.77 | 0.1459 | 1.21 | 0.3459 |
| Q9Y566 | SH3 and multiple ankyrin repeat domains protein 1 OS=Homo sapiens OX=9606 GN=SHANK1 PE=1 SV=2 | SHANK1 | 224.96 | 1.64 | 0.4133 | 1.29 | 0.5216 | 1.27 | 0.7881 |
| O60667 | Fas apoptotic inhibitory molecule 3 OS=Homo sapiens OX=9606 GN=FCMR PE=1 SV=1 | FCMR | 43.146 | 0.89 | 0.3577 | 0.70 | 0.0612 | 1.28 | 0.4442 |
| P49755 | Transmembrane emp24 domain-containing protein 10 OS=Homo sapiens OX=9606 GN=TMED10 PE=1 SV=2 | TMED10 | 24.976 | 0.96 | 0.8304 | 1.07 | 0.7760 | 0.90 | 0.2921 |
| P35579 | Myosin-9 OS=Homo sapiens OX=9606 GN=MYH9 PE=1 SV=4 | MYH9 | 226.53 | 0.63 | 0.0432 | 0.79 | 0.1368 | 0.79 | 0.1920 |
| Q9NTJ5 | Phosphatidylinositide phosphatase SAC1 OS=Homo sapiens OX=9606 GN=SACM1L PE=1 SV=2 | SACM1L | 66.966 |  |  |  |  |  |  |
| Q08830 | Fibrinogen-like protein 1 OS=Homo sapiens OX=9606 GN=FGL1 PE=1 SV=3 | FGL1 | 36.379 | 2.83 | 0.0008 | 2.08 | 0.0003 | 1.36 | 0.0611 |
| A0M8Q6 | Immunoglobulin lambda constant 7 OS=Homo sapiens OX=9606 GN=IGLC7 PE=1 SV=3 | IGLC7 | 11.253 | 0.94 | 0.7734 | 1.12 | 0.5782 | 0.84 | 0.3685 |
| P17600 | Synapsin-1 OS=Homo sapiens OX=9606 GN=SYN1 PE=1 SV=3 | SYN1 | 74.111 | 1.07 | 0.4788 | 1.11 | 0.2429 | 0.96 | 0.6374 |
| Q8TBF8 | Protein FAM81A OS=Homo sapiens OX=9606 GN=FAM81A PE=2 SV=3 | FAM81A | 42.391 | 0.63 | 0.3006 | 0.80 | 0.3338 | 0.79 | 0.4686 |
| A0A0B4J1Y9 | Immunoglobulin heavy variable 3-72 OS=Homo sapiens OX=9606 GN=IGHV3-72 PE=3 SV=1 | IGHV3-72 | 13.203 | 0.71 | 0.0896 | 0.98 | 0.6481 | 0.72 | 0.0183 |
| A0A0C4DH29 | Immunoglobulin heavy variable 1-3 OS=Homo sapiens OX=9606 GN=IGHV1-3 PE=3 SV=1 | IGHV1-3 | 13.008 | 0.69 | 0.0161 | 0.95 | 0.7275 | 0.72 | 0.0108 |
| P02788 | Lactotransferrin OS=Homo sapiens OX=9606 GN=LTF PE=1 SV=6 | LTF | 78.181 | 1.08 | 0.9413 | 0.95 | 0.8889 | 1.14 | 0.8212 |
| P20810 | Calpastatin OS=Homo sapiens OX=9606 GN=CAST PE=1 SV=4 | CAST | 76.572 | 0.92 | 0.6061 | 0.69 | 0.4324 | 1.32 | 0.5251 |
| P61225 | Ras-related protein Rap-2b OS=Homo sapiens OX=9606 GN=RAP2B PE=1 SV=1 | RAP2B | 20.504 | 0.82 | 0.1688 | 0.84 | 0.1809 | 0.98 | 0.7751 |
| P63092 | Guanine nucleotide-binding protein G(s) subunit alpha isoforms short OS=Homo sapiens OX=9606 GN=GNAS PE=1 SV=1 | GNAS | 45.664 | 1.01 | 0.9708 | 0.96 | 0.7689 | 1.05 | 0.7207 |
| Q05193 | Dynamin-1 OS=Homo sapiens OX=9606 GN=DNM1 PE=1 SV=2 | DNM1 | 97.407 |  |  |  |  |  |  |
| P14314 | Glucosidase 2 subunit beta OS=Homo sapiens OX=9606 GN=PRKCSH PE=1 SV=2 | PRKCSH | 59.425 | 0.84 | 0.7075 | 0.96 | 0.8365 | 0.87 | 0.6429 |
| P09382 | Galectin-1 OS=Homo sapiens OX=9606 GN=LGALS1 PE=1 SV=2 | LGALS1 | 14.716 | 0.97 | 0.9234 | 0.83 | 0.3733 | 1.17 | 0.4586 |
| P36980 | Complement factor H-related protein 2 OS=Homo sapiens OX=9606 GN=CFHR2 PE=1 SV=1 | CFHR2 | 30.65 | 0.89 | 0.3999 | 1.00 | 0.8499 | 0.89 | 0.3238 |
| P00390 | Glutathione reductase, mitochondrial OS=Homo sapiens OX=9606 GN=GSR PE=1 SV=2 | GSR | 56.256 |  |  |  |  |  |  |
| P42224 | Signal transducer and activator of transcription 1-alpha/beta OS=Homo sapiens OX=9606 GN=STAT1 PE=1 SV=2 | STAT1 | 87.334 | 0.53 | 0.0295 | 0.82 | 0.4283 | 0.65 | 0.0565 |
| P05156 | Complement factor I OS=Homo sapiens OX=9606 GN=CFI PE=1 SV=2 | CFI | 65.75 | 1.30 | 0.2852 | 1.61 | 0.0095 | 0.81 | 0.2117 |
| P27797 | Calreticulin OS=Homo sapiens OX=9606 GN=CALR PE=1 SV=1 | CALR | 48.141 | 0.76 | 0.1534 | 0.77 | 0.3491 | 1.00 | 0.5435 |
| P46597 | Acetylserotonin O-methyltransferase OS=Homo sapiens OX=9606 GN=ASMT PE=1 SV=1 | ASMT | 38.452 | 0.51 | 0.0282 | 0.70 | 0.3050 | 0.73 | 0.2763 |
| Q96HQ0 | Zinc finger protein 419 OS=Homo sapiens OX=9606 GN=ZNF419 PE=1 SV=2 | ZNF419 | 58.563 | 0.59 | 0.1100 | 0.54 | 0.1167 | 1.10 | 0.9120 |
| P35542 | Serum amyloid A-4 protein OS=Homo sapiens OX=9606 GN=SAA4 PE=1 SV=2 | SAA4 | 14.746 | 0.99 | 0.8741 | 1.26 | 0.1573 | 0.79 | 0.3065 |
| Q8NCG7 | Sn1-specific diacylglycerol lipase beta OS=Homo sapiens OX=9606 GN=DAGLB PE=1 SV=2 | DAGLB | 73.731 | 0.68 | 0.4115 | 0.29 | 0.3979 | 2.32 | 0.7722 |
| Q15147 | 1-phosphatidylinositol 4,5-bisphosphate phosphodiesterase beta-4 OS=Homo sapiens OX=9606 GN=PLCB4 PE=1 SV=3 | PLCB4 | 134.46 | 1.23 | 0.0952 | 0.26 | 0.4140 | 4.80 | 0.2977 |
| P00352 | Retinal dehydrogenase 1 OS=Homo sapiens OX=9606 GN=ALDH1A1 PE=1 SV=2 | ALDH1A1 | 54.861 | 1.53 | 0.8428 | 2.17 | 0.4750 | 0.70 | 0.3576 |
| Q9H173 | Nucleotide exchange factor SIL1 OS=Homo sapiens OX=9606 GN=SIL1 PE=1 SV=1 | SIL1 | 52.084 | 1.00 | 0.9830 | 0.48 | 0.2338 | 2.09 | 0.2359 |
| P04406 | Glyceraldehyde-3-phosphate dehydrogenase OS=Homo sapiens OX=9606 GN=GAPDH PE=1 SV=3 | GAPDH | 36.053 | 0.81 | 0.0835 | 1.00 | 0.9849 | 0.81 | 0.0432 |
| Q15582 | Transforming growth factor-beta-induced protein ig-h3 OS=Homo sapiens OX=9606 GN=TGFBI PE=1 SV=1 | TGFBI | 74.68 | 0.91 | 0.2891 | 0.94 | 0.3392 | 0.97 | 0.7338 |
| Q96GR2 | Long-chain-fatty-acid--CoA ligase ACSBG1 OS=Homo sapiens OX=9606 GN=ACSBG1 PE=2 SV=2 | ACSBG1 | 81.289 | 1.52 | 0.1453 | 0.84 | 0.5671 | 1.80 | 0.1078 |
| Q8IZ41 | Ras and EF-hand domain-containing protein OS=Homo sapiens OX=9606 GN=RASEF PE=1 SV=1 | RASEF | 82.878 | 3.34 | 0.0367 | 1.85 | 0.1110 | 1.81 | 0.2233 |
| Q5T5P2 | Sickle tail protein homolog OS=Homo sapiens OX=9606 GN=KIAA1217 PE=1 SV=2 | KIAA1217 | 214.11 | 1.10 | 0.7039 | 0.86 | 0.4857 | 1.28 | 0.3421 |
| Q92900 | Regulator of nonsense transcripts 1 OS=Homo sapiens OX=9606 GN=UPF1 PE=1 SV=2 | UPF1 | 124.34 | 0.48 | 0.1749 | 0.29 | 0.1970 | 1.68 | 0.8962 |
| P11766 | Alcohol dehydrogenase class-3 OS=Homo sapiens OX=9606 GN=ADH5 PE=1 SV=4 | ADH5 | 39.724 |  |  |  |  |  |  |
| Q969U7 | Proteasome assembly chaperone 2 OS=Homo sapiens OX=9606 GN=PSMG2 PE=1 SV=1 | PSMG2 | 29.396 | 0.53 | 0.0060 | 0.80 | 0.7474 | 0.66 | 0.2024 |
| P27105 | Erythrocyte band 7 integral membrane protein OS=Homo sapiens OX=9606 GN=STOM PE=1 SV=3 | STOM | 31.73 | 0.73 | 0.0254 | 0.79 | 0.1635 | 0.92 | 0.4239 |
| Q15084 | Protein disulfide-isomerase A6 OS=Homo sapiens OX=9606 GN=PDIA6 PE=1 SV=1 | PDIA6 | 48.121 | 0.80 | 0.2191 | 0.85 | 0.3286 | 0.94 | 0.5509 |
| Q15942 | Zyxin OS=Homo sapiens OX=9606 GN=ZYX PE=1 SV=1 | ZYX | 61.277 | 0.77 | 0.1259 | 0.78 | 0.2920 | 0.99 | 0.7230 |
| P01040 | Cystatin-A OS=Homo sapiens OX=9606 GN=CSTA PE=1 SV=1 | CSTA | 11.006 | 1.23 | 0.8799 | 0.77 | 0.6043 | 1.61 | 0.0283 |
| P01764 | Immunoglobulin heavy variable 3-23 OS=Homo sapiens OX=9606 GN=IGHV3-23 PE=1 SV=2 | IGHV3-23 | 12.582 | 1.40 | 0.2900 | 1.16 | 0.4815 | 1.20 | 0.4891 |
| O95347 | Structural maintenance of chromosomes protein 2 OS=Homo sapiens OX=9606 GN=SMC2 PE=1 SV=2 | SMC2 | 135.65 |  |  |  |  |  |  |
| P12110 | Collagen alpha-2(VI) chain OS=Homo sapiens OX=9606 GN=COL6A2 PE=1 SV=4 | COL6A2 | 108.58 | 1.15 | 0.1721 | 0.98 | 0.8288 | 1.17 | 0.0854 |
| Q9Y210 | Short transient receptor potential channel 6 OS=Homo sapiens OX=9606 GN=TRPC6 PE=1 SV=1 | TRPC6 | 106.33 | 1.20 | 0.9826 | 0.63 | 0.3118 | 1.91 | 0.2821 |
| P19652 | Alpha-1-acid glycoprotein 2 OS=Homo sapiens OX=9606 GN=ORM2 PE=1 SV=2 | ORM2 | 23.602 | 0.85 | 0.2786 | 1.29 | 0.3054 | 0.66 | 0.0076 |
| O75747 | Phosphatidylinositol 4-phosphate 3-kinase C2 domain-containing subunit gamma OS=Homo sapiens OX=9606 GN=PIK3C2G PE=1 SV=3 | PIK3C2G | 165.71 | 1.82 | 0.1063 | 1.00 | 0.8824 | 1.81 | 0.0266 |
| P68371 | Tubulin beta-4B chain OS=Homo sapiens OX=9606 GN=TUBB4B PE=1 SV=1 | TUBB4B | 49.83 |  |  |  |  |  |  |
| P14543 | Nidogen-1 OS=Homo sapiens OX=9606 GN=NID1 PE=1 SV=3 | NID1 | 136.38 | 1.23 | 0.3036 | 0.99 | 0.7201 | 1.24 | 0.1418 |
| P55056 | Apolipoprotein C-IV OS=Homo sapiens OX=9606 GN=APOC4 PE=1 SV=1 | APOC4 | 14.553 | 0.74 | 0.4039 | 0.85 | 0.4098 | 0.87 | 0.6352 |
| Q9NQ76 | Matrix extracellular phosphoglycoprotein OS=Homo sapiens OX=9606 GN=MEPE PE=1 SV=1 | MEPE | 58.418 | 1.64 | 0.1113 | 0.56 | 0.2614 | 2.93 | 0.0444 |
| Q14789 | Golgin subfamily B member 1 OS=Homo sapiens OX=9606 GN=GOLGB1 PE=1 SV=2 | GOLGB1 | 376.01 | 3.13 | 0.3761 | 1.38 | 0.6692 | 2.27 | 0.3729 |
| A0A075B6K6 | Immunoglobulin lambda variable 4-3 OS=Homo sapiens OX=9606 GN=IGLV4-3 PE=3 SV=1 | IGLV4-3 | 13.33 | 1.02 | 0.9119 | 1.29 | 0.3157 | 0.79 | 0.4306 |
| Q15021 | Condensin complex subunit 1 OS=Homo sapiens OX=9606 GN=NCAPD2 PE=1 SV=3 | NCAPD2 | 157.18 | 3.83 | 0.2533 | 3.20 | 0.4011 | 1.20 | 0.4979 |
| P48553 | Trafficking protein particle complex subunit 10 OS=Homo sapiens OX=9606 GN=TRAPPC10 PE=1 SV=2 | TRAPPC10 | 142.19 | 0.69 | 0.9130 | 0.74 | 0.2416 | 0.92 | 0.8636 |
| P05546 | Heparin cofactor 2 OS=Homo sapiens OX=9606 GN=SERPIND1 PE=1 SV=3 | SERPIND1 | 57.07 | 1.61 | 0.0589 | 1.42 | 0.0505 | 1.13 | 0.3650 |
| P07900 | Heat shock protein HSP 90-alpha OS=Homo sapiens OX=9606 GN=HSP90AA1 PE=1 SV=5 | HSP90AA1 | 84.659 | 0.99 | 0.8938 | 0.89 | 0.4459 | 1.11 | 0.5549 |
| Q9NZT1 | Calmodulin-like protein 5 OS=Homo sapiens OX=9606 GN=CALML5 PE=1 SV=2 | CALML5 | 15.892 | 2.09 | 0.4110 | 1.43 | 0.7901 | 1.46 | 0.3097 |
| O15162 | Phospholipid scramblase 1 OS=Homo sapiens OX=9606 GN=PLSCR1 PE=1 SV=1 | PLSCR1 | 35.049 | 0.59 | 0.0602 | 0.95 | 0.7223 | 0.62 | 0.0261 |
| P50395 | Rab GDP dissociation inhibitor beta OS=Homo sapiens OX=9606 GN=GDI2 PE=1 SV=2 | GDI2 | 50.663 | 0.72 | 0.1886 | 1.00 | 0.9803 | 0.72 | 0.1496 |
| Q96JF0 | Beta-galactoside alpha-2,6-sialyltransferase 2 OS=Homo sapiens OX=9606 GN=ST6GAL2 PE=1 SV=2 | ST6GAL2 | 60.157 | 0.68 | 0.0636 | 0.77 | 0.1081 | 0.88 | 0.4137 |
| Q9Y6C2 | EMILIN-1 OS=Homo sapiens OX=9606 GN=EMILIN1 PE=1 SV=3 | EMILIN1 | 106.69 | 1.16 | 0.3237 | 1.02 | 0.9975 | 1.14 | 0.0882 |
| P04275 | von Willebrand factor OS=Homo sapiens OX=9606 GN=VWF PE=1 SV=4 | VWF | 309.26 | 2.55 | 0.0002 | 1.59 | 0.0081 | 1.61 | 0.0133 |
| O43805 | Sjoegren syndrome nuclear autoantigen 1 OS=Homo sapiens OX=9606 GN=SSNA1 PE=1 SV=2 | SSNA1 | 13.596 | 0.14 | 0.4832 | 0.71 | 0.2927 | 0.20 | 0.5622 |
| O60879 | Protein diaphanous homolog 2 OS=Homo sapiens OX=9606 GN=DIAPH2 PE=1 SV=1 | DIAPH2 | 125.57 | 1.61 | 0.1694 | 1.21 | 0.4138 | 1.34 | 0.0518 |
| P29597 | Non-receptor tyrosine-protein kinase TYK2 OS=Homo sapiens OX=9606 GN=TYK2 PE=1 SV=3 | TYK2 | 133.65 | 0.49 | 0.0298 | 0.65 | 0.2941 | 0.76 | 0.3207 |
| Q8IZP2 | Putative protein FAM10A4 OS=Homo sapiens OX=9606 GN=ST13P4 PE=5 SV=1 | ST13P4 | 27.406 | 0.88 | 0.5687 | 0.91 | 0.8149 | 0.96 | 0.7772 |
| O14513 | Nck-associated protein 5 OS=Homo sapiens OX=9606 GN=NCKAP5 PE=1 SV=2 | NCKAP5 | 208.53 | 2.54 | 0.0300 | 0.92 | 0.9223 | 2.75 | 0.0441 |
| Q5VT25 | Serine/threonine-protein kinase MRCK alpha OS=Homo sapiens OX=9606 GN=CDC42BPA PE=1 SV=1 | CDC42BPA | 197.3 | 1.08 | 0.7919 | 1.61 | 0.1689 | 0.67 | 0.2995 |
| Q99436 | Proteasome subunit beta type-7 OS=Homo sapiens OX=9606 GN=PSMB7 PE=1 SV=1 | PSMB7 | 29.965 | 1.84 | 0.0450 | 1.12 | 0.5295 | 1.65 | 0.4200 |
