# Supplementary material for: Identification of exosome protein panels as predictive biomarkers for non-small cell lung cancer
Source: Biol Proced Online. 2023 Nov 13;25:29. doi: 10.1186/s12575-023-00223-0 (PMC10641949; doi:10.1186/s12575-023-00223-0)
Supplement: Supplementary file 4 — Additional file 4. Table S2. All detected proteins that passed through TMT. [file 12575_2023_223_MOESM4_ESM.doc]

| **Compared sample name** | **Up-regulated** | **Down-regulated** |
| --- | --- | --- |
| M/A | 47 | 116 |
| M/N | 28 | 30 |
| N/A | 65 | 76 |


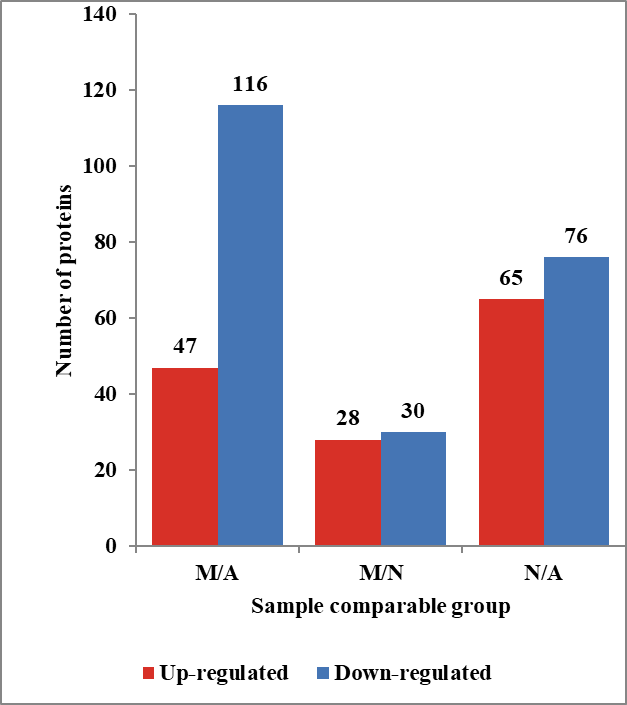


M/A

| **Protein accession** | **Protein description** | **Gene name** | **MW [kDa]** | **M/A Ratio** | | **Regulated Type** | **M/A**  **P value** |
| --- | --- | --- | --- | --- | --- | --- | --- |
| Q5VZ89 | DENN domain-containing protein 4C OS=Homo sapiens OX=9606 GN=DENND4C PE=1 SV=3 | DENND4C | 212.71 | 5.395 | | Up | 0.0088152 |
| Q6ZUS5 | Coiled-coil domain-containing protein 121 OS=Homo sapiens OX=9606 GN=CCDC121 PE=1 SV=1 | CCDC121 | 33.06 | | 3.61 | Up | 0.004339 |
| P02792 | Ferritin light chain OS=Homo sapiens OX=9606 GN=FTL PE=1 SV=2 | FTL | 20.019 | | 3.396 | Up | 0.00122431 |
| Q8IZ41 | Ras and EF-hand domain-containing protein OS=Homo sapiens OX=9606 GN=RASEF PE=1 SV=1 | RASEF | 82.878 | | 3.335 | Up | 0.036699 |
| Q08830 | Fibrinogen-like protein 1 OS=Homo sapiens OX=9606 GN=FGL1 PE=1 SV=3 | FGL1 | 36.379 | | 2.825 | Up | 0.00082071 |
| P02679 | Fibrinogen gamma chain OS=Homo sapiens OX=9606 GN=FGG PE=1 SV=3 | FGG | 51.511 | | 2.778 | Up | 0.00011878 |
| P02675 | Fibrinogen beta chain OS=Homo sapiens OX=9606 GN=FGB PE=1 SV=2 | FGB | 55.928 | | 2.674 | Up | 0.000163327 |
| Q15485 | Ficolin-2 OS=Homo sapiens OX=9606 GN=FCN2 PE=1 SV=2 | FCN2 | 34.001 | | 2.557 | Up | 0.00025796 |
| P04275 | von Willebrand factor OS=Homo sapiens OX=9606 GN=VWF PE=1 SV=4 | VWF | 309.26 | | 2.552 | Up | 0.000155947 |
| O14513 | Nck-associated protein 5 OS=Homo sapiens OX=9606 GN=NCKAP5 PE=1 SV=2 | NCKAP5 | 208.53 | | 2.539 | Up | 0.029979 |
| Q5JV73 | FERM and PDZ domain-containing protein 3 OS=Homo sapiens OX=9606 GN=FRMPD3 PE=2 SV=2 | FRMPD3 | 199.21 | | 2.477 | Up | 0.030102 |
| P02671 | Fibrinogen alpha chain OS=Homo sapiens OX=9606 GN=FGA PE=1 SV=2 | FGA | 94.972 | | 2.443 | Up | 7.4585E-07 |
| Q7Z408 | CUB and sushi domain-containing protein 2 OS=Homo sapiens OX=9606 GN=CSMD2 PE=1 SV=2 | CSMD2 | 380.03 | | 2.427 | Up | 0.034742 |
| P46952 | "3-hydroxyanthranilate 3,4-dioxygenase OS=Homo sapiens OX=9606 GN=HAAO PE=1 SV=2" | HAAO | 32.556 | | 2.408 | Up | 0.0077385 |
| O00602 | Ficolin-1 OS=Homo sapiens OX=9606 GN=FCN1 PE=1 SV=2 | FCN1 | 35.078 | | 2.322 | Up | 0.022515 |
| P18428 | Lipopolysaccharide-binding protein OS=Homo sapiens OX=9606 GN=LBP PE=1 SV=3 | LBP | 53.383 | | 2.19 | Up | 0.0179351 |
| P08697 | Alpha-2-antiplasmin OS=Homo sapiens OX=9606 GN=SERPINF2 PE=1 SV=3 | SERPINF2 | 54.565 | | 1.961 | Up | 0.00109819 |
| P02748 | Complement component C9 OS=Homo sapiens OX=9606 GN=C9 PE=1 SV=2 | C9 | 63.173 | | 1.946 | Up | 0.0032596 |
| P01705 | Immunoglobulin lambda variable 2-23 OS=Homo sapiens OX=9606 GN=IGLV2-23 PE=1 SV=2 | IGLV2-23 | 11.893 | | 1.939 | Up | 0.037096 |
| O60508 | Pre-mRNA-processing factor 17 OS=Homo sapiens OX=9606 GN=CDC40 PE=1 SV=1 | CDC40 | 65.521 | | 1.917 | Up | 0.0180803 |
| Q9UPN9 | E3 ubiquitin-protein ligase TRIM33 OS=Homo sapiens OX=9606 GN=TRIM33 PE=1 SV=3 | TRIM33 | 122.53 | | 1.882 | Up | 0.00150363 |
| Q9BXR6 | Complement factor H-related protein 5 OS=Homo sapiens OX=9606 GN=CFHR5 PE=1 SV=1 | CFHR5 | 64.419 | | 1.871 | Up | 0.0008362 |
| Q99436 | Proteasome subunit beta type-7 OS=Homo sapiens OX=9606 GN=PSMB7 PE=1 SV=1 | PSMB7 | 29.965 | | 1.844 | Up | 0.045037 |
| Q9NVF7 | F-box only protein 28 OS=Homo sapiens OX=9606 GN=FBXO28 PE=1 SV=1 | FBXO28 | 41.149 | | 1.794 | Up | 0.049398 |
| Q99570 | Phosphoinositide 3-kinase regulatory subunit 4 OS=Homo sapiens OX=9606 GN=PIK3R4 PE=1 SV=3 | PIK3R4 | 153.1 | | 1.752 | Up | 0.0043382 |
| Q16610 | Extracellular matrix protein 1 OS=Homo sapiens OX=9606 GN=ECM1 PE=1 SV=2 | ECM1 | 60.673 | | 1.731 | Up | 0.0041441 |
| Q9Y2R2 | Tyrosine-protein phosphatase non-receptor type 22 OS=Homo sapiens OX=9606 GN=PTPN22 PE=1 SV=2 | PTPN22 | 91.704 | | 1.697 | Up | 0.022021 |
| P57739 | Claudin-2 OS=Homo sapiens OX=9606 GN=CLDN2 PE=1 SV=1 | CLDN2 | 24.548 | | 1.664 | Up | 0.0070797 |
| Q8WWZ8 | Oncoprotein-induced transcript 3 protein OS=Homo sapiens OX=9606 GN=OIT3 PE=1 SV=2 | OIT3 | 60.021 | | 1.639 | Up | 0.0181034 |
| Q13867 | Bleomycin hydrolase OS=Homo sapiens OX=9606 GN=BLMH PE=1 SV=1 | BLMH | 52.562 | | 1.574 | Up | 0.029625 |
| Q86X52 | Chondroitin sulfate synthase 1 OS=Homo sapiens OX=9606 GN=CHSY1 PE=1 SV=3 | CHSY1 | 91.783 | | 1.536 | Up | 0.043962 |
| P04003 | C4b-binding protein alpha chain OS=Homo sapiens OX=9606 GN=C4BPA PE=1 SV=2 | C4BPA | 67.033 | | 1.518 | Up | 0.00043595 |
| P10909 | Clusterin OS=Homo sapiens OX=9606 GN=CLU PE=1 SV=1 | CLU | 52.494 | | 1.513 | Up | 0.0059589 |
| P35241 | Radixin OS=Homo sapiens OX=9606 GN=RDX PE=1 SV=1 | RDX | 68.563 | | 1.497 | Up | 0.047401 |
| A0A075B6K0 | Immunoglobulin lambda variable 3-16 OS=Homo sapiens OX=9606 GN=IGLV3-16 PE=3 SV=2 | IGLV3-16 | 12.466 | | 1.49 | Up | 0.027641 |
| Q5T0Z8 | Uncharacterized protein C6orf132 OS=Homo sapiens OX=9606 GN=C6orf132 PE=1 SV=4 | C6orf132 | 124.03 | | 1.481 | Up | 0.00128412 |
| P01011 | Alpha-1-antichymotrypsin OS=Homo sapiens OX=9606 GN=SERPINA3 PE=1 SV=2 | SERPINA3 | 47.65 | | 1.458 | Up | 0.04178 |
| P05160 | Coagulation factor XIII B chain OS=Homo sapiens OX=9606 GN=F13B PE=1 SV=3 | F13B | 75.51 | | 1.446 | Up | 0.039839 |
| P00488 | Coagulation factor XIII A chain OS=Homo sapiens OX=9606 GN=F13A1 PE=1 SV=4 | F13A1 | 83.266 | | 1.442 | Up | 0.0069826 |
| P98160 | Basement membrane-specific heparan sulfate proteoglycan core protein OS=Homo sapiens OX=9606 GN=HSPG2 PE=1 SV=4 | HSPG2 | 468.83 | | 1.43 | Up | 0.0110351 |
| Q86UW7 | Calcium-dependent secretion activator 2 OS=Homo sapiens OX=9606 GN=CADPS2 PE=1 SV=2 | CADPS2 | 147.73 | | 1.351 | Up | 0.0056387 |
| P07225 | Vitamin K-dependent protein S OS=Homo sapiens OX=9606 GN=PROS1 PE=1 SV=1 | PROS1 | 75.122 | | 1.342 | Up | 0.0080989 |
| Q9HCU9 | Breast cancer metastasis-suppressor 1 OS=Homo sapiens OX=9606 GN=BRMS1 PE=1 SV=1 | BRMS1 | 28.46 | | 1.335 | Up | 0.039163 |
| P20851 | C4b-binding protein beta chain OS=Homo sapiens OX=9606 GN=C4BPB PE=1 SV=1 | C4BPB | 28.357 | | 1.32 | Up | 0.0138185 |
| Q9ULI3 | Protein HEG homolog 1 OS=Homo sapiens OX=9606 GN=HEG1 PE=1 SV=3 | HEG1 | 147.46 | | 1.32 | Up | 0.028858 |
| Q08554 | Desmocollin-1 OS=Homo sapiens OX=9606 GN=DSC1 PE=1 SV=2 | DSC1 | 99.986 | | 1.276 | Up | 0.0160555 |
| P0C0L4 | Complement C4-A OS=Homo sapiens OX=9606 GN=C4A PE=1 SV=2 | C4A | 192.78 | | 1.206 | Up | 0.0056208 |
| P01591 | Immunoglobulin J chain OS=Homo sapiens OX=9606 GN=JCHAIN PE=1 SV=4 | JCHAIN | 18.098 | | 0.792 | Down | 0.038417 |
| Q8N392 | Rho GTPase-activating protein 18 OS=Homo sapiens OX=9606 GN=ARHGAP18 PE=1 SV=3 | ARHGAP18 | 74.976 | | 0.784 | Down | 0.037517 |
| Q9H4M9 | EH domain-containing protein 1 OS=Homo sapiens OX=9606 GN=EHD1 PE=1 SV=2 | EHD1 | 60.626 | | 0.779 | Down | 0.027463 |
| O43866 | CD5 antigen-like OS=Homo sapiens OX=9606 GN=CD5L PE=1 SV=1 | CD5L | 38.087 | | 0.758 | Down | 0.034344 |
| P50148 | Guanine nucleotide-binding protein G(q) subunit alpha OS=Homo sapiens OX=9606 GN=GNAQ PE=1 SV=4 | GNAQ | 42.142 | | 0.756 | Down | 0.045802 |
| P02774 | Vitamin D-binding protein OS=Homo sapiens OX=9606 GN=GC PE=1 SV=1 | GC | 52.963 | | 0.735 | Down | 0.0050604 |
| A0A0B4J1V0 | Immunoglobulin heavy variable 3-15 OS=Homo sapiens OX=9606 GN=IGHV3-15 PE=3 SV=1 | IGHV3-15 | 12.926 | | 0.735 | Down | 0.0052171 |
| Q9NQC3 | Reticulon-4 OS=Homo sapiens OX=9606 GN=RTN4 PE=1 SV=2 | RTN4 | 129.93 | | 0.732 | Down | 0.035739 |
| P0DOX6 | Immunoglobulin mu heavy chain OS=Homo sapiens OX=9606 PE=1 SV=2 | --- | 63.485 | | 0.731 | Down | 0.041942 |
| Q9NRW1 | Ras-related protein Rab-6B OS=Homo sapiens OX=9606 GN=RAB6B PE=1 SV=1 | RAB6B | 23.461 | | 0.726 | Down | 0.030061 |
| P27105 | Erythrocyte band 7 integral membrane protein OS=Homo sapiens OX=9606 GN=STOM PE=1 SV=3 | STOM | 31.73 | | 0.726 | Down | 0.025436 |
| Q05586 | "Glutamate receptor ionotropic, NMDA 1 OS=Homo sapiens OX=9606 GN=GRIN1 PE=1 SV=1" | GRIN1 | 105.37 | | 0.72 | Down | 0.0121209 |
| Q12913 | Receptor-type tyrosine-protein phosphatase eta OS=Homo sapiens OX=9606 GN=PTPRJ PE=1 SV=3 | PTPRJ | 145.94 | | 0.714 | Down | 0.034877 |
| Q9H0U4 | Ras-related protein Rab-1B OS=Homo sapiens OX=9606 GN=RAB1B PE=1 SV=1 | RAB1B | 22.171 | | 0.711 | Down | 0.035522 |
| P02647 | Apolipoprotein A-I OS=Homo sapiens OX=9606 GN=APOA1 PE=1 SV=1 | APOA1 | 30.777 | | 0.706 | Down | 0.00118192 |
| P61586 | Transforming protein RhoA OS=Homo sapiens OX=9606 GN=RHOA PE=1 SV=1 | RHOA | 21.768 | | 0.704 | Down | 0.013224 |
| O00139 | Kinesin-like protein KIF2A OS=Homo sapiens OX=9606 GN=KIF2A PE=1 SV=3 | KIF2A | 79.954 | | 0.704 | Down | 0.027495 |
| P01871 | Immunoglobulin heavy constant mu OS=Homo sapiens OX=9606 GN=IGHM PE=1 SV=4 | IGHM | 49.439 | | 0.703 | Down | 0.024937 |
| P11021 | Endoplasmic reticulum chaperone BiP OS=Homo sapiens OX=9606 GN=HSPA5 PE=1 SV=2 | HSPA5 | 72.332 | | 0.701 | Down | 0.0050229 |
| P01834 | Immunoglobulin kappa constant OS=Homo sapiens OX=9606 GN=IGKC PE=1 SV=2 | IGKC | 11.765 | | 0.701 | Down | 0.036962 |
| P0DP25 | Calmodulin-3 OS=Homo sapiens OX=9606 GN=CALM3 PE=1 SV=1 | CALM3 | 16.837 | | 0.695 | Down | 0.033836 |
| Q92522 | Histone H1x OS=Homo sapiens OX=9606 GN=H1FX PE=1 SV=1 | H1FX | 22.487 | | 0.693 | Down | 0.0105597 |
| Q99700 | Ataxin-2 OS=Homo sapiens OX=9606 GN=ATXN2 PE=1 SV=2 | ATXN2 | 140.28 | | 0.69 | Down | 0.044559 |
| A0A087WSX0 | Immunoglobulin lambda variable 5-45 OS=Homo sapiens OX=9606 GN=IGLV5-45 PE=3 SV=1 | IGLV5-45 | 13.162 | | 0.689 | Down | 0.036339 |
| P01859 | Immunoglobulin heavy constant gamma 2 OS=Homo sapiens OX=9606 GN=IGHG2 PE=1 SV=2 | IGHG2 | 35.9 | | 0.687 | Down | 0.0036573 |
| A0A0C4DH29 | Immunoglobulin heavy variable 1-3 OS=Homo sapiens OX=9606 GN=IGHV1-3 PE=3 SV=1 | IGHV1-3 | 13.008 | | 0.686 | Down | 0.0160795 |
| P0DP04 | Immunoglobulin heavy variable 3-43D OS=Homo sapiens OX=9606 GN=IGHV3-43D PE=3 SV=1 | IGHV3-43D | 13.017 | | 0.679 | Down | 0.00022109 |
| P06733 | Alpha-enolase OS=Homo sapiens OX=9606 GN=ENO1 PE=1 SV=2 | ENO1 | 47.168 | | 0.674 | Down | 0.0167369 |
| P19086 | Guanine nucleotide-binding protein G(z) subunit alpha OS=Homo sapiens OX=9606 GN=GNAZ PE=1 SV=3 | GNAZ | 40.923 | | 0.672 | Down | 0.039436 |
| P50991 | T-complex protein 1 subunit delta OS=Homo sapiens OX=9606 GN=CCT4 PE=1 SV=4 | CCT4 | 57.924 | | 0.66 | Down | 0.0094815 |
| O75954 | Tetraspanin-9 OS=Homo sapiens OX=9606 GN=TSPAN9 PE=1 SV=1 | TSPAN9 | 26.779 | | 0.66 | Down | 0.023361 |
| P05556 | Integrin beta-1 OS=Homo sapiens OX=9606 GN=ITGB1 PE=1 SV=2 | ITGB1 | 88.414 | | 0.66 | Down | 0.036444 |
| Q9ULV4 | Coronin-1C OS=Homo sapiens OX=9606 GN=CORO1C PE=1 SV=1 | CORO1C | 53.248 | | 0.658 | Down | 0.032124 |
| P31146 | Coronin-1A OS=Homo sapiens OX=9606 GN=CORO1A PE=1 SV=4 | CORO1A | 51.026 | | 0.658 | Down | 0.0151808 |
| P62937 | Peptidyl-prolyl cis-trans isomerase A OS=Homo sapiens OX=9606 GN=PPIA PE=1 SV=2 | PPIA | 18.012 | | 0.656 | Down | 0.048343 |
| Q5VTJ3 | Kelch domain-containing protein 7A OS=Homo sapiens OX=9606 GN=KLHDC7A PE=1 SV=5 | KLHDC7A | 84.478 | | 0.653 | Down | 0.0190788 |
| P01876 | Immunoglobulin heavy constant alpha 1 OS=Homo sapiens OX=9606 GN=IGHA1 PE=1 SV=2 | IGHA1 | 37.654 | | 0.65 | Down | 0.0182367 |
| P16284 | Platelet endothelial cell adhesion molecule OS=Homo sapiens OX=9606 GN=PECAM1 PE=1 SV=2 | PECAM1 | 82.521 | | 0.65 | Down | 0.031096 |
| P61158 | Actin-related protein 3 OS=Homo sapiens OX=9606 GN=ACTR3 PE=1 SV=3 | ACTR3 | 47.371 | | 0.65 | Down | 0.049102 |
| Q14247 | Src substrate cortactin OS=Homo sapiens OX=9606 GN=CTTN PE=1 SV=2 | CTTN | 61.585 | | 0.649 | Down | 0.026365 |
| O95810 | Caveolae-associated protein 2 OS=Homo sapiens OX=9606 GN=CAVIN2 PE=1 SV=3 | CAVIN2 | 47.173 | | 0.644 | Down | 0.0108155 |
| P01709 | Immunoglobulin lambda variable 2-8 OS=Homo sapiens OX=9606 GN=IGLV2-8 PE=1 SV=2 | IGLV2-8 | 12.382 | | 0.641 | Down | 0.039882 |
| Q04756 | Hepatocyte growth factor activator OS=Homo sapiens OX=9606 GN=HGFAC PE=1 SV=1 | HGFAC | 70.681 | | 0.639 | Down | 0.0032403 |
| P08567 | Pleckstrin OS=Homo sapiens OX=9606 GN=PLEK PE=1 SV=3 | PLEK | 40.124 | | 0.632 | Down | 0.023564 |
| P35579 | Myosin-9 OS=Homo sapiens OX=9606 GN=MYH9 PE=1 SV=4 | MYH9 | 226.53 | | 0.627 | Down | 0.043176 |
| P37837 | Transaldolase OS=Homo sapiens OX=9606 GN=TALDO1 PE=1 SV=2 | TALDO1 | 37.54 | | 0.62 | Down | 0.026778 |
| P00338 | L-lactate dehydrogenase A chain OS=Homo sapiens OX=9606 GN=LDHA PE=1 SV=2 | LDHA | 36.688 | | 0.615 | Down | 0.022384 |
| P61224 | Ras-related protein Rap-1b OS=Homo sapiens OX=9606 GN=RAP1B PE=1 SV=1 | RAP1B | 20.825 | | 0.612 | Down | 0.024383 |
| P06312 | Immunoglobulin kappa variable 4-1 OS=Homo sapiens OX=9606 GN=IGKV4-1 PE=1 SV=1 | IGKV4-1 | 13.38 | | 0.605 | Down | 0.00178497 |
| P0DOX3 | Immunoglobulin delta heavy chain OS=Homo sapiens OX=9606 PE=1 SV=1 | --- | 56.224 | | 0.605 | Down | 0.013576 |
| Q6UW60 | Proprotein convertase subtilisin/kexin type 4 OS=Homo sapiens OX=9606 GN=PCSK4 PE=1 SV=2 | PCSK4 | 82.794 | | 0.602 | Down | 0.023219 |
| P18206 | Vinculin OS=Homo sapiens OX=9606 GN=VCL PE=1 SV=4 | VCL | 123.8 | | 0.597 | Down | 0.013564 |
| P01780 | Immunoglobulin heavy variable 3-7 OS=Homo sapiens OX=9606 GN=IGHV3-7 PE=1 SV=2 | IGHV3-7 | 12.943 | | 0.595 | Down | 0.0033992 |
| Q99567 | Nuclear pore complex protein Nup88 OS=Homo sapiens OX=9606 GN=NUP88 PE=1 SV=2 | NUP88 | 83.541 | | 0.593 | Down | 0.0056045 |
| P04179 | "Superoxide dismutase [Mn], mitochondrial OS=Homo sapiens OX=9606 GN=SOD2 PE=1 SV=3" | SOD2 | 24.75 | | 0.591 | Down | 0.049276 |
| O14791 | Apolipoprotein L1 OS=Homo sapiens OX=9606 GN=APOL1 PE=1 SV=5 | APOL1 | 43.974 | | 0.587 | Down | 0.00152066 |
| P00739 | Haptoglobin-related protein OS=Homo sapiens OX=9606 GN=HPR PE=2 SV=2 | HPR | 39.029 | | 0.586 | Down | 0.0068976 |
| P10124 | Serglycin OS=Homo sapiens OX=9606 GN=SRGN PE=1 SV=3 | SRGN | 17.652 | | 0.585 | Down | 0.003384 |
| P21333 | Filamin-A OS=Homo sapiens OX=9606 GN=FLNA PE=1 SV=4 | FLNA | 280.74 | | 0.585 | Down | 0.011639 |
| A0A0B4J1X8 | Immunoglobulin heavy variable 3-43 OS=Homo sapiens OX=9606 GN=IGHV3-43 PE=3 SV=1 | IGHV3-43 | 13.077 | | 0.581 | Down | 0.040181 |
| P63104 | 14-3-3 protein zeta/delta OS=Homo sapiens OX=9606 GN=YWHAZ PE=1 SV=1 | YWHAZ | 27.745 | | 0.58 | Down | 0.02192 |
| P01042 | Kininogen-1 OS=Homo sapiens OX=9606 GN=KNG1 PE=1 SV=2 | KNG1 | 71.957 | | 0.578 | Down | 0.028815 |
| Q9Y490 | Talin-1 OS=Homo sapiens OX=9606 GN=TLN1 PE=1 SV=3 | TLN1 | 269.76 | | 0.578 | Down | 0.0191155 |
| P58546 | Myotrophin OS=Homo sapiens OX=9606 GN=MTPN PE=1 SV=2 | MTPN | 12.895 | | 0.577 | Down | 0.016939 |
| Q86UX7 | Fermitin family homolog 3 OS=Homo sapiens OX=9606 GN=FERMT3 PE=1 SV=1 | FERMT3 | 75.952 | | 0.575 | Down | 0.0146418 |
| Q04917 | 14-3-3 protein eta OS=Homo sapiens OX=9606 GN=YWHAH PE=1 SV=4 | YWHAH | 28.218 | | 0.574 | Down | 0.02578 |
| P48059 | LIM and senescent cell antigen-like-containing domain protein 1 OS=Homo sapiens OX=9606 GN=LIMS1 PE=1 SV=4 | LIMS1 | 37.251 | | 0.572 | Down | 0.028801 |
| Q13576 | Ras GTPase-activating-like protein IQGAP2 OS=Homo sapiens OX=9606 GN=IQGAP2 PE=1 SV=4 | IQGAP2 | 180.58 | | 0.565 | Down | 0.031041 |
| Q13418 | Integrin-linked protein kinase OS=Homo sapiens OX=9606 GN=ILK PE=1 SV=2 | ILK | 51.419 | | 0.562 | Down | 0.022944 |
| P04075 | Fructose-bisphosphate aldolase A OS=Homo sapiens OX=9606 GN=ALDOA PE=1 SV=2 | ALDOA | 39.42 | | 0.562 | Down | 0.0073972 |
| Q6P435 | Putative uncharacterized SMG1-like protein OS=Homo sapiens OX=9606 PE=5 SV=1 | --- | 17.652 | | 0.558 | Down | 0.0116395 |
| Q8WZ69 | Putative uncharacterized protein C11orf40 OS=Homo sapiens OX=9606 GN=C11orf40 PE=2 SV=1 | C11orf40 | 24.619 | | 0.556 | Down | 0.023875 |
| P12814 | Alpha-actinin-1 OS=Homo sapiens OX=9606 GN=ACTN1 PE=1 SV=2 | ACTN1 | 103.06 | | 0.555 | Down | 0.029336 |
| A0A0C4DH68 | Immunoglobulin kappa variable 2-24 OS=Homo sapiens OX=9606 GN=IGKV2-24 PE=3 SV=1 | IGKV2-24 | 13.079 | | 0.553 | Down | 0.0127793 |
| Q9HBI1 | Beta-parvin OS=Homo sapiens OX=9606 GN=PARVB PE=1 SV=1 | PARVB | 41.714 | | 0.552 | Down | 0.034665 |
| Q8IYT4 | Katanin p60 ATPase-containing subunit A-like 2 OS=Homo sapiens OX=9606 GN=KATNAL2 PE=1 SV=3 | KATNAL2 | 61.252 | | 0.546 | Down | 0.0088357 |
| P60953 | Cell division control protein 42 homolog OS=Homo sapiens OX=9606 GN=CDC42 PE=1 SV=2 | CDC42 | 21.258 | | 0.546 | Down | 0.00072076 |
| O00299 | Chloride intracellular channel protein 1 OS=Homo sapiens OX=9606 GN=CLIC1 PE=1 SV=4 | CLIC1 | 26.922 | | 0.544 | Down | 0.0062975 |
| P01019 | Angiotensinogen OS=Homo sapiens OX=9606 GN=AGT PE=1 SV=1 | AGT | 53.154 | | 0.541 | Down | 0.0090425 |
| P13224 | Platelet glycoprotein Ib beta chain OS=Homo sapiens OX=9606 GN=GP1BB PE=1 SV=1 | GP1BB | 21.717 | | 0.532 | Down | 0.0191984 |
| P42224 | Signal transducer and activator of transcription 1-alpha/beta OS=Homo sapiens OX=9606 GN=STAT1 PE=1 SV=2 | STAT1 | 87.334 | | 0.532 | Down | 0.029502 |
| Q969U7 | Proteasome assembly chaperone 2 OS=Homo sapiens OX=9606 GN=PSMG2 PE=1 SV=1 | PSMG2 | 29.396 | | 0.532 | Down | 0.0060207 |
| O43149 | Zinc finger ZZ-type and EF-hand domain-containing protein 1 OS=Homo sapiens OX=9606 GN=ZZEF1 PE=1 SV=6 | ZZEF1 | 331.07 | | 0.529 | Down | 0.01792 |
| P67936 | Tropomyosin alpha-4 chain OS=Homo sapiens OX=9606 GN=TPM4 PE=1 SV=3 | TPM4 | 28.521 | | 0.528 | Down | 0.024264 |
| P07195 | L-lactate dehydrogenase B chain OS=Homo sapiens OX=9606 GN=LDHB PE=1 SV=2 | LDHB | 36.638 | | 0.524 | Down | 0.0048568 |
| P31946 | 14-3-3 protein beta/alpha OS=Homo sapiens OX=9606 GN=YWHAB PE=1 SV=3 | YWHAB | 28.082 | | 0.517 | Down | 0.0141966 |
| P63267 | "Actin, gamma-enteric smooth muscle OS=Homo sapiens OX=9606 GN=ACTG2 PE=1 SV=1" | ACTG2 | 41.876 | | 0.517 | Down | 0.0077781 |
| O75083 | WD repeat-containing protein 1 OS=Homo sapiens OX=9606 GN=WDR1 PE=1 SV=4 | WDR1 | 66.193 | | 0.516 | Down | 0.0024173 |
| Q9NR20 | Dual specificity tyrosine-phosphorylation-regulated kinase 4 OS=Homo sapiens OX=9606 GN=DYRK4 PE=1 SV=2 | DYRK4 | 59.608 | | 0.515 | Down | 0.0137631 |
| P46597 | Acetylserotonin O-methyltransferase OS=Homo sapiens OX=9606 GN=ASMT PE=1 SV=1 | ASMT | 38.452 | | 0.512 | Down | 0.028235 |
| O15144 | Actin-related protein 2/3 complex subunit 2 OS=Homo sapiens OX=9606 GN=ARPC2 PE=1 SV=1 | ARPC2 | 34.333 | | 0.511 | Down | 0.022225 |
| O75558 | Syntaxin-11 OS=Homo sapiens OX=9606 GN=STX11 PE=1 SV=1 | STX11 | 33.195 | | 0.506 | Down | 0.0042554 |
| P03951 | Coagulation factor XI OS=Homo sapiens OX=9606 GN=F11 PE=1 SV=1 | F11 | 70.108 | | 0.502 | Down | 0.026756 |
| P59998 | Actin-related protein 2/3 complex subunit 4 OS=Homo sapiens OX=9606 GN=ARPC4 PE=1 SV=3 | ARPC4 | 19.667 | | 0.502 | Down | 0.0181175 |
| P29597 | Non-receptor tyrosine-protein kinase TYK2 OS=Homo sapiens OX=9606 GN=TYK2 PE=1 SV=3 | TYK2 | 133.65 | | 0.493 | Down | 0.02978 |
| O15145 | Actin-related protein 2/3 complex subunit 3 OS=Homo sapiens OX=9606 GN=ARPC3 PE=1 SV=3 | ARPC3 | 20.546 | | 0.48 | Down | 0.0169366 |
| P60660 | Myosin light polypeptide 6 OS=Homo sapiens OX=9606 GN=MYL6 PE=1 SV=2 | MYL6 | 16.93 | | 0.474 | Down | 0.0182156 |
| Q9HD89 | Resistin OS=Homo sapiens OX=9606 GN=RETN PE=1 SV=1 | RETN | 11.419 | | 0.467 | Down | 0.0065018 |
| P04211 | Immunoglobulin lambda variable 7-43 OS=Homo sapiens OX=9606 GN=IGLV7-43 PE=3 SV=2 | IGLV7-43 | 12.451 | | 0.45 | Down | 0.0036614 |
| P10720 | Platelet factor 4 variant OS=Homo sapiens OX=9606 GN=PF4V1 PE=1 SV=1 | PF4V1 | 11.553 | | 0.45 | Down | 0.00060071 |
| P60709 | "Actin, cytoplasmic 1 OS=Homo sapiens OX=9606 GN=ACTB PE=1 SV=1" | ACTB | 41.736 | | 0.449 | Down | 0.0100002 |
| Q9H299 | SH3 domain-binding glutamic acid-rich-like protein 3 OS=Homo sapiens OX=9606 GN=SH3BGRL3 PE=1 SV=1 | SH3BGRL3 | 10.438 | | 0.447 | Down | 0.0054824 |
| A0A075B6K4 | Immunoglobulin lambda variable 3-10 OS=Homo sapiens OX=9606 GN=IGLV3-10 PE=3 SV=2 | IGLV3-10 | 12.441 | | 0.421 | Down | 0.047763 |
| P0DKL9 | ARL14 effector protein-like OS=Homo sapiens OX=9606 GN=ARL14EPL PE=4 SV=1 | ARL14EPL | 17.712 | | 0.387 | Down | 0.040624 |
| P23528 | Cofilin-1 OS=Homo sapiens OX=9606 GN=CFL1 PE=1 SV=3 | CFL1 | 18.502 | | 0.383 | Down | 0.00175779 |
| P01602 | Immunoglobulin kappa variable 1-5 OS=Homo sapiens OX=9606 GN=IGKV1-5 PE=1 SV=2 | IGKV1-5 | 12.781 | | 0.375 | Down | 0.0024376 |
| Q99715 | Collagen alpha-1(XII) chain OS=Homo sapiens OX=9606 GN=COL12A1 PE=1 SV=2 | COL12A1 | 333.14 | | 0.372 | Down | 0.035519 |
| A0A0B4J1X5 | Immunoglobulin heavy variable 3-74 OS=Homo sapiens OX=9606 GN=IGHV3-74 PE=3 SV=1 | IGHV3-74 | 12.839 | | 0.333 | Down | 0.0196997 |
| P02776 | Platelet factor 4 OS=Homo sapiens OX=9606 GN=PF4 PE=1 SV=2 | PF4 | 10.845 | | 0.324 | Down | 0.0098035 |
| Q9ULD9 | Zinc finger protein 608 OS=Homo sapiens OX=9606 GN=ZNF608 PE=1 SV=4 | ZNF608 | 162.21 | | 0.319 | Down | 0.0087378 |
| O00231 | 26S proteasome non-ATPase regulatory subunit 11 OS=Homo sapiens OX=9606 GN=PSMD11 PE=1 SV=3 | PSMD11 | 47.463 | | 0.314 | Down | 0.0173839 |
| O95841 | Angiopoietin-related protein 1 OS=Homo sapiens OX=9606 GN=ANGPTL1 PE=2 SV=1 | ANGPTL1 | 56.719 | | 0.228 | Down | 0.04478 |
| O15049 | NEDD4-binding protein 3 OS=Homo sapiens OX=9606 GN=N4BP3 PE=1 SV=3 | N4BP3 | 60.469 | | 0.22 | Down | 0.037543 |
| O94986 | Centrosomal protein of 152 kDa OS=Homo sapiens OX=9606 GN=CEP152 PE=1 SV=4 | CEP152 | 195.62 | | 0.203 | Down | 0.025758 |
| Q8TEP8 | Centrosomal protein of 192 kDa OS=Homo sapiens OX=9606 GN=CEP192 PE=1 SV=3 | CEP192 | 279.11 | | 0.144 | Down | 0.000115718 |
| Q6ZS30 | Neurobeachin-like protein 1 OS=Homo sapiens OX=9606 GN=NBEAL1 PE=2 SV=3 | NBEAL1 | 307.23 | | 0.123 | Down | 0.01354 |

M/N

| **Protein accession** | **Protein description** | **Gene name** | **MW [kDa]** | **M/N Ratio** | **Regulated Type** | **M/N P value** |
| --- | --- | --- | --- | --- | --- | --- |
| Q02985 | Complement factor H-related protein 3 OS=Homo sapiens OX=9606 GN=CFHR3 PE=1 SV=2 | CFHR3 | 37.323 | 3.251 | Up | 0.0067609 |
| Q99878 | Histone H2A type 1-J OS=Homo sapiens OX=9606 GN=HIST1H2AJ PE=1 SV=3 | HIST1H2AJ | 13.936 | 2.475 | Up | 0.04512 |
| P02741 | C-reactive protein OS=Homo sapiens OX=9606 GN=CRP PE=1 SV=1 | CRP | 25.038 | 2.443 | Up | 0.025044 |
| P02792 | Ferritin light chain OS=Homo sapiens OX=9606 GN=FTL PE=1 SV=2 | FTL | 20.019 | 2.268 | Up | 0.000175935 |
| P18428 | Lipopolysaccharide-binding protein OS=Homo sapiens OX=9606 GN=LBP PE=1 SV=3 | LBP | 53.383 | 2.169 | Up | 0.00072385 |
| Q08830 | Fibrinogen-like protein 1 OS=Homo sapiens OX=9606 GN=FGL1 PE=1 SV=3 | FGL1 | 36.379 | 2.076 | Up | 0.00034268 |
| Q15485 | Ficolin-2 OS=Homo sapiens OX=9606 GN=FCN2 PE=1 SV=2 | FCN2 | 34.001 | 1.972 | Up | 0.00074496 |
| Q5T0Z8 | Uncharacterized protein C6orf132 OS=Homo sapiens OX=9606 GN=C6orf132 PE=1 SV=4 | C6orf132 | 124.03 | 1.826 | Up | 0.0026577 |
| P01721 | Immunoglobulin lambda variable 6-57 OS=Homo sapiens OX=9606 GN=IGLV6-57 PE=1 SV=2 | IGLV6-57 | 12.566 | 1.815 | Up | 0.041816 |
| Q14195 | Dihydropyrimidinase-related protein 3 OS=Homo sapiens OX=9606 GN=DPYSL3 PE=1 SV=1 | DPYSL3 | 61.963 | 1.734 | Up | 0.036039 |
| Q9BXR6 | Complement factor H-related protein 5 OS=Homo sapiens OX=9606 GN=CFHR5 PE=1 SV=1 | CFHR5 | 64.419 | 1.686 | Up | 0.000022504 |
| P02748 | Complement component C9 OS=Homo sapiens OX=9606 GN=C9 PE=1 SV=2 | C9 | 63.173 | 1.624 | Up | 0.0048779 |
| P05156 | Complement factor I OS=Homo sapiens OX=9606 GN=CFI PE=1 SV=2 | CFI | 65.75 | 1.608 | Up | 0.0095211 |
| P04275 | von Willebrand factor OS=Homo sapiens OX=9606 GN=VWF PE=1 SV=4 | VWF | 309.26 | 1.588 | Up | 0.0081215 |
| P02675 | Fibrinogen beta chain OS=Homo sapiens OX=9606 GN=FGB PE=1 SV=2 | FGB | 55.928 | 1.552 | Up | 0.0112249 |
| P02679 | Fibrinogen gamma chain OS=Homo sapiens OX=9606 GN=FGG PE=1 SV=3 | FGG | 51.511 | 1.544 | Up | 0.014921 |
| P01031 | Complement C5 OS=Homo sapiens OX=9606 GN=C5 PE=1 SV=4 | C5 | 188.3 | 1.386 | Up | 0.0130407 |
| P05160 | Coagulation factor XIII B chain OS=Homo sapiens OX=9606 GN=F13B PE=1 SV=3 | F13B | 75.51 | 1.369 | Up | 0.0157806 |
| Q9ULI3 | Protein HEG homolog 1 OS=Homo sapiens OX=9606 GN=HEG1 PE=1 SV=3 | HEG1 | 147.46 | 1.363 | Up | 0.032443 |
| P07358 | Complement component C8 beta chain OS=Homo sapiens OX=9606 GN=C8B PE=1 SV=3 | C8B | 67.046 | 1.345 | Up | 0.036355 |
| Q86X52 | Chondroitin sulfate synthase 1 OS=Homo sapiens OX=9606 GN=CHSY1 PE=1 SV=3 | CHSY1 | 91.783 | 1.306 | Up | 0.046379 |
| Q7Z408 | CUB and sushi domain-containing protein 2 OS=Homo sapiens OX=9606 GN=CSMD2 PE=1 SV=2 | CSMD2 | 380.03 | 1.305 | Up | 0.030604 |
| Q9UGM5 | Fetuin-B OS=Homo sapiens OX=9606 GN=FETUB PE=1 SV=2 | FETUB | 42.054 | 1.303 | Up | 0.041963 |
| P04003 | C4b-binding protein alpha chain OS=Homo sapiens OX=9606 GN=C4BPA PE=1 SV=2 | C4BPA | 67.033 | 1.297 | Up | 0.0104752 |
| Q13201 | Multimerin-1 OS=Homo sapiens OX=9606 GN=MMRN1 PE=1 SV=3 | MMRN1 | 138.11 | 1.274 | Up | 0.041359 |
| P07225 | Vitamin K-dependent protein S OS=Homo sapiens OX=9606 GN=PROS1 PE=1 SV=1 | PROS1 | 75.122 | 1.25 | Up | 0.032884 |
| P0C0L4 | Complement C4-A OS=Homo sapiens OX=9606 GN=C4A PE=1 SV=2 | C4A | 192.78 | 1.221 | Up | 0.0198573 |
| O00187 | Mannan-binding lectin serine protease 2 OS=Homo sapiens OX=9606 GN=MASP2 PE=1 SV=4 | MASP2 | 75.702 | 1.211 | Up | 0.022436 |
| P05154 | Plasma serine protease inhibitor OS=Homo sapiens OX=9606 GN=SERPINA5 PE=1 SV=3 | SERPINA5 | 45.674 | 0.823 | Down | 0.0049575 |
| P22748 | Carbonic anhydrase 4 OS=Homo sapiens OX=9606 GN=CA4 PE=1 SV=2 | CA4 | 35.032 | 0.806 | Down | 0.023701 |
| Q9NZM3 | Intersectin-2 OS=Homo sapiens OX=9606 GN=ITSN2 PE=1 SV=3 | ITSN2 | 193.46 | 0.762 | Down | 0.0082188 |
| P0DP04 | Immunoglobulin heavy variable 3-43D OS=Homo sapiens OX=9606 GN=IGHV3-43D PE=3 SV=1 | IGHV3-43D | 13.017 | 0.756 | Down | 0.0195152 |
| Q9NQ79 | Cartilage acidic protein 1 OS=Homo sapiens OX=9606 GN=CRTAC1 PE=1 SV=2 | CRTAC1 | 71.42 | 0.754 | Down | 0.00164312 |
| P07948 | Tyrosine-protein kinase Lyn OS=Homo sapiens OX=9606 GN=LYN PE=1 SV=3 | LYN | 58.573 | 0.747 | Down | 0.022044 |
| Q9HD89 | Resistin OS=Homo sapiens OX=9606 GN=RETN PE=1 SV=1 | RETN | 11.419 | 0.743 | Down | 0.026661 |
| P55072 | Transitional endoplasmic reticulum ATPase OS=Homo sapiens OX=9606 GN=VCP PE=1 SV=4 | VCP | 89.321 | 0.737 | Down | 0.030221 |
| P40227 | T-complex protein 1 subunit zeta OS=Homo sapiens OX=9606 GN=CCT6A PE=1 SV=3 | CCT6A | 58.024 | 0.729 | Down | 0.0162042 |
| P60953 | Cell division control protein 42 homolog OS=Homo sapiens OX=9606 GN=CDC42 PE=1 SV=2 | CDC42 | 21.258 | 0.729 | Down | 0.023859 |
| P30685 | "HLA class I histocompatibility antigen, B-35 alpha chain OS=Homo sapiens OX=9606 GN=HLA-B PE=1 SV=1" | HLA-B | 40.455 | 0.726 | Down | 0.041544 |
| Q6ZRY4 | RNA-binding protein with multiple splicing 2 OS=Homo sapiens OX=9606 GN=RBPMS2 PE=1 SV=1 | RBPMS2 | 22.496 | 0.722 | Down | 0.033001 |
| P43652 | Afamin OS=Homo sapiens OX=9606 GN=AFM PE=1 SV=1 | AFM | 69.068 | 0.696 | Down | 0.023685 |
| P04211 | Immunoglobulin lambda variable 7-43 OS=Homo sapiens OX=9606 GN=IGLV7-43 PE=3 SV=2 | IGLV7-43 | 12.451 | 0.678 | Down | 0.034522 |
| O75116 | Rho-associated protein kinase 2 OS=Homo sapiens OX=9606 GN=ROCK2 PE=1 SV=4 | ROCK2 | 160.9 | 0.672 | Down | 0.0106779 |
| P26447 | Protein S100-A4 OS=Homo sapiens OX=9606 GN=S100A4 PE=1 SV=1 | S100A4 | 11.728 | 0.672 | Down | 0.048956 |
| Q8WVV4 | Protein POF1B OS=Homo sapiens OX=9606 GN=POF1B PE=1 SV=3 | POF1B | 68.064 | 0.672 | Down | 0.022805 |
| P08758 | Annexin A5 OS=Homo sapiens OX=9606 GN=ANXA5 PE=1 SV=2 | ANXA5 | 35.936 | 0.671 | Down | 0.047076 |
| P55160 | Nck-associated protein 1-like OS=Homo sapiens OX=9606 GN=NCKAP1L PE=1 SV=3 | NCKAP1L | 128.15 | 0.653 | Down | 0.026185 |
| Q8TBC5 | Zinc finger and SCAN domain-containing protein 18 OS=Homo sapiens OX=9606 GN=ZSCAN18 PE=2 SV=2 | ZSCAN18 | 54.803 | 0.642 | Down | 0.034563 |
| Q96QZ7 | "Membrane-associated guanylate kinase, WW and PDZ domain-containing protein 1 OS=Homo sapiens OX=9606 GN=MAGI1 PE=1 SV=3" | MAGI1 | 164.58 | 0.642 | Down | 0.0171611 |
| A0A075B6I0 | Immunoglobulin lambda variable 8-61 OS=Homo sapiens OX=9606 GN=IGLV8-61 PE=3 SV=7 | IGLV8-61 | 12.814 | 0.641 | Down | 0.037302 |
| P07195 | L-lactate dehydrogenase B chain OS=Homo sapiens OX=9606 GN=LDHB PE=1 SV=2 | LDHB | 36.638 | 0.634 | Down | 0.025841 |
| Q8IYT4 | Katanin p60 ATPase-containing subunit A-like 2 OS=Homo sapiens OX=9606 GN=KATNAL2 PE=1 SV=3 | KATNAL2 | 61.252 | 0.63 | Down | 0.0021773 |
| P01602 | Immunoglobulin kappa variable 1-5 OS=Homo sapiens OX=9606 GN=IGKV1-5 PE=1 SV=2 | IGKV1-5 | 12.781 | 0.607 | Down | 0.029557 |
| P12532 | "Creatine kinase U-type, mitochondrial OS=Homo sapiens OX=9606 GN=CKMT1A PE=1 SV=1" | CKMT1A | 47.036 | 0.605 | Down | 0.0004159 |
| P10124 | Serglycin OS=Homo sapiens OX=9606 GN=SRGN PE=1 SV=3 | SRGN | 17.652 | 0.601 | Down | 0.020363 |
| Q9H0K1 | Serine/threonine-protein kinase SIK2 OS=Homo sapiens OX=9606 GN=SIK2 PE=1 SV=1 | SIK2 | 103.91 | 0.577 | Down | 0.0067436 |
| Q6YHK3 | CD109 antigen OS=Homo sapiens OX=9606 GN=CD109 PE=1 SV=2 | CD109 | 161.69 | 0.57 | Down | 0.020544 |
| Q99715 | Collagen alpha-1(XII) chain OS=Homo sapiens OX=9606 GN=COL12A1 PE=1 SV=2 | COL12A1 | 333.14 | 0.506 | Down | 0.0157413 |

N/A

| **Protein accession** | **Protein description** | **Gene name** | **MW [kDa]** | **N/A Ratio** | **Regulated Type** | **N/A P value** |
| --- | --- | --- | --- | --- | --- | --- |
| Q5JV73 | FERM and PDZ domain-containing protein 3 OS=Homo sapiens OX=9606 GN=FRMPD3 PE=2 SV=2 | FRMPD3 | 199.21 | 3.474 | Up | 0.00022003 |
| Q86U86 | Protein polybromo-1 OS=Homo sapiens OX=9606 GN=PBRM1 PE=1 SV=1 | PBRM1 | 192.95 | 3.184 | Up | 0.046497 |
| Q9NQ76 | Matrix extracellular phosphoglycoprotein OS=Homo sapiens OX=9606 GN=MEPE PE=1 SV=1 | MEPE | 58.418 | 2.934 | Up | 0.044356 |
| O14513 | Nck-associated protein 5 OS=Homo sapiens OX=9606 GN=NCKAP5 PE=1 SV=2 | NCKAP5 | 208.53 | 2.748 | Up | 0.044099 |
| Q96LP6 | Uncharacterized protein C12orf42 OS=Homo sapiens OX=9606 GN=C12orf42 PE=2 SV=2 | C12orf42 | 39.738 | 2.501 | Up | 0.0095983 |
| Q6ZUS5 | Coiled-coil domain-containing protein 121 OS=Homo sapiens OX=9606 GN=CCDC121 PE=1 SV=1 | CCDC121 | 33.06 | 2.297 | Up | 9.4587E-07 |
| Q16610 | Extracellular matrix protein 1 OS=Homo sapiens OX=9606 GN=ECM1 PE=1 SV=2 | ECM1 | 60.673 | 2.021 | Up | 0.000104088 |
| O60508 | Pre-mRNA-processing factor 17 OS=Homo sapiens OX=9606 GN=CDC40 PE=1 SV=1 | CDC40 | 65.521 | 1.993 | Up | 0.0162637 |
| P02671 | Fibrinogen alpha chain OS=Homo sapiens OX=9606 GN=FGA PE=1 SV=2 | FGA | 94.972 | 1.984 | Up | 8.5724E-07 |
| P32456 | Guanylate-binding protein 2 OS=Homo sapiens OX=9606 GN=GBP2 PE=1 SV=3 | GBP2 | 67.208 | 1.983 | Up | 0.022763 |
| Q5T619 | Zinc finger protein 648 OS=Homo sapiens OX=9606 GN=ZNF648 PE=2 SV=1 | ZNF648 | 62.34 | 1.928 | Up | 0.0091002 |
| Q7Z408 | CUB and sushi domain-containing protein 2 OS=Homo sapiens OX=9606 GN=CSMD2 PE=1 SV=2 | CSMD2 | 380.03 | 1.86 | Up | 0.00021926 |
| P08697 | Alpha-2-antiplasmin OS=Homo sapiens OX=9606 GN=SERPINF2 PE=1 SV=3 | SERPINF2 | 54.565 | 1.843 | Up | 0.0029049 |
| O75747 | Phosphatidylinositol 4-phosphate 3-kinase C2 domain-containing subunit gamma OS=Homo sapiens OX=9606 GN=PIK3C2G PE=1 SV=3 | PIK3C2G | 165.71 | 1.814 | Up | 0.026583 |
| P02679 | Fibrinogen gamma chain OS=Homo sapiens OX=9606 GN=FGG PE=1 SV=3 | FGG | 51.511 | 1.799 | Up | 0.00064391 |
| Q9BXR5 | Toll-like receptor 10 OS=Homo sapiens OX=9606 GN=TLR10 PE=1 SV=2 | TLR10 | 94.563 | 1.792 | Up | 0.0131974 |
| P03973 | Antileukoproteinase OS=Homo sapiens OX=9606 GN=SLPI PE=1 SV=2 | SLPI | 14.326 | 1.773 | Up | 0.0107649 |
| P05543 | Thyroxine-binding globulin OS=Homo sapiens OX=9606 GN=SERPINA7 PE=1 SV=2 | SERPINA7 | 46.324 | 1.744 | Up | 0.0080439 |
| Q12805 | EGF-containing fibulin-like extracellular matrix protein 1 OS=Homo sapiens OX=9606 GN=EFEMP1 PE=1 SV=2 | EFEMP1 | 54.64 | 1.731 | Up | 0.0137847 |
| P02675 | Fibrinogen beta chain OS=Homo sapiens OX=9606 GN=FGB PE=1 SV=2 | FGB | 55.928 | 1.722 | Up | 0.00048457 |
| P02452 | Collagen alpha-1(I) chain OS=Homo sapiens OX=9606 GN=COL1A1 PE=1 SV=5 | COL1A1 | 138.94 | 1.695 | Up | 0.026142 |
| P10909 | Clusterin OS=Homo sapiens OX=9606 GN=CLU PE=1 SV=1 | CLU | 52.494 | 1.694 | Up | 0.000042332 |
| P00488 | Coagulation factor XIII A chain OS=Homo sapiens OX=9606 GN=F13A1 PE=1 SV=4 | F13A1 | 83.266 | 1.69 | Up | 0.00095684 |
| Q8TBC5 | Zinc finger and SCAN domain-containing protein 18 OS=Homo sapiens OX=9606 GN=ZSCAN18 PE=2 SV=2 | ZSCAN18 | 54.803 | 1.659 | Up | 0.030597 |
| Q99592 | Zinc finger and BTB domain-containing protein 18 OS=Homo sapiens OX=9606 GN=ZBTB18 PE=1 SV=1 | ZBTB18 | 58.354 | 1.658 | Up | 0.0199017 |
| Q9Y6D5 | Brefeldin A-inhibited guanine nucleotide-exchange protein 2 OS=Homo sapiens OX=9606 GN=ARFGEF2 PE=1 SV=3 | ARFGEF2 | 202.04 | 1.632 | Up | 0.035341 |
| P04275 | von Willebrand factor OS=Homo sapiens OX=9606 GN=VWF PE=1 SV=4 | VWF | 309.26 | 1.607 | Up | 0.0133226 |
| P01040 | Cystatin-A OS=Homo sapiens OX=9606 GN=CSTA PE=1 SV=1 | CSTA | 11.006 | 1.606 | Up | 0.028342 |
| Q5T749 | Keratinocyte proline-rich protein OS=Homo sapiens OX=9606 GN=KPRP PE=1 SV=1 | KPRP | 64.135 | 1.599 | Up | 0.0082021 |
| Q6ZS17 | Rho family-interacting cell polarization regulator 1 OS=Homo sapiens OX=9606 GN=RIPOR1 PE=1 SV=1 | RIPOR1 | 132.31 | 1.58 | Up | 0.0096615 |
| Q99969 | Retinoic acid receptor responder protein 2 OS=Homo sapiens OX=9606 GN=RARRES2 PE=1 SV=1 | RARRES2 | 18.617 | 1.578 | Up | 0.022443 |
| Q9BZW5 | Transmembrane 6 superfamily member 1 OS=Homo sapiens OX=9606 GN=TM6SF1 PE=1 SV=2 | TM6SF1 | 41.636 | 1.541 | Up | 0.0111014 |
| P57739 | Claudin-2 OS=Homo sapiens OX=9606 GN=CLDN2 PE=1 SV=1 | CLDN2 | 24.548 | 1.54 | Up | 0.0054431 |
| Q15120 | "[Pyruvate dehydrogenase (acetyl-transferring)] kinase isozyme 3, mitochondrial OS=Homo sapiens OX=9606 GN=PDK3 PE=1 SV=1" | PDK3 | 46.938 | 1.528 | Up | 0.021396 |
| Q9Y2I8 | WD repeat-containing protein 37 OS=Homo sapiens OX=9606 GN=WDR37 PE=1 SV=2 | WDR37 | 54.665 | 1.517 | Up | 0.044121 |
| P02792 | Ferritin light chain OS=Homo sapiens OX=9606 GN=FTL PE=1 SV=2 | FTL | 20.019 | 1.497 | Up | 0.0035825 |
| O15078 | Centrosomal protein of 290 kDa OS=Homo sapiens OX=9606 GN=CEP290 PE=1 SV=2 | CEP290 | 290.38 | 1.474 | Up | 0.022684 |
| Q02413 | Desmoglein-1 OS=Homo sapiens OX=9606 GN=DSG1 PE=1 SV=2 | DSG1 | 113.75 | 1.471 | Up | 0.0067165 |
| P02655 | Apolipoprotein C-II OS=Homo sapiens OX=9606 GN=APOC2 PE=1 SV=1 | APOC2 | 11.284 | 1.445 | Up | 0.049695 |
| O00602 | Ficolin-1 OS=Homo sapiens OX=9606 GN=FCN1 PE=1 SV=2 | FCN1 | 35.078 | 1.44 | Up | 0.002843 |
| Q13867 | Bleomycin hydrolase OS=Homo sapiens OX=9606 GN=BLMH PE=1 SV=1 | BLMH | 52.562 | 1.434 | Up | 0.0141811 |
| P98160 | Basement membrane-specific heparan sulfate proteoglycan core protein OS=Homo sapiens OX=9606 GN=HSPG2 PE=1 SV=4 | HSPG2 | 468.83 | 1.433 | Up | 0.00069683 |
| P11047 | Laminin subunit gamma-1 OS=Homo sapiens OX=9606 GN=LAMC1 PE=1 SV=3 | LAMC1 | 177.6 | 1.433 | Up | 0.0067985 |
| P02656 | Apolipoprotein C-III OS=Homo sapiens OX=9606 GN=APOC3 PE=1 SV=1 | APOC3 | 10.852 | 1.428 | Up | 0.023956 |
| P15924 | Desmoplakin OS=Homo sapiens OX=9606 GN=DSP PE=1 SV=3 | DSP | 331.77 | 1.411 | Up | 0.00159855 |
| P02766 | Transthyretin OS=Homo sapiens OX=9606 GN=TTR PE=1 SV=1 | TTR | 15.887 | 1.401 | Up | 0.026042 |
| P31944 | Caspase-14 OS=Homo sapiens OX=9606 GN=CASP14 PE=1 SV=2 | CASP14 | 27.679 | 1.396 | Up | 0.023395 |
| P15170 | Eukaryotic peptide chain release factor GTP-binding subunit ERF3A OS=Homo sapiens OX=9606 GN=GSPT1 PE=1 SV=1 | GSPT1 | 55.755 | 1.391 | Up | 0.0058419 |
| P08238 | Heat shock protein HSP 90-beta OS=Homo sapiens OX=9606 GN=HSP90AB1 PE=1 SV=4 | HSP90AB1 | 83.263 | 1.386 | Up | 0.0129797 |
| Q9Y2R2 | Tyrosine-protein phosphatase non-receptor type 22 OS=Homo sapiens OX=9606 GN=PTPN22 PE=1 SV=2 | PTPN22 | 91.704 | 1.385 | Up | 0.0048392 |
| P28715 | DNA repair protein complementing XP-G cells OS=Homo sapiens OX=9606 GN=ERCC5 PE=1 SV=3 | ERCC5 | 133.11 | 1.362 | Up | 0.0163969 |
| Q6UWP8 | Suprabasin OS=Homo sapiens OX=9606 GN=SBSN PE=1 SV=2 | SBSN | 60.54 | 1.359 | Up | 0.0126179 |
| Q8WWZ8 | Oncoprotein-induced transcript 3 protein OS=Homo sapiens OX=9606 GN=OIT3 PE=1 SV=2 | OIT3 | 60.021 | 1.357 | Up | 0.038115 |
| P61088 | Ubiquitin-conjugating enzyme E2 N OS=Homo sapiens OX=9606 GN=UBE2N PE=1 SV=1 | UBE2N | 17.138 | 1.356 | Up | 0.025144 |
| P02790 | Hemopexin OS=Homo sapiens OX=9606 GN=HPX PE=1 SV=2 | HPX | 51.676 | 1.348 | Up | 0.025743 |
| Q08554 | Desmocollin-1 OS=Homo sapiens OX=9606 GN=DSC1 PE=1 SV=2 | DSC1 | 99.986 | 1.346 | Up | 0.0139646 |
| P04004 | Vitronectin OS=Homo sapiens OX=9606 GN=VTN PE=1 SV=1 | VTN | 54.305 | 1.345 | Up | 0.0148394 |
| Q14766 | Latent-transforming growth factor beta-binding protein 1 OS=Homo sapiens OX=9606 GN=LTBP1 PE=1 SV=4 | LTBP1 | 186.79 | 1.307 | Up | 0.032939 |
| P01008 | Antithrombin-III OS=Homo sapiens OX=9606 GN=SERPINC1 PE=1 SV=1 | SERPINC1 | 52.602 | 1.3 | Up | 0.028881 |
| Q15517 | Corneodesmosin OS=Homo sapiens OX=9606 GN=CDSN PE=1 SV=3 | CDSN | 51.522 | 1.296 | Up | 0.045977 |
| Q9NZM3 | Intersectin-2 OS=Homo sapiens OX=9606 GN=ITSN2 PE=1 SV=3 | ITSN2 | 193.46 | 1.278 | Up | 0.0090045 |
| P00746 | Complement factor D OS=Homo sapiens OX=9606 GN=CFD PE=1 SV=5 | CFD | 27.033 | 1.27 | Up | 0.0084976 |
| P05154 | Plasma serine protease inhibitor OS=Homo sapiens OX=9606 GN=SERPINA5 PE=1 SV=3 | SERPINA5 | 45.674 | 1.264 | Up | 0.00072047 |
| Q8NI99 | Angiopoietin-related protein 6 OS=Homo sapiens OX=9606 GN=ANGPTL6 PE=1 SV=1 | ANGPTL6 | 51.694 | 1.244 | Up | 0.0146638 |
| P04217 | Alpha-1B-glycoprotein OS=Homo sapiens OX=9606 GN=A1BG PE=1 SV=4 | A1BG | 54.253 | 1.204 | Up | 0.041942 |
| P49796 | Regulator of G-protein signaling 3 OS=Homo sapiens OX=9606 GN=RGS3 PE=1 SV=2 | RGS3 | 132.33 | 0.831 | Down | 0.031964 |
| P04406 | Glyceraldehyde-3-phosphate dehydrogenase OS=Homo sapiens OX=9606 GN=GAPDH PE=1 SV=3 | GAPDH | 36.053 | 0.814 | Down | 0.043157 |
| Q99567 | Nuclear pore complex protein Nup88 OS=Homo sapiens OX=9606 GN=NUP88 PE=1 SV=2 | NUP88 | 83.541 | 0.805 | Down | 0.033236 |
| Q9HC84 | Mucin-5B OS=Homo sapiens OX=9606 GN=MUC5B PE=1 SV=3 | MUC5B | 596.33 | 0.795 | Down | 0.032843 |
| P08603 | Complement factor H OS=Homo sapiens OX=9606 GN=CFH PE=1 SV=4 | CFH | 139.09 | 0.791 | Down | 0.0140824 |
| P01834 | Immunoglobulin kappa constant OS=Homo sapiens OX=9606 GN=IGKC PE=1 SV=2 | IGKC | 11.765 | 0.773 | Down | 0.041864 |
| A0A0C4DH33 | Immunoglobulin heavy variable 1-24 OS=Homo sapiens OX=9606 GN=IGHV1-24 PE=3 SV=1 | IGHV1-24 | 12.824 | 0.772 | Down | 0.0199196 |
| P02647 | Apolipoprotein A-I OS=Homo sapiens OX=9606 GN=APOA1 PE=1 SV=1 | APOA1 | 30.777 | 0.771 | Down | 0.0051418 |
| P01594 | Immunoglobulin kappa variable 1-33 OS=Homo sapiens OX=9606 GN=IGKV1-33 PE=1 SV=2 | IGKV1-33 | 12.848 | 0.76 | Down | 0.046704 |
| P01871 | Immunoglobulin heavy constant mu OS=Homo sapiens OX=9606 GN=IGHM PE=1 SV=4 | IGHM | 49.439 | 0.76 | Down | 0.023459 |
| P14770 | Platelet glycoprotein IX OS=Homo sapiens OX=9606 GN=GP9 PE=1 SV=3 | GP9 | 19.046 | 0.757 | Down | 0.03482 |
| Q15389 | Angiopoietin-1 OS=Homo sapiens OX=9606 GN=ANGPT1 PE=1 SV=2 | ANGPT1 | 57.512 | 0.755 | Down | 0.039336 |
| P0DOX5 | Immunoglobulin gamma-1 heavy chain OS=Homo sapiens OX=9606 PE=1 SV=2 | --- | 49.328 | 0.747 | Down | 0.020197 |
| Q14697 | Neutral alpha-glucosidase AB OS=Homo sapiens OX=9606 GN=GANAB PE=1 SV=3 | GANAB | 106.87 | 0.742 | Down | 0.035576 |
| Q04756 | Hepatocyte growth factor activator OS=Homo sapiens OX=9606 GN=HGFAC PE=1 SV=1 | HGFAC | 70.681 | 0.741 | Down | 0.0110645 |
| P04075 | Fructose-bisphosphate aldolase A OS=Homo sapiens OX=9606 GN=ALDOA PE=1 SV=2 | ALDOA | 39.42 | 0.732 | Down | 0.035764 |
| P01833 | Polymeric immunoglobulin receptor OS=Homo sapiens OX=9606 GN=PIGR PE=1 SV=4 | PIGR | 83.283 | 0.731 | Down | 0.048184 |
| A0A0B4J1Y9 | Immunoglobulin heavy variable 3-72 OS=Homo sapiens OX=9606 GN=IGHV3-72 PE=3 SV=1 | IGHV3-72 | 13.203 | 0.724 | Down | 0.0182995 |
| P0DOX2 | Immunoglobulin alpha-2 heavy chain OS=Homo sapiens OX=9606 PE=1 SV=2 | --- | 48.934 | 0.723 | Down | 0.043297 |
| Q9P2P6 | StAR-related lipid transfer protein 9 OS=Homo sapiens OX=9606 GN=STARD9 PE=1 SV=3 | STARD9 | 516.34 | 0.719 | Down | 0.045298 |
| A0A0C4DH29 | Immunoglobulin heavy variable 1-3 OS=Homo sapiens OX=9606 GN=IGHV1-3 PE=3 SV=1 | IGHV1-3 | 13.008 | 0.719 | Down | 0.0108234 |
| O60832 | H/ACA ribonucleoprotein complex subunit DKC1 OS=Homo sapiens OX=9606 GN=DKC1 PE=1 SV=3 | DKC1 | 57.673 | 0.71 | Down | 0.045957 |
| Q5VTJ3 | Kelch domain-containing protein 7A OS=Homo sapiens OX=9606 GN=KLHDC7A PE=1 SV=5 | KLHDC7A | 84.478 | 0.709 | Down | 0.020479 |
| P01031 | Complement C5 OS=Homo sapiens OX=9606 GN=C5 PE=1 SV=4 | C5 | 188.3 | 0.708 | Down | 0.0042159 |
| P09543 | "2',3'-cyclic-nucleotide 3'-phosphodiesterase OS=Homo sapiens OX=9606 GN=CNP PE=1 SV=2" | CNP | 47.578 | 0.695 | Down | 0.034963 |
| A0A075B6K5 | Immunoglobulin lambda variable 3-9 OS=Homo sapiens OX=9606 GN=IGLV3-9 PE=3 SV=1 | IGLV3-9 | 12.332 | 0.687 | Down | 0.0151577 |
| O75083 | WD repeat-containing protein 1 OS=Homo sapiens OX=9606 GN=WDR1 PE=1 SV=4 | WDR1 | 66.193 | 0.678 | Down | 0.021116 |
| Q13418 | Integrin-linked protein kinase OS=Homo sapiens OX=9606 GN=ILK PE=1 SV=2 | ILK | 51.419 | 0.676 | Down | 0.044795 |
| A0A0A0MS14 | Immunoglobulin heavy variable 1-45 OS=Homo sapiens OX=9606 GN=IGHV1-45 PE=3 SV=1 | IGHV1-45 | 13.508 | 0.673 | Down | 0.01006 |
| Q9UQB8 | Brain-specific angiogenesis inhibitor 1-associated protein 2 OS=Homo sapiens OX=9606 GN=BAIAP2 PE=1 SV=1 | BAIAP2 | 60.867 | 0.672 | Down | 0.021437 |
| P04211 | Immunoglobulin lambda variable 7-43 OS=Homo sapiens OX=9606 GN=IGLV7-43 PE=3 SV=2 | IGLV7-43 | 12.451 | 0.664 | Down | 0.027057 |
| Q96QZ7 | "Membrane-associated guanylate kinase, WW and PDZ domain-containing protein 1 OS=Homo sapiens OX=9606 GN=MAGI1 PE=1 SV=3" | MAGI1 | 164.58 | 0.662 | Down | 0.0180357 |
| A0A0C4DH68 | Immunoglobulin kappa variable 2-24 OS=Homo sapiens OX=9606 GN=IGKV2-24 PE=3 SV=1 | IGKV2-24 | 13.079 | 0.658 | Down | 0.0188039 |
| P19652 | Alpha-1-acid glycoprotein 2 OS=Homo sapiens OX=9606 GN=ORM2 PE=1 SV=2 | ORM2 | 23.602 | 0.656 | Down | 0.007562 |
| O75558 | Syntaxin-11 OS=Homo sapiens OX=9606 GN=STX11 PE=1 SV=1 | STX11 | 33.195 | 0.651 | Down | 0.023576 |
| P13224 | Platelet glycoprotein Ib beta chain OS=Homo sapiens OX=9606 GN=GP1BB PE=1 SV=1 | GP1BB | 21.717 | 0.647 | Down | 0.03588 |
| P01019 | Angiotensinogen OS=Homo sapiens OX=9606 GN=AGT PE=1 SV=1 | AGT | 53.154 | 0.639 | Down | 0.0114982 |
| P06312 | Immunoglobulin kappa variable 4-1 OS=Homo sapiens OX=9606 GN=IGKV4-1 PE=1 SV=1 | IGKV4-1 | 13.38 | 0.638 | Down | 0.00190118 |
| P01876 | Immunoglobulin heavy constant alpha 1 OS=Homo sapiens OX=9606 GN=IGHA1 PE=1 SV=2 | IGHA1 | 37.654 | 0.635 | Down | 0.00053638 |
| Q3L8U1 | Chromodomain-helicase-DNA-binding protein 9 OS=Homo sapiens OX=9606 GN=CHD9 PE=1 SV=2 | CHD9 | 326.02 | 0.635 | Down | 0.0151982 |
| P01780 | Immunoglobulin heavy variable 3-7 OS=Homo sapiens OX=9606 GN=IGHV3-7 PE=1 SV=2 | IGHV3-7 | 12.943 | 0.634 | Down | 0.0026759 |
| O15145 | Actin-related protein 2/3 complex subunit 3 OS=Homo sapiens OX=9606 GN=ARPC3 PE=1 SV=3 | ARPC3 | 20.546 | 0.634 | Down | 0.029657 |
| P0DOX8 | Immunoglobulin lambda-1 light chain OS=Homo sapiens OX=9606 PE=1 SV=1 | --- | 22.83 | 0.631 | Down | 0.0024997 |
| Q9HD89 | Resistin OS=Homo sapiens OX=9606 GN=RETN PE=1 SV=1 | RETN | 11.419 | 0.628 | Down | 0.018361 |
| Q9NR20 | Dual specificity tyrosine-phosphorylation-regulated kinase 4 OS=Homo sapiens OX=9606 GN=DYRK4 PE=1 SV=2 | DYRK4 | 59.608 | 0.623 | Down | 0.025844 |
| P59998 | Actin-related protein 2/3 complex subunit 4 OS=Homo sapiens OX=9606 GN=ARPC4 PE=1 SV=3 | ARPC4 | 19.667 | 0.62 | Down | 0.039236 |
| O15162 | Phospholipid scramblase 1 OS=Homo sapiens OX=9606 GN=PLSCR1 PE=1 SV=1 | PLSCR1 | 35.049 | 0.619 | Down | 0.026076 |
| P01602 | Immunoglobulin kappa variable 1-5 OS=Homo sapiens OX=9606 GN=IGKV1-5 PE=1 SV=2 | IGKV1-5 | 12.781 | 0.617 | Down | 0.041578 |
| O14791 | Apolipoprotein L1 OS=Homo sapiens OX=9606 GN=APOL1 PE=1 SV=5 | APOL1 | 43.974 | 0.614 | Down | 0.0022796 |
| A0A0B4J1X8 | Immunoglobulin heavy variable 3-43 OS=Homo sapiens OX=9606 GN=IGHV3-43 PE=3 SV=1 | IGHV3-43 | 13.077 | 0.613 | Down | 0.026144 |
| Q99880 | Histone H2B type 1-L OS=Homo sapiens OX=9606 GN=HIST1H2BL PE=1 SV=3 | HIST1H2BL | 13.952 | 0.607 | Down | 0.034179 |
| P01709 | Immunoglobulin lambda variable 2-8 OS=Homo sapiens OX=9606 GN=IGLV2-8 PE=1 SV=2 | IGLV2-8 | 12.382 | 0.592 | Down | 0.00185965 |
| Q6UW60 | Proprotein convertase subtilisin/kexin type 4 OS=Homo sapiens OX=9606 GN=PCSK4 PE=1 SV=2 | PCSK4 | 82.794 | 0.591 | Down | 0.0069394 |
| P00739 | Haptoglobin-related protein OS=Homo sapiens OX=9606 GN=HPR PE=2 SV=2 | HPR | 39.029 | 0.581 | Down | 0.00047517 |
| O43149 | Zinc finger ZZ-type and EF-hand domain-containing protein 1 OS=Homo sapiens OX=9606 GN=ZZEF1 PE=1 SV=6 | ZZEF1 | 331.07 | 0.564 | Down | 0.0109821 |
| P23528 | Cofilin-1 OS=Homo sapiens OX=9606 GN=CFL1 PE=1 SV=3 | CFL1 | 18.502 | 0.56 | Down | 0.0162419 |
| Q02224 | Centromere-associated protein E OS=Homo sapiens OX=9606 GN=CENPE PE=1 SV=2 | CENPE | 316.41 | 0.558 | Down | 0.028925 |
| Q9UPN3 | "Microtubule-actin cross-linking factor 1, isoforms 1/2/3/5 OS=Homo sapiens OX=9606 GN=MACF1 PE=1 SV=4" | MACF1 | 838.3 | 0.551 | Down | 0.020977 |
| Q14683 | Structural maintenance of chromosomes protein 1A OS=Homo sapiens OX=9606 GN=SMC1A PE=1 SV=2 | SMC1A | 143.23 | 0.549 | Down | 0.0058973 |
| Q9UPX8 | SH3 and multiple ankyrin repeat domains protein 2 OS=Homo sapiens OX=9606 GN=SHANK2 PE=1 SV=3 | SHANK2 | 158.82 | 0.545 | Down | 0.0006228 |
| P06702 | Protein S100-A9 OS=Homo sapiens OX=9606 GN=S100A9 PE=1 SV=1 | S100A9 | 13.242 | 0.54 | Down | 0.0151637 |
| P10720 | Platelet factor 4 variant OS=Homo sapiens OX=9606 GN=PF4V1 PE=1 SV=1 | PF4V1 | 11.553 | 0.528 | Down | 0.00197818 |
| O15212 | Prefoldin subunit 6 OS=Homo sapiens OX=9606 GN=PFDN6 PE=1 SV=1 | PFDN6 | 14.582 | 0.508 | Down | 0.022179 |
| Q6P158 | Putative ATP-dependent RNA helicase DHX57 OS=Homo sapiens OX=9606 GN=DHX57 PE=1 SV=2 | DHX57 | 155.6 | 0.507 | Down | 0.038782 |
| Q9Y4P3 | Transducin beta-like protein 2 OS=Homo sapiens OX=9606 GN=TBL2 PE=1 SV=1 | TBL2 | 49.797 | 0.481 | Down | 0.0183778 |
| Q71U36 | Tubulin alpha-1A chain OS=Homo sapiens OX=9606 GN=TUBA1A PE=1 SV=1 | TUBA1A | 50.135 | 0.477 | Down | 0.0173835 |
| P07737 | Profilin-1 OS=Homo sapiens OX=9606 GN=PFN1 PE=1 SV=2 | PFN1 | 15.054 | 0.445 | Down | 0.00145787 |
| Q96AJ9 | Vesicle transport through interaction with t-SNAREs homolog 1A OS=Homo sapiens OX=9606 GN=VTI1A PE=1 SV=2 | VTI1A | 25.217 | 0.433 | Down | 0.0121448 |
| O00231 | 26S proteasome non-ATPase regulatory subunit 11 OS=Homo sapiens OX=9606 GN=PSMD11 PE=1 SV=3 | PSMD11 | 47.463 | 0.425 | Down | 0.024378 |
| O95841 | Angiopoietin-related protein 1 OS=Homo sapiens OX=9606 GN=ANGPTL1 PE=2 SV=1 | ANGPTL1 | 56.719 | 0.405 | Down | 0.0107566 |
| A0A0B4J1X5 | Immunoglobulin heavy variable 3-74 OS=Homo sapiens OX=9606 GN=IGHV3-74 PE=3 SV=1 | IGHV3-74 | 12.839 | 0.397 | Down | 0.036259 |
| Q9ULD9 | Zinc finger protein 608 OS=Homo sapiens OX=9606 GN=ZNF608 PE=1 SV=4 | ZNF608 | 162.21 | 0.386 | Down | 0.00086277 |
| Q99878 | Histone H2A type 1-J OS=Homo sapiens OX=9606 GN=HIST1H2AJ PE=1 SV=3 | HIST1H2AJ | 13.936 | 0.281 | Down | 0.026616 |
| O15049 | NEDD4-binding protein 3 OS=Homo sapiens OX=9606 GN=N4BP3 PE=1 SV=3 | N4BP3 | 60.469 | 0.18 | Down | 0.0057238 |
| Q2M243 | Coiled-coil domain-containing protein 27 OS=Homo sapiens OX=9606 GN=CCDC27 PE=2 SV=2 | CCDC27 | 75.354 | 0.172 | Down | 0.00139945 |
| Q6ZS30 | Neurobeachin-like protein 1 OS=Homo sapiens OX=9606 GN=NBEAL1 PE=2 SV=3 | NBEAL1 | 307.23 | 0.123 | Down | 0.0043444 |
