# Supplementary material for: Identification of exosome protein panels as predictive biomarkers for non-small cell lung cancer
Source: Biol Proced Online. 2023 Nov 13;25:29. doi: 10.1186/s12575-023-00223-0 (PMC10641949; doi:10.1186/s12575-023-00223-0)
Supplement: Supplementary file 5 — Additional file 5. Detailed analysis steps of bioinformatics. [file 12575_2023_223_MOESM5_ESM.doc]

Supporting information-detailed analysis steps of bioinformatics

Protein-protein interaction network: all differentially expressed protein accession number or sequence were searched against the STRING database version 10.1 for protein-protein interactions. Only interactions between the proteins belonging to the searched data set were selected, thereby excluding external candidates. STRING defines a metric called “confidence score” to define interaction confidence. We fetched all interactions that had a confidence score ≥ 0.7 (high confidence). Interaction network form STRING was visualized in R package “networkD3”.

Enrichment-based Clustering: For further hierarchical clustering of functional classification for differentially expressed protein (such as domain, pathway, complex), we first collated all the categories obtained after enrichment along with their P values, and then filtered for those categories which were at least enriched in one of the clusters with P value <0.05. This filtered P value matrix was transformed by the function x = −log10 (P value). Finally, these x values were z-transformed for each functional category. These z scores were then clustered by one-way hierarchical clustering (Euclidean distance, average linkage clustering) in Genesis. Cluster membership was visualized by a heat map using the “heatmap.2” function from the “g-plots” R-package.

Enrichment of gene ontology (GO) and kyoto encyclopedia of genes and genomes (KEGG) analysis: Proteins were classified by GO annotation into three categories: biological process, cellular compartment, and molecular function. For each category, a two-tailed Fisher’s exact test was employed to test the enrichment of the differentially expressed protein against all identified proteins. The KEGG pathways were classified into hierarchical categories according to the KEGG website. The pathway with a corrected p-value < 0.05 was considered significant.

Software in the bioinformatics analysis

| **Analyst methods** | **Software** | **Version** |
| --- | --- | --- |
| MS/MS data analysis | MaxQuant | v.1.5.2.8 <http://www.maxquant.org/> |
| Motif analysis | MoMo | V5.0.2 <http://meme-suite.org/tools/momo> |
| GO analysis | InterProScan | v.5.14-53.0 <http://www.ebi.ac.uk/interpro/> |
| Domain analysis | InterProScan | v.5.14-53.0 <http://www.ebi.ac.uk/interpro/> |
| KEGG analysis | KAAS | v.2.0 <http://www.genome.jp/kaas-bin/kaas_main> |
|  | KEGG Mapper | [V2.5 http://www.kegg.jp/kegg/mapper.html](http://www.kegg.jp/kegg/mapper.html) |
| Subcellular localization | Wolfpsort | v.0.2 <http://www.genscript.com/psort/wolf_psort.html> |
|  | CELLO | v.2.5 <http://cello.life.nctu.edu.tw/> |
| Enrichment analysis | Perl module | v.1.31 <https://metacpan.org/pod/Text::NSP::Measures::2D::Fisher> |
| Hot-map | R Package pheatmap | v.2.0.3 <https://cran.r-project.org/web/packages/cluster/> |
| PPI network | Blast | v.2.2.26 <http://blast.ncbi.nlm.nih.gov/Blast.cgi> |
|  | R package networkD3 | v.0.4 <https://cran.r-project.org/web/packages/networkD3/> |
